# The Coriell personalized medicine collaborative pharmacogenomics appraisal, evidence scoring and interpretation system

**DOI:** 10.1186/gm499

**Published:** 2013-10-18

**Authors:** Neda Gharani, Margaret A Keller, Catharine B Stack, Laura M Hodges, Tara J Schmidlen, Daniel E Lynch, Erynn S Gordon, Michael F Christman

**Affiliations:** 1 The Coriell Institute for Medical Research, 403 Haddon Avenue, Camden, NJ 08103, USA; 2 Current Address: American Red Cross, 700 Spring Garden Street, Philadelphia, PA 19123, USA; 3 Current Address: Annals of Internal Medicine, 190 N. Independence Mall West, Philadelphia, PA 19106, USA

## Abstract

Implementation of pharmacogenomics (PGx) in clinical care can lead to improved drug efficacy and reduced adverse drug reactions. However, there has been a lag in adoption of PGx tests in clinical practice. This is due in part to a paucity of rigorous systems for translating published clinical and scientific data into standardized diagnostic tests with clear therapeutic recommendations. Here we describe the Pharmacogenomics Appraisal, Evidence Scoring and Interpretation System (PhAESIS), developed as part of the Coriell Personalized Medicine Collaborative research study, and its application to seven commonly prescribed drugs.

## Background

It has long been recognized that there is significant variability in drug response with respect to efficacy, optimal dose, and adverse drug reactions (ADRs). Pharmacogenomics (PGx), the study of the genes and genetic polymorphisms that influence variability in drug response, has the potential to both personalize and optimize drug therapy. Because of this potential for improvement in efficacy and for reduction in ADRs and their associated morbidity, mortality, and cost, there is increasing interest in integrating PGx into routine clinical care [1-9]. However, despite the many examples of causative links between genetic variations and substantial inter-individual differences in drug effects, and the fact that as many as 10% of labels for drugs approved by the Food and Drug Administration (FDA) contain PGx information [10], the development of validated diagnostic tests and the uptake of the PGx information by clinicians has been slow. The future success of PGx integration in personalized medicine will depend on a number of key factors, including 1) well-designed diagnostic tools that accurately identify all patients of different ancestral backgrounds who can benefit from the targeted therapies [10]; 2) a robust infrastructure for linking genetic test results (ideally available pre-emptively) and therapeutic recommendations to the drug-prescribing decision makers, for example, through the electronic medical record (EMR); and 3) an expansion of genomics and pharmacogenomics education programs for healthcare professionals so that they are sufficiently well-informed to use the information to manage their patients’ care.

Both the need for accurate standardized diagnostic tools and a robust infrastructure for linking genetics and therapeutic recommendations require a rigorous system for translating the published clinical and scientific data into clear drug-specific interpretations. Such a system should identify the genetic components that have sufficient data to support clinical or diagnostic utility, present evidence-based interpretations of genetic results in the context of particular drugs, provide clear recommendations for the application of specific results, and highlight areas with gaps in knowledge that need further investigation. The outcome of such a critical appraisal should guide further studies aimed both at addressing the specific gaps in knowledge about a gene’s effects on a specific drug (termed a ‘drug-gene pair’) and at validating further the predictive biomarkers, thus allowing therapeutics and diagnostics developers and regulators to make meaningful risk-benefit assessments that will pave the way to clinical adoption of the PGx guidelines [11]. This requires a multifaceted approach that includes routine integration of PGx in the design and outcomes analysis of clinical drug trials; retrospective studies that link patient health outcomes with medical/medication histories, gleaned through self-reported or EMR data [12,13]; and prospective, population-based, comparative effectiveness research [14,15].

The Coriell Personalized Medicine Collaborative (CPMC) has developed a systematic process for the critical appraisal, evidence scoring, and interpretation of PGx data (the Pharmacogenomics Appraisal, Evidence Scoring and Interpretation System; PhAESIS) to evaluate and address some of the current obstacles to PGx implementation highlighted above. This process was created in support of the ongoing CPMC study, an institutional review board-approved prospective observational study designed to evaluate the utility of personalized genomic information in health management. An overview of the CPMC project [16] and the CPMC approach to genetic risk estimation for health conditions [17] has been described elsewhere. Briefly, study participants with consent provide saliva samples for genotyping. Then, using a secure web-based interface, the CPMC provides participants with educational material, collects self-reported participant data (such as medical history, medication use, family history, lifestyle factors, and optional follow-up outcome surveys), and reports personalized results for potentially actionable health conditions and genetic results related to medication response. Genetic and self-reported data are used to conduct both replication and discovery genetic analyses, and to evaluate participant use of the results over time. The CPMC utilizes two independent Advisory Groups: the Pharmacogenomics Advisory Group (PAG), which provides guidance on PGx risk reporting, and the Informed Cohort Oversight Board (ICOB), which provides guidance on reporting to study participants of their risk for common complex diseases.

In order to comprehend the current validity and utility of published PGx data, to effectively interpret this information, and to return a clinically meaningful PGx risk report for the study participants, CPMC scientists identified the need to develop a systematic process for critically evaluating and translating published drug-specific PGx data for risk reporting. The method developed is drug-centric, utilizes a multi-tier evidence-based scoring procedure to define key genetic variants influencing variation in drug response, highlights gaps in knowledge, involves guidance by an external advisory committee, and presents drug–response interpretations for use in clinical reporting of genetic results. Here, we describe the CPMC PhAESIS system, designed to guide the development of the CPMC drug PGx risk reports, and its application to individual drug-gene pairs.

## Methods

**Figure 1:**
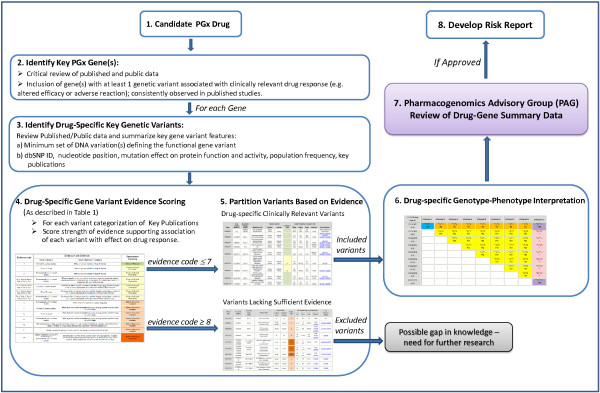
**Schematic representation of the Coriell Personalized Medicine Collaborative (CPMC) Pharmacogenomics Appraisal, Evidence-based Scoring and Interpretation System (PhAESIS) Procedure.** Candidate drugs for PGx reporting are identified and prioritized for the CPMC study based on a number of criteria (as listed in the Methods section). Once a drug is selected for evaluation, the FDA drug label, the peer-reviewed scientific and clinical literature, and public web-based databases are searched for studies that report drug-related genotype–phenotype associations (see Additional file 1: Table S1 for examples of resources). This initial search identifies genes with a significant influence on response to this drug, with at least one genetic variant that is significantly and consistently associated with a clinically relevant drug-response outcome (altered efficacy or adverse reaction). For each key PGx gene, drug-specific gene variant evidence scoring is carried out (as described in the Methods section) using the scale depicted in Table 1. Genetic variant evidence scores are used to partition variants based on potential clinical relevance. Scores of 7 or lower indicate a defined effect on drug response or clinical outcome, whereas those of 8 or higher represent a lack of or insufficient evidence for an effect. Once all of the genetic variants of potential clinical relevance (those with evidence codes ≤7) have been identified, the anticipated response of the diploid individual (who possesses two copies of the gene, one inherited from each parent) with each combination of inherited variants is defined, based on published clinical outcomes data. A Punnett square is used to represent distinct diploid individuals, each assigned a defined drug response phenotype. Curated data from steps 2 to 6 are prepared as PhAESIS summary documents for review by the CPMC Pharmacogenomics Advisory Group (PAG). If approved by the PAG, drug-specific risk reports are then developed and released to study participants.

The CPMC PhAESIS process broadly comprises six steps as described below (summarized in Figure 1). Curated data from steps 2 to 6 are prepared as PhAESIS summary documents for review by the CPMC Pharmacogenomics Advisory Group (PAG). If approved by the PAG, drug-specific risk reports are then developed and released to study participants.

### Candidate drug selection

Candidate drugs for PGx reporting are identified and prioritized for the CPMC study based on a number of criteria: 1) inclusion in the FDA label (Table of Pharmacogenomic Biomarkers in Drug Labels) [18]; 2) description in scientific or clinical publications or in relevant PGx databases, such as the Pharmacogenomics Knowledgebase (PharmGKB) [19] and the Cytochrome P450 Drug Interaction Table at Indiana University School of Medicine [20]; 3) clinical significance (effect and severity) of altered drug response (for example, a genetic result associated with a life-threatening ADR or presence of a relevant ‘black box’ warning on the FDA drug label); 4) potential actionability, as defined by the ability to alter prescribing practice (dosing or alternate therapy) or clinical management such as more frequent monitoring, to potentially mitigate risk of ADRs or to maximize drug efficacy; 5) national drug usage statistics [21,22]; and 6) CPMC cohort drug usage data (see Additional file 1: Table S1 for web-based PGx resources used for drug selection). Each of these parameters can be assessed and used to prioritize selection of a candidate drug or class of drugs for CPMC PhAESIS evaluation.

### Identification of key PGx gene(s) and drug-specific key genetic variants

Once a drug is selected for evaluation, the FDA drug label, the peer-reviewed scientific and clinical literature, and public web-based databases are searched for studies that report drug-related genotype–phenotype associations (see Additional file 1: Table S1 for examples of resources). This initial search is used to identify all genes in both the pharmacokinetic (PK) and pharmacodynamic (PD) pathways that have a significant effect on response to the drug. By definition, such genes have at least one genetic variant that is significantly and consistently associated with a clinically relevant drug response outcome (altered efficacy or ADR). Literature searches using PubMed are performed using search terms that include 1) the drug of interest AND ‘genetics OR pharmacogenetics OR pharmacogenomics’; 2) the drug and the gene of interest (for example, key genes in the PK/PD pathways); and 3) individual genetic variants or haplotypes of the gene of interest or the commonly used PGx ‘star nomenclature’ [23] for the variations in the gene. In addition, PGx-specific databases including PharmGKB [24], the Human Cytochrome P450 (CYP) Allele Nomenclature Committee web site [25], and others are reviewed for information on genetic variations and their drug–phenotype association.

Once identified, pharmacogenomic evidence for each drug-gene pair is summarized in the PhAESIS PAG submission document, including PK/PD evidence supporting an effect of the gene variant on protein function (for example, enzymatic activity and/or kinetics, plasma drug concentrations, measured difference in drug target response) and clinical outcome data supporting an association with adverse events or altered efficacy. This includes information on study design (such as, observational cohort, randomized controlled trial, or case–control design; and single study or meta-analysis), cohort size, and, for prospective studies of rare clinical outcomes, the numbers of observed events. In addition, and when available, estimates of relative effect (hazard ratios, relative risk, odds ratios) with confidence bounds are noted.

### Drug- specific gene variant evidence scoring

CPMC scientists review the identified publications and assess the strength of the evidence they present according to the scale depicted in Table 1. The evidence score, assigned to each variant in a given gene, consists of 14 categories, ranging from 1 (representing the strongest evidence; presence of consistent *in vivo* clinical data for the drug under review) to 14 (the weakest evidence; published evidence showing a lack of effect of the variant on drug response). The scoring mechanism takes into account multiple factors, starting with the study type.

**Table 1:** P**harmacogenomics gene variant evidence code assignments based on strength of evidence for a drug interaction phenotype**

**Evidence code**	**Evidence code definition**		**Assessment outcome**
	**Study category**	**Study objective/findings**	
1	Clinical outcomes studies	Consistent effect of genetic variant on drug of interest^a^	Clinically relevant
2	PK or PD study	Consistent effect of genetic variant on drug of interest^a^	Potential clinical relevance
3	Molecular/cellular functional studies	Consistent effect of genetic variant on drug of interest^a^	Potential clinical relevance
4n^,^ 4scd^,^ 4se^,^ 4ae^,^ 4ad, or 4dp	Molecular/cellular functional studies	^a^Consistent effect of genetic variant on *probe* drug (industry standard substrate used for evaluating enzyme function) *and* includes analysis of mutation type (based on the six categories defined in table footnote^b^)	Potential clinical relevance
5n^,^ 5scd^,^ 5se^,^ 5ae^,^ 5ad, or 5dp	Clinical outcomes studies	^a^Consistent effect of genetic variant on *another* drug(s) *and* includes analysis of the mutation type (based on the six categories defined in table footnote^b^)	Potential clinical relevance
6n^,^ 6scd^,^ 6se^,^ 6ae^,^ 6ad, or 6dp	PK or PD study	^a^Consistent effect of genetic variant on *another* drug(s) *and* includes analysis of the mutation type (based on the six categories defined in table footnote^b^)	Potential clinical relevance
7n^,^ 7scd^,^ 7se^,^ 7ae^,^ 7ad, or 7dp	Molecular/cellular functional studies	^a^Consistent effect of genetic variant on *another* drug(s) *and* includes analysis of the mutation type (based on the six categories defined in table footnote^b^)	Potential clinical relevance
8	Molecular/cellular functional studies	Effect of genetic variant on a *probe* drug *only*	Clinical relevance unknown
9	Clinical outcomes studies	Effect of genetic variant on *another* drug *only*, *or* drug-specific altered activity for *other* drugs, or inconsistent effect on drug of interest	Clinical relevance unknown
10	PK or PD study	Effect of genetic variant on *another* drug *only*, *or* drug-specific altered activity for *other* drugs, or inconsistent effect on drug of interest	Clinical relevance unknown
11	Molecular/cellular functional studies	Effect of genetic variant on *another* drug *only*, *or* drug-specific altered activity for *other* drugs, or inconsistent effect on drug of interest	Clinical relevance unknown
12	Clinical outcomes studies, PK or PD study, or molecular/cellular functional studies	Genotype frequency data suggestive of very rare or ‘private’ mutation, defined as a genetic variant found in a single individual or single family without being reported in reference populations	Clinical relevance unknown
13	Genetic variation screening studies, without additional functional or clinical studies	Insufficient data	Clinical relevance unknown
14	Clinical outcomes studies, PK or PD study, or molecular/cellular functional studies	Demonstrates no effect of the genetic variant on drug response. Includes variants that have evidence for association with drug response but attributed to linkage disequilibrium with another variant with defined function	Clinical relevance unsupported

*Abbreviations*: *PD* pharmacodynamics, *PK* pharmacokinetics.

^a^For evidence scores 1 to 7, the drug phenotype association should be consistent across different studies. In cases of discordant published data, the evidence is weighted based on study design and size, with larger studies and those that do not raise concerns about study methods (such as use of co-medications and genotype groupings that might skew the expected outcomes) carrying more weight. If consideration of study design does not resolve the observe inconsistencies, then a score of 9 to 11 is assigned, as appropriate.

^b^The six codes are: N, null mutation (abolishes function); scd, mutation located in known important substrate-binding or catalytic domain or in a highly evolutionarily conserved residue; se, mutation leading to splicing error/protein truncation (this can reduce or abolish function); ae, mutation leading to altered gene expression (this can reduce or increase protein function); ad, mutation leading to accelerated degradation of protein or mRNA (this can reduce or abolish function); *and* dp, gene duplication (this may increase protein function).

Studies are broadly categorized into study types A (greatest PGx evidence) to D (lowest PGx evidence), as follows.

A. Clinical outcomes studies. These studies show measurable difference in clinical endpoints such as side effects, rate of cure, morbidity, and mortality. Such studies demonstrate that the genetic variant significantly changes the medical outcome in response to the administered drug. Studies in this category can include clinical trials, cohort studies, case–control studies, case reports, and case series.

B. PK and PD studies. PK studies are defined as those that examine the effect of the genetic variant on the absorption, distribution, metabolism, or elimination of the drug. In these studies, the genetic variant is associated with variability in the level or concentration of the drug and its metabolites at the site of action. PD studies are defined as those that examine genetic variants in the drug targets showing a measureable difference in the biomarker’s response to the drug. Although the measured variables (biomarkers) may be considered as surrogates for a clinical response, they cannot be translated directly to clinical outcomes as the effect on clinical outcomes may be insufficiently significant to alter practice or policy. These studies include *in vivo* or *ex vivo* studies that measure PK or PD responses to a given drug, and may include clinical trials, cohort studies, case–control studies, and case reports and case series.

C. Molecular and cellular functional studies. These studies use *in vitro* functional assays to examine how the genetic variant alters the function of the enzyme or protein or the whole cell. For example, such studies might evaluate the effect of the variant on enzyme kinetics, gene activation, and expression or alteration of specific cellular properties involved in the response to a drug.

D. Genetic variation screening studies. These include studies in which the PGx gene variant was identified through DNA sequencing analysis or other genetic analysis, either in control or patient populations, without any additional functional or clinical studies to support a functional role for the variant.

Thus, the greatest support comes from clinical outcomes data (A), followed by PK/PD data (B), followed by *in vitro* molecular and/or cellular functional data (C), with the lowest evidence coming from genetic variation screening studies (D). Evidence scores (1 to 14) further differentiate between an effect of the genetic variant directly on the drug under review or indirectly on another drug (such as, another drug or probe substrate; an industry standard used to evaluate activity of specific P450 enzymes [26]) (Table 1). If direct evidence is available for the drug under review, this is considered a significant association with the phenotype, regardless of the level of knowledge about the function of the variant. However, if only indirect evidence is available for a given genetic variation, then the evidence is supplemented based on the variation or mutation type having specific examples assumed to show a sufficiently broad effect on the function of the protein to allow extrapolation to an effect on all drugs (such as, a null mutation that abolishes the protein function; see Table 1 for mutation types included). For example, evidence codes 1, 5, and 9 are all based on clinical outcomes data. However, code 1 is for a direct effect of the variant on the drug under review, whereas codes 5 and 9 are indirect evidence for an effect on another drug(s). The difference between codes 5 and 9 is that variants with code 5 are expected to have a universal effect on drug response based on mutation type, whereas the broader effect of those with code 9 is either unknown or unsupported based on the type or location of the mutation, or based on the observed variable or drug-specific effects. Once all the evidence for a genetic variant is gathered, a single score is assigned to each variant based on the greatest strength of evidence (that is, the lowest evidence code number).

For evidence scores 1 to 7, the drug–phenotype association should be consistent across different studies. However, given the variability in study size and quality in the published literature, evidence may be weighted in favor of larger studies and on those that do not raise concerns about study methods and design (such as use of co-medications and genotype groupings that might skew the expected outcomes). If consideration of study quality resolves apparent inconsistencies, then a score of 1 to 7 is assigned. If the data are inconclusive, the clinical relevance of the variant is unknown, and a score of 9 to 11 is returned, as appropriate.

### Identification of drug-specific genetic variants of potential clinical relevance

Genetic variant evidence scores are used to partition variants based on potential clinical relevance. Gene variant evidence scores of less than 7 indicate a defined effect on drug response or clinical outcome, whereas those greater than 8 represent a lack of or insufficient evidence for an effect (Table 1). Within the former group of variants with defined effect on drug response, those with evidence code 1 include variants that have validated clinical evidence to support their effect (that is, they are considered clinically relevant); those with evidence codes 2 to 7 lack clinical outcomes data but have been found to have a measurable difference in drug response (they are potentially clinically relevant). Variants with evidence codes 8 to 14 are those that either have limited or inconsistent data for response to other drug(s) (clinical relevance unknown), or lack supportive data for response to the drug under review (clinical relevance unsupported). An evidence score of 8 or higher also highlights possible gaps in scientific or clinical data. This group is typically enriched for variants rare in Caucasian populations or those with undefined effect on the protein function.

For each gene, drug-specific gene variant summary tables are prepared, and included in the PAG submission document. These include information on the minimum set of DNA variations (such as single nucleotide polymorphisms (SNPs), insertion/deletions, or copy number variations) defining the functional gene variant (such as, haplotype or star allele). For each variation, the reference SNP ID number (rs#), the nucleotide change, mutation effect, and variant frequency in populations of Caucasian, East Asian, and African descent are recorded. In addition, for each gene variant, the effect on protein function and activity, the associated metabolic or PK/PD phenotype, and the strength of evidence score of the variant-response association, is provided. See Additional file 1: Tables S3, S6, S9, S11, S13, S15, S20, S23, and S27 for examples of the gene variant summaries in the context of different drugs.

### Drug-specific genotype-phenotype interpretation

Once all of the genetic variants of potential clinical relevance (those with evidence codes of 7 or higher) have been identified, the anticipated response of the diploid individual (who possesses two copies of the gene, one inherited from each parent) with each combination of inherited variants, is defined. If published guidelines based on empirical data are available, these will be used to classify diplotypes to specific drug response groups. For example, the *CYP2D6* metabolizer type or activity level for an individual is predicted based either on their highest functioning *CYP2D6* allele [27,28] or on an allele/genotype scoring approach [29-32]. If specific guidelines are not available, assignment of drug response phenotype is based on observations from published clinical outcomes data for the specific drug-gene pair. In some cases where there is a lack of published data for a specific diplotype, the predicted phenotype will be unknown. Likewise, if there are ambiguities in the published data, either due to inconsistent observations or due to consistent data with an effect in the opposite direction to that expected, an unknown clinical phenotype is assigned to the diplotype. All of these examples of data ambiguity (discordant or insufficient published data) highlight gaps in knowledge for further study.

In cases of rare observations where data exists for other alleles of similar effect, the phenotype for a given diplotype is extrapolated based on the general rules for other similar variants/genotypes. For example, CYP2C19*6 is a rare variant that results in negligible catalytic activity towards the universal *CYP2C* substrate tolbutamide, and is classified as a reduced activity variant similar to CYP2C19*2 and CYP2C19*3. Diplotypes carrying CYP2C19*6 are therefore assumed to have a similar response to clopidogrel as those with the CYP2C19*2 variant.

**Figure 2:**
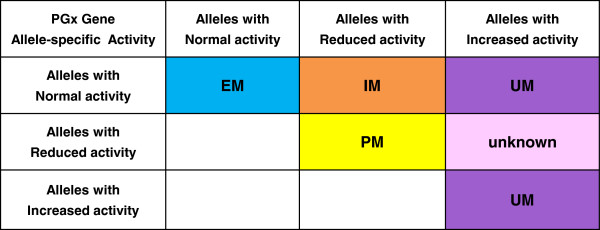
**Example of a simple Punnett square providing general rules for assignment of predicted drug-specific response phenotypes for a particular drug-metabolizing pharmacogenomics (PGx) gene.** Annotation of pharmacokinetics/pharmacodynamics (PK/PD) outcome*:* EM, extensive metabolizers; IM, intermediate metabolizers; PM, poor metabolizers; UM, ultra-rapid metabolizers; unknown, drug metabolizing phenotype currently unknown. Note that for other types of PGx genes such as drug transporters, the PK/PD phenotype can be similarly annotated in the Punnett square table, for example as ‘normal’ for normal transport; ‘decreased’ for reduced transport; and ‘increased’ for increased transport. Annotation of the associated clinical outcome: shades of blue indicate ‘normal’ response to the drug; yellow, most extreme adverse drug reaction (ADR) or altered efficacy resulting from deficiency or reduced function/activity of the PGx protein product; orange, clinically distinct or milder adverse drug reaction/altered efficacy resulting from protein deficiency or reduced function; purple, distinct ADR or altered efficacy resulting from excess or increased function of the protein product; pink, unknown phenotype for the defined diplotype. The group of diplotypes with unknown phenotype represent a gap in knowledge where further research is warranted.

As part of the data curation process, a Punnett square is used to represent distinct diploid individuals, each assigned with a defined drug response phenotype. A ‘simple’ Punnett square is first constructed to provide the general rules used for interpretation of genotype-drug response phenotype (Figure 2). These rules are then extended to a ‘full’ Punnett square that includes all the potential diplotypes for the drug-gene pair under review. Drug response phenotypes represented in the Punnett square tables include both PK/PD and clinical response data. In cases where the data for specific diplotypes are ambiguous (discordant or insufficient published data) the phenotype assignment will be ‘unknown’ (Figure 2). In cases of genetic variation in genes that encode drug-metabolizing enzymes, the phenotype is based both on a ‘metabolizer’ type and on the drug-specific clinical outcome (as it relates to efficacy or ADR) that is associated with the particular genotype. Similarly, for a drug transporter gene, the genotype associated phenotype may be decreased, normal, or increased transport, which may be associated with reduced efficacy and/or risk of an ADR. It is also important to note that the predicted PK/PD phenotype for a specific diplotype may vary by drug. For example, for the drug-metabolizing enzyme CYP2C19, the CYP2C19*1/*17 diplotype is associated with an unknown metabolizer status (unk) with respect to proton pump inhibitors (PPIs), but an ultra-rapid metabolizer (UM) phenotype with respect to clopidogrel (see Additional file 1: Tables S4 and S7).

### The CPMC PAG

The CPMC PAG, founded in 2010, is an expert advisory panel of pharmacists, geneticists, a bioethicist, pharmacologists, and clinicians with experience in PGx (see the CPMC advisory board web site [33] for PAG membership). The group, made up of a chair and associate members, meets at least once per year to review PhAESIS documents, and advise the CPMC scientific study team on whether and how to incorporate each drug-gene pair under consideration into the CPMC study.

PhAESIS documents, submitted to the PAG for review, comprise a detailed appraisal of the drug and drug-specific PGx, and provide a summary of curated data from steps 2 to 6 (above and in Figure 1). Each drug-specific document includes a description and mechanism of action of the drug, summary of the key publications supporting the PGx evidence, and the PhAESIS evidence scoring of key genetic variants, drug-specific genotype-phenotype correlations and predicted drug response interpretations. If for a given drug-gene pair, there are inconsistencies in the published data (for the gene or specific variants) that render the data inconclusive, the CPMC may still choose to include these in the PhAESIS report, and to seek guidance from the PAG on the validity of the data.

Functioning in a similar way to a standard study section, the PAG discusses the content of the PhAESIS document, with commentary by primary and secondary reviewers. The members discuss the material in a group setting, with the opportunity to question the CPMC scientific team. After discussion, the PAG votes on whether and how to include the drug-gene pair in the CPMC. The PAG advises the CPMC study on: 1) what PGx relevant drug-gene information is sufficiently valid and has potential clinical utility (is at a minimum potentially clinically relevant), and is therefore worthy of release to study participants as personalized PGx results; and 2) defining whether and what genetic results for a particular drug-gene pair constitute a level of drug response ‘actionability’ that may obligate a different (more urgent) communication path to participants. For example, study participants who are predicted to have diminished effectiveness of clopidogrel due to *CYP2C19* poor metabolizer status would warrant a higher level of messaging via the web portal than would extensive metabolizers. This phenotype-specific messaging ensures that such participants have made an informed decision regarding whether or not to view their results. This approach is faithful to the study premise and original consent, which leaves to each study participant the decision to view, or not view, each personal genetic result.

### CPMC study PGx risk reporting

Once approved by the PAG, the drug-gene specific genotype-phenotype assignments summarized in the extended Punnett square table (see Additional file 1: Tables S4 and S7) are used to develop personalized PGx risk reports for release to CPMC participants. The process for developing PGx reports falls outside the scope of the PhAESIS system described here, and will not be detailed in this paper. Briefly, the CPMC develops drug-specific genotype translation tables and information technology infrastructure for dynamically extracting personal data (genetic results for multiple variants, and relevant demographics) from the project database in order to generate personalized risk reports. PGx risk reports are delivered through the secure CPMC web portal (example reports can be viewed at the CPMC web site [34]) Risk reports provided to CPMC participants contain PGx genetic results, result interpretation, educational summaries, detailed information on genetic and non-genetic risk factors affecting drug response, and the range and frequency of drug response phenotypes in the population most relevant to the participant (Caucasian, African, or East Asian ancestry). Limitations, methods, and links to external resources are also provided through the web-based report. To aid participants’ understanding, the CPMC provides them with access to free counseling by board-certified genetic counselors and pharmacists.

## Results

To date the PAG has reviewed PhAESIS reports on more than 11 drugs and 18 associated PGx genes. Seven drugs/drug class and nine associated genes have been approved for PGx reporting to CPMC participants (clopidogrel and *CYP2C19*; warfarin and *CYP2C9*/*VKORC1*/*CYP4F2*; PPIs and *CYP2C19*; codeine and *CYP2D6*; thiopurines and *TPMT*; simvastatin and *SLCO1B1*; celecoxib and *CYP2C9*). One drug-gene pair (tamoxifen and *CYP2D6*) has been deferred pending more data, and three have been rejected for various reasons, including insufficient clinical data or lack of evidence for clinical utility. A summary of the PAG review outcomes is provided in Table 2. The deferral of a decision on tamoxifen and *CYP2D6* is an example of how the PAG can provide expert guidance in situations where the published data are ambiguous. The large body of data evaluating the prognostic and predictive relevance of *CYP2D6* gene testing to guide tamoxifen therapy for breast cancer was inconsistent, and the CPMC chose to present these to the PAG for specific guidance. After deliberation, the PAG deemed the data inconclusive, and given that data from a large clinical study was anticipated (the International Tamoxifen Pharmacogenomics Consortium (ITPC) [35]), the vote on this drug-gene pair was deferred pending that publication.

**Table 2:** Summary of the Coriell Personalized Medicine Collaborative drug-gene pharmacogenomics reports evaluated by the Pharmacogenomics Advisory Group

**Drug and gene pair(s)**	**Approval status**	**Reason for rejection/deferral**	**Highly actionable genetic results**^**a**^
**Warfarin and *VKORC1*, *CYP2C9*, *CYP4F2*, or *GGCX***	All except *GGCX* approved	Insufficient clinical support for *GGCX* at time of submission evaluation	Low warfarin dose requirement^b^
**Clopidogrel and *CYP2C19***	Approved	NA	Poor metabolizers
**Tamoxifen and *CYP2D6***	Deferred	The data linking prognostic and predictive relevance of *CYP2D6* variants to guide tamoxifen therapy for breast cancer was inconclusive. Vote has been deferred pending publication of anticipated clinical trials data	Vote deferred
**Codeine and *CYP2D6***	Approved	NA	Ultra-rapid metabolizers
**Metoprolol and *CYP2D6***	Rejected	Lack of clinical evidence, and given prescribing practices, the genetic results are unlikely to influence drug dose adjustment	NA
**Thiopurines and *TPMT***	Approved	NA	Intermediate and poor metabolizers
**PPIs and *CYP2C19***	Approved	NA	None
**Diazepam and *CYP2C19***	Rejected	Evidence for clinical consequences is weak	NA
**Statins and *CYP3A4/CYP3A5*, *SLCO1B1*, *LDLR*, or *HMGCR***	Simvastatin and *SLCO1B1*: approved, *CYP3A4*/*CYP3A5*: deferred. *LDLR* and *HMGCR*: rejected	*LDLR* and *HMGCR*: evidence for clinical utility is lacking	Simvastatin and *SLCO1B1*: genetic results associated with decreased hepatic drug uptake
**Celecoxib and *CYP2C9***	Approved	NA	*3/*3
**Fluorouracil and *DPYD***	Rejected	Combination of recent evidence for lower penetrance of reduced activity variants, and a lack of good alternative treatment reduces the clinical utility of the PGx information	NA

*Abbreviations*: *NA* not applicable.

^a^Genetic results that warrant additional communication to participants who are at increased risk, and who have not viewed their risk report.

^b^‘Low dose’ is defined as a daily therapeutic dose of 0.5 to 2 mg based on the FDA drug label genotype guidelines table.

Drug response interpretations for the PAG-approved drug-gene pairs are summarized (Tables 3, 4, 5, 6, 7, 8, and 9). These tables include gene variants with evidence scores of 7 or higher defined during the PhAESIS evaluation of each drug-gene pair (see Additional file 1: Sections 1 to 7 for abbreviated curated data from PAG submissions, including variant summary tables, genotype-phenotype interpretation Punnett squares, and PGx evidence for each of the approved drug-gene pairs). The tables include genetic results with validated clinical evidence (Tables 3, 4, 5, 6, 7, 8, and 9), considered to be clinically relevant (those that include variants with evidence code 1) (see Additional file 1: Tables S3, S6, S9, S11, S13, S15, S20, S23, and S27). Genetic results that include variants with evidence codes 2 to 7 (potentially clinically relevant) are also included (Tables 3, 4, 5, 6, 7, 8, and 9); however, these require further validation to support their inclusion for clinical reporting. For each of the drug-specific genotype categories, both the PK/PD phenotype and the associated clinical phenotype (drug response outcome and interpretation) are provided. If specific FDA PGx guidelines are available for the drug-gene category, these are indicated along with the expected population frequencies for Caucasian, African, and East Asian ancestries.

**Table 3:** **Genotype-phenotype drug response interpretations of the Pharmacogenomics Advisory Group-approved drug-gene pair clopidogrel and *CYP2C19***^**a**^

**Common diplotypes**^**b**^	**Expected population frequency, %**^**c**^			**PK/PD phenotype**	**Clinical phenotype**	**FDA guidelines**
	**Caucasian ancestry**	**African ancestry**	**East Asian ancestry**			
CYP2C19*1/*1	38	36	35.5	EM: normal enzymatic function and normal drug activation; normal platelet inhibition	Likely to have a normal response to standard dose of clopidogrel	–
CYP2C19*1/*2, CYP2C19*1/*8, and other rare diplotypes (see Additional file 1: Table S4)	19	22	46	IM: reduced enzymatic function resulting in reduced drug activation; decreased platelet inhibition	Increased risk of ischemic event while on clopidogrel.^d^ Should use alternative anti-platelet medication	–
CYP2C19*2/*2 and other rare diplotypes (see Additional file 1: Table S4)	2	3	15	PM: greatly reduced or abolished enzymatic function, leading to little or no drug activation; greatly diminished platelet inhibition	Increased risk of ischemic event while on clopidogrel.^d^ Should use alternative anti-platelet medication	*CYP2C19* PMs with ACS or undergoing PCI treated with Plavix at recommended doses exhibit higher cardiovascular event rates than do patients with normal *CYP2C19* function. Consider alternative treatment or treatment strategies in patients identified as *CYP2C19* PMs
CYP2C19*1/*17, CYP2C19*17/*17	34	31	2	UM: enhanced enzymatic function leading to greater drug activation	Possible increased risk of bleeding; but also likely to derive greater protection from ischemic event while on clopidogrel	–
CYP2C19*2/*17 and other rare diplotypes (see Additional file 1: Table S4)	7	8	1.5	Unk: metabolizer status undetermined and therefore unknown. PD data indicates platelet response is intermediate between likely IMs and EMs	Unknown effect on drug response	–

*Abbreviations*: *ACS* acute coronary syndromes, *EM* Extensive metabolizer, *FDA* Food and Drugs Administration, *IM* intermediate metabolizer, *PCI* percutaneous coronary intervention, *PD* pharmacodynamic, *PK* pharmacokinetic, *PM* poor metabolizer, *UM* Ultra-rapid metabolizer, *Unk* metabolizer status unknown.

^a^Supporting evidence may be found in Additional file 2: Section S1.0-1.7, and Additional file 1:Tables S2-S4. These include summaries of the PhAESIS evaluation and referenced publications supporting the drug-gene clinical phenotypes.

^b^Diplotypes with frequencies of less than 0.4% in Caucasians are included above. Other rare diplotypes that fall under the same phenotype category can be found in the genotype-phenotype Punnett table (see Additional file 1: Table S4). Diplotypes above and in the genotype-phenotype Punnett table include both clinically validated genetic results (those that include variants with evidence code 1) and results that include variants with evidence scores 2 to 7 (potentially clinically relevant). The latter require further validation to support their inclusion for clinical reporting.

^c^Population frequencies are estimated based on reported gene variant allele frequencies (see Additional file 1: Table S3) and Hardy-Weinberg principles.

^d^Greater risk of ischemic event in patients with ACS undergoing PCI.

**Table 4:** **Genotype-phenotype drug response interpretations of the Pharmacogenomics Advisory Group-approved drug-gene pairs proton pump inhibitors and *CYP2C19***^**a**^

**Common diplotypes**^**b**^	**Expected population frequency, %**^**c**^			**PK/PD phenotype**	**Clinical phenotype**	**FDA guidelines**
	**Caucasian ancestry**	**African ancestry**	**East Asian ancestry**			
CYP2C19*1/*1	38	35	35	EM: normal enzymatic function and normal drug elimination	Likely to have normal response to standard dose of PPIs	–
CYP2C19*1/*2, CYP2C19*1/*8, CYP2C19*2/*17, and other rare diplotypes (see Additional file 1: Table S7)	26	31	48	IM: reduced enzymatic function leading to reduced drug elimination and greater drug exposure	Both IM and PM likely to have improved PPI efficacy at standard dose of PPI as measured by intragastric pH, duration of inhibition, and cure rates for GERD and *Helicobacter pylori*	–
CYP2C19*2/*2 and other rare diplotypes (see Additional file 1: Table S7)	2	3.5	15	PM: greatly reduced or abolished enzymatic function, leading to reduced drug elimination and greater drug exposure		–
CYP2C19*17/*17	5	4.5	0.04	UM: enhanced enzymatic function leading to greater drug elimination and reduced drug exposure	Decreased PPI efficacy at standard doses	–
CYP2C19*1/*17	29	26	2	Unk: metabolizer status undetermined and therefore unknown; PD data shows platelet response is intermediate between IMs and EMs	Unknown effect on drug response	

*Abbreviations*: *EM* Extensive metabolizer, *FDA* Food and Drugs Administration, *GERD* gastroesophageal reflux disease, *IM* intermediate metabolizer, *PD* pharmacodynamic, *PK* pharmacokinetic, *PM* poor metabolizer, *PPI* proton pump inhibitors, *UM* Ultra-rapid metabolizer, *Unk* metabolizer status unknown.

^a^Supporting evidence may be found in Additional file 2: Section S2.0-2.7, and Additional file 1: Tables S5 and S7. These include summaries of the PhAESIS evaluation and referenced publications supporting the drug-gene clinical phenotypes.

^b^Diplotypes with frequencies of less than 0.4% in Caucasians are included above. Other rare diplotypes that fall under the same phenotype category can be found in the genotype-phenotype Punnett table (see Additional file 1: Table S7). Diplotypes above and in the genotype-phenotype Punnett table include both clinically validated genetic results (those that include variants with evidence code 1) and results that include variants with evidence scores 2 to 7 (potentially clinically relevant). The latter require further validation to support their inclusion for clinical reporting.

^c^Population frequencies are estimated based on reported gene variant allele frequencies (see Additional file 1: Table S6) and Hardy-Weinberg principles.

**Table 5:** **Genotype-phenotype drug response interpretations of the Pharmacogenomics Advisory Group-approved drug-gene pair celecoxib and *CYP2C9***

**Common diplotypes**^**b**^	**Expected population frequency, %**^**c**^			**PK/PD phenotype**	**Clinical phenotype**	**FDA guidelines**
	**Caucasian ancestry**	**African ancestry**	**East Asian ancestry**			
CYP2C9*1/*1	67	84.5	92	EM: normal enzymatic function and drug elimination	Expected to have a normal analgesic response at standard dose of celecoxib. Colorectal adenoma treatment: no additional efficacy with 400 mg celecoxib twice daily compared with 200 mg twice daily	–
CY2C9*1/*2, CYP2C1/*3, and other rare diplotypes (see Additional file 1: Table S10)	30	15	8	Likely IM: reduced enzymatic function and drug elimination, leading to increased drug exposure	Insufficient data; predicted risk of side effects is unknown	–
CYP2C9*3/*3, CYP2C*2/*3, CYP2C*2/*2, and other rare diplotypes (see Additional file 1: Table S10)	3	0.6	0.2	PM: greatly reduced enzymatic function and drug elimination, leading to greater drug exposure	Greater risk of adverse cardiovascular events with 400 mg celecoxib twice daily. Colorectal adenoma treatment: decreased recurrence with 400 mg celecoxib twice daily	Consider 50% of the standard starting dose in PMs; consider alternative treatment in PMs with juvenile rheumatoid arthritis

*Abbreviations*: *EM* Extensive metabolizer, *FDA* Food and Drugs Administration, *IM* intermediate metabolizer, *PD* pharmacodynamic, *PK* pharmacokinetic, *PM* poor metabolizer.

^*a*^Supporting evidence may be found in Additional file 2: Section S3.0 to 3.7 and Additional file 1: Tables S8 to S10. These include summaries of the PhAESIS evaluation and referenced publications supporting the drug-gene clinical phenotypes.

^b^Diplotypes with frequencies of less than 0.4% in Caucasians are included above. Other rare diplotypes that fall under the same phenotype category can be found in the genotype-phenotype Punnett table (see Additional file 1: Table S10). Diplotypes above and in the genotype-phenotype Punnett table include both clinically validated genetic results (those that include variants with evidence code 1) and results that include variants with evidence scores 2 to 7 (potentially clinically relevant). The latter require further validation to support their inclusion for clinical reporting.

^c^Population frequencies are estimated based on reported gene variant allele frequencies (see Additional file 1: Table S9) and Hardy-Weinberg principles.

**Table 6:** **Genotype-phenotype drug response interpretations of the Pharmacogenomics Advisory Group-approved drug-gene pairs: warfarin and *CYP2C9/VKORC1/CYP4F2***^**a**^

**Common diplotypes**^**b**^	**Expected population frequency, %**^**c**^			**PK/PD phenotype**	**Clinical phenotype**	**FDA guidelines**
	**Caucasian ancestry**	**African ancestry**	**East Asian ancestry**			
Warfarin and *CYP2C9*						
CYP2C9*1/*1	67	76	88	EM: normal enzymatic function and normal drug elimination	Expected to have a normal response at standard dose of warfarin	The warfarin drug label includes a table of *CYP2C19* and *VKORC1* genotype-based therapeutic dosing guidelines, which provides the range of expected therapeutic doses based on genotype combinations (based on *VKORC1* -1639G > A (rs9923231) and CYP2C9*2 and CYP2C9*3 variants) (see Additional file 1: Table S17)
CYP2C9*1/*2, CYP2C9*1/*14 and other rare diplotypes (see Additional file 1: Table S12)	20	17	4	IM: reduced enzymatic function, leading to reduced drug elimination and greater drug exposure	At increased risk of bleeding at standard dose of warfarin.^**d**^. Takes longer to reach therapeutic INR; requires lower dose of warfarin	
CYP2C9*1/*3, CYP2C*2/*2, CYP2C*2/*3, CYP2C*3/*3, and other rare diplotypes (see Additional file 1: Table S12)	13	7	8	PM: greatly reduced enzymatic function, leading to reduced drug elimination and greatly increased drug exposure	At increased risk of bleeding at standard dose of warfarin.^**d**^ Takes longer to reach therapeutic INR; requires lower dose of warfarin	
Warfarin and *VKORC1*						
−1639G > A (rs9923231): GG	40	74	1	Normal mRNA expression; normal enzyme activity and efficient vitamin K cycling	Associated with a requirement for higher therapeutic warfarin dose.^**d**^ Potentially at increased risk of thrombosis at standard dose	
−1639G > A (rs9923231): GA	47	24	20	Reduced mRNA expression; reduced enzyme activity and vitamin K cycling	Associated with a requirement for intermediate therapeutic warfarin dose^**d**^	
−1639G > A (rs9923231): AA	13	2	79	Greatly reduced mRNA expression level; significant reduction in enzyme activity and vitamin K cycling	Associated with a requirement for lower therapeutic warfarin dose.^**d**^ Potentially at increased risk of bleeding events	
Warfarin and *CYP4F2*						
1297G > A, *CYP4F2* V433M: GG	53	83	63	Higher *CYP4F*2 activity results in reduced hepatic vitamin K levels	Associated with a requirement for lower therapeutic warfarin dose.^**d**^ Potentially at increased risk of bleeding events	–
1297G > A, *CYP4F2* V433M: GA	40	16	33	Intermediate *CYP4F2* activity results in intermediate hepatic vitamin K levels	Associated with a requirement for intermediate therapeutic warfarin dose^**d**^	–
1297G > A, *CYP4F2* V433M: AA	7	1	4	Reduced *CYP4F2* activity results in increased hepatic vitamin K levels	Associated with a requirement for higher therapeutic warfarin dose.^**d**^ Potentially at increased risk of thrombosis at standard dose	–

*Abbreviations*: *EM* Extensive metabolizer, *FDA* Food and Drugs Administration, *IM* intermediate metabolizer, *INR* international normalized ratio, *PD* pharmacodynamic, *PK* pharmacokinetic, *PM* poor metabolizer.

^a^Supporting evidence may be found in Additional file 2: Section S4.0 to 4.7 and Additional file 1: Tables S11 to S17. These include summaries of the PhAESIS evaluation and referenced publications supporting the drug-gene clinical phenotypes.

^b^Diplotypes with frequencies of less than0.4% in Caucasians are included above. Other rare diplotypes that fall under the same phenotype category can be found in the genotype-phenotype Punnett tables (see Additional file 1: Tables S12, S14 and S16). Diplotypes above and in the genotype-phenotype Punnett tables include clinically validated genetic results (those that include variants with evidence code 1).

cPopulation frequencies are estimated based on reported gene variant allele frequencies (see Additional file 1: Table S11, S13 and S15) and Hardy-Weinberg principles.

^d^Empiric starting doses range from 3 to 5 mg/day [36,37]; FDA therapeutic dosing guideline table ranges: low dose (0.5 to 2 mg/day), intermediate dose (3 to 4 mg/day) and high dose (5 to 7 mg/day).

**Table 7:** **Genotype-phenotype drug response interpretations of Pharmacogenomics Advisory Group-approved drug-gene pair: Codeine and *CYP2D6***^**a**^

**Common diplotypes**^**b**^	**Expected population frequency, %**^**c**^			**PK/PD phenotype**	**Clinical phenotype**	**FDA guidelines**
	**Caucasian** **ancestry**	**African ancestry**	**East Asian ancestry**			
CYP2D6*1/*1, CYP2D6*1/*3, CYP2D6*1/*4, CYP2D6*1/*5, CYP2D6*1/*6, CYP2D6*1/*7, CYP2D6*1/*9, CYP2D6*1/*10, and other rare diplotypes (see Additional file 1: Table S21)^d^	80	69	45	EM: normal enzymatic function and normal conversion of codeine to morphine	Normal analgesic response to standard dose of codeine	–
CYP2D6*4/*41, CYP2D6*4/*9, CYP2D6*4/*10, CYP2D6*41/*41, CYP2D6*5/*41, CYP2D6*9/*41, CYP2D6*10/*41, and other rare diplotypes (see Additional file 1: Table S21)	12	26	26	IM; reduced enzymatic function, leading to reduced conversion of codeine to morphine	Reduced analgesic response (pain relief). May require an increased dose to obtain an analgesic effect or should consider alternative pain medication	–
CYP2D6*3/*4, CYP2D6*4/*4, CYP2D6*4/*5, CYP2D6*4/*7 and other rare diplotypes (see Additional file 1: Table S21)	8	3	14	PM: greatly reduced or abolished enzymatic function, leading to greatly reduced conversion of codeine to morphine	Little or no analgesic response (pain relief). Should consider alternative pain medication	–
Rare in Caucasians. The following are common in East Asians: CYP2D6*1/*1 × N, CYP2D6*1 × N/*1 × N, CYP2D6*1 × N/*4, CYP2D6*1 × N/*5, CYP2D6*1 × N/*10, CYP2D6*1 × N/*10C(*36)	<0.1	2	15	UM: enhanced enzymatic function, leading to greater conversion of codeine to morphine and higher drug exposure	Increased risk of drug toxicity and ADRs. Should consider alternative pain medication	*CYP2D6* PMs and UMs may experience different efficacy. Even at labeled dosage regimens, UMs may experience overdose symptoms. Use of codeine by UM mothers can potentially lead to serious ADRs, including death, in nursing infants

*Abbreviations*: *ADR* adverse drug reaction, *EM* Extensive metabolizer, *FDA* Food and Drugs Administration, *IM* intermediate metabolizer, *PD* pharmacodynamic, *PK* pharmacokinetic, *PM* poor metabolizer, *UM* Ultra-rapid metabolizer.

^a^Supporting evidence may be found in Additional file 2: Section S5.0 to 5.7 and Additional file 1: Tables S19 to S21. These include summaries of the PhAESIS evaluation and referenced publications supporting the drug-gene clinical phenotypes.

^b^Diplotypes with frequencies of less than 0.4% in Caucasians are included above. Other rare diplotypes that fall under the same phenotype category can be found in the genotype-phenotype Punnett table (see Additional file 1: Table S21). Diplotypes above and in the genotype-phenotype Punnett table include both clinically validated genetic results (those that include variants with evidence code 1) and results that include variants with evidence scores 2 to 7 (potentially clinically relevant). The latter require further validation to support their inclusion for clinical reporting.

^c^Population frequencies are estimated based on reported gene variant allele frequencies (see Additional file 1: Table S20) and Hardy-Weinberg principles.

^d^CYP2D6*1 denoted in the above table represents either the *1 or *2 allele with normal *CYP2D6* activity.

**Table 8:** **Genotype-phenotype drug response interpretations of the Pharmacogenomics Advisory Group-approved drug-gene pairs: thiopurines and *TPMT***^**a**^

**Common Diplotypes**^**b**^	**Expected population frequency, %**^**c**^			**PK/PD phenotype**	**Clinical phenotype**	**FDA guidelines**
	**Caucasian ancestry**	**African ancestry**	**East Asian ancestry**			
TPMT*1/*1	94	81	97	EM: normal enzymatic function and normal drug elimination	Expected to respond to a standard dose of thiopurine drugs. Not at increased risk of drug toxicity	–
TPMT*1/*2, TPMT*1/3A, and other rare diplotypes (see Additional file 1: Table S24)	6	18	3	IM: reduced enzymatic function, leading to reduced drug elimination and greater drug exposure	At increased risk of drug toxicity such as myelosuppression when taking standard dose of thiopurine drugs. Risk of side effects can be reduced by reducing standard dose by 50 to 70%	Heterozygous patients with low or intermediate TPMT activity are more likely to experience toxicity
Rare in Caucasians, The following are more common in African ancestry: TPMT*3A/*3A, TPMT*3B/*3B	0.1	1	0	PM: very low or absent enzymatic function, leading to greatly reduced drug elimination and increased drug exposure	At increased risk of drug toxicity such as myelosuppression when taking thiopurine drugs. Should consider alternative medication	Homozygous-deficient patients (two non-functional *TPMT* alleles) given usual dose of thiopurines are at increased risk of toxicity

*Abbreviations*: *EM* Extensive metabolizer, *FDA* Food and Drugs Administration, *IM* intermediate metabolizer, *PD* pharmacodynamic, *PK* pharmacokinetic, *PM* poor metabolizer.

^a^Supporting evidence may be found in Additional file 2: Section S6.0 to 6.7 and Additional file 1: Tables S22 to S24. These include summaries of the PhAESIS evaluation and referenced publications supporting the drug-gene clinical phenotypes.

^b^Diplotypes with frequencies of less than 0.4% in Caucasians are included above. Other rare diplotypes that fall under the same phenotype category can be found in the genotype-phenotype Punnett table (see Additional file 1: Table S24). Diplotypes above and in the genotype-phenotype Punnett table include both clinically validated genetic results (those that include variants with evidence code 1) and results that include variants with evidence scores 2 to 7 (potentially clinically relevant). The latter require further validation to support their inclusion for clinical reporting.

^a^Population frequencies are estimated based on reported gene variant allele frequencies (see Additional file 1: Table S23) and Hardy-Weinberg principles.

**Table 9:** **Genotype-phenotype drug response interpretations of the Pharmacogenomics Advisory Group-approved drug-gene pairs simvastatin and *SLCO1B1***^**a**^

**Common diplotypes**^**b**^	**Expected population frequency, %**^**c**^			**PK/PD phenotype**	**Clinical phenotype**	**FDA guidelines**
	**Caucasian** **ancestry**	**African an** **cestry**	**East Asian ancestry**			
SLCO1B1*1/*1	64	92	79	Normal drug transport	Normal response and risk of adverse drug reactions to simvastatin	80 mg simvastatin maximum dose
SLCO1B1*1/V174A, SLCO1B1*1/*6, and another rare diplotype, SLCO1B1*1/*3	32	8	20	Intermediate decreased drug transport	Intermediate increase in risk of myopathy from simvastatin	40 mg simvastatin maximum dose
SLCO1B1 V174A/V174A, SLCO1B1*6/V174A, and other rare diplotypes (see Additional file 1: Table S28)	4	<1	1	Decreased drug transport	Increased risk of myopathy from simvastatin	20 mg simvastatin maximum dose

*Abbreviations*: *FDA* Food and Drugs Administration, *PD* pharmacodynamic, *PK* pharmacokinetic.

^a^Supporting evidence may be found in Additional file 2: Section S7.0 to 7.7 and Additional file 1: Tables S26 to S28. These include summaries of the PhAESIS evaluation and referenced publications supporting the drug-gene clinical phenotypes.

^b^Diplotypes with frequencies of less than 0.4% in Caucasians are included above. Other rare diplotypes that fall under the same phenotype category can be found in the genotype-phenotype Punnett table (see Additional file 1: Table S28). Diplotypes above and in the genotype-phenotype Punnett table include both clinically validated genetic results (those that include variants with evidence code 1) and results that include variants with evidence scores 2 to 7 (potentially clinically relevant). The latter require further validation to support their inclusion for clinical reporting.

^c^Population frequencies are estimated based on reported gene variant allele frequencies (see Additional file 1: Table S27) and Hardy-Weinberg principles.

During the PhAESIS evaluation process, gaps in scientific and clinical evidence for specific gene variants and variant combinations are highlighted for further study. Gaps in knowledge for each of the drug-gene pairs presented in this report are summarized in the respective ‘Gaps in PGx knowledge’ subsection of Additional file 2: Sections S1 to S7. Broadly, these include the following:

1) Limited published PGx data for many of the drug-gene pairs evaluated. Although all show clinical validity for at least one genetic result (association with an altered clinical outcome) most lack sufficient data, having only a limited number of studies demonstrating a clear association with drug response or tolerance (for example only evidence linking CYP2C9*3/*3 genotype and celecoxib-*CYP2C9* tolerance; mostly case report-based evidence linking codeine ADRs and *CYP2D6* UM metabolizer status; and limited data on CYP2C19*17 genotypes and PPI efficacy).

2) In particular, there are limited data on rare or ancestry-specific variants with respect to effects on protein function or drug-specific clinical response (typically variants with evidence codes ≥8).

3) A general need to expand basic and clinical research, given that the greatest body of published data comes from Caucasian populations, and therefore has limited application to the general world population (typically for variants with evidence code ≥2).

4) Missing genotype data on other known functional variants in the gene(s) under evaluation, confounding the interpretation of published drug response results (for example, the lack of genotype data on other loss of function variants and CYP2C19*17 in many of the earlier PGx studies of this gene; similarly, lack of qualitative and quantitative data on *CYP2D6* copy number variants).

5) Lack of data on haplotype structure/phase with possible subsequent confounding of drug response interpretation (for example, the effect of *SLCO1B1* variant N130D on simvastatin response (see Additional file 2: Section S7)); another example includes the presence of rare alleles such as the CYP2C19*17 variant in *cis* with either a CYP2C19*2 or CYP2C19*4 allele as observed in the CPMC cohort (data not shown) and reported by others [38]. Given that most studies are population-based rather than family-based, and therefore lack phasing information, the presence of double heterozygotes for these variants may lead to misinterpretation of the true metabolizer type (for example, a CYP2C19*2/CYP2C19*17 intermediate metabolizer for PPI response vs. CYP2C19*1/CYP2C19*2+CYP2C19*17, for which metabolizer type is unknown).

## Discussion

In order to implement PGx reporting in the CPMC, and to facilitate interpretation and dissemination of personalized PGx data to the study participants, the CPMC set out to gather, systematically review, and critically appraise published and public PGx data from a variety of sources. An evidence-based scoring system was developed to parse the clinical relevance of gene variants in the context of specific drugs.

The CPMC PhAESIS method has several key strengths: 1) By taking a drug-centered approach, the full extent of current PGx knowledge is summarized, allowing simultaneous identification of genetic results with sufficient data to support clinical diagnostic applications, and highlighting the questions that remain to be answered. 2) The multi-tier evidence scoring system allows all published and supported key functional variants to be identified, including those in minority ethnic and racial populations. 3) The scoring system provides an invaluable overview of what genetic data are clinically supported and where gaps in knowledge exist. Filling these gaps is crucial to the successful development of diagnostic tools that are able to identify all patients likely to benefit from the targeted personalized drug therapies. 4) Another key feature of the CPMC PhAESIS system is the use of an external expert advisory panel to vet the results of the systematic review. This approach adds further weight to the conclusions and interpretations of the data to be reported. The PAG also provides guidance on which subset of genetic results for a particular drug are of sufficient clinical significance (highly actionable) and therefore warrant a higher level of communication with CPMC study participants (Table 2).

The CPMC is not alone in recognizing the need to develop a system that distills the published research data into clear and evidence-based therapeutic guidelines to ease the implementation of PGx in clinical practice. There are several other groups and organizations working to collate the research literature using a gene specific approach. These include, among others, the PharmGKB, a web-based database of curated and annotated data on PGx gene variants and gene-drug-disease relationships [24]; the Human Cytochrome P450 (CYP) Allele Nomenclature Committee website, which provides updated information on PGx relevant genetic variations of human CYP enzymes [25]; and the Cytochrome P450 Drug Interaction Table at Indiana University School of Medicine [20], which provides lists and publication references for drug-gene interactions. These are invaluable tools for researchers, including the CPMC. In the case of the drug interaction website, whose primary goal has been to provide information that can mitigate ADRs in polypharmacy settings, this resource is utilized by clinicians and researchers alike.

Other, more targeted efforts aimed at providing clinical pharmacogenomics guidelines that can be utilized by diagnostics developers and healthcare providers include those of the Clinical Pharmacogenetics Implementation Consortium (CPIC), established by the National Institutes of Health Pharmacogenomics Research Network and PharmGKB [39], and the Pharmacogenetics Working Group (PWG), established by the Royal Dutch Association for the Advancement of Pharmacy [40,41]. Another initiative, aimed at establishing a systematic, evidence-based process for assessing genetic tests and other applications of genomic technology in transition from research to clinical and public health practice, is the CDC-sponsored Evaluation of Genomic Applications in Practice and Prevention (EGAPP) [42]. The EGAPP initiative evaluates tests such as those for predictive testing for inherited risk of common diseases and pharmacogenetic testing for variation in drug response. All three of these groups aim to provide peer-reviewed guidelines for pharmacogenetics-based therapeutic (dose) recommendations. All have developed a process that involves systematic review of published literature, scoring of evidence for drug phenotype or genotype categories, and interpretation of this evidence to guide therapeutic recommendations. Like the CPMC, CPIC, PWG, and EGAPP also employ expert panels of researchers and clinicians working in the field of study to guide the evaluation process and resulting recommendations. However, the CPMC is unique in that the expert advisory panel is independent of the CPMC study and includes a broader representation of stakeholders including practicing physicians and an ethicist. In addition, CPMC PAG evaluation and approval occurs in the context of a research study, and the threshold for reporting may be lower, compared with the goals of CPIC and PWG, in order to include genetic variants that are potentially clinically relevant. For example, Swen *et al*. (PWG) [40] included CYP2C19*2, CYP2C19*3 and CYP2C19*17 only in their metabolizer type classifications, and the CPIC publications placed a greater emphasis on variants common in Caucasian populations for which there is a greater body of data (such as the *2 and *3 alleles of both *CYP2C19* and *CYP2C9*), although reference to other variants of potential effect are made in the supplementary materials sections of the publications [36,43]. Such variants (typically those with an evidence score of 2 to 7) are highlighted by the PhAESIS evaluation as needing further clinical validation by the CPMC or other researchers to support their clinical utility.

Like the CPMC approach, evidence scoring by CPIC, PWG, and EGAPP is based on level or strength of evidence and clinical relevance [39-41]. However, the CPMC tiered evidence scoring method allows clearer distinction of the clinical relevance of individual PGx genetic variants, and like the EGAPP effort, highlights gaps in knowledge for further study. For example, distinction can be made between a variant with a score of 1 (with published clinical outcomes data for the drug under review) versus a score of 3 (indicating *in vitro* data supporting the effect of the variant on protein function) versus a score of 5 (clinical outcomes data for another drug) versus a score of 13 (where there are no functional or clinical data available and the clinical relevance is therefore unknown). The primary goal of CPIC and PWG is to provide published guidelines for healthcare providers and diagnostics developers for immediate clinical implementation. PWG has the added advantage of integrating their recommendations into the Dutch electronic drug database that is utilized as part of the clinical automated medication surveillance system [41]. By contrast, the primary goal of the CPMC is to deliver the PGx genetic interpretations within a research setting directly to the study participants, to allow both the investigation of participant understanding and behavior in response to receiving PGx risk results, and the carrying out of drug-gene specific discovery and validation research to confirm prior associations and/or address current gaps in knowledge.

The key limitation of the PhAESIS system is shared by all of the other described systems for evaluating and reviewing published and public PGx data, in that all the systems are limited to the data available at the time of the initial evaluation. All, including the CPMC, require efforts to update information on an ongoing basis. In the context of the CPMC research study, a revision schedule has not been systematically implemented. However, the CPMC has brought updates of previously approved drug-gene pairs to the PAG for re-evaluation in cases where subsequently published data could potentially reverse or modify the original decision of the PAG. This is the case for *CYP2C19* and clopidogrel, which was first reviewed and approved by the PAG in March 2010 and re-evaluated in October 2010 following publication of controversial data in August 2010 [44]. However, these new data did not affect the approval to release results for this drug-gene pair, and the results report was subsequently implemented and released to study participants. The CPMC is exploring the use of automation in many parts of the system to facilitate the PhAESIS process. This could include automated publication database searches and prioritization for review and evidence scoring by a scientist reviewer, and variant data-gathering for many of the tables included in the reports.

In addition, like other published approaches, the method is limited by the available published data both in terms of the completeness of the genetic data (inclusion of all relevant variants and information on phasing and gene linkage disequilibrium structure) and the uniformity of study design and treatment regimen used (including dosing and duration variability, and disclosure about other concomitant drug therapies).

Another limitation of the PhAESIS approach is that the evidence scoring was developed specifically for drugs that are affected by germline or inherited genomic variations and not those that are affected by somatic mutations, such as in tumor or cancer genomes. Although specific anticancer drugs (such as thiopurines, tamoxifen, and 5-fluorouracil) have been evaluated, the published data for these were based on genomic variants and not on the analysis of the tumor/cancer genome. In essence, a similar evidence scoring procedure can be developed that includes data from either or both inherited and cancer genomic data. At present, the evidence table is not designed to capture evidence from the tumor genome, but potentially can be adapted in the future to include this.

Finally, a limitation not of the PhAESIS method but of its implementation within the CPMC study is a focus on genes that are represented on the genotyping platforms currently used. The CPMC can interrogate 1.8 million SNPs and insertion/deletions across the genome in the Affymetrix Genomewide Human 6.0 array, and 1,936 variants specific to genes for drug absorption, disposition, metabolism, and elimination in the Affymetrix DMET Plus array. In addition, copy number variation at the CYP2D6 gene can be detected by a recently implemented Luminex® assay. In the future, adoption of whole genome sequencing by the project is likely to address this limitation.

## Conclusions

In conclusion, the system described here for the evaluation and translation of PGx data for specific drug-gene interactions has broad application for guiding the development of PGx testing for diagnostic use, identifying gaps in knowledge for further research, and providing results interpretation guidelines for the education of stakeholders (healthcare professionals and health consumers). In this way, the CPMC is contributing to efforts facilitating the implementation of PGx into personalized medical practice.

## Abbreviations

ADR: Adverse drug reaction; CPIC: Clinical Pharmacogenetics Implementation Consortium; CPMC: Coriell Personalized Medicine Collaborative; EGAPP: Evaluation of Genomic Applications in Practice and Prevention; EMR: Electronic medical record; FDA: Food and Drug Administration; ICOB: Informed Cohort Oversight Board; PAG: Pharmacogenomics Advisory Group; PD: Pharmacodynamic; PGx: Pharmacogenomics; PhAESIS: Pharmacogenomics Appraisal, Evidence Scoring and Interpretation System; PharmGKB: Pharmacogenomics Knowledgebase; PK: Pharmacokinetic; PWG: Pharmacogenetics Working Group; SNPs: Single nucleotide polymorphisms; UM: Ultra-rapid metabolizer.

## Competing interests

The authors declare that they have no competing interests.

## Authors’ contributions

NG, MAK, and CBS conceived and developed the PhAESIS method and participated in the drafting and writing of the manuscript. NG, LH, TJS, and DEL generated drug-gene PhAESIS submission reports for PAG evaluation for the drug-gene pairs included in this paper. LH and DEL helped with the drafting of the manuscript. ESG and MFC provided intellectual input into the PhAESIS method. MFC participated in the coordination of the CPMC study. All authors edited the paper and read and approved the final manuscript.

## Supplementary Material

Additional file 1 Contains all the tables (Tables S1 to S28) referenced in Additional file 2 and in the main manuscript. Note that, given the research setting, gene variant evaluations were prioritized to those present on the genotyping platforms used by the Coriell Personalized Medicine Collaborative (CPMC) study (Affymetrix DMET Plus and Genomewide Human 6.0 arrays). As such the gene variant evidence (see Additional file 1: Tables S3, S6, S9, S11, S13, S15, S20, S23, and S27) includes variants on these platforms and any other key variants identified during literature and database searches. Other reported variants are not systematically included. Click here for file

Additional file 2 **Consists of extracts from Pharmacogenomics Appraisal, Evidence Scoring and Interpretation System (PhAESIS) submission documents for the seven drugs and nine genes approved for risk reporting by the Coriell Personalized Medicine Collaborative (CPMC) Pharmacogenomics Advisory Group (PAG).** Sections S1 to S7 represent summary annotations of PhAESIS reports submitted to and subsequently approved by the CPMC PAG. The concise summaries include a description and mechanism of action of the drug under review, an overview of the PGx data for the drug, summary of the drug-gene evidence for the key PGx genes, strength of evidence scoring of genetic variants, genotype-phenotype interpretations, current FDA and other clinical association guidelines, and gaps in PGx knowledge for the drug-gene pair. The data are taken directly from PAG reports with the date of PhAESIS review by the PAG provided. In some cases, more recent data are also cited in the text. Click here for file
