# Supplementary material for: The Coriell personalized medicine collaborative pharmacogenomics appraisal, evidence scoring and interpretation system
Source: Genome Med. 2013 Oct 18;5(10):93. doi: 10.1186/gm499 (PMC3978656; doi:10.1186/gm499)
Supplement: Additional file 1 — Contains all the tables (Tables S1 to S28) referenced in Additional file 2 and in the main manuscript. Note that, given the research setting, gene variant evaluations were prioritized to those present on the genotyping platforms used by the Coriell Personalized Medicine Collaborative (CPMC) study (Affymetrix DMET Plus and Genomewide Human 6.0 arrays). As such the gene variant evidence (see Additional file 1: Tables S3, S6, S9, S11, S13, S15, S20, S23, and S27) includes variants on these platforms and any other key variants identified during literature and database searches. Other reported variants are not systematically included. [file gm499-S1.docx]

**­Table S1.** Resources for Published and Public Pharmacogenomics Literature and Databases

| **Public Resource** | **Description­­** | **Reference** |
| --- | --- | --- |
| Coriell Personalized Medicine Collaboration | Prospective observational study designed to evaluate the utility of personalized genomic information in health management | www.cpmc.coriell.org |
| CYP450 allele nomenclature web site | The Human Cytochrome P450 (CYP) Allele Nomenclature Database­­ | http://www.cypalleles.ki.se/ |
| CYP450 Drug Interaction Table | List of drugs paired with specific cytochrome P450 isoforms that are responsible, at least in part, for the metabolism of the drug | http://medicine.iupui.edu/clinpharm/ddis/table.aspx |
| dbSNP | An extensive source of information on short genetic variations | http://www.ncbi.nlm.nih.gov/projects/SNP/ |
| Drugs@FDA | A link to FDA Approved Drug Label | http://www.accessdata.fda.gov/scripts/cder/drugsatfda/ |
| Drug Topics | National drug prescription statistics for brand and generic drugs | http://drugtopics.modernmedicine.com/ |
| Pharmacy Times Top 200 Drugs | A source for the annual statistics on the total prescriptions and sales for the top 200 drugs in the United States | http://www.pharmacytimes.com/publications/issue/2012/ July2012/Top-200-Drugs-of-2011 |
| FDA Table of PGx Biomarkers in Drug labels | List of FDA-approved drugs with pharmacogenomic information in their labels. | http://www.fda.gov/Drugs/ScienceResearch/ResearchAreas/ Pharmacogenetics/ucm083378.htm |
| HapMap Browser | A source for population based variant allele frequency information | http://hapmap.ncbi.nlm.nih.gov/ |
| PharmGKB | A source of curated and annotated data on PGx gene variants and gene-drug-disease relationships | https://www.pharmgkb.org/ |
| PubMed | A source for published peer-reviewed scientific and clinical articles | http://www.ncbi.nlm.nih.gov/pubmed/ |
| 1000 Genomes Project browser | A source for population-based variant allele frequency information | http://browser.1000genomes.org/index.html |
| National Institutes of Health PGx Research Network | A source of PGx research tools, samples, and data for guiding personalized medicine treatment | http://www.pgrn.org |

**Table S2. Studies of CYP2C19 variation and clinical outcomes in clopidogrel-treated patients**

| **Reference** | **Study Design** | **Patient Population** | **# Patients** | **Primary / Secondary Endpoints** | **# Events Observed** | **Primary / Secondary Results** |
| --- | --- | --- | --- | --- | --- | --- |
| [[1](#_ENREF_1)] | substudy of TRITON-TIMI | ACS | N=1477 | CVD death, MI, or stroke (primary)  stent thrombosis (secondary) | 129 (primary)  20 (secondary) | 12.1% (PM+IM) vs. 8.0% (EM); HR=1.53 (1.07 to 2.19) p=0.01  2.6% (PM+IM) vs. 0.8% (EM); HR=3.09 (1.19 to 8.0) p=0.02 |
| [[2](#_ENREF_2)] | observational | acute MI | N=2208 | CVD death, MI, or stroke | 294 | Pooling IM with EM:  21.5% (PM) vs. 13.3% (IM+EM); HR=1.98 (1.10 to 3.58)  Pooling IM with PM: HR=0.69 (0.51 to 0.93) |
| [[3](#_ENREF_3)] | observational | young MI survivors (<45 years old) | N=259 | death, MI, or urgent revascularization (primary)  stent thrombosis (secondary) | 26 (primary)  12 (secondary) | Event rate per 100 p-years:  10.9 (*2 carriers) vs. 2.89 (EM); HR=3.69 (1.69 to 8.05) p<0.001  Event rate per 1000 p-years:  6.79 (*2 carriers) vs. 1.14 (EM); HR=6.02 (1.81 to 20.04) p<0.001 |
| [[4](#_ENREF_4)] | observational | PCI | N=2485 | definite stent thrombosis | 17 | 1.5% (*2 carriers) vs. 0.4% (EM); HR=3.8 (1.5 to 10.0) p=0.006 |
| [[5](#_ENREF_5)] | observational | PCI | N=772 | definite or probable stent thrombosis | 24 | 5.3% (*2 carriers) vs. 2.1% (EM); OR=3.4 (1.0 to 12.8) p=0.047 |
| [[6](#_ENREF_6)] | observational | PCI | N=797 | death or MI (after stent placement) | 24 | 2.0% (*2 carriers) vs. 3.4% (EM) p=0.37 |
| [[7](#_ENREF_7)] | observational | PCI | N=1524 | 30 day incidence of bleeding  30 day incidence of stent thrombosis | 17 major and 45 minor bleeding  14 ischemic events | Multivariate analysis showed independent association of **17* allele with platelet aggregation (*P*<0.001) and bleeding event (*P*=0.006). No significant influence of **17* on the stent thrombosis was found (*P*=0.79). |
| [[8](#_ENREF_8)] | Randomized placebo-controlled trials CURE and ACTIVE A | ACS  AF | N=5059 (2549 clopidogrel; 2510 placebo)  N=1156 | CVD death, nonfatal MI, or stroke  Main safety outcome: major bleeding | CURE – 231 clopidogrel/316 placebo | Rate of primary efficacy outcome between the clopidogrel and placebo groups: PM/IM clopidogrel (8%) vs. placebo (11.6%) [HR with clopidogrel: 0.69; 95% CI 0.49-0.98]; rate among non-carriers was 9.5% (clopidogrel) and 13% placebo [HR with clopidogrel: 0.72; 95% CI 0.59-0.87].  Conversely *17 carriers significantly benefited from clopidogrel vs. placebo (7.7%) primary outcome on clopidogrel vs. 13% on placebo [HR with clopidogrel: 0.55; 95% CI 0.42-0.73]; rate among non-carriers was 10% (clopidogrel) and 12.2% placebo [HR with clopidogrel: 0.85; 95% CI 0.68-1.05].  Effect of clopidogrel on bleeding did not vary by genotype. |
| [[9](#_ENREF_9)] | observational | Acute MI | N=928 | Need for target vessel revascularization (TLR) (primary)  major adverse cardiovascular events (MACE); including death, myocardial infarction [MI], and TLR (secondary) | 177 TLR  239 MACE | CYP2C19*2 carriers not at increased risk of TLR or MACE compared to those with CYP2C19*1/*1 genotype (P=0.842 and P=0.529 respectively)  CYP2C19*17 carriers had a 37% relative reduction in the TLR incidence (14.0% vs 22.3%, P = .002), and a 22% relative reduction of the secondary end point MACE (22.0% vs 28.1%, P = .04) compared with noncarriers, respectively. |
| [[10](#_ENREF_10)] | Genetic substudy of PLATO trial | ACS | N=5148 genotypes for *2-*8 &*17 | CVD death, MI, or stroke(primary)  Major Bleeding events (secondary) | 481  483 | Carriers of loss-of-function allele (5.7%) vs. non-carriers 3.8%; HR=1.53 (1.07 to 2.19) p=0.028 within 30 days of treatment  Carriers of *17 had a higher risk of major bleeding (11.9%) vs. 9.5% of those with *1/*1 genotype (P=0.022) |

ACS = acute coronary syndrome; MI = myocardial infarction; PM = poor metabolizer, IM = intermediate metabolizer, EM = extensive metabolizer, CVD = cardiovascular disease, HR = hazard ratio, PCI = percutaneous coronary intervention, OR = odds ratio, AF = atrial fibrillation

**Table S3. Metabolic phenotypes, frequency and evidence scoring of gene allelic variants for *CYP2C19*-clopidogrel**

| **Allele** | **Key Variant dbSNP ID** | **Nucleotide Change (cDNA)** | **Mutation Effect** | **Enzyme Activity** | | **Evidence code^a^** | **Allele Frequency (# Subjects (%))** | | | | **Key References**  **(PMID#)** |
| --- | --- | --- | --- | --- | --- | --- | --- | --- | --- | --- | --- |
|  |  |  |  | **In Vitro/ In Vivo** | **Metabolic Phenotype** |  | **Caucasian** | **African** | **Asian** | **Reference^e^** |  |
| CYP2C19*1 | wild type | reference | lacks variants below | normal | normal | 1 | (61%) ^d^ | (60%) ^d^ | (60%) ^d^ | Estimated**^d^** | reference in studies below |
| CYP2C19*2 | rs4244285^b^ | 681G>A | P227P, splice defect [[11](#_ENREF_11), [12](#_ENREF_12)], frameshift, truncated and null protein [[11](#_ENREF_11)], increased clopidogrel ADRs | none | none | 1 | 379  (15%) | 246 (17%) | 288 (33%) | 1000GP | [[1](#_ENREF_1), [6](#_ENREF_6), [11-14](#_ENREF_11)] |
| CYP2C19*3 | rs4986893^c^ | 636G>A | W212X, truncated and null protein, in Asian mephenytoin PM [[15](#_ENREF_15)], increased clopidogrel ADRs | none | none | 1 | 379  (<1%) | 246 (0.4%) | 288 (5%) | 1000GP | [[1](#_ENREF_1), [2](#_ENREF_2), [15](#_ENREF_15), [16](#_ENREF_16)] |
| CYP2C19*4 | rs28399504 | 1A>G | M1V, initiation error, no expression in vitro, in mephenytoin PM [[17](#_ENREF_17)], increased clopidogrel ADRs | none | none | 1 | 379 (0%) | 246 (0%) | 288 (0.2%) | 1000GP | [[1](#_ENREF_1), [2](#_ENREF_2), [17](#_ENREF_17), [18](#_ENREF_18)] |
| CYP2C19*5 | rs56337013 | 1297C>T | R433W in heme-binding region, no holoprotein; no activity in vitro for mephenytoin, omeprazole [[19](#_ENREF_19)], tolubutamide [[20](#_ENREF_20)], in 3% Caucasian and 4% Bai Asian mephenytoin PMs [[20](#_ENREF_20), [21](#_ENREF_21)] | none | none | 1 | (<1%) | (0%) | 303 (<1%) | [[20](#_ENREF_20), [21](#_ENREF_21)] | [[1](#_ENREF_1), [2](#_ENREF_2), [19-21](#_ENREF_19)] |
| CYP2C19*8 | rs41291556 | 358T>C | W120R, no mephenytoin activity in vitro [[19](#_ENREF_19)], low/no tolubutamide, mephenytoin, and omeprazole activity in vitro [[19](#_ENREF_19), [22](#_ENREF_22)], in mephenytoin PM, increased clopidogrel ADRs | none/ reduced | none | 1 | 379 (0.3%) | 246 (0.4%) | 288 (0%) | 1000GP | *[*[*1*](#_ENREF_1)*,* [*14*](#_ENREF_14)*,* [*19*](#_ENREF_19)*,* [*22*](#_ENREF_22)*]* |
| CYP2C19*17 | rs12248560 | -806C>T | 5’, high transcription, gain-of-function, possible increased risk of bleeding on clopidogrel | increased | increased | 1 | 379  (23%) | 246 (22%) | 288 (2%) | 1000GP | [[7](#_ENREF_7), [10](#_ENREF_10), [23](#_ENREF_23)] |
| CYP2C19*6 | rs72558184 | 395G>A | R132Q, in 3% Caucasian mephenytoin PMs, in vitro activity low for tolubutamide and mephenytoin, and absent for omeprazole [[19](#_ENREF_19), [24](#_ENREF_24)] | none | none | 6scd | 172 (<1%) | 44 (0%) | 88 (0%) | *[*[*19*](#_ENREF_19)*,* [*24*](#_ENREF_24)*]* | [[19](#_ENREF_19), [22](#_ENREF_22)] |
| CYP2C19*7 | rs72558186 | intronic IVS5+2T>A | 5’ donor splice defect [[12](#_ENREF_12), [22](#_ENREF_22)], in 3% Caucasian mephenytoin PMs [[22](#_ENREF_22)] | none | none | 6se | 173 (<1%) | 44 (0%) | 86 (0%) | [[17](#_ENREF_17), [22](#_ENREF_22)] | [[12](#_ENREF_12), [22](#_ENREF_22)] |
| CYP2C19*10 | rs6413438 | 680C>T | P227L, low mephenytoin activity, CLint, and Vmax in vitro [[19](#_ENREF_19), [25](#_ENREF_25)], low omeprazole activity and high km in vitro [[19](#_ENREF_19)] | reduced | ND | 8 | 379  (0%) | 246 (0%) | 288  (0.9%) | 1000GP | [[19](#_ENREF_19), [25](#_ENREF_25)] |
| CYP2C19*9 | rs17884712 | 431G>A | R144H, low in vitro mephenytoin activity [[19](#_ENREF_19), [25](#_ENREF_25)] | slightly reduced | ND | 11 | 379  (0%) | 246 (1.2%) | 288 (0%) | 1000GP | [[19](#_ENREF_19), [25](#_ENREF_25)] |
| CYP2C19*12 | rs55640102 | 1473A>C | X491C, unstable protein in vitro [[25](#_ENREF_25)] | unstable protein | ND | 11 | 44 (0%) | 23 (4%) | 89 (0%) | ALFRED [[25](#_ENREF_25)] | [[25](#_ENREF_25)] |
| CYP2C19*SD | rs4244285b | 681G>C | P227P | ND | ND | 13 | ND | ND | ND | dbSNP | ND |
| CYP2C19 G439X | rs5787121 | 1314G>del | G439X, frameshift | ND | ND | 13 | 24 multiethnic (2%) | | | dbSNP | ND |
| CYP2C19*13 | rs17879685 | 1228C>T | R410C [[25](#_ENREF_25)], mephenytoin activity low at 25mcM, normal at 200mcM in vitro; normal omeprazole activity in vitro [[19](#_ENREF_19)] | normal/ reduced | ND | 14 | 156 (0%) | 49 (1%) | 88 (0%) | dbSNP  [[25](#_ENREF_25)] | [[19](#_ENREF_19), [25](#_ENREF_25)] |
| CYP2C19*14 | rs55752064 | 50T>C | L17P [[25](#_ENREF_25)], normal in vitro omeprazole activity [[19](#_ENREF_19)] | normal | ND | 14 | 45 (0%) | (2%) | 200 (0%) | [[25](#_ENREF_25)]  ALFRED | [[19](#_ENREF_19), [25](#_ENREF_25)] |
| CYP2C19*15 | rs17882687 | 55A>C | I19L , high in vitro mephenytoin Vmax; normal omeprazole activity in vitro [[19](#_ENREF_19)] | normal/ increased | ND | 14 | 43 (1%) | 16 (5%) | 96 (3%) | [[25](#_ENREF_25)] ALFRED | [[19](#_ENREF_19), [25](#_ENREF_25)] |

ND = no data/not determined, PM = poor metabolizer, UM = ultra-rapid metabolizer, ADR = adverse drug reaction, Vmax = maximum metabolic velocity, CL = clearance, CLint = intrinsic clearance, Km = concentration at half-Vmax. ^a^ **=** evidence code assigned to each allele indicates the strength and type of evidence for a drug-related phenotype based on the scheme presented in **Table 1** (green indicates clinically relevant evidence, yellow=potential clinical relevance, light orange= clinical relevance unknown, and dark orange= clinical relevance unsupported). ^b^ = rs4244285 is tri-allelic (681G>A/C), where A defines CYP2C19*2 and C defines CYP2C19*SD; ^c^ = 636G>A/T (W212X/C) is tri-allelic, where A defines CYP2C19*3 (rs4986893) and T is unreported by dbSNP, **^d^** = CYP2C19*1 allele frequency is estimated and takes into account the allele frequencies of other variants reported in this table with an evidence code ≤ 7. ^e^ = allele frequency from 1000 Genomes Project phase 1, HapMap, dbSNP, ALFRED, or specific reference.

**Table S4. *CYP2C19*-Clopidogrel predicted genotype-phenotype interpretations (extended punnett square)**

| **CYP2C19 allele**  **(freq. %)** | **CYP2C19*1** | **CYP2C19*2** | **CYP2C19*3** | **CYP2C19*4** | **CYP2C19*5** | **CYP2C19*6** | **CYP2C19*7** | **CYP2C19*8** | **CYP2C19*17** |
| --- | --- | --- | --- | --- | --- | --- | --- | --- | --- |
| **CYP2C19*1**  **(61.3)** | *1/*1 (38%) EM | *1/*2 (18%) IM | *1/*3 (<0.1%) IM | *1/*4 (<0.1%) IM | *1/*5 (<0.1%) IM | *1/*6 (<0.1%) IM | *1/*7 (<0.1%) IM | *1/*8 (0.4%)  IM | *1/*17 (29%) UM |
| **CYP2C19*2**  **(15)** |  | *2/*2 (2.3%) PM | *2/*3 (<0.1%) PM | *2/*4 (<0.1%) PM | *2/*5 (<0.1%) PM | *2/*6 (<0.1%) PM | *2/*7 (<0.1%) PM | *2/*8 (0.1%) PM | *2/*17 (7%) unk |
| **CYP2C19*3**  **(<0.1)** |  |  | *3/*3 (<0.1%) PM | *3/*4 (<0.1%)  PM | *3/*5 (<0.1%)  PM | *3/*6 (<0.1%)  PM | *3/*7 (<0.1%)  PM | *3/*8 (<0.1%)  PM | *3/*17 (<0.1%) unk |
| **CYP2C19*4**  **(<0.1)** |  |  |  | *4/*4 (<0.1%)  PM | *4/*5 (<0.1%)  PM | *4/*6 (<0.1%) PM | *4/*7 (<0.1%)  PM | *4/*8 (<0.1%)  PM | *4/*17 (<0.1%)  unk |
| **CYP2C19*5**  **(<0.1)** |  |  |  |  | *5/*5 (<0.1%)  PM | *5/*6 (<0.1%)  PM | *5/*7 (<0.1%)  PM | *5/*8 (<0.1%)  PM | *5/*17 (<0.1%)  unk |
| **CYP2C19*6**  **(<0.1)** |  |  |  |  |  | *6/*6 (<0.1%)  PM | *6/*7 (<0.1%)  PM | *6/*8 (<0.1%)  PM | *6/*17 (<0.1%)  unk |
| **CYP2C19*7**  **(<0.1)** |  |  |  |  |  |  | *7/*7 (<0.1%)  PM | *7/*8 (<0.1%)  PM | *7/*17 (<0.1%)  unk |
| **CYP2C19*8**  **(0.3)** |  |  |  |  |  |  |  | *8/*8 (<0.1%)  PM | *8/*17 (0.1%)  unk |
| **CYP2C19*17**  **(23.0)** |  |  |  |  |  |  |  |  | *17/*17 (5%) UM |

Approximate expected genotype frequencies have been estimated based upon reported allele frequencies for Caucasians (Table S3) and Hardy Weinberg principals. Each potential CYP2C19 genotype has been assigned a predicted drug metabolizing phenotype based on predicted metabolic activity of individual alleles (normal, enhanced or reduced enzymatic function) such that extensive metabolizer (EM) is defined as 2 alleles with normal activity; ultra-rapid metabolizers (UM) with 2 enhanced activity alleles or 1 normal and 1 enhanced allele; Intermediate metabolizers (IM) have 1 normal and 1 poor metabolizing allele; and Poor metabolizers (PM) have 2 reduced activity alleles. The drug metabolizing phenotype for the presence of 1 enhanced activity and 1 reduced activity alleles is currently unknown and denoted by “unk”. Color shading is used to define expected drug response based on published outcomes data such that: Blue indicates likely normal response to clopidogrel; Yellow/Orange indicate those at increased risk of ischemic events when taking clopidogrel (with yellow at a higher risk) in patients undergoing percutaneous coronary intervention (PCI); and Purple represents those potentially at an increased risk of bleeding, but whom are likely to derive greater protection from ischemic event while on clopidogrel. Pink indicate unknown clinical phenotypic assignments.

**Table S5. Studies of CYP2C19 variation and clinical outcomes in PPI-treated patients**

| **Reference** | **Subjects, Drug** | **Comparison Groups** | **Key Endpoint Results** | **Conclusions and Comments** |
| --- | --- | --- | --- | --- |
| [[26](#_ENREF_26)] | Healthy (n=19), Caucasian, esomeprazole or pantoprazole | **CYP2C19** n=2 UM: *1/*17  n=7 EM: *1/*1 n=7 IM: *1/*2  n=2 IM: *2/*17  n=1 PM: *2/*2 | **For pantoprazole,**  **Median 24h inhibition of intragastric pH:**  EM vs PM: day 1, p=0.030; day 5, p=0.043  **% time inhibition intragastric pH:**  EM vs PM: day 1, p=0.025; day 5, p=0.004  **Pantoprazole AUC:** Day 1 EM: 3.82 vs PM: 7.95, p=0.023  Day 5 EM: 4.12 vs PM: 8.90, p=0.010 | For EMs vs PMs, significant differences for 40mg pantoprazole were found on day 1 and 5 for: acid inhibition and AUC. Esomeprazole was more effective than pantoprazole, and had no significant genotypic associations. No one carried CYP2C19*3 or *6 alleles. Intragastric pH was among the lowest measured for both PPIs for two *1/*17 carriers; while *2/*17 carriers had values above and below the group median pH, respectively, in most cases. |
| [[27](#_ENREF_27)] | GERD (n=65), unknown ethnicity, lansoprazole | **CYP2C19** n=24 EM: *1/*1  n=28 IM: *1/*2, *1/*3  n=13 PM: *2/*2, *2/*3, *3/*3 | **% GERD cure rate (95% CI) EM:IM:PM:** 45.8 (25.5-67.3): 67.9 (47.6-84.2): 84.6 (54.5-98.1)  **Relative GERD cure rate (CI):**  EM: 1.00 vs IM: 5.497 (1.2-24.3) p=0.0246  EM: 1.00 vs PM: 15.897 (2.2-117.2) p=0.0038 | Log regression shows genotype and pre-treatment grade of GERD is significantly associated with the success or failure of curing GERD. Plasma PPI levels were associated with genotype groups (EM, IM, PM). Success/failure was associated with lansoprazole plasma levels. |
| [[28](#_ENREF_28)] | Healthy (n=15), Japanese, omeprazole or rabeprazole | **CYP2C19** n=6 EM: *1/*1  n=5 IM: *1/*2, *1/*3  n=4 PM: *2/*2, *2/*3 | **Median intragastric pH (single dose):** p<0.01 (EM vs IM, EM vs PM, IM vs PM) for omeprazole; p=0.01 (IM vs PM) for rabeprazole  **Median intragastric pH (repeated dose):** p<0.02 (EM vs IM, EM vs PM) for omeprazole | Significant efficacy differences were found for omeprazole and rabeprazole according to genotype: intragastric pH. Omeprazole also showed significant pharmacokinetic differences by genotype: AUC prodrug, AUC metabolite. |
| [[29](#_ENREF_29)] | Acid disorders ± *H. pylori* (n=25: 17 non-active ulcer, 7 GERD, 1 EE), Caucasian, omeprazole | **CYP2C19** n=11 EM: *1/*1  n=12 IM: *1/*2, *1/*3  n=2 PM: *2/*2 | **Median intragastric pH:** p<0.0001,  EM vs PM: 3.1 (2.7-3.6) vs 5.5 (5.1-5.9)  **% time intragastric pH>4:** p<0.0001,  IM vs EM: 72.4% vs. 37.1%  **Gastrin:** p=0.002, EM vs IM: 16% vs. 157% (±***H. pylori*:** p=0.02, with/without: 226% vs 80%) | Significant differences between EMs and PMs were found: median 24h intragastric pH, duration pH>4, and meal-stimulated plasma gastrin increase. Serum gastrin concentration differences were more pronounced in *H. pylori*-positives than negatives, stratified by genotypic groups (EM, IM, PM). |
| [[30](#_ENREF_30)] | healthy (n=18), unknown ethnicity, rabeprazole | **CYP2C19** n=5 EM: *1/*1  n=6 IM: *1/*2, *1/*3  n=4 PM: *2/*2 | **Mean intragastric pH:** p=0.0278, EM:IM:PM ( 3.34±1.04, 3.97±0.85, 4.88±0.91) **Serum gastrin conc:** p=0.0359, EM:IM:PM (1410.0±290.0, 2054±735.8, 2182±331.7)  **% inhibition rate at pH3:** p=0.0491, EM:IM:PM (37.9±38.2, 59.4±20.7, 76.1±19.6)  **Relative EM:IM:PM Cmax:** p=0.0052 (1.0:1.7:3.6), **AUC:** p=0.0005 (1.0:2.0:4.3),  **Cl/F:** p=0.0035 (18.7±8.8, 9.9±5.9, 3.6±1.1) | Significant differences were found among genotypic groups (EM, IM, PM) given both single oral 10mg or 20mg rabeprazole dose for: mean 24-hour intragastric pH, percent intragastric inhibition at pH3, serum gastrin (pg-h/mL), and rabeprazole Cmax, AUC and apparent clearance (mL-kg/min). |
| [[31](#_ENREF_31)] | healthy (n=9), Japanese, omeprazole | **CYP2C19** n=5 EM: *1/*1  n=4 IM: *1/*2, *1/*3  n=6 PM: *2/*2, *2/*3 | **Mean intragastric pH:** p=0.0001  EM:IM:PM (2.14±0.10, 3.10±0.11, 4.47±0.36)  **Gastrin conc:** p=0.0001, EM:IM:PM (421.2±116.9, 1402.6±163.5, 5108.8±319.4)  **Omeprazole:gastrin AUC**: r=0.770, p=0.0008  **Omeprazole AUC:intragastric pH**:  r=0.873, p<0.0001 | Metabolic ratio significantly differed across genotypic groups (EM, IM, PM), as did serum gastrin. Omeprazole AUC correlated with gastrin AUC and intragastric pH. |
| [[32](#_ENREF_32)] | healthy (n=17),  Caucasian,  omeprazole | **CYP2C19** n=12 EM: *1/*1  n=5 UM: *17/*17 | **Omeprazole AUC:**  UM vs EM: (1973 vs 4151), p=0.04  **Omeprazole-sulphone AUC:**  UM vs EM: (1083 vs 3343), p=0.03  **Parent:metabolite AUC**:  r2 = 0.95, p< 0.0001 | Following single dose omeprazole in UMs vs EMs, in UMs, omeprazole AUC was 2.1-fold lower, sulphone metabolite was 3.1-fold lower, with good correlation for omeprazole and metabolite AUC. Royal Dutch Association for the Advancement of Pharmacy recommends PPI dose increases based upon the data for five *17/*17 carriers ^(31=PM:21412232)^. |
| [[33](#_ENREF_33)] | healthy (n=20), Han Chinese, rabeprazole | **CYP2C19** n=7 EM: *1/*1  n=6 IM: *1/*2, *1/*3  n=7 PM: *2/*2 | **Relative EM:IM:PM mean intragastric pH:** NS 1.0:1.3:1.8 (1 dose), 1.0:1.2: 1.7 (>1 dose)  **Relative EM:IM:PM plasma gastrin conc:** NS  E1.0:1.2:1.5 (1 dose), 1.0:1.5:1.6 (>1 dose)  **Rabeprazole AUC**: p<0.05 EM: 1150±327 vs PM: 2015±589 (1 dose) EM: 1445±205 vs PM: 2495±739 (>1 dose) | No significant differences were found among the genotypic groups (EM, IM, PM) after a single (day 1) or repeated (day 8) dose for: intragastric pH or mean gastrin AUC; although PPI AUC significantly differed in EMs vs PMs given single and repeated 20 mg doses. Thus, the rabeprazole pharmacokinetics but not efficacy is significantly influenced by CYP2C19. |
| [[34](#_ENREF_34)] | *H. pylori* + ulcer (n=249), Japanese,  amoxicillin + clarithromycin + (omeprazole or lansoprazole or rabeprazole) | **CYP2C19** n=81 EM: *1/*1  n=125 IM: *1/*2, *1/*3  n=43 PM: *2/*2, *2/*3, *3/*3  **IL1B -511 (acid secretion):**  n=68 normal: CC  n=172 abnormal: CT+TT | ***H. pylori* cure rate:** p<0.05  normal secretion + PM: 93.3%  normal secretion + IM: 63.6%  normal secretion + EM: 60.0%  **independent factors for Tx failure:**  clarithromycin: p<0.0001  CYP2C19 genotype: p=0.03 | The IL1B -511 variant influences CYP2C19 genotype effect on cure rate of 1wk PPI polytherapy for *H. pylori*. The overall cure rate in the normal acid-secretion group (i.e. IL1B -511 CC) was 74.3%; and significantly higher among PMs than IMs and EMs. In the low acid-secretion group (i.e. IL1B -511 CT+TT), there was no difference in cure rate per CYP2C19 genotype. |
| [[35](#_ENREF_35)] | *H.pylori* + ulcer (n=139), Caucasian, pantoprazole + amoxicillin + metronidazole | **CYP2C19**  5 groups  n=6: *1/*1  n=7: *1/*2 n=6: *1/*17  n=3: *2/*17  n=3: *17/*17  **CYP2C19** 6 groups  n=47: *1/*1  n=24: *1/*2 n=47: *1/*17  n=2: *2/*2  n=17: *2/*17  n=12: *17/*17 | **pantoprazole AUC:** p= 0.030 in 5 groups for pharmacokinetic assessment  ***H. pylori* cure** **success/failure:** NS in 3 groups  n=2 *2/*2  n=31 *1/*2, *2/*17  n=96 *1/*1, *17/*17 , *1/*17  ***H. pylori* cure success/failure:** NS in 2 groups (from 3 above groups) comparing *2 (n=53) vs non-*2 (n=96), OR=2.32, univariate p=0.10  ***H. pylori* cure success/failure:** NS in 6 groups  comparing genotype frequencies among successes (n=103) and failures (n=36) | Pantoprazole levels differed significantly in 5 genotype groups. Success/failure of PPI treatment for *H. pylori* is not significantly associated with variants of CYP2C19, ABCB1 (3435C>T), or IL1B (+3954C>T). |
| [[36](#_ENREF_36)] | *H.pylori* + ulcer (n=125), Caucasian, pantoprazole + amoxicillin + metronidazole | **CYP2C19**   n=44: *1/*1  n=20: *1/*2 n=1: *2/*2 n=45: *1/*17 n=7: *2/*17 n=8: *17/*17 | **Treatment success vs failure relative to *1/*1** *1/*1: 23 vs. 21 (reference) *2/*17: 7 vs. 0 (p=0.033) All *2 genotypes: 23 vs. 5 (OR=4.2, p=0.015) **Treatment success vs failure by allele frequency** *2: 15.4% (n=23) vs. 5.3% (n=21), p=0.015 *1: p=0.060, NS *17: p=0.663, NS | *H. pylori* treatment success (n=78) versus failure (n=47) were compared for genotype and allele frequency. Compared to *1/*1, treatment success is greater in carriers of the *2 allele. No significant difference in treatment success versus failure was seen for *1 or *17 carriers by allelic association alone. Royal Dutch Association for the Advancement of Pharmacy recommends PPI dose increases for *17/*17 carriers based upon this data ^(31=PM:21412232)^; however efficacy was not associated to *17 in this study. |
| [[37](#_ENREF_37)] | Brazilian Caucasian volunteers (n=9), omeprazole | **CYP2C19**   n=1: *2/*17  n=3: *1/*1  n=3: *1/*17  n=2: *17/*17 | **MR, -log MR, metabolizer status**  *2 carriers (n=1): <3.55, <0.55, EM  non-*2 carriers (n=8): <3.5, <0.5, EM **MR, -log MR, metabolizer status** *17/*2: 3.55, 0.55, EM  *1/*1: 0.93, −0.03, EM  *1/*1: 1.23, 0.09, EM  *1/*1: 3.02, 0.48, EM  *1/*17: 0.28, −0.55, EM  *1/*17: 1.35, 0.13, EM  *1/*17: 1.91, 0.28, EM  *17/*17: 0.20, −0.69, EM  *17/*17: 1.78, 0.25, EM  **Omeprazole MR :midazolam clearance**  r= -0.7544, p=0.04 | Authors suggest all *17 carriers (*1/*17, *2/*17, *17/*17) showed CYP2C19 EM status, similar to *1/*1 carriers. The low-activity *2 allele in one *2/*17 carriers showed similar omeprazole metabolic ratio (MR) as *1 and *17 carrying genotypes. Omeprazole MR correlates well with midazolam metabolic ratio, suggesting omeprazole is a good marker for CYP3A activity. Royal Dutch Association for the Advancement of Pharmacy recommends PPI dose increases based upon the data for these *17/*17 carriers ^(31=PM:21412232)^; however this data shows *17 homozygotes have omeprazole MR values within the range of *1 homozygotes, and within the range of *1/*17 carriers. |
| [[23](#_ENREF_23)] | Swedish (n=97), omeprazole | **CYP2C19**   n=71: *1/*1  n=32: *1/*17  n=4: *17/*17 | **Median omeprazole metabolic ratio** *1/*1 (0.50) > *17/*17 (0.25), p=0.010  *1/*1 (0.50) > *1/*17, p=0.028 | In vivo omeprazole metabolic ratio significantly differed by CYP2C19 genotype (*1/*1 > *1/*17 > *17/*17). Standard dosing in UMs is predicted to give 35-40% lower omeprazole AUC than in EMs. Royal Dutch Association for the Advancement of Pharmacy recommends PPI dose increases based upon the data for these four *17/*17 carriers ^(31=PM:21412232)^. |

NS = not significant, PPI = proton pump inhibitor, GERD = gastroesophageal reflux disease, EE = erosive esophagus, EM = extensive metabolizer (or homozygous EM), IM = intermediate metabolizer (or heterozygous EM), PM = poor metabolizer, UM = ultra-rapid metabolizer, AUC = area under the concentration-time curve, CL/F = apparent drug clearance, conc = concentration, CI = confidence interval, OR = odds ratio.

**Table S6.** **Metabolic phenotypes, frequency, and evidence scoring of gene allelic variants for *CYP2C19*-PPIs**

| **Allele** | **Key Variant dbSNP ID** | **Nucleotide Change (cDNA)** | **Mutation Effect** | **Enzyme Activity** | | **Evidence Code**^a^ | **Allele Frequency (# Subjects (%))** | | | | | | **Key References (PMID#)** |
| --- | --- | --- | --- | --- | --- | --- | --- | --- | --- | --- | --- | --- | --- |
|  |  |  |  | **In Vitro/ In Vivo** | **Metabolic Phenotype** |  | **Caucasian** | **African** | | | **Asian** | **Reference^e^** |  |
| CYP2C19*1 | wild type | reference | lacks variants below | normal | normal | 1 | (61%)**^d^** | (59%)**^d^** | | | (59%)**^d^** | Estimated**^d^** | reference in all studies below |
| CYP2C19*2 | rs4244285**^b^** | 681G>A | P227P, splice defect [[11](#_ENREF_11), [12](#_ENREF_12)], frameshift, truncated and null protein [[11](#_ENREF_11)], in LD with CYP2C9*13 in Asians [[38](#_ENREF_38)], increased efficacy in some studies | none | none | 1 | 379  (15%) | 246  (17%) | | | 288 (33%) | 1000GP | [[26-31](#_ENREF_26), [35](#_ENREF_35), [36](#_ENREF_36), [39](#_ENREF_39)] |
| CYP2C19*3 | rs4986893**^c^** | 636G>A | W212X, truncated, null protein, increased efficacy in most studies | none | none | 1 | 379  (<1%) | 246  (0.4%) | | | 288 (5%) | 1000GP | [[27-31](#_ENREF_27), [33](#_ENREF_33), [34](#_ENREF_34)] |
| CYP2C19*17 | rs12248560 | -806C>T | 5’, high transcription, gain-of function, increased in vivo PPI metabolism in some studies | increased | increased | 2 | 379  (23%) | 246  (22%) | | | 288 (2%) | 1000GP | [[23](#_ENREF_23), [32](#_ENREF_32), [35-37](#_ENREF_35), [40](#_ENREF_40), [41](#_ENREF_41)] |
| CYP2C19*5 | rs56337013 | 1297C>T | R433W in heme-binding region, no holoprotein; no in vitro omeprazole activity | none | none | 3 | (<1%) | (0%) | | | 303 (<1%) | [[20](#_ENREF_20), [21](#_ENREF_21)] | *[*[*19*](#_ENREF_19)*]* |
| CYP2C19*6 | rs72558184 | 395G>A | R132Q, no in vitro omeprazole activity | none | none | 3 | 172  (<1%) | 44  (0%) | | | 88 (0%) | *[*[*19*](#_ENREF_19)*,* [*24*](#_ENREF_24)*]* | *[*[*19*](#_ENREF_19)*,* [*24*](#_ENREF_24)*]* |
| CYP2C19*8 | rs41291556 | 358T>C | W120R, low/no omeprazole activity in vitro | none/ reduced | none | 3 | 379  (0.3%) | 246  (0.4%) | | | 288 (0%) | 1000GP | *[*[*19*](#_ENREF_19)*,* [*22*](#_ENREF_22)*]* |
| CYP2C19*9 | rs17884712 | 431G>A | R144H, high omeprazole km in vitro | slightly reduced | ND | 3 | 379  (0%) | 246 (1.2%) | | | 288 (0%) | 1000GP | *[*[*19*](#_ENREF_19)*]* |
| CYP2C19*10 | rs6413438 | 680C>T | P227L, low omeprazole activity and high km in vitro | reduced | ND | 3 | 379  (0%) | 246 (0%) | | | 288  (0.9%) | 1000GP | *[*[*19*](#_ENREF_19)*]* |
| CYP2C19*4 | rs28399504 | 1A>G | M1V, initiation error, no expression in vitro, in mephenytoin PM [[17](#_ENREF_17)], increased clopidogrel ADRs | none | none | 5n | 379  (0%) | 246  (0%) | | | 288 (0.2%) | 1000GP | [[1](#_ENREF_1), [2](#_ENREF_2), [17](#_ENREF_17), [18](#_ENREF_18)] |
| CYP2C19*7 | rs72558186 | intronic IVS5+2T>A | 5’ donor splice defect [[12](#_ENREF_12), [22](#_ENREF_22)], in 3% Caucasian mephenytoin PMs [[22](#_ENREF_22)] | none | none | 6se | 173 (<1%) | 44 (0%) | | | 86 (0%) | [[17](#_ENREF_17), [22](#_ENREF_22)] | [[12](#_ENREF_12), [22](#_ENREF_22)] |
| CYP2C19*12 | rs55640102 | 1473A>C | X491C, unstable protein in vitro [[25](#_ENREF_25)] | unstable protein | ND | 11 | 44 (0%) | 23 (4%) | | | 89 (0%) | ALFRED [[25](#_ENREF_25)] | ND |
| CYP2C19*16 | no rsID | 1324C>T | R442C, low holoprotein, low Vmax and CL omeprazole in vitro [[19](#_ENREF_19)] | reduced | ND | 12 | ND | ND | | | (<1%) | [[19](#_ENREF_19)] | [[19](#_ENREF_19)] |
| CYP2C19*18 | rs138142612 | 986G>A | R329H, low omeprazole activity in vitro [[19](#_ENREF_19)] | reduced | ND | 12 | ND | ND | | | (<1%) | [[19](#_ENREF_19)]  dbSNP | [[19](#_ENREF_19)] |
|  |  |  |  |  |  |  | 2276 ethnicity unknown (0%) | | | | |  |  |
| CYP2C19*19 | no rsID | 151A>G | S51G, high Km and low CL omeprazole in vitro [[19](#_ENREF_19)] | reduced | ND | 12 | ND | | ND | | (<1%) | [[19](#_ENREF_19)] | [[19](#_ENREF_19)] |
| CYP2C19*SD | rs4244285b | 681G>C | P227P | ND | ND | 13 | ND | | ND | | ND | dbSNP | ND |
| CYP2C19 G439X | rs5787121 | 1314G>del | G439X, frameshift | ND | ND | 13 | 24 multiethnic (2%) | | | | | dbSNP | ND |
| CYP2C19*13 | rs17879685 | 1228C>T | R410C [[25](#_ENREF_25)], normal omeprazole activity in vitro [[19](#_ENREF_19)] | normal | ND | 14 | 156 (0%) | | 49 (1%) | | 88 (0%) | dbSNP [[25](#_ENREF_25)] | [[19](#_ENREF_19)] |
| CYP2C19*14 | rs55752064 | 50T>C | L17P [[25](#_ENREF_25)], normal omeprazole activity in vitro [[19](#_ENREF_19)] | normal | ND | 14 | 45 (0%) | | (2%) | | 200 (0%) | [[25](#_ENREF_25)]  ALFRED | [[19](#_ENREF_19)] |
| CYP2C19*15 | rs17882687 | 55A>C | I19L [[25](#_ENREF_25)], normal omeprazole activity in vitro [[19](#_ENREF_19)] | normal | ND | 14 | 43 (1%) | | 16 (5%) | | 96 (3%) | [[25](#_ENREF_25)] ALFRED | [[19](#_ENREF_19)] |
| CYP2C19 M74T | rs28399505 | 221T>C | M74T, normal omeprazole activity in vitro [[19](#_ENREF_19)] | normal | ND | 14 | (0%) | | (0%) | | 200 (<1%) | [[19](#_ENREF_19)] ALFRED | [[19](#_ENREF_19)] |
| CYP2C19 E122A | rs17885179 | 356A>C | E122A, high km and low CL, but near normal omeprazole activity in vitro [[19](#_ENREF_19)] | normal | ND | 14 | ND | | singleton | | ND | dbSNP | [[19](#_ENREF_19)] |
|  |  |  |  |  |  |  | 2276 ethnicity unknown (<1%) | | | | |  |  |
| CYP2C19 F168L | rs28399510 | 502T>C | F168L, low CL, but near normal omeprazole activity in vitro [[19](#_ENREF_19)] | normal | ND | 14 | (<1%) | | (0%) | 200 (0%) | | [[19](#_ENREF_19)] ALFRED | [[19](#_ENREF_19)] |

ND = no data/not determined, PM = poor metabolizer, UM = ultra-rapid metabolizer, PPI = proton pump inhibitor, ADR = adverse drug reaction, Vmax = maximum metabolic velocity, CL = clearance, CLint = intrinsic clearance, Km = concentration at half-Vmax. ^a^ **=** evidence code assigned to each allele indicates the strength and type of evidence for a drug-related phenotype based on the scheme presented in **Table 1**(green indicates clinically relevant evidence, yellow=potential clinical relevance, light orange= clinical relevance unknown, and dark orange= clinical relevance unsupported). ^b^ = rs4244285 is tri-allelic (681G>A/C), where A defines CYP2C19*2 and C defines CYP2C19*SD; ^c^ = 636G>A/T (W212X/C) is tri-allelic, where A defines CYP2C19*3 (rs4986893) and T is unreported by dbSNP, **^d^** = CYP2C19*1 allele frequency is estimated and takes into account the allele frequencies of other variants reported in this table with an evidence code ≤ 7, ^e^ = allele frequency from 1000 Genomes Project phase 1, HapMap, dbSNP, ALFRED, or specific reference.

**Table S7. *CYP2C19*-PPIs predicted genotype-phenotype interpretations (extended punnett square)**

| **CYP2C19 allele**  **(freq. %)** | **CYP2C19*1** | **CYP2C19*2** | **CYP2C19*3** | **CYP2C19*4** | **CYP2C19*5** | **CYP2C19*6** | **CYP2C19*7** | **CYP2C19*8** | **CYP2C19*9** | **CYP2C19*10** | **CYP2C19*17** |
| --- | --- | --- | --- | --- | --- | --- | --- | --- | --- | --- | --- |
| **CYP2C19*1**  **(61.3)** | *1/*1 (38%) EM | *1/*2 (18%) IM | *1/*3 (<0.1%) IM | *1/*4 (<0.1%) IM | *1/*5 (<0.1%) IM | *1/*6 (<0.1%) IM | *1/*7 (<0.1%) IM | *1/*8 (0.4%)  IM | *1/*9 (<0.1%) IM | *1/*10 (<0.1%) IM | *1/*17 (29%) unk |
| **CYP2C19*2**  **(15)** |  | *2/*2 (2.3%) PM | *2/*3 (<0.1%) PM | *2/*4 (<0.1%) PM | *2/*5 (<0.1%) PM | *2/*6 (<0.1%) PM | *2/*7 (<0.1%) PM | *2/*8 (0.1%) PM | *2/*9 (<0.1%)  PM | *2/*10 (<0.1%)  PM | *2/*17 (7%) IM |
| **CYP2C19*3**  **(<0.1)** |  |  | *3/*3 (<0.1%) PM | *3/*4 (<0.1%)  PM | *3/*5 (<0.1%)  PM | *3/*6 (<0.1%)  PM | *3/*7 (<0.1%)  PM | *3/*8 (<0.1%)  PM | *3/*9 (<0.1%)  PM | *3/*10 (<0.1%)  PM | *3/*17 (<0.1%) IM |
| **CYP2C19*4**  **(<0.1)** |  |  |  | *4/*4 (<0.1%)  PM | *4/*5 (<0.1%)  PM | *4/*6 (<0.1%) PM | *4/*7 (<0.1%)  PM | *4/*8 (<0.1%)  PM | *4/*9 (<0.1%)  PM | *4/*10 (<0.1%)  PM | *4/*17 (<0.1%)  IM |
| **CYP2C19*5**  **(<0.1)** |  |  |  |  | *5/*5 (<0.1%)  PM | *5/*6 (<0.1%)  PM | *5/*7 (<0.1%)  PM | *5/*8 (<0.1%)  PM | *5/*9 (<0.1%)  PM | *5/*10 (<0.1%)  PM | *5/*17 (<0.1%)  IM |
| **CYP2C19*6**  **(<0.1)** |  |  |  |  |  | *6/*6 (<0.1%)  PM | *6/*7 (<0.1%)  PM | *6/*8 (<0.1%)  PM | *6/*9 (<0.1%)  PM | *6/*10 (<0.1%)  PM | *6/*17 (<0.1%)  IM |
| **CYP2C19*7**  **(<0.1)** |  |  |  |  |  |  | *7/*7 (<0.1%)  PM | *7/*8 (<0.1%)  PM | *7/*9 (<0.1%)  PM | *7/*10 (<0.1%)  PM | *7/*17 (<0.1%)  IM |
| **CYP2C19*8**  **(0.3)** |  |  |  |  |  |  |  | *8/*8 (<0.1%)  PM | *8/*9 (<0.1%)  PM | *8/*10 (<0.1%)  PM | *8/*17 (0.1%)  IM |
| **CYP2C19*9**  **(<0.1)** |  |  |  |  |  |  |  |  | *9/*9 (<0.1%)  PM | *9/*10 (<0.1%)  PM | *9/*17 (<0.1%) IM |
| **CYP2C19*10**  **(<0.1)** |  |  |  |  |  |  |  |  |  | *10/*10 (<0.1%)  PM | *10/*17 (<0.1%)  IM |
| **CYP2C19*17**  **(23.0)** |  |  |  |  |  |  |  |  |  |  | *17/*17 (5%) UM |

Approximate expected genotype frequencies have been estimated based upon reported allele frequencies for Caucasians (Table S6) and Hardy-Weinberg principles. Each potential CYP2C19 genotype has been assigned a predicted drug metabolizing phenotype based on predicted metabolic activity of individual alleles (normal, enhanced or reduced enzymatic function) such that extensive metabolizer (EM) is defined as 2 normal activity alleles; ultra-rapid metabolizer (UM) as 2 enhanced activity alleles; intermediate metabolizer (IM) as 1 normal or enhanced activity allele with 1 reduced activity allele; and poor metabolizer (PM) as 2 reduced activity alleles. The metabolizer phenotype for one normal and one enhanced activity alleles is currently unknown (unk). Color shading is used to define expected drug response based on published outcomes data such that: blue indicates likely normal drug response, orange indicates intermediate drug elimination; yellow indicates poor drug elimination, purple indicates increased drug elimination, and pink indicates undetermined metabolizer status and drug elimination phenotype. Compared to wildtype EMs, IMs and/or PMs have been associated with improved PPI efficacy as measured by intragastric pH inhibition, duration of inhibition, and cure rates for GERD and *H. pylori*, while UMs have been associated with decreased PPI efficacy at the normal dose. Irrespective of metabolizer status as determined by CYP2C19 genotype, PPIs are contraindicated for use with other drugs dependent upon the same metabolic pathway or low gastric pH; and this effect is further mitigated in reduced metabolizers.

**Table S8. Studies of CYP2C9 Variation for Clinical Outcomes in Celecoxib-Treated Patients**

| **Study** | **Subjects, Drug** | **Comparison Groups** | **Key Endpoint Results** | **Conclusions and Comments** |
| --- | --- | --- | --- | --- |
| [[42](#_ENREF_42)] | n=33 cases,  n=50 controls,  multiple NSAIDs extensively metabolized by CYP2C9 | **CYP2C9**  n=42: *1  n=17: *2  n=10: *3  genotypes  n=12: wild type  n=18: het variant  n=3: hom variant | **Carrier risk of GI bleeding for extensively metabolized NSAIDs**  OR=2.5 het variant carriers  OR=3.7 hom variant carriers, p<0.015 OR=2.60 (1.10–6.19) all variant carriers, chi-squared = 5.68, p=0.017  **Carrier risk of GI bleeding for celecoxib**  ADRs (n=3) vs (n=4) no ADRs,  genotype distribution unknown | A large study investigated the risk of acute GI bleeding with NSAID use in 94 ADR cases and 128 ADR controls for CYP2C9-metabolized NSAIDs. Among 83 users of the extensively metabolized NSAIDs (aceclofenac, celecoxib, diclofenac, ibuprofen, indomethazine, lornoxicam, piroxicam, and naproxen), significantly more carriers of *2 and *3 had risk of bleeding relative to no bleeding. However, only 7 celecoxib users were tested, and CYP2C9 genotype was not reported in these individuals. This data is therefore observational. |
| [[43](#_ENREF_43)] | n=4 pediatric solid tumor patients, celecoxib | **CYP2C9**  n=2 *1/*1  n=1 *1/*2  n=1 *3/*3 | **Single-dose AUC, Cl, Cmax, t1/2**  *1/*1: 2418, 10760, 0.7, 3.2  *1/*1: 1865, 11599, 0.6, 6.5  *1/*2: 3713, 13037, 0.7, 2.9  *3/*3: 2940, 108251, 0.09, 29.9  **Steady-state AUC, Cl, Cmax, t1/2**  *1/*1: 1677, 10905, 0.7, 4.1  *1/*1: 2079, 14031, 0.4, 3.0  *1/*2: 3451, 11119, 0.6, 3.8  *3/*3: 8461, 93725, 0.02, 41.2 | Pediatric patients were given 250mg/m2 celecoxib twice daily and assessed for CYP2C9 genotype influence for steady-state and single-dose PK. The *3 homozygote showed 10-fold greater AUC, one-tenth the clearance, and 7-fold greater half-life for a single dose compared to the mean values for other genotypes. At steady-state, the Cmax was 6-7-fold greater, and AUC was 10-fold higher in the *3/*3 carrier compared to the other genotypes. The *1/*2 heterozygote showed slightly greater drug exposure than wild type for single dose pharmacokinetics, and comparable pharmacokinetic values at steady state (most values within the range of wild type), relative to wild type. No acute cardiovascular or other toxicity was observed in this short-term study. |
| [[44](#_ENREF_44)] | n=26 ADR cases,  n=52 ADR controls,  Italians,  n=15 celecoxib,  n=33 diclofenac,  n=9 ibuprofen,  n=6 naproxen,  n=15 piroxicam | **CYP2C9**  n=58 *1/*1 n=15 *1/*2  n=12 *1/*3  n=3 *2/*3  n=0 *2/*2, *3/*3 | **Odds ratio bleeding vs no bleeding (n)**  *1/*2: 26.9% (7) vs 15.4% (8), OR=3.8, p=0.036  *1/*3: 34.6% (9) vs 5.8% (3), OR=12.9, p<0.001  *2/*3: 3.8% (1) vs 3.8% (2), NS  **Odds ratio bleeding risk**  *3 carriers: 7.3 | A case-control study of patients endoscopically tested for NSAID-induced gastroduodenal bleeding from acute (<1 month) doses of five CYP2C9-metabolized NSAIDs (celecoxib, diclofenac, ibuprofen, naproxen, piroxicam) showed the *1/*2 and *1/*3 genotypes and *3 allele associated with increased risk of bleeding. Variants in other genes were not associated with NSAID-induced bleeding (e.g. APOE, ACE). *However, because this study was not parsed for effects by drug, it is not possible to determine the influence of CYP2C9 variants on celecoxib treatment alone.* |
| [[45](#_ENREF_45)] | n=1,660 colorectal adenoma patients,  celecoxib, placebo | **CYP2C9**  n=1,102: wild type  n=357: *2, non*3 group (*1/*2, *2/*2)  n=201: *3 group (*1/*3, *2/*3, *3/*3)  wild type: 67%  *2 carriers: 21%  *3 carriers: 12%  n=346: *1/*2  n=11: *2/*2  n=17: *2/*3  n=78: *1/*3  n=6: *3/*3  n=499: placebo n=498: low dose  n=500: high dose | **Risk of adenoma recurrence at year 3**  high vs low dose  n=498 all genotypes: RR=0.82, p=0.03  wild type: RR=0.89, NS  *1/*2, *2/*2: RR=0.83, NS  *1/*3, *2/*3, *3/*3: RR=0.51, p=0.01 (19.7% greater reduction)  *1/*3, *2/*3, *3/*3 vs wild type: NS, p=0.09  high dose (vs placebo)  n=500 all genotypes: RR=0.54, p<0.001  n=328 wild type: RR=0.56, p<0.001  n=108 *1/*2, *2/*2: RR=0.54, p<0.001  n=64 *1/*3, *2/*3, *3/*3: RR=0.41, p<0.001  low dose (vs placebo)  n=498 all genotypes: RR=0.65, p<0.001  n=330 wild type: RR=0.64, p<0.001  n=99 *1/*2, *2/*2: RR=0.63, p=0.003 n=64 *1/*3, *2/*3, *3/*3: RR=0.76, p=0.18, NS  **Risk of cardiovascular ADR**  high dose (vs placebo)  all genotypes: RR=1.69  wild type: RR=1.37  *1/*2, *2/*2: RR=2.75  *1/*3, *2/*3, *3/*3: RR=2.69 | The Adenoma Prevention with Celecoxib (APC) trial was carried out after removal of colorectal adenomas in 1,660 patients at high risk for recurrence. Patients were randomly assigned placebo, 200 mg twice daily celecoxib (low dose), or 400 mg twice daily celecoxib (high dose), and assessed for adenoma recurrence at 3 years.  RESULTS: Risk of adenoma recurrence with low or high celecoxib doses in comparing *2 or *3 carriers with wild type were not significantly different; but at the high dose, *3 carriers showed about 20% greater reduction in the risk of adenoma recurrence compared to low dose. Treatment improved outcome in all genotypes, with preferentially greater improvement at high dose among *3 carriers. At low doses, *3 carriers showed no significant efficacy compared to placebo for unclear reasons. The cumulative incidence of cardiovascular and thrombotic events was increased in both *2 and *3 carriers at high doses compared to wild type. |

ND = not determined/no data, NS = non-significant, het = heterozygote, hom = homozygote, NSAID = non-steroidal anti-inflammatory drug, CRC = colorectal cancer, GI = gastrointestinal, ADR = adverse drug reaction, AUC = area under the plasma concentration-time curve, RR = relative risk.

**Table S9. Metabolic phenotypes, frequency and evidence scoring of gene allelic variants for *CYP2C9*-celecoxib**

| **Allele** | **Key Variant dbSNP ID** | **Nucleotide Change (cDNA)** | **Mutation Effect** | | **Enzyme Activity** | | **Evidence Code**^a^ | **Allele Frequency (# Subjects (%))** | | | | | | **Key References (PMID#)** |
| --- | --- | --- | --- | --- | --- | --- | --- | --- | --- | --- | --- | --- | --- | --- |
|  |  |  |  |  | **In Vitro/ In Vivo** | **Metabolic Phenotype** |  | **Caucasian** | **African** | | **Asian** | | **Reference ^b^** |  |
| CYP2C9*1 | wild type | reference | lacks variants below | | normal | normal | 1 | (82%)^d^ | (92%)^d^ | | (96%)^d^ | | Estimated^d^ | reference in studies below |
| CYP2C9*2 | rs1799853 | 430C>T | Arg144Cys, ↓↔ celecoxib,  ↑ADR at 400mg/bid | | in vitro modest decrease/ normal | decrease | 1 | 379  (12%) | 246  (2%) | | 288  (0%) | | 1000GP | [[45-48](#_ENREF_45)] |
| CYP2C9*3 | rs1057910 | 1075A>C | Ile359Leu, ↓ celecoxib,  ↑ADR at 400mg/bid  slight ↑efficacy | | decrease | decrease | 1 | 379  (6%) | 246  (1%) | | 288  (4%) | | 1000GP | [[43](#_ENREF_43), [45-50](#_ENREF_45)] |
| CYP2C9*5 | [rs28371686](http://www.ncbi.nlm.nih.gov/projects/SNP/snp_ref.cgi?rs=28371686) | 1080C>G | Asp360Glu, ↓ warfarin, diclofenac, lauric acid, losartan, phenytoin | | decrease | decrease | 4scd | 379  (0%) | 246  (2%) | | 288  (0%) | | 1000 GP | [[51-54](#_ENREF_51)] |
| CYP2C9*11 | rs28371685 | 1003C>T | Arg335Trp,  ↓ tolbutamide, phenytoin, warfarin | | decrease | decrease | 4scd | 379  (0%) | 246  (3%) | | 288  (0%) | | 1000GP | [[54-56](#_ENREF_54)] |
| CYP2C9*6 | rs9332131 | 818A>del | K273 frameshift,  ↓ aspirin, losartan, warfarin, phenytoin | | decrease | decrease | 5n | 172  (0%) | 79  (<1%) | | 504  (0%) | | [[51](#_ENREF_51), [57](#_ENREF_57)] | [[54](#_ENREF_54), [56-59](#_ENREF_56)] |
| CYP2C9*13 | rs72558187 | 269T>C | Leu90Pro,  ↓luciferin, lornoxicam diclofenac, tolbutamide | | decrease | decrease | 9 | ND | 300 (0%) | | 147 (2%) | | [[60](#_ENREF_60), [61](#_ENREF_61)] | [[61-63](#_ENREF_61)] |
| CYP2C9*8 | rs7900194 | 449G>A | | Arg150His,  ↓ phenytoin, warfarin | decrease | ND | 10 | 379  (0%) | 246  (5%) | | 288  (0%) | 1000GP | | [[54](#_ENREF_54), [56](#_ENREF_56)] |
| CYP2C9*14 | rs72558189 | 374G>A | | Arg125His,  ↓ tolbutamide, warfarin | decrease | ND | 10 | ND | ND | | 59 (0%) | [[64](#_ENREF_64)] | | [[64](#_ENREF_64), [65](#_ENREF_65)] |
|  |  |  |  |  |  |  |  | 26 (2%) Indians | | | |  |  |  |
| CYP2C9*12 | [rs9332239](http://www.ncbi.nlm.nih.gov/projects/SNP/snp_ref.cgi?rs=9332239) | 1465C>T | | Pro489Ser,  ↓ tolbutamide | decrease | decrease | 11 | 379  (0%) | 246  (0%) | 288  (0%) | | 1000GP | | [[55](#_ENREF_55)] |
| CYP2C9*15 | rs72558190 | 485C>A | | Ser162X, frameshift, no expression, in vitro unstable protein,  ↔ warfarin | unstable protein | decrease | 11 | ND | ND | 59 (0%) | | [[64](#_ENREF_64)] | | [[64](#_ENREF_64), [65](#_ENREF_65)] |
|  |  |  |  |  |  |  |  | 26 (2%) Indians | | | |  |  |  |
| CYP2C9*16 | rs72558192 | 895A>G | | Thr299Ala,  ↓ tolbutamide,  ↔ warfarin | varied | varied | 11 | ND | ND | 59 (<1%) | | [[64](#_ENREF_64)] | | [[64](#_ENREF_64), [65](#_ENREF_65)] |
| CYP2C9*25 | rs72558188 | 353del>A | | Lys118ArgX9, frameshift, null allele, protein truncation, no expression, no diclofenac activity | unstable protein | ND | 11 | ND | ND | 263 (<1%) | | [[66](#_ENREF_66)] | | [[66](#_ENREF_66)] |
| CYP2C9*4 | rs56165452 | 1076T>C | | Ile359Thr^c^,  ↓ diclofenac, phenytoin | decrease | decrease | 12 | 140 (0%) | 300 (0%) | 132 (<1%) | | [[52](#_ENREF_52), [60](#_ENREF_60), [67](#_ENREF_67)] | | [[59](#_ENREF_59)] |
| CYP2C9  Y358C | [rs1057909](http://www.ncbi.nlm.nih.gov/projects/SNP/snp_ref.cgi?rs=1057909) | 1073A>G | | Tyr358Cys | ND | ND | 13 | 60  (0%) | 60 (0%) | 85 (0%) | | HapMap | | ND |
| CYP2C9 A441A | rs2017319 | 1323C>T | | Ala441Ala | ND | ND | 13 | 378  (0%) | 246  (9%) | 288  (0%) | | 1000GP | | ND |
| CYP2C9 G475G | rs1057911 | 1425A>T | | Gly475Gly | ND | ND | 13 | 379  (6%) | 246  (1%) | 288  (4%) | | 1000GP | | ND |
| CYP2C9*7 | rs67807361 | 55C>A | | Leu19Ile,  ↔ tolbutamide | normal | normal | 14 | 43  (0%) | 44  (0%) | 88  (0%) | | PGKB | | [[55](#_ENREF_55)] |
| CYP2C9*9 | rs2256871 | 752A>G | | His251Arg,  ↔ phenytoin | normal | normal | 14 | 45  (0%) | 45  (13%) | 88  (0%) | | PGKB | | [[54](#_ENREF_54)] |
| CYP2C9*10 | rs9332130 | 815A>G | | Glu272Gly, ↔ tolbutamide | normal | normal | 14 | 58  (0.9%) | 58  (0%) | 87  (0%) | | HapMap | | [[55](#_ENREF_55)] |

ND = no data/not determined, ADR = adverse drug reaction, bid = twice daily dose, a **=** evidence code assigned to each allele provides an indication of the strength and type of evidence for a drug-related phenotype and is based on the scheme presented in **Table 1** (green indicates clinically relevant evidence, yellow=potential clinical relevance, light orange= clinical relevance unknown, and dark orange= clinical relevance unsupported)., b **=** allele frequency from1000 Genome Project phase 1, PGKB, or specific reference, ^c^ = highly conserved nucleotide, **^d^** = CYP2C9*1 allele frequency is estimated and takes into account the allele frequencies of other variants reported in this table with an evidence code ≤ 7.

**Table S10. *CYP2C9*-Celecoxib predicted genotype-phenotype interpretations (extended punnett square)**

| **Allele**  **(frequency)** | **CYP2C9*1** | **CYP2C9*2** | **CYP2C9*3** | **CYP2C9*5** | **CYP2C9*6** | **CYP2C9*11** |
| --- | --- | --- | --- | --- | --- | --- |
| **CYP2C9*1**  **(82%)** | *1/*1 (67%)  EM | *1/*2 (20%)  IM | *1/*3 (10%)  IM | *1/*5 (<0.1%)  IM | *1/*6 (<0.1%)  IM | *1/*11 (<0.1%)  IM |
| **CYP2C9*2**  **(12%)** |  | *2/*2 (1.4%)  PM | *2/*3 (1.4%)  PM | *2/*5 (<0.1%)  PM | *2/*6 (<0.1%)  PM | *2/*11 (<0.1%)  PM |
| **CYP2C9*3**  **(6%)** |  |  | *3/*3 (0.4%)  PM | *3/*5 (<0.1%)  PM | *3/*6 (<0.1%)  PM | *3/*11 (<0.1%)  PM |
| **CYP2C9*5**  **(<0.1%)** |  |  |  | *5/*5 (<0.1%)  PM | *5/*6 (<0.1%)  PM | *5/*11 (<0.1%)  PM |
| **CYP2C9*6**  **(<0.1%)** |  |  |  |  | *6/*6 (<0.1%)  PM | *6/*11 (<0.1%)  PM |
| **CYP2C9*11**  **(<0.1%)** |  |  |  |  |  | *11/*11 (<0.1%)  PM |

Approximate expected genotype frequencies have been estimated based upon reported allele frequencies for Caucasians and Hardy Weinberg principals. Genotypes are assigned a predicted drug metabolizing phenotype based on the metabolic activity of individual alleles (normal or reduced function) for celecoxib or other substrates. Each potential CYP2C9 genotype has been assigned a predicted drug metabolizing phenotype based on the predicted metabolic phenotype of individual alleles. The *1 allele shows normal activity; *2 , *3, *5, *6 and *11 show reduced activity. Extensive metabolizers (EMs) have 2 normal alleles; intermediate metabolizers (IMs) have 1 normal and 1 reduced-activity allele; and poor metabolizers (PMs) have 2 greatly reduced-activity alleles. Color defines the predicted phenotypic outcome (adverse drug reactions and increased efficacy only for all PMs): blue = likely normal response; yellow increased risk of renal and cardiovascular ADR; pink = unknown.

**Table S11.** **Metabolic phenotypes, frequency and evidence scoring of gene allelic variants for *CYP2C9*-warfarin**

| **Allele** | **Key Variant dbSNP ID** | **Nucleotide Change (cDNA)** | **Mutation Effect** | **Enzyme Activity^b^** | | **Evidence code**^a^ | **Allele Frequency (# Subjects (%))** | | | | **Key References**  **(PMID#)** |
| --- | --- | --- | --- | --- | --- | --- | --- | --- | --- | --- | --- |
|  |  |  |  | **In vitro/ In vivo** | **Metabolic Phenotype** |  | **Caucasian** | **African** | **Asian** | **Reference**^c^ |  |
| **CYP2C9*1** | wild type | reference | lacks variants below | normal | normal | 1 | (82%)^c^ | (87%)^c^ | (94%)^c^ | Estimated**^c^** | reference in studies below |
| **CYP2C9*2** | rs1799853 | 430C>T | Arg144Cys, Vmax about 50% of wild type | decreased | reduced | 1 | 379  (12%) | 246  (2%) | 288  (0%) | 1000GP | [[46](#_ENREF_46), [68-70](#_ENREF_68)] |
| **CYP2C9*3** | rs1057910 | 1075A>C | Ile359Lys, results in 10% clearance of warfarin compared to *1, carriers require lower warfarin dose | decreased | reduced | 1 | 379  (6%) | 246  (1%) | 288  (4%) | 1000GP | [[49](#_ENREF_49), [68](#_ENREF_68), [69](#_ENREF_69), [71](#_ENREF_71)] |
| **CYP2C9*5** | rs28371686 | 1080C>G | Asp360Glu, low warfarin clearance, carriers need lower warfarin dose | decreased | reduced | 1 | 379  (0%) | 246  (2%) | 288  (0%) | 1000 GP | [[52](#_ENREF_52), [56](#_ENREF_56)] |
| **CYP2C9*6** | rs9332131 | 818A>del | carriers need lower warfarin dose | none | reduced | 1 | 172  (0%) | 79 (<1%) | 504 (0%) | [[51](#_ENREF_51), [57](#_ENREF_57)] | [[56](#_ENREF_56)] |
| **CYP2C9*8** | rs7900194 | 449G>A | Arg150His, carriers need lower warfarin dose | decreased | reduced | 1 | 379  (0%) | 246  (5%) | 288  (0%) | 1000GP | [[54](#_ENREF_54), [56](#_ENREF_56)] |
| **CYP2C9*11** | rs28371685 | 1003C>T | carriers need lower warfarin dose | decreased | reduced | 1 | 379  (0%) | 246 (3%) | 288 (0%) | 1000GP | [[56](#_ENREF_56), [72](#_ENREF_72)] |
| **CYP2C9*14** | rs72558189 | 374G>A | ^m^ Arg125His, 80-90% lower tolbutamide activity, carrier needed lower warfarin dose | decreased | reduced | 1 | ND | ND | 26 (2%)^d^ | [[64](#_ENREF_64)] | [[64](#_ENREF_64), [65](#_ENREF_65)] |
| **CYP2C9*13** | rs72558187 | 269T>C | seen in a Chinese PM for lornoxicam and tolbutamide | decreased | reduced | 9 | ND | 300 (%) | 147 (1%) | [[60](#_ENREF_60), [61](#_ENREF_61)] | [[61](#_ENREF_61)] |
| **CYP2C9*12** | rs9332239 | ^e^1465C>T | Pro489Ser, lower tolbutamide activity | decreased | reduced | 11 | 379  (0%) | 246  (0%) | 288  (0%) | 1000GP | [[55](#_ENREF_55)] |
| **CYP2C9*15** | rs72558190 | ^f^485C>A | Ser162X, frameshift, premature stop codon, unstable protein in vitro | unstable protein | reduced | 11 | ND | ND | 59 (0%) | [[64](#_ENREF_64)] | [[64](#_ENREF_64), [65](#_ENREF_65)] |
|  |  |  |  |  |  |  | 26 (2%) Indians | | | |  |
| **CYP2C9*16** | rs72558192 | ^g^ 895A>G | Thr299Ala, 80-90% lower tolbutamide activity | decreased | reduced | 11 | ND | ND | 59 (<1%) | [[64](#_ENREF_64)] | [[64](#_ENREF_64), [65](#_ENREF_65)] |
| **CYP2C9*25** | rs72558188 | ^h^353_362delAGAAATGGAA | frameshift, premature stop codon, unstable protein in vitro | unstable protein | reduced | 11 | ND | ND | 263 (<1%) | [[66](#_ENREF_66)] | [[66](#_ENREF_66)] |
| **CYP2C9*4** | rs56165452 | 1076A>G | **^j^** Ile359Thr, diclofenac clearance 20-30% of wild type | decreased | reduced | 12scd | 140  (0%) | 300 (0%) | 132 (<1%) ^i^ | [[52](#_ENREF_52), [60](#_ENREF_60), [67](#_ENREF_67)] | [[59](#_ENREF_59), [67](#_ENREF_67)] |
| **CYP2C9 Y358C** | rs1057909 | A>G | Tyr358Cys | ND | ND | 13 | 60  (0%) | 60 (0%) | 85 (0%) | HapMap | ND |
| **CYP2C9 A441A** | rs2017319 | 1323C>T | Ala441Ala | ND | ND | 13 | 378  (0%) | 246  (9%) | 288  (0%) | 1000GP | ND |
| **CYP2C9 G475G** | rs1057911 | 1425A>T | Gly475Gly | ND | ND | 13 | 379  (6%) | 246  (1%) | 288  (4%) | 1000GP | ND |
| **CYP2C9*7** | rs67807361 | 55C>A | Leu19Ile, tolbutamide activity similar to wild type | normal | ND | 14 | 43  (0%) | 44 (0%) | 88  (0%) | PGKB | [[55](#_ENREF_55)] |
| **CYP2C9*9** | rs2256871 | 752A>G | His251Arg, tolbutamide activity similar to wild type | normal | ND | 14 | 45 (0%) | 45 (13%) | 88 (0%) | PGKB | [[55](#_ENREF_55)] |
| **CYP2C9*10** | rs9332130 | 815A>G | E272G, tolbutamide activity similar to wild type | normal | ND | 14 | 58  (0.9%) | 58 (0%) | 87 (0%) | HapMap | [[55](#_ENREF_55)] |

ND = no data/not determined; PM = poor metabolizer; tolbutamide is an antidiabetic drug and universal CYP2C substrate, and diclofenac is an NSAID.

^a^ **=** evidence code assigned to each allele provides an indication of the strength and type of evidence for a drug-related phenotype and is based on the scheme presented in **Table 1** (green indicates clinically relevant evidence, yellow=potential clinical relevance, light orange= clinical relevance unknown, and dark orange= clinical relevance unsupported). For alleles with multiple types of supporting data, the code for the greatest strength of evidence is assigned,

^b^ = in vitro enzyme kinetics (e.g. Vmax) for substrates measured for recombinant proteins expressed in bacterial/yeast or mammalian cDNA expression system; In vivo studies have evaluated the effect of CYP2C9 genotype on warfarin clearance and maintenance dose requirements in patients on warfarin therapy,

^c^ = Allele frequency from 1000 Genome Project phase 1, HapMap, PGKB, or specific reference; CYP2C9*1 allele frequency is estimated and takes into account the allele frequencies of other variants reported in this table with an evidence code ≤ 7

^d^ = identified in one South Asian subject from Indian, not observed in 59 Chinese or 37 Malays.

^e^ = seen in one subject of unknown ethnicity from the NIH DNA Polymorphism Discovery Resource

^f^ = seen in one South Asian patient from India who did not require a lower warfarin dose than the ethnic group mean,

^g^ = seen in one Chinese patient who did not require a lower warfarin dose than the ethnic group mean,

^h^ = seen in a heterozygous Japanese diabetic treated with glimepiride with unknown metabolism phenotype

^j^ = seen in a Japanese patient requiring a low phenytoin dose. It is at the same residue (Ile359Thr) as the mutation giving rise to CYP2C9*3 (Ile359Leu). Residue 359 is in the substrate recognition site of CYP2C9. Both 359Leu and 359Thr show similar reduction in intrinsic clearance of diclofenac. The effect of 359Thr on S-warfarin clearance has not been studied,

^k^ = seen in 1% of 159 Caucasian patients on warfarin [[72](#_ENREF_72)],

^m^ = carrier required lower warfarin dose than the ethnic group mean.

**Table S12.** ***CYP2C9*-warfarin predicted genotype-phenotype interpretations (extended Punnett square)**

| **Allele (frequency %)** | **CYP2C9*1** | **CYP2C9*2** | **CYP2C9*3** | **CYP2C9*5** | **CYP2C9*6** | **CYP2C9*8** | **CYP2C9*11** | **CYP2C9*14** |
| --- | --- | --- | --- | --- | --- | --- | --- | --- |
| **CYP2C9*1**  **(82)** | *1/*1 (67%)  EM | *1/*2 (20%)  IM | *1/*3 (10%)  PM | *1/*5 (<0.1%)  PM | *1/*6 (<0.1%)  PM | *1/*8 (<0.1%)  IM | *1/*11 (<0.1%)  IM | *1/*14 (<0.1%)  IM |
| **CYP2C9*2**  **(12)** |  | *2/*2 (1.4%)  PM | *2/*3 (1.4%)  PM | *2/*5 (<0.1%)  PM | *2/*6 (<0.1%)  PM | *2/*8 (<0.1%)  PM | *2/*11 (<0.1%)  PM | *2/*14 (<0.1%)  PM |
| **CYP2C9*3**  **(6)** |  |  | *3/*3 (0.4%)  PM | *3/*5 (<0.1%)  PM | *3/*6 (<0.1%)  PM | *3/*8 (<0.1%)  PM | *3/*11 (<0.1%)  PM | *3/*14 (<0.1%)  PM |
| **CYP2C9*5**  **(<0.1)** |  |  |  | *5/*5 (<0.1%)  PM | *5/*6 (<0.1%)  PM | *5/*8 (<0.1%)  PM | *5/*11 (<0.1%)  PM | *5/*14 (<0.1%)  PM |
| **CYP2C9*6**  **(<0.1)** |  |  |  |  | *6/*6 (<0.1%)  PM | *6/*8 (<0.1%)  PM | *6/*11 (<0.1%)  PM | *6/*14 (<0.1%)  PM |
| **CYP2C9*8**  **(<0.1)** |  |  |  |  |  | *8/*8 (<0.1%)  PM | *8/*11 (<0.1%)  PM | *8/*14 (<0.1%)  PM |
| **CYP2C9*11**  **(<0.1)** |  |  |  |  |  |  | *11/*11 (<0.1%)  PM | *11/*14 (<0.1%)  PM |
| **CYP2C9*14**  **(<0.1)** |  |  |  |  |  |  |  | *14/*14 (<0.1%)  PM |

Approximate expected genotype frequencies have been estimated based upon reported allele frequencies for Caucasians and Hardy Weinberg principals. Each potential CYP2C9 genotype has been assigned a predicted drug metabolizing phenotype based on predicted metabolic phenotype of individual alleles (*1 as normal drug metabolism; *2,*8 *11 and *14 as slow metabolizing alleles and *3, *5 and *6 as very slow metabolizing alleles) such that extensive metabolizer (EM) is defined as 2 alleles with normal metabolism; Intermediate metabolizers (IM) have 1 normal and 1 slow metabolizing allele; and Poor metabolizers (PM) have at least one very slow metabolizing allele or 2 slow metabolizing alleles. In general warfarin dosing requirements decrease by CYP2C9 phenotype from EM>IM>PM, but dosing is also dependent on other known genetic (e.g. VKORC1 and CYP4F2) and non-genetic (such as age, gender, body mass index and target INR) factors. Color gradient is a crude indicator of frequency with darker being more frequent than lighter, CYP2C9 allele frequency (grey); EM phenotype (blue); IM (yellow) and PM (orange) are those at increased risk of bleeding events.

**Table S13.** **Metabolic phenotypes, frequency and evidence scoring of gene allelic variants for *VKORC1*-warfarin**

| **Allele** | **Key variant**  **dbSNP ID** | **Nucleotide change (cDNA)** | **Mutation Effect** | **Enzyme Activity ^b^** | | **Evidence code**^a^ | **Allele Frequency (# subjects (%))** | | | | **Key References**  **(PMID#)** |
| --- | --- | --- | --- | --- | --- | --- | --- | --- | --- | --- | --- |
|  |  |  |  | **In vitro / In vivo** | **Metabolic Phenotype** |  | **Caucasian** | **African** | **Asian** | **Reference^ƒ^** |  |
| **^c^VKORC1*2 (A-Hap)** | rs9923231 | -1639G>A | reduced mRNA expression,  homozygous carriers require lower warfarin dose | warfarin sensitive | require lower warfarin dose | 1 | 119  (37%) | 96 (14%) | 120 (89%) | [[73](#_ENREF_73)] | [[73-75](#_ENREF_73)] |
| **VKORC1 R98W** | rs72547528 | 504C>T | seen in 2 unrelated patients with VKCFD2 | VKOR activity significantly reduced | ND | 11 | **^d^**192 (0%) | | | [[76](#_ENREF_76)] | [[76](#_ENREF_76), [77](#_ENREF_77)] |
| **VKORC1 L128R ^e^** | rs28940305 | 609T>G | seen in a patient with warfarin resistance | warfarin resistant | no response to warfarin | 12 | **^d^**192 (0%) | | | [[76](#_ENREF_76)] | [[76](#_ENREF_76)] |
| **VKORC1 R58G ^e^** | rs28940304 | 398A>G | seen in a patient with warfarin resistance | warfarin resistant | requires higher warfarin dose | 12 | **^d^**192 (0%) | | | [[76](#_ENREF_76)] | [[76](#_ENREF_76)] |
| **VKORC1**  **V29L ^e^** | rs28940302 | 311G>T | seen in a patient with warfarin resistance | warfarin resistant | requires higher warfarin dose | 12 | **^d^**192 (0%) | | | [[76](#_ENREF_76)] | [[76](#_ENREF_76)] |
| **VKORC1 V45A ^e^** | rs28940303 | 360T>C | seen in a patient with warfarin resistance | warfarin resistant | no response to warfarin | 12 | **^d^**192 (0%) | | | [[76](#_ENREF_76)] | [[76](#_ENREF_76)] |
| **VKORC1*1^g^** | “wild type” | reference | lacks other VKORC1 variants described in this table | ND | ND | 13 | 200  (<1%) | 23 (31%) | 24 (<1%) | [[75](#_ENREF_75)] | [[73](#_ENREF_73), [75](#_ENREF_75)] |
| **VKORC1*3 (B-Hap)** | rs7294 | 3730G>A | increased mRNA expression,  homozygous carriers require higher warfarin dose | partial warfarin resistant | require higher warfarin dose | 14 | 119  (35%) | 96 (43%) | 120 (10%) | [[73](#_ENREF_73)] | [[73](#_ENREF_73), [75](#_ENREF_75), [78](#_ENREF_78)] |
| **VKORC1*4 (B-Hap)** | rs17708472 | intron 1 698C>T | increased mRNA expression,  homozygous carriers require higher warfarin dose | partial warfarin resistant | require higher warfarin dose | 14 | 119  (24%) | 96 (6%) | 120 (0%) | [[73](#_ENREF_73)] | [[73](#_ENREF_73)] |

ND = no data/not determined, VKCFD2 = Vitamin K-dependent clotting factors, combined deficiency 2. ^a^ **=** evidence code assigned to each allele provides an indication of the strength and type of evidence for a drug-related phenotype and is based on the scheme presented in **Table 1** (green indicates clinically relevant evidence, yellow=potential clinical relevance, light orange= clinical relevance unknown, and dark orange= clinical relevance unsupported). For alleles with multiple types of supporting data, the code for the greatest strength of evidence is assigned; ^b^ = *in vitro* VKORC1 mRNA levels were quantified by quantitative PCR after extraction of total DNA and RNA from 53 human control liver specimens. DNA samples we used to assign individuals to A-haplotype (VKORC1*2) and B-haplotype (VKORC1*3 and VKORC1*4) groups, mRNA expression levels were measured and compared between the 3 genotype groups AA, AB and BB. I*n vivo* studies compared daily warfarin maintenance dosage between AA, AB and BB groups. ^c^ = VKORC1*2 is also identified by a number of tag-SNP (including the 1173C>T; dbSNP ID rs9934438) that are in almost perfect linkage disequilibrium with -1639G>A variant, ^d^ **=** mutations not detected in 384 human chromosomes of unknown ethnicity. **^e^ =** The rare mutations V29L, R58G, L128R and A45V were each identified in single warfarin resistant patient, all of whom were heterozygous mutation carriers. No information about other variants or haplotypes of the VKORC1 gene were provided for these individuals. Carriers of the V29L and R58G required high warfarin doses (HD) while carriers of L128R and A45V did not respond (NR) to warfarin at all doses tested. **^ƒ^** = Allele frequency from 1000 Genome Project, HapMap, dbSNP, ALFRED, or specific reference; **^g^** = The predicted metabolic phenotype of the *1 allele has not been specifically studied and so the phenotype of *1 carriers is unknown.

**Table S14. Predicted drug dosing phenotypes for VKORC1 genotypes**

| **VKORC1*2**  **rs9923231** | **G** | **A** |
| --- | --- | --- |
| **G**  **0.63** | G/G  HD (40%) | G/A  ID (47%) |
| **A**  **0.37** |  | A/A  LD (13%) |

Each potential VKORC1 genotype has been assigned a predicted drug dosing phenotype based on published data in Caucasian populations that show AA genotype is associated with a low warfarin dose phenotype (LD); GA with an intermediate dose (ID) phenotype; and GG with a high dose (HD) phenotype. LD (yellow) individuals are at increased risk of bleeding event; and HD (orange) are those at increased risk of thrombosis. Warfarin dosing is also dependent on other known genetic (e.g. CYP2C9 and VKORC1) and non-genetic (such as age, gender, body mass index and target INR) factors.

**Table S15. Metabolic phenotypes, frequency and evidence scoring of gene allelic variants for *CYP4F2*-warfarin**

| **Allele** | **Key Variant dbSNP ID** | **Nucleotide Change (cDNA)** | **Mutation Effect** | **Enzyme Activity^b^** | | **Evidence Code**^a^ | **Allele Frequency (#Subjects (%))** | | | | **Key References**  **(PMID#)** |
| --- | --- | --- | --- | --- | --- | --- | --- | --- | --- | --- | --- |
|  |  |  |  | **In Vitro/ In Vivo** | **Metabolic Phenotype** |  | **Caucasian** | **African** | **Asian** | **Reference^c^** |  |
| **CYP4F2*1** | wild type | reference | lacks variants below | normal | normal | 1 | 379  (73%) | 246  (91%) | 288  (79%) | 1000GP | [[79](#_ENREF_79)] |
| **CYP4F2*3** | rs2108622 | 1297G>A | Val433Met, reduced hepatic concentrations, 60% lower activity than wild type | decreased | requires higher warfarin dose | 1 | 379  (27%) | 246  (9%) | 288  (21%) | 1000GP | [[79-85](#_ENREF_79)] |
| **CYP4F2 P13R** | rs2906890 | 38C>G | Pro13Arg | ND | ND | 13 | ND | ND | ND | ND | ND |
| **CYP4F2 W12C** | rs2906891 | 36G>C | Trp12Cys | ND | ND | 13 | ND | ND | ND | ND | ND |
| **CYP4F2 H343H** | rs2074900 | 11602C>T | His343His | ND | ND | 13 | ND | ND | ND | ND | ND |
| **CYP4F2 P55P** | rs3093106 | 165A>G | Pro55Pro | ND | ND | 13 | ND | ND | ND | ND | ND |
| **CYP4F2 G93G** | rs8100960 | 2042A>C | Gly93Gly | ND | ND | 13 | ND | ND | ND | ND | ND |
| **CYP4F2 N112N** | rs8110714 | 2099C>T | Asn112Asn | ND | ND | 13 | ND | ND | ND | ND | ND |
| **CYP4F2 A116A** | rs3093136 | 5034C>G | Ala116Ala | ND | ND | 13 | ND | ND | ND | ND | ND |
| **CYP4F2 G185V** | rs3093153 | 7207G>T | Gly185Val | ND | ND | 13 | 58  (3%) | 59  (2%) | 45  (0%) | HapMap | ND |
| **CYP4F2 L278F** | rs4605294 | 8103C>T | Leu278Phe | ND | ND | 13 | ND | ND | ND | ND | ND |
| **CYP4F2*2** | rs3093105 | 34T>G | Trp12Gly, normal arachidonic acid and leukotriene B4 activity in vitro, untested vitamin K activity | normal | ND | 14 | 23  (11%) | 24  (21%) | 42  (8%)^i^ | [[79](#_ENREF_79)] HapMap^i^ | [[79](#_ENREF_79)] |

ND = no data/not determined, ^a^ **=** evidence code assigned to each allele provides an indication of the strength and type of evidence for a drug-related phenotype and is based on the scheme presented in **Table 1** (green indicates clinically relevant evidence, yellow=potential clinical relevance, light orange= clinical relevance unknown, and dark orange= clinical relevance unsupported). For alleles with multiple types of supporting data, the code for the greatest strength of evidence is assigned, **^b^ =** vitamin K oxidase activity measured in vitro and protein levels measured in human liver microsomes from 12 donors genotyped for CYP4F2 rs2108622 (CC, CT, TT) [[80](#_ENREF_80)]. In vivo studies compared daily or weekly warfarin maintenance dosage between CC, CT and TT groups, **^c^ =** Allele frequency from 1000 Genome Project phase 1, HapMap, or specific reference (i)

**Table S16. Predicted drug metabolizing phenotypes for “included” CYP4F2 genotypes**

| **Allele  (frequency)** | **CYP4F2*1**  **(V433)** | **CYP4F2*2**  **(M433)** |
| --- | --- | --- |
| **CYP4F2*1**  **(V433)**  **0.73** | *1/*1  LD (53%) | *1/*2  ID (40%) |
| **CYP4F2*2**  **(M433)**  **0.27** |  | *2/*2  HD (7%) |

Each CYP4F2 genotype has been assigned a predicted drug interacting phenotype based on published data that suggests a linear additive effect of the T allele (M433 or CYP4F2*2) such that wild type *1/*1 individuals have a lower warfarin dose requirement (LD), *1/*2 individuals have an intermediate requirement (ID) and *2/*2 individuals require a higher warfarin dose (HD). ID phenotype (blue);LD (yellow); and HD (orange). Warfarin dosing is also dependent on other known genetic (e.g. CYP2C9 and VKORC1) and non-genetic (such as age, gender, body mass index and target INR) factors.

**Table S17. FDA CYP2C9 and VKORC1 based therapeutic dosing guidelines (adapted from the Coumadin drug label [**[**86**](#_ENREF_86)**])**

| **VKOCR1** | **CYP2C19** | | | | | |
| --- | --- | --- | --- | --- | --- | --- |
|  | ***1/*1** | ***1/*2** | ***1/*3** | ***2/*2** | ***2/*3** | ***3/*3** |
| **GG** | 5-7mg | 5-7mg | 3-4mg | 3-4mg | 3-4mg | 0.5-2mg |
| **AG** | 5-7mg | 3-4mg | 3-4mg | 3-4mg | 0.5-2mg | 0.5-2mg |
| **AA** | 3-4mg | 3-4mg | 0.5-2mg | 0.5-2mg | 0.5-2mg | 0.5-2mg |

“Range of expected therapeutic warfarin doses based on CYP2C9 and VKORC1 genotypes. Ranges are derived from multiple published clinical studies. Other clinical factors (e.g. age, race, body weight, sex, concomitant medications, and comorbidities) are generally accounted for along with genotype in the ranges expressed in the table. VKORC1 -1639 G -> A (rs9923231) variant is used in this table. Other co-inherited VKORC1 variants may also be important determinants of warfarin dose. Patients with *1/*3, *2/*2, *2/*3 and *3/*3 may require more prolonged time (>2 to 4 weeks) to achieve maximum INR effect for a given dosage regimen.”

**Table** **S18**. Alternative phenotype classification system for *CYP2D6* alleles

1. **b.)**

| CYP2D6 Alleles | Functional allele count |
| --- | --- |
| *3, *4, *5, *6, *7, *14, *21, *36, *40, *42, *44, *56, *4xN | 0 |
| *9, *10, *17, *29,*41,*10xN, *41xN | 0.5 |
| *1, *2 | 1 |
| *1xN, *2xN | 2+ |

| Sum # functional alleles | Metabolic Phenotype Classification |
| --- | --- |
| 0 | PM |
| 0.5-1.0 | IM |
| 1.5-2.0 | EM |
| 2+ | UM |

a.) Phenotype classification based on gene dose method; b.) Translation of allele count method to traditional *CYP2D6* phenotypes. (Adapted from Gaedigk 2008, [[87](#_ENREF_87)]).

**Table** **S19**. Studies of *CYP2D6* Variation and Clinical Outcomes

| **Study** | **N** | **Study**  **Design** | **Patient Population** | **Genotype/ Phenotypes** | **Primary/Secondary Endpoints** | **Results** | **Comments**  **/ Limitations** |
| --- | --- | --- | --- | --- | --- | --- | --- |
| [[88](#_ENREF_88)] | 18 | RCT, double blind, 3 way cross-over design.  Placebo, Morphine (20mg) and Codeine (170mg) given to individuals and implemented a pain tolerance to cold pressor test (CPT). | German volunteers | 9 EM genotypes:  Genotype N  *1/*1: 2  *1/*2: 4  *1/*5: 1  *1/*10: 1  *3/*2: 1  9 PM genotypes:  Genotype N  *4/*4: 6  *4/*5: 2  *3/*5: 1 | Pain tolerance (time) for CPT, before (placebo) and after 170 mg codeine administration, and a comparable dose of 20mg morphine sulfate.  PK: AUC, C_max_ (nmol/l), t1/2 and CI_oral_ compared between EMs and PMs for both codeine and morphine.  ADEs: Multiple symptoms reported, calculated an avg. point score over time by phenotype. | After codeine, EMs had a significantly longer mean time (p<0.01) of pain tolerance than PMs.  PMs had no difference in pain tolerance after codeine compared to placebo.  PK: After codeine, EMs produced had similar amounts of morphine concentration in serum but. PMs had only trace amounts of morphine in serum after codeine dose. Morphine metabolites were greatly diminished in PMs compared to EMs as well.  No significant differences between in C_max_, AUC, t1/2 or CI_oral_ of codeine were found after codeine administration between the two phenotypes.  ADEs: No sig. differences found in adverse events from codeine were found between two groups (p> 0.05). | This study provides *in vivo* PK and clinical outcome evidence for codeine and CYP2D6 PM and EM genotypes.  PK: PMs had sig lower plasma levels of morphine (codeine metabolite) than EMs, but other PK parameters for codeine were not stat. diff.  Clinical: PMs had sig. less pain tolerance than EMs for the Cold Pressor test.  ADEs: Adverse events (side effects) from codeine were found to be the same between EMs and PMs, despite less pain tolerance in PMs. |
| [[89](#_ENREF_89)] | 16 | Open, RCT cross-over design. Dextromethorphan (30mg) and codeine (50mg) MRs assessed for extreme metabolizer phenotypes. | Healthy German Caucasian volunteers;  Out of 515 volunteers, 32 men and 25 women selected by CYP2D6 genotype. | High codeine MR group:  Genotype N  *4/*4: 5  *4/*6: 1  *4/*41: 1  *3/*4: 1  Lowest codeine MR group:  Genotype N  *1/*1 4  2x *1/*1 4 | PK - Codeine metabolism  Examined how 2 genotyping scores and DM phenotyping predicted Codeine metabolism  No PD in subjects was assessed. | For low morphine formation, (PMs, *4 alleles) either genotyping system worked well (88%) in predicting highest codeine MR.  For the UMs, genotyping CYP2D6 (duplication *1allele) missed 50% of low codeine MRs (high morphine formation), 3 of those UM were detected by Dextromethorphan (DM) MR. | PK: Known CYP2D6 PM alleles predict high codeine MR well.  Genotype-Phenotype:  This study shows that UM phenotype is only captured 50% by CYP2D6 duplication alleles (*1xN).  Using both CYP2D6 genotyping and DM MR tests, increased correct assignment of codeine metabolizer phenotype status to 87%. |
| [[90](#_ENREF_90)] | 11 | Pharmakinetic study | End stage renal disease patients (undergoing hemodialysis) from Karolinska University Hospital, Sweden | 11 patients selected from 228 prevalent cases:  EMs  *1/*1 : 5  *1/*4: 4  PMs  *4/*4: 2  Excluded one EM patient due to high morphine metabolite conc. | Mean plasma concentrations of codeine metabolites (C6G, M3G, and M6G). | Although no statistical was calculated, the EMs had a much higher mean plasma concentration of morphine metabolites (M3G and M6G) than either PM in these hemodialysis patients. | Limitations:  Small study, unclear how to interpret results. |
| [[91](#_ENREF_91)] | 54 | Prospective, open-label study receiving care at Univ. of IL Medical Center | Sickle cell anemia patients  (Selected pop)  Excluded:  Those with contraindication to codeine, hx of acute pain crisis in past 2 weeks. Not clear if this would select out some CYP2D6 genotypes. | 54 patients  Allele and genotype frequencies in Blacks with sickle cell disease  N=30 N=24  Genotype Grp I Grp II  *other/*other 21 13  *other/*17 5 2  *other/*29 2 4  *other/*41 1 0  *17/*17 1 4  *29/*29 0 1 | PK: Group I if morphine was measured in plasma or Group II if it was not detected.  Primary outcome: CYP2D6 genotype  Secondary outcome: ER visit or hospital admission | PK: Frequency of  *17 and *29 homozygotes were borderline sig. diff. between groups defined by morphine levels at p=0.07.  Mean Cmax and AUC->6 for morphine levels was 40% and 30% lower in *17 carriers (n=12) compared to non-carriers (n=34). M3G and M6G metabolites Cmax and AUC were sig. lower (P<0.05) in Group II compared to Group I.  Clinical: Mean # ER visits and hospital admissions in 12 mo. before study were higher in Group II than Group I.  ER visits did not diff by group, but hospital admits were sig diff (p<0.05).Grp I: 0.9 +/- 1.4;  GrpII: 2.2 +/- 4.1 | PK: *17 and *29 homozygotes had sig diff lower PK parameter values compared to non-carriers of these alleles.  Clinical: More hospital admissions in no morphine group. |
| [[92](#_ENREF_92)] | 187 | Observational PK study of analgesia efficacy in elective adominal surgical patients | Patients were given tramadol at end of surgery. MRs measured afterwards and assessed need for rescue pain medication. | Genotype Phenotype N  *1/*1 EM 68  *1/*3 IM 2  *1/*4 IM 41  *1/*5 IM 5  *1/*6 IM 1  *1/*10 IM 8  *1/*41 IM 23  *10/*41 IM 2  *3/*41 IM 2  *4/*41 IM 2  *6/*10 IM 1  *6/*41 IM 1  *45/*45 IM 1  *3/*4 PM 1  *4/*4 PM 14  *1/*4xN IM 1  *1/*41xN IM 2  *1/*1xN UM 8 | PK: Tramadol/ ODT concentrations measured over time  Clinical assessment:  Measured by need for rescue post-operative analgesia. Patients were classified as  non-responders if they needed rescue analgesia during 48-hr recovery period and/or gave a negative rating for pain management. | PK: UMs, EMs and IMs had significantly higher levels of ODT at 90 and 180 min. after tramadol administration than UMs (p<0.001).  AUC curves of tramadol separated the PMs from other genotypes but not the higher functioning genotypes from each other. | Clinical:  PMs needed rescue medication more frequently than other genotypes (p<0.001). More PMs were classified as non-responders compared to active CYP2D6 genotypes (p<0.001).  Limitations:  Study measures metabolic capacity of tramadol (opiod related), not codeine.  But it does examine some of rare duplication (null) alleles. |
| [[93](#_ENREF_93)] | 73 | Case-control study of children with sickle cell anemia unresponsive to codeine | Children (4-18 yrs)with sickle cell anemia, on codeine, visiting ER for pain crisis, in Milwakee, Wisconsin | Disease severity ≤1.5 AS(%)  Mild (n=36) 44%  Severe (n=37) 68%  Severe taking  Hydroxyurea(n=27) 78%  Alleles ≤1.5 AS: *4, *5, *6, *10, *17, *40 | Clinical:  Comparing children on hydrourea(severe disease) vs. mild disease by CYP2D6 functional activity score (AS≤1.5) | Disease Severity OR (95%CI)  Mild 1.0 (ref)  Severe 2.3 (1.0-6.7)  Severe on HU 4.4 (1.4-13.4)  P<0.05 | Children were 5 times more likely to have an ER visit (failing pain relief outpatient codeine) if they had CYP2D6 allele score ≤1.5 (IM or PMs).  Small sample size, no adj. for confounders. |
| [[94](#_ENREF_94)] | 181 | PD study  IMI breaks down by CYP2C19 into DESI which is metabolized by CYP2D6. | Psychiatric patients taking Imipramine (IMI) anti-depressant , aged 18-65 yrs with MDD in Netherlands. | 181 psychiatric patients:  CYP2D6 allele freq:  *1: 37%  *2: 22%  *3: 2%  *4: 18%  *5: 4%  *6: 1%  *9: 3%  *10: 2%  *41: 10%  Duplication: 8%  (doesn’t specify allele) | Phenotype based on allele-specific graded:  SGD= 1 for each fully functional allele (*1,*2)  SGD= 0 for each null allele (*3,*4,*5, *6)  SGD= 0.5 for each decreased activity allele (*9, *10, *41) | The SGD groups showed a significant difference in IMI and DESI plasma concentration per dose unit. (p<0.0001). A trend of increasing levels of IMI dose was required to achieve steady state IMI levels with increasing SGD alleles. | Drug dose requirement for each SGD to achieve steady state target of IMI compared to SGD = 2 (100%):  #alleles: % dose required  0: 40%  0.5: 48%  1: 67%  1.5: 75%  > 2 : 156% |
| [[95](#_ENREF_95)] | 26 | Longitudinal PK study to assess the CYP2D6 allele-specific metabolic capacity of codeine. | Healthy male Caucasian volunteers from hospital in Germany | Grouped by allele-specific from genotype (ASG):  Score Genotype N  0 *3/*3 1  *4/*4 2  1.5 *1/*9 1  *1/*10 1  *2/*41 1  *35/*41 1  2.0 *1/*1 4  *1/*2 3  *1/*35 1  2.5 *1x2/9 1  *1x2/*10 1  *1x2/*41 1  3.0 *35x2/*1 1  *2x2/*35 2  *1x2/*35 1  *2x2/*1 2  *1x2/*1 1 | A trend test was applied to the CI/weight and AUC (morphine)  parameters to see if the allele-specific grade phenotype would significantly predict metabolic capacity of codeine.  ASG conversion to traditional phenotyping:  PMs= 0  EMs= 1.5 to 2  UMs > 2 | PK:  ASG CI/wt AUC  0 1.7 0.5  1.5 1.7 8.4  2.0 2.0 12.0  2.5 1.8 16.0  3.0 2.4 16.0  Trend: p=0.01 p<0.001  A significant trend was found between allele specific score and the metabolism of codeine into morphine. The morphine AUCs differed over 30-fold between PMs and UMs.  PD: Little evidence of any differences between CYP2D6 genotypes on codeine’s effect on pupil diameter. | PK: A sig. diff. was found between UM and EM groups for the AUC of morphine and morphine metabolite levels (M3G, M6G).  UM codeine metabolism was 1.5x faster in those with gene duplication compared to EMs.  ADEs:  91% of UMs reported sedation compared to 50% EMs (p=0.03).  Allele specific scoring is more precise way to predict metabolism phenotypes. |

MR: metabolic ratio, ADE: adverse drug event, PK: pharmacokinetic, sig: significant; diff: differences; funct= functional; DR: dose-response; AS: allele activity score; KW=Kruskal-Wallis test; OR: odds ratio, CI: confidence interval, EM: Extensive metabolizer; PM: Poor metabolizer; IM: Intermediate metabolizer; UM: Ultra-rapid metabolizer.

**Table S20.** **Metabolic phenotypes, frequency and evidence scoring of gene allelic variants for *CYP2D6*-codeine**

| **Allele** | **Key Variant**  **dbSNP ID** | **Nucleotide Change (cDNA)** | **Mutation Effect^b^** | **Enzyme Activity** | **Evidence Code^a^** | **Allele Frequency (# Subjects (%))** | | | | **Key References**  **(PMID#)** |
| --- | --- | --- | --- | --- | --- | --- | --- | --- | --- | --- |
|  |  |  |  |  |  | **Caucasian** | **African** | **Asian** | **Reference^c^** |  |
| **CYP2D6*1** | wild type | reference | lacks variants below | normal | 1 | 2449  (37%)^d^ | 554 (31%)^d^ | 758 (32%)^d^ | [[96](#_ENREF_96), [97](#_ENREF_97)] | reference in studies below |
| **CYP2D6*2** | rs16947  rs1135840 | 2850C>T 4180G>C | Arg296Cys, Ser486Thr and lacks variants below^e^, greater pain tolerance with codeine than PMs in clinical study (n=18) | normal | 1 | 314  (29%)^d^ | 254  (33%)^d^ | 482  (16%)^d^ | [[98](#_ENREF_98)] | [[88](#_ENREF_88), [99](#_ENREF_99)] |
| **CYP2D6*3** | rs35742686 | 2549delA | Arg259Gly, frameshift, no activity, less pain tolerance with codeine than EMs in clinical study (n=18) | none | 1 | 2449 (2%) | 601 (<1%) | 913 (<1%) | [[96](#_ENREF_96), [100](#_ENREF_100)] | [[88](#_ENREF_88)] |
| **CYP2D6*4** | rs3892097 | 1846G>A | splice defect [[12](#_ENREF_12)], Pro34Ser, no activity, associated with no morphine production from codeine, less pain tolerance with codeine than EMs in clinical study (n=18) | none | 1 | 2449  (20%) | 651 (7%) | 1889 (8%) | [[96](#_ENREF_96), [100](#_ENREF_100)] | [[88](#_ENREF_88)] |
| **CYP2D6*5** | N/A | gene deletion | deletion of entire CYP2D6 gene, no in vivo protein, no activity, less pain tolerance with codeine than EMs in clinical study (n=18) | none | 1 | 2449 (3%) | 651 (7%) | 1644 (8%) | [[96](#_ENREF_96), [100](#_ENREF_100)] | [[88](#_ENREF_88), [101](#_ENREF_101)] |
| **CYP2D6*29** | rs59421388 | 1012C>T | Val338Met, low protein expression, low debrisoquine activity, less pain relief from codeine in sickle cell anemia patients | decreased | 1 | 672 (<1%) | 251 (7%) | 360 (<1%) | [[100](#_ENREF_100)] | [[91](#_ENREF_91), [102](#_ENREF_102), [103](#_ENREF_103)] |
| **CYP2D6*2xN** | N/A^f^ | *2 duplication | *2 duplication, N active genes, infant overdose death case report from transmission of high morphine levels from UM mother, CNS depression in breast-fed infants of 3 UM mothers in case-control study (n=72) | increased | 1 | 2449  (<1%) | 554 (<1%) | 790 (4%) | [[96](#_ENREF_96), [100](#_ENREF_100)] | [[104-106](#_ENREF_104)] |
| **CYP2D6*1xN** | N/A | *1 duplication | *1 duplication, N active genes, seen in morphine UM | increased | 2 | 833  (<1%) | 251  (1%) | 603 (4%) | [[98](#_ENREF_98), [100](#_ENREF_100)] | [[106](#_ENREF_106)] |
| **CYP2D6*4xN** | N/A^f^ | *4 duplication | no functional protein created in an in vivo tramadol PK study, debrisoquine metabolic ratio similar to PM | none | 4n | 833  (<1%) | 127  (4%) | 603 (<1%) | [[98](#_ENREF_98), [100](#_ENREF_100)] | [[107](#_ENREF_107), [108](#_ENREF_108)] |
| **CYP2D6*6** | rs5030655 | 1707delT | 118freameshift, premature stop codon, no activity, seen in CYP2D6 PM | none | 5n | 2449  (1%) | 554 (<1%) | 630 (1%) | [[96](#_ENREF_96), [100](#_ENREF_100)] | [[109-111](#_ENREF_109)] |
| **CYP2D6*7** | rs5030867 | 2935A>C | His324Pro, seen in sparteine PM, inactive in silico, reduced-activity in vitro debrisoquine, dextromethorphan, bufuralol | estimated none | 5n | 97  (2%) | 554  (<1%) | 89 (<1%) | [[96](#_ENREF_96), [111](#_ENREF_111)] |  |
| **CYP2D6*10** | rs1065852 (in combination with WT allele for rs3892097) | 100C>T (Absence of 1846G>A) | Pro34Ser, low activity, seen in Japanese sparteine PMs, dextromethorphan metabolic ratio significantly higher than EMs in Chinese study (n=131) | decreased | 5ae | 1320  (5%) | 405  (6%) | *10:  98 (9)^g^,  tandem  *10-*36:  98 (33) | [[100](#_ENREF_100), [112](#_ENREF_112)] | [[99](#_ENREF_99), [113-118](#_ENREF_113)] |
| **CYP2D6*14** | rs5030865^h^ | 1758G>A | Gly169Arg, splice defect, no activity, dextromethorphan metabolic ratio significantly higher than EMs or *10 carriers in Chinese study (n=131), inactive in vitro dextromethorphan, bufuralol, in vivo inactive debrisoquine | none | 5ae | 672 (0%) | 251 (0%) | 758 (<1%) | [[97](#_ENREF_97), [119](#_ENREF_119), [120](#_ENREF_120)] | [[116-119](#_ENREF_116), [121](#_ENREF_121)] |
| **CYP2D6*21** | rs72549352 | 2573insC | **267frameshift**, premature stop codon, in vivo inactive debrisoquine | none | 5se | ND | ND | 758 (<1%) | [[97](#_ENREF_97)] | [[116](#_ENREF_116), [122-125](#_ENREF_122)] |
| **CYP2D6*41** | rs28371725 | 2988G>A | splice defect, promoter variant, low protein expression in human liver | decreased | 5se | 2449  (9%) | 554  (2%) | 758 (2%) | [[96](#_ENREF_96), [97](#_ENREF_97)] | [[114-116](#_ENREF_114), [126](#_ENREF_126), [127](#_ENREF_127)] |
| **CYP2D6*10xN** | N/A^f^ | *10 duplication | seen in tandem duplication with CYP2D6*36, reduced dextromethorphan and morphine activity, increased metabolic ratio | decreased | 5dp | 157  (<1%) | 843 (<1%) | 863 (<1%) | [[100](#_ENREF_100)] | [[97](#_ENREF_97), [98](#_ENREF_98), [100](#_ENREF_100), [112](#_ENREF_112)] |
| **CYP2D6*9** | rs72549350  (or rs5030656) | 2615_2617 delAAG | Lys281frameshift, premature stop codon, low protein expression, graded allele-specific activity significantly correlated with serum imipramine levels | decreased | 6ae | 2449  (3%) | 554 (<1%) | 603 (<1%) | [[96](#_ENREF_96), [100](#_ENREF_100)] | [[94](#_ENREF_94), [128](#_ENREF_128)] |
| **CYP2D6*17** | rs28371706 | 1023C>T | Thr107Ile, seen in African debrisoquine PM, in vitro expression 20% of wild type | decreased | 6ae | 2449  (<1%) | 651 (21%) | 790 (2%) | [[96](#_ENREF_96), [100](#_ENREF_100)] | [[120](#_ENREF_120), [129](#_ENREF_129), [130](#_ENREF_130)] |
| **CYP2D6*36  (or *10C)** | **Gene conversion plus** ,  rs1065852 (in combination with WT allele for rs3892097) | CYP2D7 gene conversion, 100C>T | **¡** CYP2D7 conversion in exon 9, P34S, inactive in vitro, seen in debrisoquine PM, seen in duplication and in tandem with *10, in vivo inactive for venlafaxine and dextromethorphan | none | 6n | 418  (0%) | 814  (<1) | *36:  76 (<1)^g^  tandem  *10-*36:  98 (33) | [[112](#_ENREF_112), [131](#_ENREF_131)] | [[117](#_ENREF_117), [131-134](#_ENREF_131)] |
| **CYP2D6*40** | rs72549356 | 1863ins18 | 18 base pair insertion, no activity, seen in dextromethorphan PM, in vivo inactive for dx | none | 6n | 408  (0%) | 251 (<1%) | ND | [[120](#_ENREF_120)] | [[120](#_ENREF_120), [135](#_ENREF_135)] |
| **CYP2D6*42** | rs72549346 | 3259insGT | 365frameshift, no activity, premature stop codon, seen in dextromethorphan PM, in vivo inactive for dx | none | 6n | 203  (0%) | 193  (<1%) | 758 (0%) | [[97](#_ENREF_97), [136](#_ENREF_136)] | [[135](#_ENREF_135), [136](#_ENREF_136)] |
| **CYP2D6*44** | rs72549349 | 2950G>C | splice defect, no activity, seen in a Japanese dextromethorphan PM, in vivo inactive for dx | none | 6n | ND | ND | 286 (0%) | [[137](#_ENREF_137)] | [[123](#_ENREF_123)] |
| **CYP2D6*56** | rs72549347 | 3201C>T | Arg344X, premature stop codon, no activity, seen in human hepatocytes with no CYP2D6 activity, inactive in vitro and in vivo for dextromethorphan | none | 6n | 339 (0%) | 422 (<1%) | 39  (0%) | [[138](#_ENREF_138)] | [[124](#_ENREF_124), [138](#_ENREF_138)] |
| **CYP2D6*41xN** | N/A^f^ | *41 duplication | low tramadol activity in surgical patients when with wild type allele | decreased | 6dp | 250 (<1%) | 127  (0%) | 241  (<1%) | [[98](#_ENREF_98), [100](#_ENREF_100)] | [[107](#_ENREF_107)] |
| **CYP2D6*18** | rs1135836 | 4125_4133 dupGTGCCCACT | **468_470dupVPT**, reduced-activity in vitro seen in Japanese sparteine PMs | decreased | 10 | 408  (0%) | 251  (0%) | 758 (<1) | [[97](#_ENREF_97), [120](#_ENREF_120)] | [[117](#_ENREF_117), [139](#_ENREF_139)] |
| **CYP2D6*12** | rs5030862 | 124G>A | Gly42Arg, seen in Caucasian sparteine PM | none | 11 | 672 (<1%) | 251 (0%) | 162 (0%) | [[119](#_ENREF_119), [120](#_ENREF_120), [140](#_ENREF_140)] | [[134](#_ENREF_134), [141](#_ENREF_141)] |
| **CYP2D6*15** | rs72549357 | 137_138 insT | **L46frameshift**, premature stop codon, T insertion in exon 1 | none | 11 | 408  (0%) | 251 (0%) | ND | [[120](#_ENREF_120), [135](#_ENREF_135)] | [[142](#_ENREF_142)] |
| **CYP2D6*8** | rs5030865^h^ | 1758G>T | G169X, non-functional protein, splice defect, premature stop codon, seen in subject with CYP2D6 deficiency | none | 12 | 408  (0%) | 251 (0%) | ND | [[120](#_ENREF_120)] | [[143](#_ENREF_143)] |
| **CYP2D6*11** | rs5030863 | 883G>C | non-functional protein, splice defect, premature stop codon, seen in CYP2D6 PM | none | 12 | 101  (<1%) | 251 (0%) | 162 (0%) | [[120](#_ENREF_120), [140](#_ENREF_140), [144](#_ENREF_144)] | [[144](#_ENREF_144)] |
| **CYP2D6*19** | rs72549353 | 2539_2542 delAACT | **225frameshift**, ↓transcription | none | 12 | 672  (<1%) | ND | ND | [[119](#_ENREF_119)] | [[119](#_ENREF_119), [145](#_ENREF_145)] |
| **CYP2D6*20** | rs72549354 | 1973_1974 insG | **212frameshift**, premature stop, no protein expression | none | 12 | ND | ND | ND |  | [[146](#_ENREF_146)] |
| **CYP2D6*38** | rs72549351 | 2587_2590 del/AGTC | 271frameshift, seen in debrisoquine PM | none | 12 | ND | ND | ND |  | [[147](#_ENREF_147)] |
| **CYP2D6*65** | rs1065852 rs1058164 rs16947 rs1135840 |  | variants not expected to alter protein function | ND | 13 | 694  (0%) | 544  (0%) | ND | [[148](#_ENREF_148)] | [[148](#_ENREF_148)] |
| **CYP2D6*39** | rs1058164 rs1135840 |  | normal in vitro bufuralol and dextromethorphan activity | normal | 14 | ND | ND | 800  (<1%) | [[117](#_ENREF_117)] | [[117](#_ENREF_117)] |

ND = no data/no determined, N/A = not applicable, EM = extensive metabolizer, PM = poor metabolizer, UM = ultra-rapid metabolizer,

^a^ **=** evidence code assigned to each allele provides an indication of the strength and type of evidence for a drug-related phenotype and is based on the scheme presented in **Table 1** (green indicates clinically relevant evidence, yellow=potential clinical relevance, light orange= clinical relevance unknown, and dark orange= clinical relevance unsupported). For alleles with multiple types of supporting data, the code for the greatest strength of evidence is assigned,

^b^ = In general *in vitro* substrate kinetics measured in recombinant protein expressed in bacterial, yeast, or mammalian cDNA expression systems with dextromethorphan (antitussive drug), sparteine (antiarrhythmic), debrisoquine (antihypertensive), or bufuralol (experimental beta blocker) as probe substrates for CYP2D6 activity. Clinical PK/PD evidence is based on *in vivo* codeine metabolism, association between CYP2D6 genotypes and heart rate, blood pressure, and adverse drug reactions, such as bradycardia or morphine toxicity.

**^c^ =** Allele frequency from specific reference as indicated

^d^ = Note that CYP2D6*1 and CYP2D6*2 are not genotyped directly (are not sequence analysis based) but rather are default assignments based on the absence of other variants genotyped directly in the referenced studies. The reported CYP2D6*1 and *2 frequencies are likely to be overestimations of the true frequencies. For the purpose of diplotype and metabolizer type frequency estimation (Table S21 and Table 9) the joint frequency of CYP2D6*1 plus CYP2D6*2 is estimated as 100% minus (allele frequencies of other variants reported in this table with an evidence code ≤ 7): Caucasians=55%; African Ancestry=45%; and East Asians Ancestry=29%.

^e^ = CYP2D6*2 harbors multiple variants with wild type activity that also occur with altered-function variants on other CYP2D6 haplotypes.

^f^ = gene multiplication, also harbors variants (rs#) associated with the respective *allele

^g^ = *10 or *36 allele frequency as a single variant in a diploid genotype, distinguished from the frequency given by either allele in tandem with another allele or in duplication,

^h^ = rs5030865 is tri-allelic (1758G>A/T), with the A allele giving a splice defect (Gly169Arg), and the T allele giving a non-synonymous change (Gly169X).

**Table S21. Predicted drug metabolizing phenotypes for “included” CYP2D6 genotypes**

| **Allele (frequency)** | **CYP2D6*1 (+*2)** | **CYP2D6*4** | **CYP2D6*41** | **CYP2D6*5** | **CYP2D6*9** | **CYP2D6*10** | **CYP2D6*7** | **CYP2D6*6** | **CYP2D6*3** | **CYP2D6*17** | **CYP2D6**  ***29** | **CYP2D6*14,*21, *36, *10C, *40, *42, *44, or *56: (=**X))*** | **CYP2D6*1xN or CYP2D6*2xN** | **CYP2D6*4xN** | **CYP2D6*10xN or *41xN** |
| --- | --- | --- | --- | --- | --- | --- | --- | --- | --- | --- | --- | --- | --- | --- | --- |
| **CYP2D6*1**  **(+CYP2D6*2)**  **(55%)** | *1/*1 EM (30%) | *1/*4  EM (22%) | *1/*41  EM (10%) | *1/*5  EM (3%) | *1/*9  EM (3%) | *1/*10  EM (5.5%) | *1/*7  EM (2%) | *1/*6  EM (1%) | *1/*3  EM (2.2%) | *1/*17  EM (0.01%) | *1/*29  EM (0.1%) | *1/**X*  EM(0.1%) | *1/**1N or*2N*  UM (0.1%) | *1/*44  EM (0.1%) | *1/*10N or*41N  EM (0.1%) |
| **CYP2D6*4**  **(20%)** |  | *4/*4  PM (4%) | *4/*41  IM (3.6%) | *4/*5  PM (1.2%) | *4/*9  IM (1.2%) | *4/*10  IM (2%) | *4/*7  PM (0.8%) | *4/*6  PM (0.4%) | *4/*3  PM (0.8%) | *4/*17  IM (<0.1%) | *4/*29  IM (<0.1%) | *4/**X*  PM(<0.1%) | *4/**1N or*2N*  UM (<0.1%) | *4/*4N  PM (<0.1%) | *4/*10Nor*41N  IM (<0.1%) |
| **CYP2D6*41**  **(9%)** |  |  | *41/*41  IM (0.8%) | *5/*41  IM (0.5%) | *41/*9  IM (0.5%) | *41/*10  IM (0.9%) | *41/*7  IM (0.4%) | *41/*6  IM (0.2%) | *41/*3  IM (0.4%) | *41/*17  IM (<0.1%) | *41/*29  IM (<0.1%) | *41/**X*  IM(<0.1%) | *41/**1N or*2N*  UM (<0.1%) | *41/*4N  IM (<0.1%) | *41/*10N or*41N  IM (<0.1%) |
| **CYP2D6*5**  **(3%)** |  |  |  | *5/*5  PM (0.1%) | *5/*9  IM (0.2%) | *5/*10  IM (0.3%) | *5/*7  PM (0.12%) | *5/*6  PM (0.06%) | *5/*3  PM (0.1%) | *5/*17  IM (<0.1%) | *5/*29  IM (<0.1%) | *5/**X*  PM(<0.1%) | *5/**1N or*2N*  UM (<0.1%) | *5/*4N  PM (<0.1%) | *5/*10N or*41N  IM (<0.1%) |
| **CYP2D6*9**  **(3%)** |  |  |  |  | *9/*9  IM (0.1%) | *9/*10  IM (0.3%) | *9/*7  IM (0.1%) | *9/*6  IM (0.06%) | *9/*3  IM (0.1%) | *9/*17  IM (<0.1%) | *9/*29  IM (<0.1%) | *9/**X*  IM(<0.1%) | *9/**1N or*2N*  UM (<0.1%) | *9/*4N  IM (<0.1%) | *9/*10N or*41N  IM (<0.1%) |
| **CYP2D6*10**  **(5%)** |  |  |  |  |  | *10/*10  IM (0.3%) | *10/*7  IM (0.2%) | *10/*6  IM (0.1%) | *10/*3  IM (0.2%) | *10/*17  IM (<0.1%) | *10/*29  IM (<0.1%) | *10/**X*  IM(<0.1%) | *10/**1N or*2N* UM (<0.1%) | *10/*4N  IM (<0.1%) | *10/*10N or*41N IM(<0.1%) |
| **CYP2D6*7**  **(2%)** |  |  |  |  |  |  | *7/*7  PM (<0.1%) | *7/*6  PM (<0.1%) | *7/*3  PM (0.1%) | *7/*17  IM (<0.1%) | *7/*29  IM (<0.1%) | *7/**X*  PM(<0.1%) | *7/**1N or*2N* UM (<0.1%) | *7/*4N  PM (<0.1%) | *7/*10N or*41N  IM(<0.1%) |
| **CYP2D6*6**  **(1%)** |  |  |  |  |  |  |  | *6/*6  PM (0.01%) | *6/*3  PM (<0.1%) | *6/*17  IM (<0.1%) | *6/*29  IM (<0.1%) | *6/**X*  PM(<0.1%) | *6/**1N or*2N*  UM (<0.1%) | *6/*4N  PM (<0.1%) | *6/*10N or*41N  IM(<0.1%) |
| **CYP2D6*3**  **(2%)** |  |  |  |  |  |  |  |  | *3/*3  PM (<0.1%) | *3/*17  IM (<0.1%) | *3/*29  IM (<0.1%) | *3/**X*  PM(<0.1%) | *3/**1N or*2N*  UM (<0.1%) | *3/*4N  PM (<0.1%) | *3/*10N or*41N  IM(<0.1%) |
| **CYP2D6*17**  **(<0.1%)** |  |  |  |  |  |  |  |  |  | *17/*17  IM (<0.1%) | *17/*29  IM (<0.1%) | *17/**X*  IM(<0.1%) | *17/**1N or*2N*  UM (<0.1%) | *17/*4N  IM (<0.1%) | *17/*10N or*41N  IM(<0.1%) |
| **CYP2D6*29**  **(<0.1%)** |  |  |  |  |  |  |  |  |  |  | *29/*29  IM (<0.1%) | *29/**X*  IM(<0.1%) | *29/**1N or*2N*  UM (<0.1%) | *29/*4N  IM (<0.1%) | *29/*10N or*41N  IM(<0.1%) |
| **CYP2D6*14,*21, *36, *10C, *40, *42, *44, or *56: (=*X)) (<0.1%)** |  |  |  |  |  |  |  |  |  |  |  | **X* /**X*  PM(<0.1%) | **X* /**1N or*2N*  UM (<0.1%) | **X* /*4N  PM (<0.1%) | *10N or*41N /**X*  IM(<0.1%) |
| **CYP2D6*1xN or CYP2D6*2xN**  **(<0.1%)** |  |  |  |  |  |  |  |  |  |  |  |  | **1Nor*2N* /**1N or*2N*  *UM (<0.1%)* | *4N/**1N or*2N*  UM (<0.1%) | *10N or*41N/ **1N or*2N* UM(<0.1%) |
| **CYP2D6*4xN**  **(<0.1%)** |  |  |  |  |  |  |  |  |  |  |  |  |  | *42/*4N  PM (<0.1%) | *10N or*41N /*4N  IM(<0.1%) |
| **CYP2D6*10xN or *41xN**  **(<0.1%)** |  |  |  |  |  |  |  |  |  |  |  |  |  |  | *10N or*41N /*10N or*41N  IM(<0.1%) |

Approximate expected diplotype frequencies have been estimated based upon reported allele frequencies for Caucasians and Hardy Weinberg principals. Alleles of similar effect and or similar frequency have been combined for brevity. ^a^ - a proportion of *10 alleles will have *36 in tandem and this will affect the metabolizer status of the diplotype (PM instead of IM). Each potential CYP2D6 diplotype has been assigned a predicted drug metabolizing phenotype based on the rule that the highest functioning CYP2D6 allele predicts the phenotypic activity such that individuals with two null alleles are poor metabolizers (PM); those with a normal and a null activity allele are classified as extensive metabolizers (EM); two low activity or one low and one null activity are referred to as intermediate metabolizers (IM); multiplication of a normal activity allele (e.g. *1, *2) to greater than two active copies of the gene leads to ultra-rapid metabolizer (UM) status; multiplication of a null or low activity genes does not appear to alter the drug metabolizing phenotype. Color is an indicator of expected phenotype: blue (EM): normal morphine formation and normal analgesic response to codeine; yellow (IM): reduced morphine formation and possible reduced analgesic response; orange (PM): little morphine formation and likely lack of analgesic response; and purple (UM): increased morphine formation and increased risk of toxicity and ADR

**Table S22 – Example of studies of TPMT Variation and Clinical Outcomes in Thiopurine Treated Patients**

| **Reference** | **Study Design, Disease, ethnicity, No., drug** | **Measured Geno-/ Phenotype** | **Primary / Secondary Endpoints** | **No. of Events Observed** | **Primary / Secondary Results** |
| --- | --- | --- | --- | --- | --- |
| [[149](#_ENREF_149)] | Prospective, rheumatic disease, primarily Cauc. (Scot), N=67 (49 RA, 7 SLE, 11 other), AZA | **TPMT genotype:** *1, *2, *3A, *3B, *3C | ***Primary -*** *ADR –* discontinuation of therapy due to toxicity. | 25 (37%) ADR | **TPMT genotype and ADR:** 5/6 het. patients discontinued therapy had sig. shorter therapy (median, 2 wks [range, 2-4 wks]) compares with WT TPMT patients (median, 39 wks [range 6 – 180 wks]) (P=0.018). |
| [[150](#_ENREF_150)] | Retrospective, IBD, Primarily Cauc, N=106 (84 CD, 22 UC), AZA | **TPMT genotype:** *1, *3A, *3C - assayed in 40 patients (10 intermediate & 30 high TPMT activity – there were no low activity patients)  **TPMT enzyme activity** in RBC | ***CR*** (complete remission: complete withdrawal of steroid therapy and ⁄ or sustained closure of fistula; partial remission: continued steroid dependence, but at reduced daily dose)  ***ADR*** (gastrointestinal upset, flu-like illness, Neutropenia, Hepatotixicity, pancreatitis - severe enough for AZA withdrawal) | 19 (18%) ADR  57 (63%) of 91 patients on AZA for ≥3 months had complete remission, additional 15 partial remission. | **TPMT enzyme activity:** sig. association between AZA toxicity & intermediate TPMT activity (OR, 5.4; 95% CI, 1.5–19.8; P =0.0082). **I**nverse relationship between TPMT activity and complete remission (OR, 0.21; 95% CI, 0.07–0.68; P=0.007).  **TPMT gene- phenotype correlation:** All 10 intermediate activity patients had het. genotype and all 30 high activity patients had *1/*1 genotype. |
| [[151](#_ENREF_151)] | Retrospective, kidney transplant, 22 Cauc; 14 AA, N=36, AZA | **TPMT genotype:** *1, *2, *3A, *3B, *3C  **Hematological indices** | ***ADR***: leucopenia, thrombocytopenia and abnormal hematological profile over 30 day period of AZA therapy. | Leucopenia=0  Thrombocytopenia=1 | **TPMT genotype and ADR:** Those with TPMT het. genotype (5/36) had sig. change in RBC (P=0.021), hematocrit (P=0.006) and hemoglobin (P=0.008) compared with TPMT WT genotype (31/36).  No cases of leucopenia, 1 case of thrombocytopenia in TPMT het. |
| [[152](#_ENREF_152)] | Retrospective for 50 prospective for 50 pediatric ALL patients, Cauc (Slavic), N=100 ALL. N=100 healthy controls, 6-MP | **TPMT genotype:** *1, *2, *3A, *3B, *3C | ***ADR***: hematologic toxicity assessed as ability to tolerate 6-MP therapy | Not provided – measured as weeks of missed therapy expressed as mean of genotype groups | **TPMT genotype distribution in ALL patients**: 90 (90%) *1/*1; 10% het. (9: *1/*3A; 1: *1/*2); 1 hom. *3A/*3B  **TPMT genotype and ADR** (in 50 retrospective patients): Severe leucopenia in *3A/*3B hom.; and greater risk of leucopenia in het. vs. WT hom. (P<0.01). Mean duration of missed therapy 11.3 wks in TPMT hets. vs. 3.4 wks in *1/*1 patients (P<0.01)  **50 prospective ALL**: 4 TPMT het. patients identified and treatment protocol modified (reduction by 25%-50% of standard dose with escalation to full dosage tolerated. None developed neutropenia and all completed therapy. |
| [[153](#_ENREF_153)] | Prospective, IBD, Cauc., N=215 recruited with 207 completing study (117 CD, 90 UC), AZA | **TPMT genotype:** *1, *3A, *3B, *3C  **TPMT enzyme activity** in RBC & **TGN level** in blood – both measured at baseline, 2, 4, 12 and 24 wks | ***CR****:* no steroid use or complete withdrawal by 12 wks and maintenance of response for at least 3 mths (treatment failure defined as surgery and /or other therapy use).  ***ADR*:** gastrointestinal upset, flu-like illness, rash, Hepatitis, myelotoxicity, pancreatitis . | 83/215 (39%) ADR  79/207 (38%) treatment failure | **TPMT genotype and ADR:** 15/19 TPMT het. had ADR to AZA vs. 66/188 WT TPMT (P=0.0003)  **TPMT phenotype and Clinical Response:** 43% response (24/56) with RBC levels >35 pmol⁄h⁄mg⁄ Hb vs. 81% (55/68) response below this level (P<0.001)  **TGN levels** **and Clinical Response:** 74% response (59/80) with levels >100 pmol⁄8 × 10^8^ vs. 46% (20/44) below this level (P=0.0017) |
| [[154](#_ENREF_154)] | Retrospective, kidney transplant, Asian (Thai), N=139, AZA | **TPMT genotype:** *1, *3C  **TPMT enzyme activity** in RBC | ***ADR*:** myelosuppresion (severe leucopenia, thrombocytopenia, or neutropenia) within 6 mths of AZA therapy. | 22 ADR | **TPMT gene- phenotype correlation:** 9/139 (6.5%) had *1/*3C genotype and had sig. lower TPMT activity that 130 TPMT*1/*1 patients (P<0.001).  **TPMT genotype and ADR:** *1/*3C patients were at sig. higher risk of AZA induced myelosuppression (6/9) than WT patients (16/130) (adjusted OR, 14.18 [95% CI, 3.07-65.40]; P < 0.005).  Sensitivity and specificity of TPMT*1/*3C genotype to predict ADR was 27% and 97% respectively, with positive- and negative predictive values of 50% and 95% respectively. |

**Table S23.** **Metabolic phenotypes, frequency and evidence scoring of gene allelic variants for *TPMT*-thiopurines**

| **Allele** | **Key Variant**  **dbSNP ID** | **Nucleotide Change (cDNA)** | **Mutation Effect** | **In Vitro/ In Vivo Enzyme Activity ^b^** | **Evidence Code**^a^ | **Allele Frequency (# Subjects (%))** | | | | **Key References**  **(PMID#)** |
| --- | --- | --- | --- | --- | --- | --- | --- | --- | --- | --- |
|  |  |  |  |  |  | **Caucasian** | **African** | **Asian** | **Reference^c^** |  |
| **TPMT*1** | wild type | reference sequence | Lacks all mutations described below | normal | 1 | 282  (97%) | 248  (90%) | 192  (98%) | Estimated**^c^** | reference in all studies below |
| **TPMT*2** | rs1800462 | 238G>C | Ala80Pro, low protein levels, no activity | very low | 1 | 379  (0.4%) | 246  (0.4%) | 286  (<1%) | 1000GP | [[155](#_ENREF_155), [156](#_ENREF_156)] |
| **TPMT*3A** | rs1800460^d^ rs1142345^d^ | 460G>A 719A>G | low protein levels, no activity,  accelerated degradation | very low | 1 | 379  (2.5%) | 246  (1%) | 286  (<1%) | 1000GP | [[157](#_ENREF_157)] |
| **TPMT*3B** | rs1800460^d^ | 460G>A | Ala154Thr, low protein levels, no activity, accelerated degradation, associated with severe leucopenia in 6-MP-treated patient (*3A/*3B) | very low | 1 | 379  (<1%) | 246  (<1%) | 286  (<1%) | 1000GP | [[152](#_ENREF_152), [158](#_ENREF_158)] |
| **TPMT*3C** | rs1142345^d^ | 719A>G | Tyr240Cys, decreased activity, low protein levels, accelerated degradation | very low | 1 | 379  (0.1%) | 246  (9%) | 286 (2%) | 1000GP | [[157](#_ENREF_157)] |
| **TPMT*4** | ¡rs180058 | Gint9(–1)A | Splice defect, very low activity in Caucasian carriers | very low | 2 | 147^f^  (<1%) | 41  (0%) | 192  (0%) | [[159](#_ENREF_159), [160](#_ENREF_160)] | [[161](#_ENREF_161)] |
| **TPMT**  ***(GCC)5/7** | ¡no rsID | (GCC)6> (GCC)5 or (GCC)7 | trinucleotide repeat in promoter, seen in 2 Caucasians with high TMPT activity, significantly higher in vitro expression in constructs with (GCC)5 and (GCC)7 compared to (GCC)6 | high | 11 | 200 (1%) | ND | ND | [[162](#_ENREF_162)] | [[162](#_ENREF_162)] |
| **TPMT*3D^g^** | rs72552739  rs1800460  rs1142345 | 292G>T 460G>A  719A>G | Glu98Stop plus TPMT*3A, seen in a subject with intermediate TPMT activity | very low | 12 | 44 (0%) | 44 (0%) | 88 (0%) | PGKB | [[163](#_ENREF_163)] |
| **TPMT*5** | ¡rs72552740 | 146T>C | Leu49Ser, seen in a subject with intermediate TPMT activity, thought to disrupt catalytic activity | very low | 12 | 45 (0%) | 45 (0%) | 192  (0%) | PGKB  [[160](#_ENREF_160)] | [[157](#_ENREF_157), [163](#_ENREF_163)] |
| **TPMT*6** | ¡rs75543815 | 539A>T | Tyr180Phe, seen in a Korean with low TPMT activity, accelerated degradation, enzyme activity 30% of wild type in vitro | low | 12 | 1214  (0%) | 116  (0%) | 400  (<1%) | [[164-166](#_ENREF_164)] | [[157](#_ENREF_157), [163](#_ENREF_163)] |
| **TPMT*7** | ¡rs72552736 | 681T>G | His227Gln, seen in an azathioprine-treated patient who developed severe leukopenia,  in vitro activity <10% of wild type | low | 12 | 45 (0%) | 45 (0%) | 192i  (0%) | PGKB  [[160](#_ENREF_160)] | [[157](#_ENREF_157), [167](#_ENREF_167), [168](#_ENREF_168)] |
| **TPMT*10** | ¡rs72552737 | 430G>C | Gly144Arg, seen in an azathioprine-treated patient who developed severe leukopenia, in vitro activity 70% of wild type | low | 12 | 45 (0%) | 45 (0%) | 89 (0%) | PGKB | [[157](#_ENREF_157), [167](#_ENREF_167), [169](#_ENREF_169)] |
| **TPMT*11** | ¡rs72552738 | 395G>A | Cys132Tyr, seen in a patient with low TPMT activity, in vitro activity 40% of wild type | low | 12 | 44 (0%) | 45 (0%) | 88 (0%) | PGKB | [[157](#_ENREF_157), [169](#_ENREF_169), [170](#_ENREF_170)] |
| **TPMT*12** | ¡no rsID | 374C>T | Ser125Leu, seen in an azathioprine-treated patient who developed severe leukopenia, in vitro activity 40% of wild type | reduced | 12 | ND | ND | ND | ND | [[157](#_ENREF_157), [167](#_ENREF_167), [169](#_ENREF_169)] |
| **TPMT*13** | ¡rs72552742 | 83A>T | Glu28Val, seen in an azathioprine-treated patient who developed severe leukopenia,  in vitro activity 60% of wild type | reduced | 12 | 45 (0%) | 45 (0%) | 88 (0%) | PGKB | [[157](#_ENREF_157), [167](#_ENREF_167), [169](#_ENREF_169)] |
| **TPMT*14** | ¡rs9333569 | 1A>G | Met1Val, seen in a European with low TPMT activity, no detectable activity in vitro | none | 12 | 57 (0%) | 58 (0%) | 86 (0%) | HapMap | [[169](#_ENREF_169), [171](#_ENREF_171)] |
| **TPMT*15** | ¡rs9333570 | IVS7-1G>A | splice defect, frameshift, premature stop codon, seen in European with low blood TPMT activity | low | 12 | 65 (0%) | 63(0%) | 90 (0%) | HapMap | [[169](#_ENREF_169), [171](#_ENREF_171)] |
| **TPMT*16** | ¡no rsID | 488G>A | Arg163His, seen in a Moroccan and a German with intermediate TPMT activity, this variant is thought to severely impair catalytic efficiency | low | 12 | 1214i(<1%) | 116 (0%) | 118 (0%) | [[164](#_ENREF_164), [165](#_ENREF_165)] | [[164](#_ENREF_164), [169](#_ENREF_169), [172](#_ENREF_172)] |
| **TPMT*17** | ¡no rsID | 124C>G | Gln42Glu, seen in a German with intermediate TPMT activity, in vitro activity 10% of wild type | very low | 12 | 1214i(<1%) | 116 (0%) | 118 (0%) | [[164](#_ENREF_164), [165](#_ENREF_165)] | [[164](#_ENREF_164), [169](#_ENREF_169)] |
| **TPMT*18** | ¡no rsID | 211G>A | Gly71Arg, seen in a German with intermediate TPMT activity, no detectable activity in vitro | low | 12 | 1214i(<1%) | 116 (0%) | 118 (0%) | [[164](#_ENREF_164), [165](#_ENREF_165)] | [[164](#_ENREF_164), [169](#_ENREF_169)] |
| **TPMT*20^e^** | ¡no rsID | 106G>A | Gly36Ser identified in a Japanese, in vitro activity is 10% of wild type | low | 12 | ND | ND | 200  (<1%) | [[173](#_ENREF_173)] | [[169](#_ENREF_169), [173](#_ENREF_173)] |
| **TPMT*20** | ¡no rsID | 712A>G | Lys238Glu, seen in Caucasian with intermediate TPMT activity, in vitro activity about 30% of wild type | low | 12 | 1048 (0%) | ND | ND | [[174](#_ENREF_174)] | [[169](#_ENREF_169), [174](#_ENREF_174)] |
| **TPMT*21** | ¡rs79321208 | 205C>G | Leu69Val, seen in 2 unrelated Caucasians with intermediate TPMT activity, no detectable activity in vitro | very low | 12 | 1048 (0%) | ND | ND | [[174](#_ENREF_174)] | [[169](#_ENREF_169), [174](#_ENREF_174), [175](#_ENREF_175)] |
| **TPMT*22** | ¡no rsID | 488G>C | Arg163Pro, seen in a Caucasian with intermediate TPMT activity, no detectable activity in vitro | very low | 12 | 1048 (0%) | ND | ND | [[174](#_ENREF_174)] | [[169](#_ENREF_169), [174](#_ENREF_174)] |
| **TPMT*23** | ¡rs74423290 | 500C>G | Ala167Gly, seen in a Caucasian (*3A/*23) with very low TPMT activity, in vitro activity about 60% of wild type | low | 12 | 200  (0%) | ND | ND | [[176](#_ENREF_176)] | [[169](#_ENREF_169), [176](#_ENREF_176)] |
| **TPMT*25** | ¡no rsID | 634T>C | Cys212Arg, seen in 2 unrelated Caucasians with intermediate TPMT activity, in vitro activity about 70% of wild type | low | 12 | 230  (0%) | ND | ND | [[175](#_ENREF_175)] | [[175](#_ENREF_175)] |
| **TPMT*8** | rs56161402 | 644G>A | Arg215His, seen in an African American (*1/*8) with intermediate TPMT activity,  in vitro activity similar to wild type | normal | 14 | 282  (0%) | 248i (<1%) | 192  (0%) | [[177](#_ENREF_177)] | [[157](#_ENREF_157), [169](#_ENREF_169), [177](#_ENREF_177)] |
| **TPMT*9** | ¡no rsID | 356A>C | Lys119Thr, does not appear to affect TPMT activity in vitro per data from expression studies and a normal methylator carrier | normal | 14 | 1214  (<1%) | 116  (0%) | 118  (0%) | [[164](#_ENREF_164), [165](#_ENREF_165)] | [[157](#_ENREF_157), [164](#_ENREF_164), [169](#_ENREF_169), [175](#_ENREF_175)] |
| **TPMT*24** | rs6921269 | 537G>T | Gln179His, seen in a Caucasian with intermediate TPMT activity, in vitro activity similar to wild type | normal | 14 | 230 (0%) | 58 (3%) | 87 (0%) | [[175](#_ENREF_175)] | [[175](#_ENREF_175)] |

ND = no data/no determined,

^a^ **=** evidence code assigned to each allele provides an indication of the strength and type of evidence for a drug-related phenotype and is based on the scheme presented in **Table 1** (green indicates clinically relevant evidence, yellow=potential clinical relevance, light orange= clinical relevance unknown, and dark orange= clinical relevance unsupported). For alleles with multiple types of supporting data, the code for the greatest strength of evidence is assigned,

b = in vitro kinetic parameters of TPMT activity: Km, Vmax, intrinsic clearance (Vmax/Km) of 6-TG or 6-MP S-methylation using mammalian recombinant protein cDNA expression system in COS-1 or COS-7 cells; in vivo TPMT activity measured in red blood cells from quantification of 14C-methyl-mercaptopurine produced by TPMT from 6-mercaptopurine with S-adenosyl- L-14C-methyl-methionine as the methyl donor,

^c^ = Allele frequency from 1000 Genome Project phase 1, HapMap, or specific reference; TPMT*1 allele frequency is estimated and takes into account the allele frequencies of other variants reported in this table with an evidence code ≤ 7

d = rs1800460 (TPMT*3B) and rs1142345 (TPMT*3C) are in tight linkage disequilibrium and most commonly observed together (TPMT*3A),

^e^ **=** Gly36Ser (106G>A) also referred to as TPMT*24 in [[169](#_ENREF_169)],

f **=** TPMT*4 frequency is from an Argentine (South American) population [[159](#_ENREF_159)],

^g^ = TPMT*3D haplotype formed by 292G>T on a TPMT*3A background

**Table S24. Predicted drug metabolizing phenotypes for TPMT genotypes**

| **Allele (frequency)** | **TPMT*1** | **TPMT*2** | **TPMT*3A** | **TPMT*3B** | **TPMT*3C** | **TPMT*4** |
| --- | --- | --- | --- | --- | --- | --- |
| **TPMT*1**  **(97%)** | *1/*1  (94.1%)  EM | *1/*2  (0.8%)  IM | *1/*3A  (5%)  IM | *1/*3B (0.2%)  IM | *1/*3C  (<0.05%) IM | *1/*4  (<0.05%) IM |
| **TPMT*2**  **(0.4%)** |  | *2/*2  (<0.05%)  PM | *2/*3A (<0.05%)  PM | *2/*3B (<0.05%)  PM | *2/*3C (<0.05%)  PM | *2/*4  (<0.05%)  PM |
| **TPMT*3A**  **(2.5%)** |  |  | *3A/*3A  (0.06%)  PM | *3A/*3B (<0.05%)  PM | *3A/*3C (<0.05%)  PM | *3A/*4 (<0.05%)  PM |
| **TPMT*3B**  **(0.1%)** |  |  |  | *3B/*3B (<0.05%)  PM | *3B/*3C (<0.05%)  PM | *3B/*4  (<0.05%)  PM |
| **TPMT*3C**  **(<0.1%)** |  |  |  |  | *3C/*3C (<0.05%)  PM | *3C/*4 (<0.05%)  PM |
| **TPMT*4**  **(<0.1%)** |  |  |  |  |  | *4/*4  (<0.05%)  PM |

Approximate expected genotype frequencies have been estimated based upon reported allele frequencies for Caucasians and Hardy Weinberg principals. Each potential TPMT genotype has been assigned a predicted drug metabolizing phenotype based on predicted metabolic phenotype of individual alleles (normal or reduced enzymatic function) such that extensive metabolizer (EM) is defined as 2 TPMT*1 alleles with normal activity; Intermediate metabolizers (IM) have 1 normal TPMT*1 and 1 reduced activity allele (TPMT*2 or *3A or *3B or *3C or *4); Poor metabolizers (PM) have 2 reduced activity alleles (any combination of TPMT*2, *3A, *3B or *3C or *4). Color shading is used to define expected drug response based on published outcomes data such that: Blue indicates likely normal response to thiopurine drugs; and Yellow indicates those at increased risk of drug toxicity such as myelosuppression when taking thiopurine drugs.

**Table S25. SLCO1B1 haplotypes, key variants, associated phenotypes, and frequencies**

| SLCO1B1 Haplotype or Key Variant | g.-11187G>A | g.-10499A>C | 217T>C, F73L | 245T>C, V82A | 388A>G, N130D | 411G>A, S137S | 452A>G, N151S | 455G>A, R152K | 463C>A, P155T | 467A>G, E156G | 521T>C, V174A | 571T>C, L191L | 597C>T, F199F | 721G>A, D241N | 1007C>G, P336R | 1058T>C, I353T | 1294A>G, N432D | 1385A>G, D462G | 1463G>C, G488A | 1628T>G, L543W | 1738C>T, R580X | 1929A>C, L643F | 1964A>G, D655G | 2000A>G, E667G | Haplotype Reference | Associated  Phenotype | Population Frequency |
| --- | --- | --- | --- | --- | --- | --- | --- | --- | --- | --- | --- | --- | --- | --- | --- | --- | --- | --- | --- | --- | --- | --- | --- | --- | --- | --- | --- |
| *1A |  |  |  |  |  |  |  |  |  |  |  |  |  |  |  |  |  |  |  |  |  |  |  |  | [[178](#_ENREF_178)] | wild type reference | 7% |
| *4 |  |  |  |  |  |  |  |  | **X** |  |  |  |  |  |  |  |  |  |  |  |  |  |  |  | [[179](#_ENREF_179)] | ↔R,A,S [[180](#_ENREF_180)] | ND |
| *8 |  |  |  |  |  |  |  |  |  |  |  |  |  |  |  |  |  | **X** |  |  |  |  |  |  | [[179](#_ENREF_179)] | ↔R [[180](#_ENREF_180)] | 0-1% |
| *1J |  |  |  |  |  |  |  |  |  |  |  | **X** |  |  |  |  |  |  |  |  |  |  |  |  | [[178](#_ENREF_178)] | ↔P [[178](#_ENREF_178)] | 39% |
| *19 |  |  |  |  |  |  |  |  |  |  |  | **X** |  |  |  |  |  |  |  |  |  | **X** |  |  | [[178](#_ENREF_178)] | ↔P [[178](#_ENREF_178)] | 1% |
| *10 |  |  |  |  |  |  |  |  |  |  |  |  |  |  |  |  |  |  |  |  |  |  | **X** |  | [[179](#_ENREF_179)] | ↔R [[180](#_ENREF_180)] | ≤2% |
| *11 |  |  |  |  |  |  |  |  |  |  |  |  |  |  |  |  |  |  |  |  |  |  |  | **X** | [[179](#_ENREF_179)] | ↔R [[180](#_ENREF_180)] | ND |
| *9 |  |  |  |  |  |  |  |  |  |  |  |  |  |  |  |  |  |  | **X** |  |  |  |  |  | [[179](#_ENREF_179)] | ↓R [[180](#_ENREF_180)], ↓pr [[179](#_ENREF_179)] | 0-5% |
|  |  |  |  |  |  |  |  |  |  |  |  |  |  |  |  |  |  |  |  |  |  |  |  |  |  | efficacy: ↔P [[181](#_ENREF_181)] |  |
| *1C |  |  |  |  |  |  |  | **X** |  |  |  |  |  | **X** |  |  |  |  |  |  |  |  |  |  | [[179](#_ENREF_179)] | ↓R [[180](#_ENREF_180)], ↔↓pr [[179](#_ENREF_179)] | <1% |
| *2 |  |  | **X** |  |  |  |  |  |  |  |  |  |  |  |  |  |  |  |  |  |  |  |  |  | [[179](#_ENREF_179)] | ↓R [[180](#_ENREF_180)] | 0-2% |
| *12 |  |  | **X** |  |  |  |  |  |  |  |  |  |  |  |  |  |  |  |  |  |  |  | **X** |  | [[179](#_ENREF_179)] | ↓R[[180](#_ENREF_180)] | ≤2% |
| *3 |  |  |  | **X** |  |  |  |  |  | **X** |  |  |  |  |  |  |  |  |  |  |  |  |  |  | [[179](#_ENREF_179)] | ↓R[[180](#_ENREF_180)] | ND |
| *13 |  |  |  | **X** |  |  |  |  |  | **X** |  |  |  |  |  |  |  |  |  |  |  |  |  | **X** | [[179](#_ENREF_179)] | ↓R [[180](#_ENREF_180)] | ND |
| *6 |  |  |  |  |  |  |  |  |  |  |  |  |  |  |  | **X** |  |  |  |  |  |  |  |  | [[179](#_ENREF_179)] | ↓R [[180](#_ENREF_180)] | 0-2% |
| *7 |  |  |  |  |  |  |  |  |  |  |  |  |  |  |  |  | **X** |  |  |  |  |  |  |  | [[179](#_ENREF_179)] | ↓R [[180](#_ENREF_180)] | 0-1% |
| key variant |  |  |  |  |  |  |  |  |  |  | **X** |  |  |  |  |  |  |  |  |  |  |  |  |  | - | activity: ↓P [[178](#_ENREF_178)], ↓S [[182](#_ENREF_182)] | 4-16% |
|  |  |  |  |  |  |  |  |  |  |  |  |  |  |  |  |  |  |  |  |  |  |  |  |  |  | ADRs: ↑S [[183](#_ENREF_183)] [[184](#_ENREF_184)],  ↑S,A,P [[185](#_ENREF_185)] |  |
|  |  |  |  |  |  |  |  |  |  |  |  |  |  |  |  |  |  |  |  |  |  |  |  |  |  | efficacy: ↔S [[186](#_ENREF_186)] [[187](#_ENREF_187)]  ↓A, [[187](#_ENREF_187)], ↔other statins [[187](#_ENREF_187)] [[188](#_ENREF_188)], ↓other statins [[189](#_ENREF_189)], ↓S [[183](#_ENREF_183)] |  |
| *5 |  |  |  |  |  |  |  |  |  |  | **X** |  |  |  |  |  |  |  |  |  |  |  |  |  | [[179](#_ENREF_179)] | ↓R [[180](#_ENREF_180)] ↓P [[190](#_ENREF_190)] | 0-2% |
| V174A-L643F |  |  |  |  |  |  |  |  |  |  | **X** |  |  |  |  |  |  |  |  |  |  | X |  |  | [[183](#_ENREF_183)] | ADR: trend↓S [[183](#_ENREF_183)] | ND |
| *5B |  |  |  |  |  |  |  |  |  |  | **X** |  | **X** |  |  |  |  |  |  |  |  |  |  |  | [[178](#_ENREF_178)] | ↔P [[178](#_ENREF_178)] | 1% |
| *20 |  |  |  |  | **X** |  |  |  |  |  |  |  | **X** |  |  |  |  |  |  |  |  | **X** |  |  | [[178](#_ENREF_178)] | ↔P [[178](#_ENREF_178)] | <1-5% |
| *18’+F199F |  |  |  |  | **X** |  |  |  |  |  |  |  | **X** |  |  |  |  |  | **X** |  |  |  |  |  | [[191](#_ENREF_191)] | ND | <1-3% |
| *18’’ |  |  |  |  | **X** | **X** |  |  | **X** |  |  | **X** | **X** |  |  |  |  |  |  |  |  |  |  |  | [[178](#_ENREF_178)] | ↔P [[178](#_ENREF_178)] | 7% |
| *14 |  |  |  |  | **X** |  |  |  | **X** |  |  |  |  |  |  |  |  |  |  |  |  |  |  |  | [[179](#_ENREF_179)] | ↔R,A,S [[180](#_ENREF_180)] | 16% |
| *18’ |  |  |  |  | **X** |  |  |  |  |  |  |  |  |  |  |  |  |  | **X** |  |  |  |  |  | [[180](#_ENREF_180)] | ↓R [[180](#_ENREF_180)] | ND |
| key variant |  |  |  |  | **X** |  |  |  |  |  |  |  |  |  |  |  |  |  |  |  |  |  |  |  | - | ADRs:  ↔S [[183](#_ENREF_183)], ↓S [[184](#_ENREF_184)] | 44-75% |
|  |  |  |  |  |  |  |  |  |  |  |  |  |  |  |  |  |  |  |  |  |  |  |  |  |  | efficacy: ↔S,P,A,F,L [[187](#_ENREF_187)] [[188](#_ENREF_188)], ↑S [[183](#_ENREF_183)] |  |
| *1B |  |  |  |  | **X** |  |  |  |  |  |  |  |  |  |  |  |  |  |  |  |  |  |  |  | [[179](#_ENREF_179)] | ↔R [[180](#_ENREF_180)], ↔pr [[179](#_ENREF_179)], ↔P [[192](#_ENREF_192)], ↑P [[190](#_ENREF_190)] | 8-54% |
| § *15 |  |  |  |  | **X** |  |  |  |  |  | **X** |  |  |  |  |  |  |  |  |  |  |  |  |  | [[179](#_ENREF_179)] | ↓R [[180](#_ENREF_180)] ↓P [[192](#_ENREF_192)] [[193](#_ENREF_193)] [[181](#_ENREF_181)]; ↓S ADR [[183](#_ENREF_183)] | 2-16% |
| *16’’ |  | **X** |  |  | **X** |  |  |  |  |  | **X** |  |  |  |  |  |  |  |  |  |  |  |  |  | [[194](#_ENREF_194)] | ND[[195](#_ENREF_195)] | 8% |
| *16’’’ |  | **X** |  |  | **X** |  |  |  |  |  | **X** |  | **X** |  |  |  |  |  |  |  |  |  |  |  | [[178](#_ENREF_178)]  [[191](#_ENREF_191)] | ↔P [[178](#_ENREF_178)] | <1-9% |
| *15+P336R |  |  |  |  | **X** |  |  |  |  |  | **X** |  |  |  | **X** |  |  |  |  |  |  |  |  |  | [[196](#_ENREF_196)] | ↓P[[192](#_ENREF_192)] ª; ↑P ADR [[196](#_ENREF_196)] | 2% |
| *15B |  |  |  |  | **X** |  |  |  |  |  | **X** |  | **X** |  |  |  |  |  |  |  |  |  |  |  | [[178](#_ENREF_178)] | ↓P [[178](#_ENREF_178)],  ↓P efficacy [[197](#_ENREF_197)] | 5% |
| *17’’ | **X** |  |  |  | **X** |  |  |  |  |  | **X** |  | **X** |  |  |  |  |  |  |  |  |  |  |  | [[191](#_ENREF_191)]  [[178](#_ENREF_178)]  [[198](#_ENREF_198)] | ↓P [[178](#_ENREF_178)] | <1-8% |
| *17’ | **X** |  |  |  | **X** |  |  |  |  |  | **X** |  |  |  |  |  |  |  |  |  |  |  |  |  | [[197](#_ENREF_197)] | ↓P efficacy [[197](#_ENREF_197)] | 1-5% |
| *21 | **X** |  |  |  | **X** |  |  |  |  |  |  |  | **X** |  |  |  |  |  |  |  |  | **X** |  |  | [[178](#_ENREF_178)] | see g.-11187 G>A. | <1-10% |
| *21+L191L | **X** |  |  |  | **X** |  |  |  |  |  |  | **X** | **X** |  |  |  |  |  |  |  |  |  |  |  | [[191](#_ENREF_191)] | ND | 0-5% |
| H9-C | **X** |  |  |  | **X** |  |  |  |  |  |  | **X** |  |  |  |  |  |  |  |  |  |  |  |  | [[198](#_ENREF_198)] | ND | 1% |
| H12-C | **X** |  |  |  | **X** |  |  |  |  |  |  |  |  |  |  |  |  |  |  |  |  |  |  |  | [[198](#_ENREF_198)] | ND | <1% |
| H10-C | **X** |  |  |  |  |  |  |  |  |  |  | **X** |  |  |  |  |  |  |  |  |  |  |  |  | [[198](#_ENREF_198)] | ND | 1% |
| H11-C | **X** |  |  |  |  |  |  |  |  |  | **X** |  | **X** |  |  |  |  |  |  |  |  |  |  |  | [[198](#_ENREF_198)] | ND | <1% |
| key variant | **X** |  |  |  |  |  |  |  |  |  |  |  |  |  |  |  |  |  |  |  |  |  |  |  | - | ↓P [[178](#_ENREF_178)] | 0-34% |
| *16’ |  |  |  |  |  |  | **X** |  |  |  |  |  |  |  |  |  |  |  |  |  |  |  |  |  | [[192](#_ENREF_192)] | ↓P [[192](#_ENREF_192)] | 0-4% |
| L543W |  |  |  |  |  |  |  |  |  |  |  |  |  |  |  |  |  |  |  | **X** |  |  |  |  | [[199](#_ENREF_199)] | ↓P [[200](#_ENREF_200)]; ↑P ADR [[199](#_ENREF_199)] | <1% |
| R580X |  |  |  |  |  |  |  |  |  |  |  |  |  |  |  |  |  |  |  |  | **X** |  |  |  | [[201](#_ENREF_201)] | ↓SLCO1B1 expression | <1% |

ND = no data/not determined, H’ = haplotype, A = atorvastatin, R = rosuvastatin, S= simvastatin, F = fluvastatin, L = lovastatin, P = pravastatin pr = probe substrate, ↑↓↔ = change in transporter activity based on in vitro/in vivo data, efficacy, adverse drug reactions (ADR), or expression. Efficacy should improve with increased transporter activity; and inversely, ADRs should decrease with increased transporter activity. § = The evidence is ambiguous and controversial when data is discordant for these relationships. Red = poor activity/efficacy/tolerability, green = improved activity/ efficacy/ tolerability, yellow = no effect, blue = contradictory evidence, pink = substrate-specific, grey = key variants with variant-specific phenotypes without regard to the multi-variant haplotypes in which they may be harbored, ª = P336R, N130D phase undetermined.

**Table S26. Studies of SLCO1B1 Variation for Clinical Outcomes in Simvastatin-Treated Patients**

| **References** | **Subjects, Drug** | **Comparison Groups** | **Key Endpoint Results** | **Conclusions and Comments** |
| --- | --- | --- | --- | --- |
| ­­[[181](#_ENREF_181)] | n=107  (n=69 European Americans (EA),  n=38 African Americans (AA)), pravastatin | **SLCO1B1**  V174A (521T>C)  n=88: TT (wild type) n=17: TC n=2: CC  **allele frequencies (EA, AA)**  N130D (388G>A): 0.62, 0.23  V174A (521T>C): 0.15, 0.01  G488A (1463G>C): 0, 0.01  **diplotypes (EA/AA)**  *1A/*1A: 26,3  *1A/*1B: 23,11 *1B/*1B: 2,23 *1A/*5: 1,0 *1B/*15: 7,1 *15/*15: 2,0  **ABCC2**  3563G/A, 4544G/A  **ABCB11**  1331T/C, 2029A/G  **ABCG2**  34G/A, 421C/A | **Pravastatin AUC SLCO1B1** *1A/*15 > *1A/*1A: ↑45%, p=0.013 *1A/*15 > *1B/*1B: ↑80%, p=0.001  *15/*15 > *1A/*1A: ↑91.7%, p=0.017 *15/*15 > *1B/*1B: ↑149%, p=0.011 *1A/*1A > *1B/*1B: NS trend *1B/*15 > *1A/*1A: NS trend *1B/*15 > *1B/*1B: NS trend 521 CC > TT: p=0.009 521 TC > TT: p=0.015  EA **>** AA: p=0.010  **Pravastatin Cmax SLCO1B1** *1A/*15 > *1A/*1A: ↑46%, p=0.025 *1A/*15 > *1B/*1B: ↑78%, p=0.007 *1B/*15 > *1A/*1A: NS trend *1B/*15 > *1B/*1B: NS trend *15/*15 > *1A/*1A: NS trend *15/*15 > *1B/*1B: NS trend 521 TC > TT: p=0.042 EA **>** AA: p=0.009 | Subjects were given a single dose of 40mg pravastatin and assessed for pravastatin and bilirubin levels. Pravastatin levels were significantly higher (decreased uptake) in SLCO1B1 V174A and *15 (N130D-V174A) versus *1B (N130D) and *1A carriers (EA+AA). A gene-dose effect was seen for the V174A (521T>C) SNP following the additive genetic model. Factors explaining inter-individual variability in pravastatin AUC were: the V174A (521T>C) genotype (6.5%), ethnicity (6%), gender (11%), and BSA (12%). European Americans showed significantly greater pravastatin levels than African Americans including after adjusting for 521T>C genotype, gender, BSA, and assay sensitivity. Significant ethnic differences in variant allele frequency were seen for many alleles (SLCO1B1*1B, *5, *15; ABCC2 4544A; ABCB11 1331C, 2029G). Genotypes for ABCC2, ABCB11, and ABCG2 were not associated with pravastatin nor for SLCO1B1 variants (521T>C, 388A>G, *1A/*1A, *1A/*1B, *1B/*1B, *1A/*15, *1B/*15, *15/*15) and bilirubin levels. SLCO1B1*9 was not associated with pravastatin pharmacokinetics but the number of carriers was low. Although this study was not for simvastatin, it illustrates the impact of the V174A variant as carried predominantly in the N130D-V174A (*15) haplotype, rather than the V174A (*5) allele. |
| [[192](#_ENREF_192)] | n=143 healthy Japanese, n=23 (PK), n=120 (genotyping), pravastatin | **SLCO1B1 variants**  (-11187G>A): 0.073 N130D: 0.629 F199F (597C>T): 0.430 L191L (571T>C): 0.358 V174A: 0.158 N151S: 0.037 P336R: 0.012  C485F: 0.008 1240C>A: 0.008  **Haplotypes**  *1B (N130D): 0.458  *1A: 0.325 *15 (N130D-V174A): 0.15 *16’ (N151S): 0.038  *5 (V174A): 0.0  **Diplotypes**  n=37: *1A/*1B (0.308) n=26: *1B/*1B (0.217) n=17: *1B/*15 (0.142) n=14: *1A/*15 (0.117) n=13: *1A/*1A (0.108) n=4: *1B/*16’ (0.033) n=2: *15/*16’ (0.017) n=1: *15/*15 (0.008) n=1: *1A/*16’ (0.008) n=1: *16’/*16’ (0.008) | **total CL pravastatin**  *1B/*1B > *1B/*15, p<0.05  n=2 *1A/*1A: 2.66  n=2 *1B/*16’: 2.55  n=4 *1B/*1B: 2.39 ± 0.44 n=4 *1A/*1B: 1.95 ± 0.72 n=9 *1B/*15: 1.57 ± 0.32  n=1 P336R (V174A,N130D-phase unknown): 1.22  n=1 *15/*15: 0.79  **non-renal CL pravastatin**  *1B/*1B > *1B/*15, p<0.05  n=2 *1A/*1A: 2.22  n=2 *1B/*16’: 2.12  n=4 *1B/*1B: 2.01 ± 0.42  n=4 *1A/*1B: 1.45 ± 0.72 n=9 *1B/*15: 1.11 ± 0.34  n=1 P336R+V174A,N130D: 0.81 n=1 *15/*15: 0.28 | 23 healthy Japanese males were given a single 10mg dose pravastatin and assessed for genetic differences in pharmacokinetics (e.g. AUC, CL). One SLCO1B1 P336R carrier who also carried 174V/A and 130D/D, had P336R-N130D and V174A-N130D in cis, but unknown phase for the P336R and V174A variants; and remaining genotypes fell into 7 allelic categories. Significantly reduced non-renal and total clearance of pravastatin was seen in *1B/*15 carriers of relative to *1B/*1B, following an additive or dominant genetic model. *15 homozygotes exhibited the highest AUC and lowest total and non-renal CL of all genotypes. No gene-dose effect of 521T>C (V174A) on systemic exposure to pravastatin was seen (not fitting the additive genetic model). The single P336R heterozygote showed relatively higher AUC and lower total and non-renal CL than carriers of *1A and *1B. Haplotypic and allelic frequencies were separately assessed in 120 healthy unrelated Japanese. Significant ethnic differences in SLCO1B1 allele frequencies exist (p<0.05) with N130D more frequent in Japanese and African Americans than in Caucasians and V174A less frequent in African Americans relative to Japanese and Caucasians. The N151S variant is Japanese-specific while the P155T (0.16 in Caucasians) and E667G (0.34 in African Americans) were not seen. SLC22A8 variants (153G>A, 723T>A, A389V) were not associated with pravastatin pharmacokinetics. Although this study was not for simvastatin, it illustrates the impact of the V174A variant as carried in the N130D-V174A, *15 haplotype, rather than the *5 allele, the insignificant difference between *1A and *1B in this study. |
| [[189](#_ENREF_189)] | n=66 Japanese,  n=11: atorvastatin,  n=22: pravastatin,  n=33: simvastatin | **SLCO1B1** 521T>C (V174A)  n=39-44: 521 TT  n=19-20: 521 TC | **% reduction total-C**  TT (−22.3%) > TC (−16.5%), p<0.05  **% reduction LDL-C**  TT (−29.0%) > TC (−12.4%), NS trend, p=0.094  **% reduction TG**  NS, p=0.492  **% increase HDL-C**  NS, p=0.745 | Subjects were treated with atorvastatin, pravastatin, or simvastatin (dose not reported, data not stratified by drug) and assessed for drug efficacy (e.g. lipid profile changes) relative to a single SLCO1B1 V174A (521 T>C). The V174A variant was associated with significantly less total-C reduction relative to non-carriers, according to an additive or dominant genetic model. |
| [[187](#_ENREF_187)] | n=2,735 (n=2,454 (98%) Caucasians (CA), n=160 African Americans (AA), n=36 Asians (AS),  n=85 Hispanics (HS)),  n=1902: atorvastatin, n=477: fluvastatin, n=476: lovastatin, n=462: pravastatin, n=468: simvastatin | **SLCO1B1** (haplotypes unknown)  rs4149036 C>A (intronic)  n=787: CC  n=424: CA  n=54 (4.3%): AA  rs2291073 T>G (intronic)  n=266: TT  n=39: GT  n=1 (2.5%): GG  V174A (521T>C)  n=195: TT  n=78: CT  n=5 (1.6%): CC  rs4149080 (intronic)  **frequencies (AS, AA, CA, HS)**  V174A: 0.08, 0.04, 0.16, 0.15  N130D: 0.61, 0.74, 0.41, 0.41 L643F: 0.01, 0.05, 0.06, 0.04  G488A: 0, 0.05, 0, 0  P155T: 0.03, 0.10, 0.16, 0.09 F73L: 0, 0, 0.04, 0 | **triglycerides in Caucasians**  atorvastatin and rs4149036: AA(-9.5) < AC(-20.1), CC(-19.2), p=0.00237  lovastatin and rs2291073: GG(70.6) > GT(-13.6), TT(-10.0), p=0.00339  **HDL-C in Caucasians**  atorvastatin and V174A (521T>C): TT(5.5) > CT(0.7), CC(0.5), p=0.037  fluvastatin and rs4149056: CC(0.5), CT(0.65) < TT(5.45), p=0.0061  statin and rs4149080: p=0.026 (data not given)  **LDL-C in Caucasians**  pravastatin and N130D, V174A: NS | Genetics for 43 SNPs in 16 genes were analyzed in 70% of 3,194 subjects in the Atorvastatin Comparative Cholesterol Efficacy and Safety Study (ACCESS) who were dosed 10mg atorvastatin or simvastatin, or 20mg fluvastatin, lovastatin or pravastatin, and evaluated at 6, 12, 18, and 24 weeks for response (e.g. decreased TG, total–C, LDL-C, and increased HDL-C). Doses were increased in non-responders. Significant association was found for variants of ABCB1, CYP3A5, CYP3A4, SLCO1B1, and non-ADME genes (APOE, LDLR, LIPC, LPL, ACE, and CETP). Gender and age were most significantly associated. Associations were not seen for SLCO1B1 L643F, G488A, P155T, F73L, or N130D. |
| [[182](#_ENREF_182)] | n=32 healthy Caucasians, simvastatin | all n=32: CYP3A5*3/*3  **SLCO1B1**  V174A (521T>C)  n=16: TT n=12: TC  n=4: CC  N130D (388A>G)  n=16: AA  n=8: GA  n=8: GG  **SLCO1B1** *1A (61%), *1B (5%), *5 (<1%), *15 (5%), *16’’ (8%), *17’ (5%)  **SLCO1B1** (521T>C, 388A>G)  n=16: *1A/*1A (TT-AA)  n=2: *1A/*15 (TC-AG)  n=2: *1A/*16’’ (TC-AG)  n=3: *1A/*17’ (TC-AG)  n=2: *1B/*15 (TC-GG)  n=2: *1B/*16’’ (TC-GG)  n=1: *1B/*17’(TC-GG)  n=1: *5/*15 (CC-AG)  n=1: *16’’/*16’’ (CC-AG)  n=2: *16’’/*17’ (CC-AG) | **SLCO1B1 V174A (521T>C)**  **Simvastatin acid AUC** (ng*h/mL)  CC > TC ↑120%, p<0.001  CC > TT: ↑221%, p<0.001  TC vs TT: NS  TC*15 (25) vs TC*16 (19) vs TC*17 (37): NS  **Simvastatin acid Cmax** (ng/mL)  CC (6.6) > TC (3.0): ↑162%, p<0.001  CC (6.6) > TT (2.2): ↑200%, p<0.001  **Simvastatin acid Tmax** (h)  TC (4) < TT (5): p<0.05  CC (3) < TT (5): p<0.05  **Mean AUC ratio simvastatin acid:lactone:**  CC > TC: p=0.001  CC > TT: p<0.001  **Simvastatin lactone AUC Cmax:**  CC > TT: NS trend | Subjects were given a single 40mg simvastatin dose and assessed for statin pharmacokinetics (AUC, Cmax, t1/2, tmax). Only CYP3A5*3/*3 (g.6986A>G) non-expressors were included in the study. The 521T>C (V174A) homozygotes exhibit significantly greater statin levels and lower metabolic ratio than heterozygotes or WT homozygotes for the active simvastatin acid but not for the inactive lactone form. 521T>C heterozygotes with *15, *16’’, or *17’ alleles were not significantly different for statin AUC. One of three *1A/*17’ carriers with unusually high statin levels was excluded from the analysis for being an outlier. The V174A PK association follows an additive genetic model. |
| [[193](#_ENREF_193)] | n=11 healthy Korean males,  pravastatin,  pitavastatin | **SLCO1B1**  *1A (wild type)  *15 (N130D-V174A)  n=6: *1A/*1A n=5:*15/*15 | **Pravastatin**  AUC: *15/*15 > *1A/*1A: ↑1.99x, p<0.01  Cmax: *15/*15 > *1A/*1A: ↑2.12x, p<0.01  CL/F: *15/*15 > *1A/*1A: ↓2.07x, p<0.01  **Pitavastatin**  AUC: *15/*15 > *1A/*1A: ↑2.62x, p<0.001  Cmax: *15/*15 > *1A/*1A: ↑3.12x, p<0.001  CL/F: *15/*15 > *1A/*1A: ↓2.88x, p<0.01 | Subjects were given a single 40mg pravastatin or 4mg pitavastatin dose and genotypes for 2 variants (N130D and V174A) to assess the genetics of statin PK. AUC and Cmax were significantly increased and clearance significantly decreased in *15/*15 versus *1A/*1A carriers; and this was confirmed in vitro. In vitro work showed no effect of *15 on fluvastatin. This paper helps explain *15 effects even if not for simvastatin. |
| [[183](#_ENREF_183)] | n=172 (n=83 statin-induced myopathy cases, n=89 matched controls),  Europeans from the UK,  simvastatin | **SLCO1B1**, frequency in controls  V174A: 0.15  N130D: 0.44  S137S: 0.18  P155T: 0.18  L643F: 0.08  rs4363657 T>C (intronic): 0.13  **simvastatin dosing**  n=6,031: 80mg/d  n=6,033: 20mg/d  n=16,643: 40mg/d | **Myopathy risk GWAS, 80mg in 83 cases, 89 controls**  rs4363657 T>C: p=4E-9, Bonferroni-corrected p=0.001  per copy of variant: OR=4.3  variant heterozygote: OR=4.4  variant homozygote: OR=17.4  V174A: p=2E-9  per copy of variant: OR=4.5  variant homozygote: OR=16.9  variant frequency in cases vs controls: 0.46 vs 0.13  1yr cumulative risk: WT (0.3%) < het (1%) < hom (15%)  5yr cumulative risk: WT (0.6%) < het (3%) < hom (19%)  N130D, P155T, S137S: NS (confidence interval crosses 1)  V174A-N130D: ↓ADR risk, p=0.03 *****  V174A-L643F: ↓ADR risk, p=0.06 trend  **Replication, 40mg in 16,643 patients**  per copy of V174A variant: OR=2.6, p=0.004  **GWAS and replication studies combined**  per copy of V174A variant: OR=4.7, p=3E-28  **Response, LDL-C in 16,643 patients using the joint genotype test (V174A/N130D)**  1.28% less reduction per copy of V174A: p<0.0001  0.62% more reduction per copy of N130D: p=0.0005   \| **LDL-C % reduction** \| \| \| \| \| --- \| --- \| --- \| --- \| \|  \| 174VV \| 174VA \| 174AA \| \| 130NN \| 40.6% \| 38.8% \| 40.3% \| \| 130ND \| 40.9% \| 39.6% \| 39.1% \| \| 130DD \| 42.0% \| 40.8% \| 39.1% \|   **linkage disequilibrium**  V174A-rs4363657: r2=0.97  V174A-N130D and V174A-L643F: r2<0.2 | Association was tested for simvastatin side effects and response using 316,184 genetic markers in 83 myopathy cases and 89 treated controls and replicated in 16,643 subjects. Genetic association to response (LDL-C reduction) was also tested in 16,643 patients.  *Myopathy*: SLCO1B1 N130D, P155T, and S137S showed no significant impact on the risk of myopathy, but rs4363657 (intronic) and V174A were significantly associated with greater risk of simvastatin-induced myopathy (threshold p≤5E-7). V174A accounted for >60% of myopathy cases; and its association was replicated. V174-N130D and V174-L643F haplotypes were associated with lower risk of myopathy. ******The protective effect of V174-N130D against myopathy is contrary to data from other studies.  Response:* In a joint genotype test for diplotypes formed from V174A and N130D, each variant significantly influenced the response to 40mg simvastatin daily: 1.28% less reduction in LDL-C per copy of V174A (less efficacy), and 0.62% greater reduction LDL-C per copy N130D (greater efficacy). |
| [[185](#_ENREF_185)] | n=452 total hypercholesterolemic,  n=389: Caucasians,  n=22: African Americans,  multiple statins | **SLCO1B1**  V174A (521T>C)  **composite adverse events (CAE) cases**  n=54: drug discontinuation  n=49: myalgia  n=9: CK elevation | **Female gender and CAE**  OR=2.0, p=0.04  **CAE cases vs controls**  % female: 66% > 50%, p<0.01  V174A: 35% > 25%, p=0.03  **%CAE for 0 vs 1 vs 2 variant V174A alleles**  19% < 27% < 50% trend, p=0.01  **Drug discontinuation all ADRs (n=99)**  V174A: p=0.03, chi-squared 4.8  Female gender: p=0.004, chi-squared 8.4 | A third of subjects were each given 8-weeks of 10mg atorvastatin, 20mg simvastatin, or 10mg pravastatin followed by 8-weeks of increased dose (80mg, 80mg, and 40mg respectively) and assessed for drug tolerance (controls) versus intolerance (cases) given by composite adverse events (CAE). V174A was significantly associated with CAE. A gene-dose effect was observed for V174A and %CAE with greatest risk for simvastatin. Other variants (CYP3A4*1B; CYP2C8*3, *4; CYP2C9*3; CYP2D6*4, *10) showed no association. |
| [[186](#_ENREF_186)] | n=601 Caucasians with prior myocardial infarction,  n=310: rosuvastatin,  n=291: simvastatin | **SLCO1B1**  521T>C (V174A) n=431: TT (wild type) n=170: CT+CC  **CYP3A5*3**  n=527: hom wild type n=74: het var  **ABCG2**  421C>A (Q415K) n=470: CC (wild type) n=131: CA+AA  **CYP2C9*2 or *3**  n=370: hom wild type n=230: hom/het var  **CYP2C19*2**  n=439: hom wild type n=162: hom/het var | **Simvastatin effects**  **LDL-C at 3 months SLCO1B1:** NS  wild type (n=200) vs 521 CT+CC var (n=91) 79.52 ± 25.36 > 77.77 ± 25.25, p=0.586, NS  **Rosuvastatin effects**  **LDL-C at 3 months SLCO1B1:** NS  wild type (n=231) vs CT+CC var (n=79) 73.24 ± 23.41 > 76.34 ± 20.85, p=0.298, NS  **LDL-C at 3 months CYP3A5**  *1/*1 (n=271) vs *1/*3 (n=39) 75.38 ± 23.05 > 64.65 ± 18.83, p=0.006  **LDL-C at 3 months ABCG2 421C>A**  CC (n=239) vs var (n=67 CA n=4 AA) 75.85 ± 23.37 > 67.92 ± 19.80, p=0.010  **Tx predicts target LDL-C in randomized univariate and multivariate regression at 3 months**  univariate var* (n=186): OR=2.307, p=0.006  multivariate var* (n=186): OR=2.289, p=0.017  univariate, all genotypes (n=601): OR=1.376, p=0.053  **Alanine transaminase IU/L, ABCG2 421C>A**  CC (n=238) vs CA + AA (n=71) 31.21 vs 29.82: NS, p=0.484 | Subjects with prior myocardial infarction (MI) were randomized on 40mg/day simvastatin or 10mg/day rosuvastatin for 3 months and assessed for efficacy (e.g. LDL-C reduction) and tolerability. There were no differences for patients with variants of SLCO1B1, CYP2C9, or CYP2C19. However significantly reduced LDL-C was seen for carriers of CYP3A5 and ABCG2 variants (p≤0.010) relative to wild type which remained significant after adjustment for baseline LDL-C in multivariate linear-regression (p=0.022 and p=0.006, respectively). Patients randomized to rosuvastatin were 2.3-times more likely to achieve target LDL-C than with simvastatin. Genotype interactions were significant for variants in ABCG2 (p=0.031) and CYP3A5 (p=0.020). Carriers of at least one CYP3A5 and/or ABCG2 variant (n=186) showed greater likelihood to achieve target LDL-C levels (54.0% with rosuvastatin vs 33.7% with simvastatin). No difference in hepatotoxicity (given by alanine transaminase levels) was seen for ABCG2 variant carriers compared to wild type.  * carrier of ≥1 CYP3A5 and/or ABCG2 variant |
| [[188](#_ENREF_188)] | n=1,885 hypercholesterolemic Caucasians (n=668 myocardial infarction, n=1,217 no myocardial infarction),  multiple statins | **SLCO1B1** N130D (388A>G)  V174A (521 T>C)  rs2291077 T>A (intronic)  rs12369881 G>A (intronic)  **inferred** **haplotypes**  non-variant: 0.57  N130D, not V174 (*1B): 0.27  V174A, not N130D (*5): <0.01  V174A-N130D (*15): 0.15 | **Adjusted odds ratio of statin-mediated efficacy ***  **reference**, NS  n=603 reference (0.54, 0.38-0.78)  n=1282 variant carriers (0.38, 0.30-0.49)  **N130D**, NS  n=1014 non-carriers (0.47, 0.36-0.63)  n=720 N130D het (0.35, 0.25-0.49)  n=151 N130D hom (0.43, 0.21-1.53)  **V174A**, NS n=1896 non-carriers (0.43, 0.35-0.53)  n=27 V174A carriers (0.13, 0.01-1.53)  ***15 (N130D-V174A)**, NS  n=1349 non-carriers (0.43, 0.34-0.55)  n=500 *15het (0.43, 0.28-0.66)  n=36 *15hom (0.14, 0.02-0.83)  **Synergy Index of statin effectiveness****  Variant (vs reference): 1.52 (0.99-2.34), p=0.05, q=0.32  **Linkage disequilibrium**  rs2291077-N130D, r2=0.92  rs12369881-V174A, r2=0.86  rs2291077-rs12369881:  (T-G) tags reference  (A-G) tags *1B (N130D)  (T-A) tags *5 (V174A)  (A-A) tags *15 (N130D-V174A) | In a case-control study of Caucasians haplotype-tagging SNPs (14 for SLCO1B1 and 10 for ABCB1) were tested for association with statin-mediated reduction in myocardial infarction (MI) incidence. Treatment in cases included atorvastatin, pravastatin, simvastatin and other unnamed statins (sample number insufficiently large enough to stratify data by statin). The odds ratio of efficacy with treatment was not significantly associated with SLCO1B1 alleles, although the OR was least changed with treatment in *15 homozygotes and V174A carriers (including *15 carriers). The N130D variant showed no trend in the odds of MI risk. MI risk did not differ according to SLCO1B1 genotypes (data not shown); and no SLCO1B1 SNPs significantly interacted with statin treatment. Intronic SNPs efficiently tagged coding SNPs and SLCO1B1 haplotypes. The increased OR of statin efficacy in non-variant carriers compared to variant carriers resulted in a nominally significant synergy index of 1.52.  * adjusted: sex, index date, age, region  **adjusted: sex, index date, age, region, ischemic heart disease, calcium channel blocker use |
| [[184](#_ENREF_184)] | n=4196 type II diabetics from Scotland,  simvastatin | **SLCO1B1**  V174A: 0.16 N130D: 0.38  Genotype scores associated with statin tolerance and predicted statin exposure (AUC) per N130D-V175A diplotype:  n=287: 0  n=1221: 1  n=1707: 2  n=884: 3  n=97: 4 | **Drug intolerance**  174A: OR=2.05, p=0.043 130D: protective OR=0.71, p=0.026  **LDL-C reduction: all tolerant subjects only**  174A less LDL-C reduction: p=0.01, NS 130D more LDL-C reduction: p=0.048, NS  **Associations with genotype score**  drug intolerance with score increase: OR=1.14, p=0.0200  dose ≥40mg: p=0.0023   \| **Genotype Score** \| \| \| \| \| --- \| --- \| --- \| --- \| \|  \| 174VV \| 174VA \| 174AA \| \| 130NN \| 1423 (33.9)-2 \| 186 (4.4)-3 \| 9  (0.2)-4 \| \| 130ND \| 1221 (29.1)-1 \| 698 (16.6)-3 \| 35  (0.8)-4 \| \| 130DD \| 287 (6.8)-0 \| 284  (6.8)-2 \| 53 (1.3)-4 \| \| # subjects (%) - genotype score  low (light) to high (dark) = predicted AUC \| \| \| \| | In diabetic subjects given simvastatin, drug intolerance was associated with 130D and 174A variants in SLCO1B1. The 174A allele predominates over the 130D allele in combined diplotypes corresponding to differences in drug tolerance. Genotype scores (0-4) generated from the nine diplotypes are associated with drug intolerance and predict simvastatin AUC. The V174A and N130D data fit the recessive and additive genetic models, respectively.  Val174Ala and Asn130Asp diplotypes correspond to the following star allele diplotypes:   \|  \| 174VV \| 174VA \| 174AA \| \| --- \| --- \| --- \| --- \| \| 130NN \| *1A/*1A \| *1A/*5 \| *5/*5 \| \| 130ND \| *1A/*1B \| *1A/*15 or *1B/*5 \| *5/*15 \| \| 130DD \| *1B/*1B \| *1B/*15 \| *15/*15 \| |

NS = not significant, ND = not determined, unk = unknown, WT = wild type, het = heterozygote, hom = homozygote, MAF = mutant allele frequency, H’ = haplotype, MAF = minor allele frequency, PK = pharmacokinetics, PD = pharmacodynamics, DDI = drug-drug interaction, AUC = area under the concentration-time curve, Cmax = peak plasma concentration, Tmax = time of peak concentration, t1/2 = half-life, CL = clearance, Ae = amount excreted into urine, ke = elimination rate constant, ADR = adverse drug reaction, TG = triglycerides, total-C = total cholesterol, LDL-C = low-density lipid cholesterol, HDL-C = high-density lipid cholesterol, BSA = body surface area, HeFH = heterozygous familial hypercholesterolemia, CHD = coronary heart disease, CVD = cardiovascular disease, MI = myocardial infarction, CK = creatine kinase, ALT = alanine aminotransferase, OR = odds ratio, CI = confidence interval, ADME = absorption, distribution, metabolism, elimination.

**Table S27. Metabolic phenotypes, frequency and evidence scoring of allelic variants for *SLCO1B1*-simvastatin**

| **Allele** | **Key Variant dbSNP ID** | **Nucleotide Change (cDNA)** | **Mutation Effect** | **Phenotype** | | **Evidence Code**^a^ | **Allele Frequency (# Subjects (%))** | | | | | | **Key References (PMID#)** |
| --- | --- | --- | --- | --- | --- | --- | --- | --- | --- | --- | --- | --- | --- |
|  |  |  |  | **In Vitro/ In Vivo** | **Clinical** |  | **Caucasian** | | **African** | | **Asian** | **Reference^b^** |  |
| SCLO1B1*1 | wild type | reference | lacks variants below | normal | normal | 1 | (80%) | | (96%) | | (89%) | Estimated **^b^** | reference in studies below |
| SCLO1B1 V174A *5 (also in 7 haplotypes) | rs4149056 | 521T>C | **¦** Val174Ala  in TMD, ↓in vivo trafficking ^(75=PM:21178985)^**^c^** | in vivo adult age-specific**‡** decrease | simvastatin ↑ADR  (↔↓ efficacy) | 1 | V174A: 2454 (16%)  *5: 1885 (<1%)  *5B: 41 (1%)  *15: 32 (5%)  *15B: 41 (5%) *16”: 32 (8%)  *16’’’: (<1%)  *17’: 32 (5%) | | V174A: 160 (4%)  16’’’: (4%) | | V174A: 86 (11%)  *5: 267 (<1%)  *15: 267 (10%) *16: 120 (4%) | [[178](#_ENREF_178), [182](#_ENREF_182), [183](#_ENREF_183), [185](#_ENREF_185), [187](#_ENREF_187), [188](#_ENREF_188), [191](#_ENREF_191), [192](#_ENREF_192), [202](#_ENREF_202)] | [[178](#_ENREF_178), [180-182](#_ENREF_180), [184-187](#_ENREF_184), [190-192](#_ENREF_190), [195-197](#_ENREF_195), [203-208](#_ENREF_203)] |
| SCLO1B1 I353T *6 | rs55901008 | 1058T>C | **¦** Ile353Thr  in TMD ^(71=PM:11477075)^ | in vitro decrease | ND | 4scd | I335T: 44 (2%) | | I335T: 22 (0%) | | I335T: 120 (0%) | [[179](#_ENREF_179), [192](#_ENREF_192)] | [[179](#_ENREF_179), [180](#_ENREF_180)] |
| SLCO1B1 V82A *3 (also in *13) | rs56061388  rs72559745 | 245T>C  467A>G | **¦** Val82Ala in TMD, ↓in vivo trafficking ^(75=PM:21178985)^, + Glu156Gly | in vitro decrease | ND | 7scd | V82A:49 (2%)  E156G:49 (2%) | | V82A: 160 (0%)  V82A:22 (0%)  E156G:22 (0%) | | ND | [[179](#_ENREF_179), [192](#_ENREF_192)] | [[179](#_ENREF_179), [180](#_ENREF_180)] |
| SCLO1B1 N432D *7 | rs56387224 | 1294A>G | Asn432Asp | in vitro substrate-specific decrease | ND | 8 | N432D: 49 (1%) | | N432D: singleton | | N432D: 120 (0%) | [[179](#_ENREF_179), [192](#_ENREF_192)] | [[179](#_ENREF_179), [180](#_ENREF_180)] |
| SCLO1B1 D655G *10 (also in *12) | rs56199088 | 1964A>G | Asp655Gly | in vitro  substrate-specific  decrease | ND | 8 | D655G: 49 (2%) | | D655G: singleton | | D655G: 120 (0%) | [[179](#_ENREF_179), [192](#_ENREF_192)] | [[179](#_ENREF_179)] |
| SCLO1B1 N151S *16’ | rs2306282 | 452A>G | Asn151Ser | in vivo (PK)  decrease | insufficient data **^d^** | 10 | N151S: 49 (0%) | | N151S: 22 (0%) | | *16’: 120 (4%) | [[179](#_ENREF_179), [192](#_ENREF_192)] | [[192](#_ENREF_192)] |
| SCLO1B1 P336R  (in *15+P336R) | rs72559747 | 1007C>G | Pro336Arg | in vivo (PK)  decrease | pravastatin ↑ADR | 10 | P336R: 285 (0%) | | ND | | *15+P336R: 120 (2%) | [[192](#_ENREF_192), [196](#_ENREF_196), [209](#_ENREF_209)] | [[192](#_ENREF_192), [196](#_ENREF_196)] |
| SCLO1B1  -11187G>A (in 4 haplotypes) | rs4149014 | -11187 G>A | 5’ promoter SNP | in vivo  decrease | pravastatin ↑AUC ↓efficacy | 10 | *17’: (5%) *17”: 41 (4%)  *21: 41 (3%) | | ND | | *17’: (1%)  *17’’: 100 (8%)  *21: 100 (2%)  *21+L191L: (5%) | [[178](#_ENREF_178), [179](#_ENREF_179), [191](#_ENREF_191), [198](#_ENREF_198)] | [[178](#_ENREF_178)] |
| SCLO1B1*18’ (also in *18’+F199F) | rs2306283 rs59502379 | 388A>G 1463G>C | Asn130Asp +  Gly488Ala | in vitro decrease | ND | 11 | G488A  2454 (0%) | | G488A: 160 (5%), *18’+F199F: (3%) | | *18’+F199F: (<1%) | [[178](#_ENREF_178), [179](#_ENREF_179), [187](#_ENREF_187), [191](#_ENREF_191)] | [[180](#_ENREF_180)] |
| SCLO1B1*1C | no rsID  no rsID | 455G>A 721G>A | Arg152Lys +  Asp241Asn | in vitro substrate-specific decrease | ND | 12 | *1C: 49 (0%) | | *1C: 22 (0%) | | *1C: 267 (0%) | [[179](#_ENREF_179), [202](#_ENREF_202)] | [[179](#_ENREF_179), [180](#_ENREF_180), [202](#_ENREF_202)] |
| SCLO1B1 L193R | rs72559746 | 578T>G | **¦** Leu193Arg  null, retained intracellularly | in vitro decrease | ND | 12 | L193R: 330 (<1%) | | ND | | ND | [[210](#_ENREF_210)] | [[210](#_ENREF_210)] |
| SCLO1B1 L543W | no rsID | 1628T>G | **¦** Leu543Trp  in TMD ^(91=PM:15681900)^ | in vitro/ in vivo  decrease | pravastatin ↑ADR | 12 | L543W: 285 (0%) | | ND | | L543W: (<1%) | [[199](#_ENREF_199), [209](#_ENREF_209)] | [[199](#_ENREF_199), [200](#_ENREF_200)] |
| SCLO1B1 C485F | no rsID | 1454G>T | Cys485Phe | ND | ND | 13 | ND | | ND | | C485F: 120 (1%) | [[192](#_ENREF_192)] | ND |
| SCLO1B1 R580X | rs71581941 | 1738C>T | **¦** Arg580X  in SBS ^(93=PM:20821001)^ | ND | ND | 13 | R580X: 1000 (<1%) unknown ethnicity | | | | | dbSNP | [[201](#_ENREF_201)] |
| SCLO1B1 I211M | rs184348772 | 633A>G | **¦** Ile211Met  in TMD ^(93=PM:20821001)^ | ND | ND | 13 | ND | ND | | I211M: 96 (<1%) | | [[211](#_ENREF_211)] | [[211](#_ENREF_211)] |
| SCLO1B1 C613R | no rsID | 1837T>A | Cys613Arg | ND | ND | 13 | ND | ND | | C613R: 96 (1%) | | [[211](#_ENREF_211)] | [[211](#_ENREF_211)] |
| SCLO1B1 L626X | rs183624077 | 1877T>A | **¦** Leu626X in TMD ^(93=PM:20821001)^, truncated ^(103=PM:19122343)^ | ND | ND | 13 | ND | ND | | L626X: 96 (1%) | | [[211](#_ENREF_211)] | [[211](#_ENREF_211)] |
| SCLO1B1 F73L *2 (also in *12) | rs56101265 | 217T>C | **¦** Phe73Leu in TMD, ↓in vivo trafficking ^(75=PM:21178985)^, ↓expression | in vitro decrease | ↔ statin efficacy | 14 | F73L: 1454 (4%) | F73L: 160 (0%) | | F73L: 120 (0%)  *2: 267 (0%) | | [[179](#_ENREF_179), [187](#_ENREF_187), [192](#_ENREF_192), [202](#_ENREF_202)] | [[179](#_ENREF_179), [180](#_ENREF_180), [187](#_ENREF_187)] |
| SCLO1B1 N130D  (in 17 haplotypes) | rs2306283 | 388A>G | Asn130Asp | in vivo adult age-specific **^c^** increase | simvastatin ↔↑ADR, efficacy | 14 | N130D: 2454 (44%) *1B: 32 (8%) | N130D: 160 (74%) | | N130D: 96 (75%)  *1B: 267 (54%) | | [[182](#_ENREF_182), [187](#_ENREF_187), [191](#_ENREF_191), [202](#_ENREF_202), [211](#_ENREF_211)] | [[180](#_ENREF_180), [183](#_ENREF_183), [184](#_ENREF_184), [187](#_ENREF_187), [190](#_ENREF_190), [195](#_ENREF_195)] |
| SCLO1B1 P155T *4 (also in *14, *18’’) | rs11045819 | 463C>A | Pro155Thr | in vitro  normal | ↔ statin efficacy | 14 | P155T: 2454 (16%),  *18’’: 41 (7%) | P155T: 160 (10%) | | P155T: 36 (3%) | | [[178](#_ENREF_178), [187](#_ENREF_187), [212](#_ENREF_212)] | [[178-180](#_ENREF_178), [187](#_ENREF_187), [210](#_ENREF_210), [212](#_ENREF_212), [213](#_ENREF_213)] |
|  |  |  |  |  |  |  | *14: 136 (16%) Brazilians | | | | |  |  |
| SCLO1B1 D462G *8 | rs72559748 | 1385A>G | Asp462Gly | in vitro  normal | ND | 14 | D462G: 49 (1%) | D462G: 22 (0%) | | D462G: 120 (0%) | | [[179](#_ENREF_179), [192](#_ENREF_192)] | [[179](#_ENREF_179), [180](#_ENREF_180)] |
| SCLO1B1 G488A  *9 (also in *18’, *18’+F199F) | rs59502379 | 1463G>C | Gly488Ala | in vitro  decrease | pravastatin ↔ efficacy | 14 | G488A: 2454 (0%) | G488A: 160 (5%), *18’+F199F: (3%) | | G488A: 192 (<1%) | | [[179](#_ENREF_179), [187](#_ENREF_187), [191](#_ENREF_191)] | [[179](#_ENREF_179), [181](#_ENREF_181), [187](#_ENREF_187)] |
| SCLO1B1 L191L *1J (also in 5 haplotypes) | rs4149057 | 571T>C | Leu191Leu | in vivo (PK)  normal | ↔ statin PK | 14 | *1J: 41 (39%)  *1K: 41 (2%)  *1L: 41 (1%)  *18’’: 41 (7%)  *19: 41 (1%) | L191L: 132 (31%) | | L191L: 172 (36%) | | [[178](#_ENREF_178)] | [[178](#_ENREF_178)] |
| SCLO1B1 E667G *11 (also in *13) | rs55737008 | 2000A>G | Glu667Gly | in vitro  normal | ND | 14 | E667G: 49 (2%) | E667G: 22 (34%) | | ND | | [[179](#_ENREF_179)] | [[179](#_ENREF_179)] |
| SCLO1B1 F199F (in 8 haplotypes) | rs2291075 | 597C>T | Phe199Phe,  ↔expression^(90=PM:17415554)^ | normal PK | ↔ statin PK | 14 | *5B: 41 (1%) *18’’: 41 (7%) | 16’’’: (4%) *18’+F199F: (3%) | | F199F: 170 (52%),  *18’+F199F: (<1%) | | [[178](#_ENREF_178), [191](#_ENREF_191), [198](#_ENREF_198)] | [[178](#_ENREF_178), [198](#_ENREF_198)] |
| SCLO1B1 L643F (in 4 haplotypes) | rs34671512 | 1929A>C | Leu643Phe | in vitro  normal | ↔ statin PK/PD, efficacy | 14 | L643F: 2454 (6%)  *19: 41 (1%)  *20: 41 (4%)  *21: 41 (4%) | L643F: 160 (5%) | | L643F: 36 (1%) | | [[178](#_ENREF_178), [187](#_ENREF_187)] | [[178](#_ENREF_178), [183](#_ENREF_183), [187](#_ENREF_187), [209](#_ENREF_209)] |

ND = not determined/no data, H’ = haplotype, SNP = single nucleotide polymorphism, PK = pharmacokinetics, PD = pharmacodynamics, AUC = area under the concentration-time curve, ADR = adverse drug reaction, TMD = transmembrane domain, SBS = substrate binding site, **¦** = highly conserved sequence,

^a^ = evidence code assigned to each allele provides an indication of the strength and type of evidence for a drug-related phenotype and is based on the scheme presented in **Table 1** (green indicates clinically relevant evidence, yellow=potential clinical relevance, light orange= clinical relevance unknown, and dark orange= clinical relevance unsupported). For alleles with multiple types of supporting data, the code for the greatest strength of evidence is assigned,

^b^ = allele frequency from HapMap, dbSNP, or specific reference, SLCO1B1*1 allele frequency is estimated and takes into account the allele frequencies of other variants reported in this table with an evidence code ≤ 7

c = children exhibit the opposite activity phenotype from adults.

d = the impact of the *16’ allele is uncertain, as the only PK data comes from a carrier with the increased-activity *1B allele. As a diplotype, two *1B/*16’ carriers show similar but slightly reduced pravastatin clearance than two *1A/*1A carriers ^(84=PM:12811365)^.

**Table S28. Predicted risk of simvastatin-induced adverse reactions for “included” SLCO1B1 genotypes**

| **Allele (Frequency)** | **SLCO1B1 *1** | **SLCO1B1**  ***6** | **SLCO1B1 *3** | **SLCO1B1 V174A** |
| --- | --- | --- | --- | --- |
| **SLCO1B1 *1 (82%)** | *1/*1 (64%) normal uptake activity | *1/*6 (3%) intermediate decreased uptake activity | *1/*3  (3%) intermediate decreased uptake activity | *1/V174A  (26%) intermediate decreased uptake activity |
| **SLCO1B1 *6 (2%)** |  | *6/*6  (0.04%) decrease uptake activity | *6/*3  (0.08%) decrease uptake activity | *6/V174A  (0.6%) decrease uptake activity |
| **SLCO1B1**  ***3 (<0.01%)** |  |  | *3/*3  (0.04%) increase | *3/V174A  (0.6%) decrease uptake activity |
| **SLCO1B1  V174A (16%)** |  |  |  | V174A/V174A (3%) decrease uptake activity |

Approximate expected genotype frequencies have been estimated based upon reported allele frequencies for Caucasians and Hardy Weinberg principals. Genotypes are assigned a predicted clinical phenotype based on the transporter uptake activity predicted for individual alleles with statins or other substrates. Color shading relates to the expected likelihood of simvastatin-induced adverse reactions, such as elevated creatine kinase and myopathy based on published outcomes and general rules as defined: dark purple = increased risk of ADR, light purple = intermediate increase risk of ADR, blue = likely normal response.

**REFERENCES**

1. Mega JL, Close SL, Wiviott SD, Shen L, Hockett RD, Brandt JT, Walker JR, Antman EM, Macias W, Braunwald E, Sabatine MS: **Cytochrome p-450 polymorphisms and response to clopidogrel.** *The New England journal of medicine* 2009, **360:**354-362.

2. Simon T, Verstuyft C, Mary-Krause M, Quteineh L, Drouet E, Meneveau N, Steg PG, Ferrieres J, Danchin N, Becquemont L, et al: **Genetic determinants of response to clopidogrel and cardiovascular events.** *The New England journal of medicine* 2009, **360:**363-375.

3. Collet JP, Hulot JS, Pena A, Villard E, Esteve JB, Silvain J, Payot L, Brugier D, Cayla G, Beygui F, et al: **Cytochrome P450 2C19 polymorphism in young patients treated with clopidogrel after myocardial infarction: a cohort study.** *Lancet* 2009, **373:**309-317.

4. Sibbing D, Stegherr J, Latz W, Koch W, Mehilli J, Dorrler K, Morath T, Schomig A, Kastrati A, von Beckerath N: **Cytochrome P450 2C19 loss-of-function polymorphism and stent thrombosis following percutaneous coronary intervention.** *European heart journal* 2009, **30:**916-922.

5. Giusti B, Gori AM, Marcucci R, Saracini C, Sestini I, Paniccia R, Buonamici P, Antoniucci D, Abbate R, Gensini GF: **Relation of cytochrome P450 2C19 loss-of-function polymorphism to occurrence of drug-eluting coronary stent thrombosis.** *The American journal of cardiology* 2009, **103:**806-811.

6. Trenk D, Hochholzer W, Fromm MF, Chialda LE, Pahl A, Valina CM, Stratz C, Schmiebusch P, Bestehorn HP, Buttner HJ, Neumann FJ: **Cytochrome P450 2C19 681G>A polymorphism and high on-clopidogrel platelet reactivity associated with adverse 1-year clinical outcome of elective percutaneous coronary intervention with drug-eluting or bare-metal stents.** *Journal of the American College of Cardiology* 2008, **51:**1925-1934.

7. Sibbing D, Koch W, Gebhard D, Schuster T, Braun S, Stegherr J, Morath T, Schomig A, von Beckerath N, Kastrati A: **Cytochrome 2C19*17 allelic variant, platelet aggregation, bleeding events, and stent thrombosis in clopidogrel-treated patients with coronary stent placement.** *Circulation* 2010, **121:**512-518.

8. Pare G, Mehta SR, Yusuf S, Anand SS, Connolly SJ, Hirsh J, Simonsen K, Bhatt DL, Fox KA, Eikelboom JW: **Effects of CYP2C19 genotype on outcomes of clopidogrel treatment.** *The New England journal of medicine* 2010, **363:**1704-1714.

9. Tiroch KA, Sibbing D, Koch W, Roosen-Runge T, Mehilli J, Schomig A, Kastrati A: **Protective effect of the CYP2C19 *17 polymorphism with increased activation of clopidogrel on cardiovascular events.** *American heart journal* 2010, **160:**506-512.

10. Wallentin L, James S, Storey RF, Armstrong M, Barratt BJ, Horrow J, Husted S, Katus H, Steg PG, Shah SH, et al: **Effect of CYP2C19 and ABCB1 single nucleotide polymorphisms on outcomes of treatment with ticagrelor versus clopidogrel for acute coronary syndromes: a genetic substudy of the PLATO trial.** *Lancet* 2010, **376:**1320-1328.

11. de Morais SM, Wilkinson GR, Blaisdell J, Nakamura K, Meyer UA, Goldstein JA: **The major genetic defect responsible for the polymorphism of S-mephenytoin metabolism in humans.** *The Journal of biological chemistry* 1994, **269:**15419-15422.

12. Rogan PK, Svojanovsky S, Leeder JS: **Information theory-based analysis of CYP2C19, CYP2D6 and CYP3A5 splicing mutations.** *Pharmacogenetics* 2003, **13:**207-218.

13. Shuldiner AR, O'Connell JR, Bliden KP, Gandhi A, Ryan K, Horenstein RB, Damcott CM, Pakyz R, Tantry US, Gibson Q, et al: **Association of cytochrome P450 2C19 genotype with the antiplatelet effect and clinical efficacy of clopidogrel therapy.** *JAMA : the journal of the American Medical Association* 2009, **302:**849-857.

14. Varenhorst C, James S, Erlinge D, Brandt JT, Braun OO, Man M, Siegbahn A, Walker J, Wallentin L, Winters KJ, Close SL: **Genetic variation of CYP2C19 affects both pharmacokinetic and pharmacodynamic responses to clopidogrel but not prasugrel in aspirin-treated patients with coronary artery disease.** *European heart journal* 2009, **30:**1744-1752.

15. De Morais SM, Wilkinson GR, Blaisdell J, Meyer UA, Nakamura K, Goldstein JA: **Identification of a new genetic defect responsible for the polymorphism of (S)-mephenytoin metabolism in Japanese.** *Molecular pharmacology* 1994, **46:**594-598.

16. Chen BL, Zhang W, Li Q, Li YL, He YJ, Fan L, Wang LS, Liu ZQ, Zhou HH: **Inhibition of ADP-induced platelet aggregation by clopidogrel is related to CYP2C19 genetic polymorphisms.** *Clinical and experimental pharmacology & physiology* 2008, **35:**904-908.

17. Ferguson RJ, De Morais SM, Benhamou S, Bouchardy C, Blaisdell J, Ibeanu G, Wilkinson GR, Sarich TC, Wright JM, Dayer P, Goldstein JA: **A new genetic defect in human CYP2C19: mutation of the initiation codon is responsible for poor metabolism of S-mephenytoin.** *The Journal of pharmacology and experimental therapeutics* 1998, **284:**356-361.

18. Gladding P, Webster M, Zeng I, Farrell H, Stewart J, Ruygrok P, Ormiston J, El-Jack S, Armstrong G, Kay P, et al: **The pharmacogenetics and pharmacodynamics of clopidogrel response: an analysis from the PRINC (Plavix Response in Coronary Intervention) trial.** *JACC Cardiovascular interventions* 2008, **1:**620-627.

19. Wang H, An N, Wang H, Gao Y, Liu D, Bian T, Zhu J, Chen C: **Evaluation of the effects of 20 nonsynonymous single nucleotide polymorphisms of CYP2C19 on S-mephenytoin 4'-hydroxylation and omeprazole 5'-hydroxylation.** *Drug metabolism and disposition: the biological fate of chemicals* 2011, **39:**830-837.

20. Ibeanu GC, Blaisdell J, Ghanayem BI, Beyeler C, Benhamou S, Bouchardy C, Wilkinson GR, Dayer P, Daly AK, Goldstein JA: **An additional defective allele, CYP2C19*5, contributes to the S-mephenytoin poor metabolizer phenotype in Caucasians.** *Pharmacogenetics* 1998, **8:**129-135.

21. Xiao ZS, Goldstein JA, Xie HG, Blaisdell J, Wang W, Jiang CH, Yan FX, He N, Huang SL, Xu ZH, Zhou HH: **Differences in the incidence of the CYP2C19 polymorphism affecting the S-mephenytoin phenotype in Chinese Han and Bai populations and identification of a new rare CYP2C19 mutant allele.** *The Journal of pharmacology and experimental therapeutics* 1997, **281:**604-609.

22. Ibeanu GC, Blaisdell J, Ferguson RJ, Ghanayem BI, Brosen K, Benhamou S, Bouchardy C, Wilkinson GR, Dayer P, Goldstein JA: **A novel transversion in the intron 5 donor splice junction of CYP2C19 and a sequence polymorphism in exon 3 contribute to the poor metabolizer phenotype for the anticonvulsant drug S-mephenytoin.** *The Journal of pharmacology and experimental therapeutics* 1999, **290:**635-640.

23. Sim SC, Risinger C, Dahl ML, Aklillu E, Christensen M, Bertilsson L, Ingelman-Sundberg M: **A common novel CYP2C19 gene variant causes ultrarapid drug metabolism relevant for the drug response to proton pump inhibitors and antidepressants.** *Clinical pharmacology and therapeutics* 2006, **79:**103-113.

24. Ibeanu GC, Goldstein JA, Meyer U, Benhamou S, Bouchardy C, Dayer P, Ghanayem BI, Blaisdell J: **Identification of new human CYP2C19 alleles (CYP2C19*6 and CYP2C19*2B) in a Caucasian poor metabolizer of mephenytoin.** *The Journal of pharmacology and experimental therapeutics* 1998, **286:**1490-1495.

25. Blaisdell J, Mohrenweiser H, Jackson J, Ferguson S, Coulter S, Chanas B, Xi T, Ghanayem B, Goldstein JA: **Identification and functional characterization of new potentially defective alleles of human CYP2C19.** *Pharmacogenetics* 2002, **12:**703-711.

26. Hunfeld NG, Touw DJ, Mathot RA, Mulder PG, RH VANS, Kuipers EJ, Kooiman JC, Geus WP: **A comparison of the acid-inhibitory effects of esomeprazole and pantoprazole in relation to pharmacokinetics and CYP2C19 polymorphism.** *Alimentary pharmacology & therapeutics* 2010, **31:**150-159.

27. Furuta T, Shirai N, Watanabe F, Honda S, Takeuchi K, Iida T, Sato Y, Kajimura M, Futami H, Takayanagi S, et al: **Effect of cytochrome P4502C19 genotypic differences on cure rates for gastroesophageal reflux disease by lansoprazole.** *Clinical pharmacology and therapeutics* 2002, **72:**453-460.

28. Shirai N, Furuta T, Moriyama Y, Okochi H, Kobayashi K, Takashima M, Xiao F, Kosuge K, Nakagawa K, Hanai H, et al: **Effects of CYP2C19 genotypic differences in the metabolism of omeprazole and rabeprazole on intragastric pH.** *Alimentary pharmacology & therapeutics* 2001, **15:**1929-1937.

29. Sagar M, Tybring G, Dahl ML, Bertilsson L, Seensalu R: **Effects of omeprazole on intragastric pH and plasma gastrin are dependent on the CYP2C19 polymorphism.** *Gastroenterology* 2000, **119:**670-676.

30. Horai Y, Kimura M, Furuie H, Matsuguma K, Irie S, Koga Y, Nagahama T, Murakami M, Matsui T, Yao T, et al: **Pharmacodynamic effects and kinetic disposition of rabeprazole in relation to CYP2C19 genotypes.** *Alimentary pharmacology & therapeutics* 2001, **15:**793-803.

31. Furuta T, Ohashi K, Kosuge K, Zhao XJ, Takashima M, Kimura M, Nishimoto M, Hanai H, Kaneko E, Ishizaki T: **CYP2C19 genotype status and effect of omeprazole on intragastric pH in humans.** *Clinical pharmacology and therapeutics* 1999, **65:**552-561.

32. Baldwin RM, Ohlsson S, Pedersen RS, Mwinyi J, Ingelman-Sundberg M, Eliasson E, Bertilsson L: **Increased omeprazole metabolism in carriers of the CYP2C19*17 allele; a pharmacokinetic study in healthy volunteers.** *British journal of clinical pharmacology* 2008, **65:**767-774.

33. Hu YM, Mei Q, Xu XH, Hu XP, Hu NZ, Xu JM: **Pharmacodynamic and kinetic effect of rabeprazole on serum gastrin level in relation to CYP2C19 polymorphism in Chinese Hans.** *World journal of gastroenterology : WJG* 2006, **12:**4750-4753.

34. Take S, Mizuno M, Ishiki K, Nagahara Y, Yoshida T, Inaba T, Yamamoto K, Okada H, Yokota K, Oguma K, Shiratori Y: **Interleukin-1beta genetic polymorphism influences the effect of cytochrome P 2C19 genotype on the cure rate of 1-week triple therapy for Helicobacter pylori infection.** *The American journal of gastroenterology* 2003, **98:**2403-2408.

35. Gawronska-Szklarz B, Siuda A, Kurzawski M, Bielicki D, Marlicz W, Drozdzik M: **Effects of CYP2C19, MDR1, and interleukin 1-B gene variants on the eradication rate of Helicobacter pylori infection by triple therapy with pantoprazole, amoxicillin, and metronidazole.** *European journal of clinical pharmacology* 2010, **66:**681-687.

36. Kurzawski M, Gawronska-Szklarz B, Wrzesniewska J, Siuda A, Starzynska T, Drozdzik M: **Effect of CYP2C19*17 gene variant on Helicobacter pylori eradication in peptic ulcer patients.** *European journal of clinical pharmacology* 2006, **62:**877-880.

37. Rocha A, Coelho EB, Moussa SA, Lanchote VL: **Investigation of the in vivo activity of CYP3A in Brazilian volunteers: comparison of midazolam and omeprazole as drug markers.** *European journal of clinical pharmacology* 2008, **64:**901-906.

38. Si D, Wang J, Zhang Y, Zhong D, Zhou H: **Distribution of CYP2C9*13 allele in the Chinese Han and the long-range haplotype containing CYP2C9*13 and CYP2C19*2.** *Biopharmaceutics & drug disposition* 2012, **33:**342-345.

39. Wang Y, Zhang H, Meng L, Wang M, Yuan H, Ou N, Zhang H, Li Z, Shi R: **Influence of CYP2C19 on the relationship between pharmacokinetics and intragastric pH of omeprazole administered by successive intravenous infusions in Chinese healthy volunteers.** *European journal of clinical pharmacology* 2010, **66:**563-569.

40. Kearns GL, Leeder JS, Gaedigk A: **Impact of the CYP2C19*17 allele on the pharmacokinetics of omeprazole and pantoprazole in children: evidence for a differential effect.** *Drug metabolism and disposition: the biological fate of chemicals* 2010, **38:**894-897.

41. Chang M, Dahl ML, Tybring G, Gotharson E, Bertilsson L: **Use of omeprazole as a probe drug for CYP2C19 phenotype in Swedish Caucasians: comparison with S-mephenytoin hydroxylation phenotype and CYP2C19 genotype.** *Pharmacogenetics* 1995, **5:**358-363.

42. Martinez C, Blanco G, Ladero JM, Garcia-Martin E, Taxonera C, Gamito FG, Diaz-Rubio M, Agundez JA: **Genetic predisposition to acute gastrointestinal bleeding after NSAIDs use.** *British journal of pharmacology* 2004, **141:**205-208.

43. Stempak D, Bukaveckas BL, Linder M, Koren G, Baruchel S: **Cytochrome P450 2C9 genotype: impact on celecoxib safety and pharmacokinetics in a pediatric patient.** *Clinical pharmacology and therapeutics* 2005, **78:**309-310.

44. Pilotto A, Seripa D, Franceschi M, Scarcelli C, Colaizzo D, Grandone E, Niro V, Andriulli A, Leandro G, Di Mario F, Dallapiccola B: **Genetic susceptibility to nonsteroidal anti-inflammatory drug-related gastroduodenal bleeding: role of cytochrome P450 2C9 polymorphisms.** *Gastroenterology* 2007, **133:**465-471.

45. Chan AT, Zauber AG, Hsu M, Breazna A, Hunter DJ, Rosenstein RB, Eagle CJ, Hawk ET, Bertagnolli MM: **Cytochrome P450 2C9 variants influence response to celecoxib for prevention of colorectal adenoma.** *Gastroenterology* 2009, **136:**2127-2136 e2121.

46. Tang C, Shou M, Rushmore TH, Mei Q, Sandhu P, Woolf EJ, Rose MJ, Gelmann A, Greenberg HE, De Lepeleire I, et al: **In-vitro metabolism of celecoxib, a cyclooxygenase-2 inhibitor, by allelic variant forms of human liver microsomal cytochrome P450 2C9: correlation with CYP2C9 genotype and in-vivo pharmacokinetics.** *Pharmacogenetics* 2001, **11:**223-235.

47. Kirchheiner J, Stormer E, Meisel C, Steinbach N, Roots I, Brockmoller J: **Influence of CYP2C9 genetic polymorphisms on pharmacokinetics of celecoxib and its metabolites.** *Pharmacogenetics* 2003, **13:**473-480.

48. Sandberg M, Yasar U, Stromberg P, Hoog JO, Eliasson E: **Oxidation of celecoxib by polymorphic cytochrome P450 2C9 and alcohol dehydrogenase.** *British journal of clinical pharmacology* 2002, **54:**423-429.

49. Kusama M, Maeda K, Chiba K, Aoyama A, Sugiyama Y: **Prediction of the effects of genetic polymorphism on the pharmacokinetics of CYP2C9 substrates from in vitro data.** *Pharmaceutical research* 2009, **26:**822-835.

50. Lundblad MS, Ohlsson S, Johansson P, Lafolie P, Eliasson E: **Accumulation of celecoxib with a 7-fold higher drug exposure in individuals homozygous for CYP2C9*3.** *Clinical pharmacology and therapeutics* 2006, **79:**287-288.

51. Yu BN, Luo CH, Wang D, Wang A, Li Z, Zhang W, Mo W, Zhou HH: **CYP2C9 allele variants in Chinese hypertension patients and healthy controls.** *Clinica chimica acta; international journal of clinical chemistry* 2004, **348:**57-61.

52. Dickmann LJ, Rettie AE, Kneller MB, Kim RB, Wood AJ, Stein CM, Wilkinson GR, Schwarz UI: **Identification and functional characterization of a new CYP2C9 variant (CYP2C9*5) expressed among African Americans.** *Molecular pharmacology* 2001, **60:**382-387.

53. Allabi AC, Gala JL, Horsmans Y, Babaoglu MO, Bozkurt A, Heusterspreute M, Yasar U: **Functional impact of CYP2C95, CYP2C96, CYP2C98, and CYP2C911 in vivo among black Africans.** *Clinical pharmacology and therapeutics* 2004, **76:**113-118.

54. Allabi AC, Gala JL, Horsmans Y: **CYP2C9, CYP2C19, ABCB1 (MDR1) genetic polymorphisms and phenytoin metabolism in a Black Beninese population.** *Pharmacogenetics and genomics* 2005, **15:**779-786.

55. Blaisdell J, Jorge-Nebert LF, Coulter S, Ferguson SS, Lee SJ, Chanas B, Xi T, Mohrenweiser H, Ghanayem B, Goldstein JA: **Discovery of new potentially defective alleles of human CYP2C9.** *Pharmacogenetics* 2004, **14:**527-537.

56. Cavallari LH, Langaee TY, Momary KM, Shapiro NL, Nutescu EA, Coty WA, Viana MA, Patel SR, Johnson JA: **Genetic and clinical predictors of warfarin dose requirements in African Americans.** *Clinical pharmacology and therapeutics* 2010, **87:**459-464.

57. Kidd RS, Curry TB, Gallagher S, Edeki T, Blaisdell J, Goldstein JA: **Identification of a null allele of CYP2C9 in an African-American exhibiting toxicity to phenytoin.** *Pharmacogenetics* 2001, **11:**803-808.

58. Agundez JA, Martinez C, Perez-Sala D, Carballo M, Torres MJ, Garcia-Martin E: **Pharmacogenomics in aspirin intolerance.** *Current drug metabolism* 2009, **10:**998-1008.

59. Ieiri I, Tainaka H, Morita T, Hadama A, Mamiya K, Hayashibara M, Ninomiya H, Ohmori S, Kitada M, Tashiro N, et al: **Catalytic activity of three variants (Ile, Leu, and Thr) at amino acid residue 359 in human CYP2C9 gene and simultaneous detection using single-strand conformation polymorphism analysis.** *Therapeutic drug monitoring* 2000, **22:**237-244.

60. Scott SA, Jaremko M, Lubitz SA, Kornreich R, Halperin JL, Desnick RJ: **CYP2C9*8 is prevalent among African-Americans: implications for pharmacogenetic dosing.** *Pharmacogenomics* 2009, **10:**1243-1255.

61. Si D, Guo Y, Zhang Y, Yang L, Zhou H, Zhong D: **Identification of a novel variant CYP2C9 allele in Chinese.** *Pharmacogenetics* 2004, **14:**465-469.

62. Guo Y, Wang Y, Si D, Fawcett PJ, Zhong D, Zhou H: **Catalytic activities of human cytochrome P450 2C9*1, 2C9*3 and 2C9*13.** *Xenobiotica; the fate of foreign compounds in biological systems* 2005, **35:**853-861.

63. Guo Y, Zhang Y, Wang Y, Chen X, Si D, Zhong D, Fawcett JP, Zhou H: **Role of CYP2C9 and its variants (CYP2C9*3 and CYP2C9*13) in the metabolism of lornoxicam in humans.** *Drug metabolism and disposition: the biological fate of chemicals* 2005, **33:**749-753.

64. Zhao F, Loke C, Rankin SC, Guo JY, Lee HS, Wu TS, Tan T, Liu TC, Lu WL, Lim YT, et al: **Novel CYP2C9 genetic variants in Asian subjects and their influence on maintenance warfarin dose.** *Clinical pharmacology and therapeutics* 2004, **76:**210-219.

65. DeLozier TC, Lee SC, Coulter SJ, Goh BC, Goldstein JA: **Functional characterization of novel allelic variants of CYP2C9 recently discovered in southeast Asians.** *The Journal of pharmacology and experimental therapeutics* 2005, **315:**1085-1090.

66. Maekawa K, Fukushima-Uesaka H, Tohkin M, Hasegawa R, Kajio H, Kuzuya N, Yasuda K, Kawamoto M, Kamatani N, Suzuki K, et al: **Four novel defective alleles and comprehensive haplotype analysis of CYP2C9 in Japanese.** *Pharmacogenetics and genomics* 2006, **16:**497-514.

67. Imai J, Ieiri I, Mamiya K, Miyahara S, Furuumi H, Nanba E, Yamane M, Fukumaki Y, Ninomiya H, Tashiro N, et al: **Polymorphism of the cytochrome P450 (CYP) 2C9 gene in Japanese epileptic patients: genetic analysis of the CYP2C9 locus.** *Pharmacogenetics* 2000, **10:**85-89.

68. Lee CR, Goldstein JA, Pieper JA: **Cytochrome P450 2C9 polymorphisms: a comprehensive review of the in-vitro and human data.** *Pharmacogenetics* 2002, **12:**251-263.

69. Kirchheiner J, Brockmoller J: **Clinical consequences of cytochrome P450 2C9 polymorphisms.** *Clinical pharmacology and therapeutics* 2005, **77:**1-16.

70. Ho PC, Abbott FS, Zanger UM, Chang TK: **Influence of CYP2C9 genotypes on the formation of a hepatotoxic metabolite of valproic acid in human liver microsomes.** *The pharmacogenomics journal* 2003, **3:**335-342.

71. Takanashi K, Tainaka H, Kobayashi K, Yasumori T, Hosakawa M, Chiba K: **CYP2C9 Ile359 and Leu359 variants: enzyme kinetic study with seven substrates.** *Pharmacogenetics* 2000, **10:**95-104.

72. King BP, Khan TI, Aithal GP, Kamali F, Daly AK: **Upstream and coding region CYP2C9 polymorphisms: correlation with warfarin dose and metabolism.** *Pharmacogenetics* 2004, **14:**813-822.

73. Rieder MJ, Reiner AP, Gage BF, Nickerson DA, Eby CS, McLeod HL, Blough DK, Thummel KE, Veenstra DL, Rettie AE: **Effect of VKORC1 haplotypes on transcriptional regulation and warfarin dose.** *The New England journal of medicine* 2005, **352:**2285-2293.

74. Carlquist JF, Horne BD, Muhlestein JB, Lappe DL, Whiting BM, Kolek MJ, Clarke JL, James BC, Anderson JL: **Genotypes of the cytochrome p450 isoform, CYP2C9, and the vitamin K epoxide reductase complex subunit 1 conjointly determine stable warfarin dose: a prospective study.** *Journal of thrombosis and thrombolysis* 2006, **22:**191-197.

75. Geisen C, Watzka M, Sittinger K, Steffens M, Daugela L, Seifried E, Muller CR, Wienker TF, Oldenburg J: **VKORC1 haplotypes and their impact on the inter-individual and inter-ethnical variability of oral anticoagulation.** *Thrombosis and haemostasis* 2005, **94:**773-779.

76. Rost S, Fregin A, Ivaskevicius V, Conzelmann E, Hortnagel K, Pelz HJ, Lappegard K, Seifried E, Scharrer I, Tuddenham EG, et al: **Mutations in VKORC1 cause warfarin resistance and multiple coagulation factor deficiency type 2.** *Nature* 2004, **427:**537-541.

77. Oldenburg J, von Brederlow B, Fregin A, Rost S, Wolz W, Eberl W, Eber S, Lenz E, Schwaab R, Brackmann HH, et al: **Congenital deficiency of vitamin K dependent coagulation factors in two families presents as a genetic defect of the vitamin K-epoxide-reductase-complex.** *Thrombosis and haemostasis* 2000, **84:**937-941.

78. D'Andrea G, D'Ambrosio RL, Di Perna P, Chetta M, Santacroce R, Brancaccio V, Grandone E, Margaglione M: **A polymorphism in the VKORC1 gene is associated with an interindividual variability in the dose-anticoagulant effect of warfarin.** *Blood* 2005, **105:**645-649.

79. Stec DE, Roman RJ, Flasch A, Rieder MJ: **Functional polymorphism in human CYP4F2 decreases 20-HETE production.** *Physiological genomics* 2007, **30:**74-81.

80. McDonald MG, Rieder MJ, Nakano M, Hsia CK, Rettie AE: **CYP4F2 is a vitamin K1 oxidase: An explanation for altered warfarin dose in carriers of the V433M variant.** *Molecular pharmacology* 2009, **75:**1337-1346.

81. Caldwell MD, Awad T, Johnson JA, Gage BF, Falkowski M, Gardina P, Hubbard J, Turpaz Y, Langaee TY, Eby C, et al: **CYP4F2 genetic variant alters required warfarin dose.** *Blood* 2008, **111:**4106-4112.

82. Takeuchi F, McGinnis R, Bourgeois S, Barnes C, Eriksson N, Soranzo N, Whittaker P, Ranganath V, Kumanduri V, McLaren W, et al: **A genome-wide association study confirms VKORC1, CYP2C9, and CYP4F2 as principal genetic determinants of warfarin dose.** *PLoS genetics* 2009, **5:**e1000433.

83. Borgiani P, Ciccacci C, Forte V, Sirianni E, Novelli L, Bramanti P, Novelli G: **CYP4F2 genetic variant (rs2108622) significantly contributes to warfarin dosing variability in the Italian population.** *Pharmacogenomics* 2009, **10:**261-266.

84. Perez-Andreu V, Roldan V, Anton AI, Garcia-Barbera N, Corral J, Vicente V, Gonzalez-Conejero R: **Pharmacogenetic relevance of CYP4F2 V433M polymorphism on acenocoumarol therapy.** *Blood* 2009, **113:**4977-4979.

85. Teichert M, Eijgelsheim M, Rivadeneira F, Uitterlinden AG, van Schaik RH, Hofman A, De Smet PA, van Gelder T, Visser LE, Stricker BH: **A genome-wide association study of acenocoumarol maintenance dosage.** *Human molecular genetics* 2009, **18:**3758-3768.

86. **Coumadin Drug Label October 2011** [[[http://www.accessdata.fda.gov/drugsatfda_docs/label/2011/009218s107lbl.pdf]](http://www.accessdata.fda.gov/drugsatfda_docs/label/2011/009218s107lbl.pdf%5d)]

87. Gaedigk A, Simon SD, Pearce RE, Bradford LD, Kennedy MJ, Leeder JS: **The CYP2D6 activity score: translating genotype information into a qualitative measure of phenotype.** *Clinical pharmacology and therapeutics* 2008, **83:**234-242.

88. Eckhardt K, Li S, Ammon S, Schanzle G, Mikus G, Eichelbaum M: **Same incidence of adverse drug events after codeine administration irrespective of the genetically determined differences in morphine formation.** *Pain* 1998, **76:**27-33.

89. Lotsch J, Rohrbacher M, Schmidt H, Doehring A, Brockmoller J, Geisslinger G: **Can extremely low or high morphine formation from codeine be predicted prior to therapy initiation?** *Pain* 2009, **144:**119-124.

90. Molanaei H, Carrero JJ, Heimburger O, Nordfors L, Lindholm B, Stenvinkel P, Odar-Cederlof I, Bertilsson L: **Influence of the CYP2D6 polymorphism and hemodialysis on codeine disposition in patients with end-stage renal disease.** *European journal of clinical pharmacology* 2010, **66:**269-273.

91. Shord SS, Cavallari LH, Gao W, Jeong HY, Deyo K, Patel SR, Camp JR, Labott SM, Molokie RE: **The pharmacokinetics of codeine and its metabolites in Blacks with sickle cell disease.** *European journal of clinical pharmacology* 2009, **65:**651-658.

92. Stamer UM, Stuber F: **Codeine and tramadol analgesic efficacy and respiratory effects are influenced by CYP2D6 genotype.** *Anaesthesia* 2007, **62:**1294-1295; author reply 1295-1296.

93. Brousseau DC, McCarver DG, Drendel AL, Divakaran K, Panepinto JA: **The effect of CYP2D6 polymorphisms on the response to pain treatment for pediatric sickle cell pain crisis.** *The Journal of pediatrics* 2007, **150:**623-626.

94. Schenk PW, van Fessem MA, Verploegh-Van Rij S, Mathot RA, van Gelder T, Vulto AG, van Vliet M, Lindemans J, Bruijn JA, van Schaik RH: **Association of graded allele-specific changes in CYP2D6 function with imipramine dose requirement in a large group of depressed patients.** *Molecular psychiatry* 2008, **13:**597-605.

95. Bossa R, Chiericozzi M, Efstathiu G, Galatulas I, Ninci MA: **The effect of H2 receptor antagonists on neuromuscular transmission.** *In vivo* 1991, **5:**57-59.

96. Mrazek DA, Biernacka JM, O'Kane DJ, Black JL, Cunningham JM, Drews MS, Snyder KA, Stevens SR, Rush AJ, Weinshilboum RM: **CYP2C19 variation and citalopram response.** *Pharmacogenetics and genomics* 2011, **21:**1-9.

97. Lee SJ, Lee SS, Jung HJ, Kim HS, Park SJ, Yeo CW, Shin JG: **Discovery of novel functional variants and extensive evaluation of CYP2D6 genetic polymorphisms in Koreans.** *Drug metabolism and disposition: the biological fate of chemicals* 2009, **37:**1464-1470.

98. Sistonen J, Sajantila A, Lao O, Corander J, Barbujani G, Fuselli S: **CYP2D6 worldwide genetic variation shows high frequency of altered activity variants and no continental structure.** *Pharmacogenetics and genomics* 2007, **17:**93-101.

99. Lim HS, Ju Lee H, Seok Lee K, Sook Lee E, Jang IJ, Ro J: **Clinical implications of CYP2D6 genotypes predictive of tamoxifen pharmacokinetics in metastatic breast cancer.** *Journal of clinical oncology : official journal of the American Society of Clinical Oncology* 2007, **25:**3837-3845.

100. Sistonen J, Fuselli S, Palo JU, Chauhan N, Padh H, Sajantila A: **Pharmacogenetic variation at CYP2C9, CYP2C19, and CYP2D6 at global and microgeographic scales.** *Pharmacogenetics and genomics* 2009, **19:**170-179.

101. Gaedigk A, Blum M, Gaedigk R, Eichelbaum M, Meyer UA: **Deletion of the entire cytochrome P450 CYP2D6 gene as a cause of impaired drug metabolism in poor metabolizers of the debrisoquine/sparteine polymorphism.** *American journal of human genetics* 1991, **48:**943-950.

102. Wennerholm A, Johansson I, Hidestrand M, Bertilsson L, Gustafsson LL, Ingelman-Sundberg M: **Characterization of the CYP2D6*29 allele commonly present in a black Tanzanian population causing reduced catalytic activity.** *Pharmacogenetics* 2001, **11:**417-427.

103. Wennerholm A, Dandara C, Sayi J, Svensson JO, Abdi YA, Ingelman-Sundberg M, Bertilsson L, Hasler J, Gustafsson LL: **The African-specific CYP2D617 allele encodes an enzyme with changed substrate specificity.** *Clinical pharmacology and therapeutics* 2002, **71:**77-88.

104. Koren G, Cairns J, Chitayat D, Gaedigk A, Leeder SJ: **Pharmacogenetics of morphine poisoning in a breastfed neonate of a codeine-prescribed mother.** *Lancet* 2006, **368:**704.

105. Madadi P, Ross CJ, Hayden MR, Carleton BC, Gaedigk A, Leeder JS, Koren G: **Pharmacogenetics of neonatal opioid toxicity following maternal use of codeine during breastfeeding: a case-control study.** *Clinical pharmacology and therapeutics* 2009, **85:**31-35.

106. Kirchheiner J, Schmidt H, Tzvetkov M, Keulen JT, Lotsch J, Roots I, Brockmoller J: **Pharmacokinetics of codeine and its metabolite morphine in ultra-rapid metabolizers due to CYP2D6 duplication.** *The pharmacogenomics journal* 2007, **7:**257-265.

107. Stamer UM, Musshoff F, Kobilay M, Madea B, Hoeft A, Stuber F: **Concentrations of tramadol and O-desmethyltramadol enantiomers in different CYP2D6 genotypes.** *Clinical pharmacology and therapeutics* 2007, **82:**41-47.

108. Lovlie R, Daly AK, Molven A, Idle JR, Steen VM: **Ultrarapid metabolizers of debrisoquine: characterization and PCR-based detection of alleles with duplication of the CYP2D6 gene.** *FEBS letters* 1996, **392:**30-34.

109. Saxena R, Shaw GL, Relling MV, Frame JN, Moir DT, Evans WE, Caporaso N, Weiffenbach B: **Identification of a new variant CYP2D6 allele with a single base deletion in exon 3 and its association with the poor metabolizer phenotype.** *Human molecular genetics* 1994, **3:**923-926.

110. Peters EJ, Slager SL, Kraft JB, Jenkins GD, Reinalda MS, McGrath PJ, Hamilton SP: **Pharmacokinetic genes do not influence response or tolerance to citalopram in the STAR*D sample.** *PloS one* 2008, **3:**e1872.

111. Evert B, Griese EU, Eichelbaum M: **A missense mutation in exon 6 of the CYP2D6 gene leading to a histidine 324 to proline exchange is associated with the poor metabolizer phenotype of sparteine.** *Naunyn-Schmiedeberg's archives of pharmacology* 1994, **350:**434-439.

112. Kiyotani K, Shimizu M, Kumai T, Kamataki T, Kobayashi S, Yamazaki H: **Limited effects of frequent CYP2D6*36-*10 tandem duplication allele on in vivo dextromethorphan metabolism in a Japanese population.** *European journal of clinical pharmacology* 2010, **66:**1065-1068.

113. Xu Y, Sun Y, Yao L, Shi L, Wu Y, Ouyang T, Li J, Wang T, Fan Z, Fan T, et al: **Association between CYP2D6 *10 genotype and survival of breast cancer patients receiving tamoxifen treatment.** *Annals of oncology : official journal of the European Society for Medical Oncology / ESMO* 2008, **19:**1423-1429.

114. Schroth W, Goetz MP, Hamann U, Fasching PA, Schmidt M, Winter S, Fritz P, Simon W, Suman VJ, Ames MM, et al: **Association between CYP2D6 polymorphisms and outcomes among women with early stage breast cancer treated with tamoxifen.** *JAMA : the journal of the American Medical Association* 2009, **302:**1429-1436.

115. Schroth W, Antoniadou L, Fritz P, Schwab M, Muerdter T, Zanger UM, Simon W, Eichelbaum M, Brauch H: **Breast cancer treatment outcome with adjuvant tamoxifen relative to patient CYP2D6 and CYP2C19 genotypes.** *Journal of clinical oncology : official journal of the American Society of Clinical Oncology* 2007, **25:**5187-5193.

116. Kiyotani K, Mushiroda T, Imamura CK, Hosono N, Tsunoda T, Kubo M, Tanigawara Y, Flockhart DA, Desta Z, Skaar TC, et al: **Significant effect of polymorphisms in CYP2D6 and ABCC2 on clinical outcomes of adjuvant tamoxifen therapy for breast cancer patients.** *Journal of clinical oncology : official journal of the American Society of Clinical Oncology* 2010, **28:**1287-1293.

117. Sakuyama K, Sasaki T, Ujiie S, Obata K, Mizugaki M, Ishikawa M, Hiratsuka M: **Functional characterization of 17 CYP2D6 allelic variants (CYP2D6.2, 10, 14A-B, 18, 27, 36, 39, 47-51, 53-55, and 57).** *Drug metabolism and disposition: the biological fate of chemicals* 2008, **36:**2460-2467.

118. Cai WM, Chen B, Zhang WX: **Frequency of CYP2D6*10 and *14 alleles and their influence on the metabolic activity of CYP2D6 in a healthy Chinese population.** *Clinical pharmacology and therapeutics* 2007, **81:**95-98.

119. Marez D, Legrand M, Sabbagh N, Lo Guidice JM, Spire C, Lafitte JJ, Meyer UA, Broly F: **Polymorphism of the cytochrome P450 CYP2D6 gene in a European population: characterization of 48 mutations and 53 alleles, their frequencies and evolution.** *Pharmacogenetics* 1997, **7:**193-202.

120. Gaedigk A, Bradford LD, Marcucci KA, Leeder JS: **Unique CYP2D6 activity distribution and genotype-phenotype discordance in black Americans.** *Clinical pharmacology and therapeutics* 2002, **72:**76-89.

121. Wang SL, Lai MD, Huang JD: **G169R mutation diminishes the metabolic activity of CYP2D6 in Chinese.** *Drug metabolism and disposition: the biological fate of chemicals* 1999, **27:**385-388.

122. Kiyotani K, Mushiroda T, Sasa M, Bando Y, Sumitomo I, Hosono N, Kubo M, Nakamura Y, Zembutsu H: **Impact of CYP2D6*10 on recurrence-free survival in breast cancer patients receiving adjuvant tamoxifen therapy.** *Cancer science* 2008, **99:**995-999.

123. Yamazaki H, Kiyotani K, Tsubuko S, Matsunaga M, Fujieda M, Saito T, Miura J, Kobayashi S, Kamataki T: **Two novel haplotypes of CYP2D6 gene in a Japanese population.** *Drug metabolism and pharmacokinetics* 2003, **18:**269-271.

124. Li L, Pan RM, Porter TD, Jensen NS, Silber P, Russo G, Tine JA, Heim J, Ring B, Wedlund PJ: **New cytochrome P450 2D6*56 allele identified by genotype/phenotype analysis of cryopreserved human hepatocytes.** *Drug metabolism and disposition: the biological fate of chemicals* 2006, **34:**1411-1416.

125. Chida M, Yokoi T, Nemoto N, Inaba M, Kinoshita M, Kamataki T: **A new variant CYP2D6 allele (CYP2D6*21) with a single base insertion in exon 5 in a Japanese population associated with a poor metabolizer phenotype.** *Pharmacogenetics* 1999, **9:**287-293.

126. Toscano C, Klein K, Blievernicht J, Schaeffeler E, Saussele T, Raimundo S, Eichelbaum M, Schwab M, Zanger UM: **Impaired expression of CYP2D6 in intermediate metabolizers carrying the *41 allele caused by the intronic SNP 2988G>A: evidence for modulation of splicing events.** *Pharmacogenetics and genomics* 2006, **16:**755-766.

127. Newman WG, Hadfield KD, Latif A, Roberts SA, Shenton A, McHague C, Lalloo F, Howell S, Evans DG: **Impaired tamoxifen metabolism reduces survival in familial breast cancer patients.** *Clinical cancer research : an official journal of the American Association for Cancer Research* 2008, **14:**5913-5918.

128. Tyndale R, Aoyama T, Broly F, Matsunaga T, Inaba T, Kalow W, Gelboin HV, Meyer UA, Gonzalez FJ: **Identification of a new variant CYP2D6 allele lacking the codon encoding Lys-281: possible association with the poor metabolizer phenotype.** *Pharmacogenetics* 1991, **1:**26-32.

129. Masimirembwa C, Persson I, Bertilsson L, Hasler J, Ingelman-Sundberg M: **A novel mutant variant of the CYP2D6 gene (CYP2D6*17) common in a black African population: association with diminished debrisoquine hydroxylase activity.** *British journal of clinical pharmacology* 1996, **42:**713-719.

130. Oscarson M, Hidestrand M, Johansson I, Ingelman-Sundberg M: **A combination of mutations in the CYP2D6*17 (CYP2D6Z) allele causes alterations in enzyme function.** *Molecular pharmacology* 1997, **52:**1034-1040.

131. Gaedigk A, Bradford LD, Alander SW, Leeder JS: **CYP2D6*36 gene arrangements within the cyp2d6 locus: association of CYP2D6*36 with poor metabolizer status.** *Drug metabolism and disposition: the biological fate of chemicals* 2006, **34:**563-569.

132. Chida M, Ariyoshi N, Yokoi T, Nemoto N, Inaba M, Kinoshita M, Kamataki T: **New allelic arrangement CYP2D6*36 x 2 found in a Japanese poor metabolizer of debrisoquine.** *Pharmacogenetics* 2002, **12:**659-662.

133. Fukuda T, Nishida Y, Imaoka S, Hiroi T, Naohara M, Funae Y, Azuma J: **The decreased in vivo clearance of CYP2D6 substrates by CYP2D6*10 might be caused not only by the low-expression but also by low affinity of CYP2D6.** *Archives of biochemistry and biophysics* 2000, **380:**303-308.

134. Niwa T, Hiroi T, Tsuzuki D, Yamamoto S, Narimatsu S, Fukuda T, Azuma J, Funae Y: **Effect of genetic polymorphism on the metabolism of endogenous neuroactive substances, progesterone and p-tyramine, catalyzed by CYP2D6.** *Brain research Molecular brain research* 2004, **129:**117-123.

135. Gaedigk A, Ryder DL, Bradford LD, Leeder JS: **CYP2D6 poor metabolizer status can be ruled out by a single genotyping assay for the -1584G promoter polymorphism.** *Clinical chemistry* 2003, **49:**1008-1011.

136. Gaedigk A, Ndjountche L, Gaedigk R, Leeder JS, Bradford LD: **Discovery of a novel nonfunctional cytochrome P450 2D6 allele, CYP2D642, in African American subjects.** *Clinical pharmacology and therapeutics* 2003, **73:**575-576.

137. Ebisawa A, Hiratsuka M, Sakuyama K, Konno Y, Sasaki T, Mizugaki M: **Two novel single nucleotide polymorphisms (SNPs) of the CYP2D6 gene in Japanese individuals.** *Drug metabolism and pharmacokinetics* 2005, **20:**294-299.

138. Gaedigk A, Eklund JD, Pearce RE, Leeder JS, Alander SW, Phillips MS, Bradford LD, Kennedy MJ: **Identification and characterization of CYP2D6*56B, an allele associated with the poor metabolizer phenotype.** *Clinical pharmacology and therapeutics* 2007, **81:**817-820.

139. Yokoi T, Kosaka Y, Chida M, Chiba K, Nakamura H, Ishizaki T, Kinoshita M, Sato K, Gonzalez FJ, Kamataki T: **A new CYP2D6 allele with a nine base insertion in exon 9 in a Japanese population associated with poor metabolizer phenotype.** *Pharmacogenetics* 1996, **6:**395-401.

140. Kubota T, Yamaura Y, Ohkawa N, Hara H, Chiba K: **Frequencies of CYP2D6 mutant alleles in a normal Japanese population and metabolic activity of dextromethorphan O-demethylation in different CYP2D6 genotypes.** *British journal of clinical pharmacology* 2000, **50:**31-34.

141. Marez D, Legrand M, Sabbagh N, Lo-Guidice JM, Boone P, Broly F: **An additional allelic variant of the CYP2D6 gene causing impaired metabolism of sparteine.** *Human genetics* 1996, **97:**668-670.

142. Sachse C, Brockmoller J, Bauer S, Reum T, Roots I: **A rare insertion of T226 in exon 1 of CYP2D6 causes a frameshift and is associated with the poor metabolizer phenotype: CYP2D6*15.** *Pharmacogenetics* 1996, **6:**269-272.

143. Broly F, Marez D, Lo Guidice JM, Sabbagh N, Legrand M, Boone P, Meyer UA: **A nonsense mutation in the cytochrome P450 CYP2D6 gene identified in a Caucasian with an enzyme deficiency.** *Human genetics* 1995, **96:**601-603.

144. Marez D, Sabbagh N, Legrand M, Lo-Guidice JM, Boone P, Broly F: **A novel CYP2D6 allele with an abolished splice recognition site associated with the poor metabolizer phenotype.** *Pharmacogenetics* 1995, **5:**305-311.

145. Johnson AD, Zhang Y, Papp AC, Pinsonneault JK, Lim JE, Saffen D, Dai Z, Wang D, Sadee W: **Polymorphisms affecting gene transcription and mRNA processing in pharmacogenetic candidate genes: detection through allelic expression imbalance in human target tissues.** *Pharmacogenetics and genomics* 2008, **18:**781-791.

146. Marez-Allorge D, Ellis SW, Lo Guidice JM, Tucker GT, Broly F: **A rare G2061 insertion affecting the open reading frame of CYP2D6 and responsible for the poor metabolizer phenotype.** *Pharmacogenetics* 1999, **9:**393-396.

147. Leathart JB, London SJ, Steward A, Adams JD, Idle JR, Daly AK: **CYP2D6 phenotype-genotype relationships in African-Americans and Caucasians in Los Angeles.** *Pharmacogenetics* 1998, **8:**529-541.

148. Gaedigk A, Coetsee C: **The CYP2D6 gene locus in South African Coloureds: unique allele distributions, novel alleles and gene arrangements.** *European journal of clinical pharmacology* 2008, **64:**465-475.

149. Black AJ, McLeod HL, Capell HA, Powrie RH, Matowe LK, Pritchard SC, Collie-Duguid ES, Reid DM: **Thiopurine methyltransferase genotype predicts therapy-limiting severe toxicity from azathioprine.** *Ann Intern Med* 1998, **129:**716-718.

150. Ansari A, Hassan C, Duley J, Marinaki A, Shobowale-Bakre EM, Seed P, Meenan J, Yim A, Sanderson J: **Thiopurine methyltransferase activity and the use of azathioprine in inflammatory bowel disease.** *Alimentary pharmacology & therapeutics* 2002, **16:**1743-1750.

151. Formea CM, Myers-Huentelman H, Wu R, Crabtree J, Fujita S, Hemming A, Reed A, Howard R, Karlix JL: **Thiopurine S-methyltransferase genotype predicts azathioprine-induced myelotoxicity in kidney transplant recipients.** *American journal of transplantation : official journal of the American Society of Transplantation and the American Society of Transplant Surgeons* 2004, **4:**1810-1817.

152. Dokmanovic L, Urosevic J, Janic D, Jovanovic N, Petrucev B, Tosic N, Pavlovic S: **Analysis of thiopurine S-methyltransferase polymorphism in the population of Serbia and Montenegro and mercaptopurine therapy tolerance in childhood acute lymphoblastic leukemia.** *Therapeutic drug monitoring* 2006, **28:**800-806.

153. Ansari A, Arenas M, Greenfield SM, Morris D, Lindsay J, Gilshenan K, Smith M, Lewis C, Marinaki A, Duley J, Sanderson J: **Prospective evaluation of the pharmacogenetics of azathioprine in the treatment of inflammatory bowel disease.** *Alimentary pharmacology & therapeutics* 2008, **28:**973-983.

154. Vannaprasaht S, Angsuthum S, Avihingsanon Y, Sirivongs D, Pongskul C, Makarawate P, Praditpornsilpa K, Tassaneeyakul W: **Impact of the heterozygous TPMT*1/*3C genotype on azathioprine-induced myelosuppression in kidney transplant recipients in Thailand.** *Clin Ther* 2009, **31:**1524-1533.

155. Krynetski EY, Schuetz JD, Galpin AJ, Pui CH, Relling MV, Evans WE: **A single point mutation leading to loss of catalytic activity in human thiopurine S-methyltransferase.** *Proceedings of the National Academy of Sciences of the United States of America* 1995, **92:**949-953.

156. Evans WE, Horner M, Chu YQ, Kalwinsky D, Roberts WM: **Altered mercaptopurine metabolism, toxic effects, and dosage requirement in a thiopurine methyltransferase-deficient child with acute lymphocytic leukemia.** *The Journal of pediatrics* 1991, **119:**985-989.

157. Salavaggione OE, Wang L, Wiepert M, Yee VC, Weinshilboum RM: **Thiopurine S-methyltransferase pharmacogenetics: variant allele functional and comparative genomics.** *Pharmacogenetics and genomics* 2005, **15:**801-815.

158. Szumlanski C, Otterness D, Her C, Lee D, Brandriff B, Kelsell D, Spurr N, Lennard L, Wieben E, Weinshilboum R: **Thiopurine methyltransferase pharmacogenetics: human gene cloning and characterization of a common polymorphism.** *DNA Cell Biol* 1996, **15:**17-30.

159. Larovere LE, de Kremer RD, Lambooy LH, De Abreu RA: **Genetic polymorphism of thiopurine S-methyltransferase in Argentina.** *Ann Clin Biochem* 2003, **40:**388-393.

160. Hiratsuka M, Inoue T, Omori F, Agatsuma Y, Kishikawa Y, Mizugaki M: **Detection assay of rare variants of the thiopurine methyltransferase gene by PCR-RFLP using a mismatch primer in a Japanese population.** *Biological & pharmaceutical bulletin* 2000, **23:**1090-1093.

161. Otterness DM, Szumlanski CL, Wood TC, Weinshilboum RM: **Human thiopurine methyltransferase pharmacogenetics. Kindred with a terminal exon splice junction mutation that results in loss of activity.** *J Clin Invest* 1998, **101:**1036-1044.

162. Roberts RL, Gearry RB, Bland MV, Sies CW, George PM, Burt M, Marinaki AM, Arenas M, Barclay ML, Kennedy MA: **Trinucleotide repeat variants in the promoter of the thiopurine S-methyltransferase gene of patients exhibiting ultra-high enzyme activity.** *Pharmacogenetics and genomics* 2008, **18:**434-438.

163. Otterness D, Szumlanski C, Lennard L, Klemetsdal B, Aarbakke J, Park-Hah JO, Iven H, Schmiegelow K, Branum E, O'Brien J, Weinshilboum R: **Human thiopurine methyltransferase pharmacogenetics: gene sequence polymorphisms.** *Clinical pharmacology and therapeutics* 1997, **62:**60-73.

164. Schaeffeler E, Fischer C, Brockmeier D, Wernet D, Moerike K, Eichelbaum M, Zanger UM, Schwab M: **Comprehensive analysis of thiopurine S-methyltransferase phenotype-genotype correlation in a large population of German-Caucasians and identification of novel TPMT variants.** *Pharmacogenetics* 2004, **14:**407-417.

165. Schaeffeler E, Zanger UM, Eichelbaum M, Asante-Poku S, Shin JG, Schwab M: **Highly multiplexed genotyping of thiopurine s-methyltransferase variants using MALD-TOF mass spectrometry: reliable genotyping in different ethnic groups.** *Clinical chemistry* 2008, **54:**1637-1647.

166. Lee SS, Kim WY, Jang YJ, Shin JG: **Duplex pyrosequencing of the TPMT*3C and TPMT*6 alleles in Korean and Vietnamese populations.** *Clinica chimica acta; international journal of clinical chemistry* 2008, **398:**82-85.

167. Hamdan-Khalil R, Allorge D, Lo-Guidice JM, Cauffiez C, Chevalier D, Spire C, Houdret N, Libersa C, Lhermitte M, Colombel JF, et al: **In vitro characterization of four novel non-functional variants of the thiopurine S-methyltransferase.** *Biochem Biophys Res Commun* 2003, **309:**1005-1010.

168. Spire-Vayron de la Moureyre C, Debuysere H, Sabbagh N, Marez D, Vinner E, Chevalier ED, Lo Guidice JM, Broly F: **Detection of known and new mutations in the thiopurine S-methyltransferase gene by single-strand conformation polymorphism analysis.** *Hum Mutat* 1998, **12:**177-185.

169. Ujiie S, Sasaki T, Mizugaki M, Ishikawa M, Hiratsuka M: **Functional characterization of 23 allelic variants of thiopurine S-methyltransferase gene (TPMT*2 - *24).** *Pharmacogenetics and genomics* 2008, **18:**887-893.

170. Schaeffeler E, Stanulla M, Greil J, Schrappe M, Eichelbaum M, Zanger UM, Schwab M: **A novel TPMT missense mutation associated with TPMT deficiency in a 5-year-old boy with ALL.** *Leukemia* 2003, **17:**1422-1424.

171. Lindqvist M, Haglund S, Almer S, Peterson C, Taipalensu J, Hertervig E, Lyrenas E, Soderkvist P: **Identification of two novel sequence variants affecting thiopurine methyltransferase enzyme activity.** *Pharmacogenetics* 2004, **14:**261-265.

172. Hamdan-Khalil R, Gala JL, Allorge D, Lo-Guidice JM, Horsmans Y, Houdret N, Broly F: **Identification and functional analysis of two rare allelic variants of the thiopurine S-methyltransferase gene, TPMT*16 and TPMT*19.** *Biochem Pharmacol* 2005, **69:**525-529.

173. Sasaki T, Goto E, Konno Y, Hiratsuka M, Mizugaki M: **Three novel single nucleotide polymorphisms of the human thiopurine S-methyltransferase gene in Japanese individuals.** *Drug metabolism and pharmacokinetics* 2006, **21:**332-336.

174. Schaeffeler E, Eichelbaum M, Reinisch W, Zanger UM, Schwab M: **Three novel thiopurine S-methyltransferase allelic variants (TPMT*20, *21, *22) - association with decreased enzyme function.** *Hum Mutat* 2006, **27:**976.

175. Garat A, Cauffiez C, Renault N, Lo-Guidice JM, Allorge D, Chevalier D, Houdret N, Chavatte P, Loriot MA, Gala JL, Broly F: **Characterisation of novel defective thiopurine S-methyltransferase allelic variants.** *Biochem Pharmacol* 2008, **76:**404-415.

176. Lindqvist M, Skoglund K, Karlgren A, Soderkvist P, Peterson C, Kidhall I, Almer S: **Explaining TPMT genotype/phenotype discrepancy by haplotyping of TPMT*3A and identification of a novel sequence variant, TPMT*23.** *Pharmacogenetics and genomics* 2007, **17:**891-895.

177. Hon YY, Fessing MY, Pui CH, Relling MV, Krynetski EY, Evans WE: **Polymorphism of the thiopurine S-methyltransferase gene in African-Americans.** *Human molecular genetics* 1999, **8:**371-376.

178. Niemi M, Schaeffeler E, Lang T, Fromm MF, Neuvonen M, Kyrklund C, Backman JT, Kerb R, Schwab M, Neuvonen PJ, et al: **High plasma pravastatin concentrations are associated with single nucleotide polymorphisms and haplotypes of organic anion transporting polypeptide-C (OATP-C, SLCO1B1).** *Pharmacogenetics* 2004, **14:**429-440.

179. Tirona RG, Leake BF, Merino G, Kim RB: **Polymorphisms in OATP-C: identification of multiple allelic variants associated with altered transport activity among European- and African-Americans.** *The Journal of biological chemistry* 2001, **276:**35669-35675.

180. Ho RH, Tirona RG, Leake BF, Glaeser H, Lee W, Lemke CJ, Wang Y, Kim RB: **Drug and bile acid transporters in rosuvastatin hepatic uptake: function, expression, and pharmacogenetics.** *Gastroenterology* 2006, **130:**1793-1806.

181. Ho RH, Choi L, Lee W, Mayo G, Schwarz UI, Tirona RG, Bailey DG, Michael Stein C, Kim RB: **Effect of drug transporter genotypes on pravastatin disposition in European- and African-American participants.** *Pharmacogenetics and genomics* 2007, **17:**647-656.

182. Pasanen MK, Neuvonen M, Neuvonen PJ, Niemi M: **SLCO1B1 polymorphism markedly affects the pharmacokinetics of simvastatin acid.** *Pharmacogenetics and genomics* 2006, **16:**873-879.

183. Group SC, Link E, Parish S, Armitage J, Bowman L, Heath S, Matsuda F, Gut I, Lathrop M, Collins R: **SLCO1B1 variants and statin-induced myopathy--a genomewide study.** *The New England journal of medicine* 2008, **359:**789-799.

184. Donnelly LA, Doney AS, Tavendale R, Lang CC, Pearson ER, Colhoun HM, McCarthy MI, Hattersley AT, Morris AD, Palmer CN: **Common nonsynonymous substitutions in SLCO1B1 predispose to statin intolerance in routinely treated individuals with type 2 diabetes: a go-DARTS study.** *Clinical pharmacology and therapeutics* 2011, **89:**210-216.

185. Voora D, Shah SH, Spasojevic I, Ali S, Reed CR, Salisbury BA, Ginsburg GS: **The SLCO1B1*5 genetic variant is associated with statin-induced side effects.** *Journal of the American College of Cardiology* 2009, **54:**1609-1616.

186. Bailey KM, Romaine SP, Jackson BM, Farrin AJ, Efthymiou M, Barth JH, Copeland J, McCormack T, Whitehead A, Flather MD, et al: **Hepatic metabolism and transporter gene variants enhance response to rosuvastatin in patients with acute myocardial infarction: the GEOSTAT-1 Study.** *Circulation Cardiovascular genetics* 2010, **3:**276-285.

187. Thompson JF, Man M, Johnson KJ, Wood LS, Lira ME, Lloyd DB, Banerjee P, Milos PM, Myrand SP, Paulauskis J, et al: **An association study of 43 SNPs in 16 candidate genes with atorvastatin response.** *The pharmacogenomics journal* 2005, **5:**352-358.

188. Peters BJ, Rodin AS, Klungel OH, van Duijn CM, Stricker BH, van't Slot R, de Boer A, Maitland-van der Zee AH: **Pharmacogenetic interactions between ABCB1 and SLCO1B1 tagging SNPs and the effectiveness of statins in the prevention of myocardial infarction.** *Pharmacogenomics* 2010, **11:**1065-1076.

189. Tachibana-Iimori R, Tabara Y, Kusuhara H, Kohara K, Kawamoto R, Nakura J, Tokunaga K, Kondo I, Sugiyama Y, Miki T: **Effect of genetic polymorphism of OATP-C (SLCO1B1) on lipid-lowering response to HMG-CoA reductase inhibitors.** *Drug metabolism and pharmacokinetics* 2004, **19:**375-380.

190. Mwinyi J, Johne A, Bauer S, Roots I, Gerloff T: **Evidence for inverse effects of OATP-C (SLC21A6) 5 and 1b haplotypes on pravastatin kinetics.** *Clinical pharmacology and therapeutics* 2004, **75:**415-421.

191. Pasanen MK, Neuvonen PJ, Niemi M: **Global analysis of genetic variation in SLCO1B1.** *Pharmacogenomics* 2008, **9:**19-33.

192. Nishizato Y, Ieiri I, Suzuki H, Kimura M, Kawabata K, Hirota T, Takane H, Irie S, Kusuhara H, Urasaki Y, et al: **Polymorphisms of OATP-C (SLC21A6) and OAT3 (SLC22A8) genes: consequences for pravastatin pharmacokinetics.** *Clinical pharmacology and therapeutics* 2003, **73:**554-565.

193. Deng JW, Song IS, Shin HJ, Yeo CW, Cho DY, Shon JH, Shin JG: **The effect of SLCO1B1*15 on the disposition of pravastatin and pitavastatin is substrate dependent: the contribution of transporting activity changes by SLCO1B1*15.** *Pharmacogenetics and genomics* 2008, **18:**424-433.

194. Pasanen MK, Backman JT, Neuvonen PJ, Niemi M: **Frequencies of single nucleotide polymorphisms and haplotypes of organic anion transporting polypeptide 1B1 SLCO1B1 gene in a Finnish population.** *European journal of clinical pharmacology* 2006, **62:**409-415.

195. Kivisto KT, Niemi M: **Influence of drug transporter polymorphisms on pravastatin pharmacokinetics in humans.** *Pharmaceutical research* 2007, **24:**239-247.

196. Kameyama Y, Yamashita K, Kobayashi K, Hosokawa M, Chiba K: **Functional characterization of SLCO1B1 (OATP-C) variants, SLCO1B1*5, SLCO1B1*15 and SLCO1B1*15+C1007G, by using transient expression systems of HeLa and HEK293 cells.** *Pharmacogenetics and genomics* 2005, **15:**513-522.

197. Niemi M, Neuvonen PJ, Hofmann U, Backman JT, Schwab M, Lutjohann D, von Bergmann K, Eichelbaum M, Kivisto KT: **Acute effects of pravastatin on cholesterol synthesis are associated with SLCO1B1 (encoding OATP1B1) haplotype *17.** *Pharmacogenetics and genomics* 2005, **15:**303-309.

198. Jada SR, Xiaochen S, Yan LY, Xiaoqiang X, Lal S, Zhou SF, Ooi LL, Chowbay B: **Pharmacogenetics of SLCO1B1: haplotypes, htSNPs and hepatic expression in three distinct Asian populations.** *European journal of clinical pharmacology* 2007, **63:**555-563.

199. Morimoto K, Oishi T, Ueda S, Ueda M, Hosokawa M, Chiba K: **A novel variant allele of OATP-C (SLCO1B1) found in a Japanese patient with pravastatin-induced myopathy.** *Drug metabolism and pharmacokinetics* 2004, **19:**453-455.

200. Furihata T, Satoh N, Ohishi T, Ugajin M, Kameyama Y, Morimoto K, Matsumoto S, Yamashita K, Kobayashi K, Chiba K: **Functional analysis of a mutation in the SLCO1B1 gene (c.1628T>G) identified in a Japanese patient with pravastatin-induced myopathy.** *The pharmacogenomics journal* 2009, **9:**185-193.

201. Weaver YM, Hagenbuch B: **Several conserved positively charged amino acids in OATP1B1 are involved in binding or translocation of different substrates.** *The Journal of membrane biology* 2010, **236:**279-290.

202. Nozawa T, Nakajima M, Tamai I, Noda K, Nezu J, Sai Y, Tsuji A, Yokoi T: **Genetic polymorphisms of human organic anion transporters OATP-C (SLC21A6) and OATP-B (SLC21A9): allele frequencies in the Japanese population and functional analysis.** *The Journal of pharmacology and experimental therapeutics* 2002, **302:**804-813.

203. Lee E, Ryan S, Birmingham B, Zalikowski J, March R, Ambrose H, Moore R, Lee C, Chen Y, Schneck D: **Rosuvastatin pharmacokinetics and pharmacogenetics in white and Asian subjects residing in the same environment.** *Clinical pharmacology and therapeutics* 2005, **78:**330-341.

204. Gerloff T, Schaefer M, Mwinyi J, Johne A, Sudhop T, Lutjohann D, Roots I, von Bergmann K: **Influence of the SLCO1B1*1b and *5 haplotypes on pravastatin's cholesterol lowering capabilities and basal sterol serum levels.** *Naunyn-Schmiedeberg's archives of pharmacology* 2006, **373:**45-50.

205. Igel M, Arnold KA, Niemi M, Hofmann U, Schwab M, Lutjohann D, von Bergmann K, Eichelbaum M, Kivisto KT: **Impact of the SLCO1B1 polymorphism on the pharmacokinetics and lipid-lowering efficacy of multiple-dose pravastatin.** *Clinical pharmacology and therapeutics* 2006, **79:**419-426.

206. Takane H, Miyata M, Burioka N, Shigemasa C, Shimizu E, Otsubo K, Ieiri I: **Pharmacogenetic determinants of variability in lipid-lowering response to pravastatin therapy.** *Journal of human genetics* 2006, **51:**822-826.

207. Lee YJ, Lee MG, Lim LA, Jang SB, Chung JY: **Effects of SLCO1B1 and ABCB1 genotypes on the pharmacokinetics of atorvastatin and 2-hydroxyatorvastatin in healthy Korean subjects.** *International journal of clinical pharmacology and therapeutics* 2010, **48:**36-45.

208. Niemi M: **Role of OATP transporters in the disposition of drugs.** *Pharmacogenomics* 2007, **8:**787-802.

209. Seithel A, Klein K, Zanger UM, Fromm MF, Konig J: **Non-synonymous polymorphisms in the human SLCO1B1 gene: an in vitro analysis of SNP c.1929A>C.** *Molecular genetics and genomics : MGG* 2008, **279:**149-157.

210. Michalski C, Cui Y, Nies AT, Nuessler AK, Neuhaus P, Zanger UM, Klein K, Eichelbaum M, Keppler D, Konig J: **A naturally occurring mutation in the SLC21A6 gene causing impaired membrane localization of the hepatocyte uptake transporter.** *The Journal of biological chemistry* 2002, **277:**43058-43063.

211. Ho WF, Koo SH, Yee JY, Lee EJ: **Genetic variations of the SLCO1B1 gene in the Chinese, Malay and Indian populations of Singapore.** *Drug metabolism and pharmacokinetics* 2008, **23:**476-482.

212. Rodrigues AC, Perin PM, Purim SG, Silbiger VN, Genvigir FD, Willrich MA, Arazi SS, Luchessi AD, Hirata MH, Bernik MM, et al: **Pharmacogenetics of OATP Transporters Reveals That SLCO1B1 c.388A>G Variant Is Determinant of Increased Atorvastatin Response.** *International journal of molecular sciences* 2011, **12:**5815-5827.

213. Hermann M, Bogsrud MP, Molden E, Asberg A, Mohebi BU, Ose L, Retterstol K: **Exposure of atorvastatin is unchanged but lactone and acid metabolites are increased several-fold in patients with atorvastatin-induced myopathy.** *Clinical pharmacology and therapeutics* 2006, **79:**532-539.
