# Supplementary material for: The Coriell personalized medicine collaborative pharmacogenomics appraisal, evidence scoring and interpretation system
Source: Genome Med. 2013 Oct 18;5(10):93. doi: 10.1186/gm499 (PMC3978656; doi:10.1186/gm499)
Supplement: Additional file 2 — Consists of extracts from Pharmacogenomics Appraisal, Evidence Scoring and Interpretation System (PhAESIS) submission documents for the seven drugs and nine genes approved for risk reporting by the Coriell Personalized Medicine Collaborative (CPMC) Pharmacogenomics Advisory Group (PAG). Sections S1 to S7 represent summary annotations of PhAESIS reports submitted to and subsequently approved by the CPMC PAG. The concise summaries include a description and mechanism of action of the drug under review, an overview of the PGx data for the drug, summary of the drug-gene evidence for the key PGx genes, strength of evidence scoring of genetic variants, genotype-phenotype interpretations, current FDA and other clinical association guidelines, and gaps in PGx knowledge for the drug-gene pair. The data are taken directly from PAG reports with the date of PhAESIS review by the PAG provided. In some cases, more recent data are also cited in the text. [file gm499-S2.docx]

**Extracts from PhAESIS submission documents for the seven drugs and nine genes approved for risk reporting by the CPMC PAG**

The sections below represent summary annotations of Pharmacogenomics Appraisal, Evidence-Scoring and Interpretation System (PhAESIS) reports submitted to and subsequently approved by the Coriell Personalized Medicine Collaborative (CPMC) Pharmacogenomics Advisory Group (PAG). The concise summaries include description and mechanism of action of the drug under review, an overview of the PGx data for the drug, summary of the drug-gene evidence for the key PGx genes, strength of evidence scoring of genetic variants, genotype-phenotype interpretations, current FDA and other clinical association guidelines, and gaps in PGx knowledge for the drug-gene pair. The data are taken directly from PAG reports with date of PhAESIS review by the PAG provided. In some cases more recent data are also cited in the text. Note, given the research setting, gene variant evaluations were prioritized to those present on the genotyping platforms used by the CPMC study (Affymetrix DMET Plus and Genomewide Human 6.0 arrays). As such the gene variant evidence tables (**S3, S6, S9, S11, S13, S15, S20, S23** and **S27**) include variants on these platforms and any other key variants identified during literature and database searches. Other reported variants are not systematically included.

**S1.0 Clopidogrel-CYP2C19** (Reviewed by PAG in March 2010 and update reviewed October 2010)

**S1.1 Description and mechanism of action of clopidogrel**

Clopidogrel bisulfate (Plavix) is an anti‐platelet medication, used to prevent atherothrombosis by inhibiting the formation of blood clots in patients with acute coronary syndrome (ACS), established peripheral arterial disease and those who have suffered other cardiovascular disease (CVD) related events such as myocardial infarction and ischemic stroke. It is also used in patients who are undergoing percutaneous coronary intervention.

Platelet activation and aggregation play a crucial role in the pathophysiology of atherothrombosis [[1](#_ENREF_1)]. Inhibition of platelet aggregation by clopidogrel can vary considerably between patients, with 20–40% of patients being classified as non-responders, poor-responders or resistant to clopidogrel because of low inhibition of ADP-induced platelet aggregation or activation [[2](#_ENREF_2)].

Clopidogrel is a prodrug, and must be metabolized by CYP450 enzymes to produce the active thiol metabolite that elicits the pharmacodynamic response, inhibition of platelet aggregation. The active metabolite of clopidogrel irreversibly antagonizes the adenosine diphosphate (ADP) receptor (coded by the P2Y12 gene), which in turn inactivates the fibrinogen receptor and thus inhibits platelet aggregation. Intestinal absorption of clopidogrel is limited by an intestinal efflux pump P-glycoprotein coded by the *ABCB1* gene. The majority of the prodrug is metabolized by ubiquitous esterases into inactive metabolites (85% of circulating metabolites). The minority is bioactivated in a two-step process by various CYP450 isoforms including CYP1A2, CYP2B6, CYP2C9, CYP2C19 and CYP3A4 [[3](#_ENREF_3)].

**S1.2 Pharmacogenomic studies of clopidogrel**

A number of studies have investigated the role of genetic variations in several genes involved in the pharmacodynamic (PD) and pharmacokinetic (PK) response to clopidogrel. For example, studies suggest an association of the C3435T variant (rs1045642) in ABCB1 gene with clopidogrel absorption in patients with cardiovascular diseases [[4-6](#_ENREF_4)]. However, this association is inconsistent, with some studies showing a lack of effect on platelet function [[7-9](#_ENREF_7)] as well as on clinical outcome [[10](#_ENREF_10), [11](#_ENREF_11)]. Polymorphisms in the gene encoding the P2Y12 receptor have failed to show a significant impact on clopidogrel response [[12-15](#_ENREF_12)]. A number of studies have examined the role of functional genetic variants in the CYP genes encoding enzymes involved in clopidogrel metabolism (CYP1A1, CYP2B6, CYP2C9, CYP2C19, CYP3A4 and CYP3A5). Although some have shown association of reduced function genetic variants in CYP3A4 [[12](#_ENREF_12)], CYP2B6 [[16](#_ENREF_16)], CYP2C9 [[2](#_ENREF_2)] and CYP2C19 [[2](#_ENREF_2), [15-21](#_ENREF_15)] with attenuation of the PK and PD responses to clopidogrel, the most consistent associations observed for primary clinical outcomes in patient populations has been for variants in the CYP2C19 gene (Mega [[5](#_ENREF_5), [16](#_ENREF_16), [22-28](#_ENREF_22)].

**S1.3 CYP2C19 and clopidogrel response**

The anti-platelet response to clopidogrel, as measured by *ex vivo* platelet aggregation assays, differs according to functional genetic variants of CYP2C19 [[29](#_ENREF_29)]. CYP2C19 *1, encodes a fully functional enzyme. Wild type homozygotes for this allele (*1/*1) are extensive metabolizers (EM) and show normal suppression of platelet activity after taking standard doses of clopidogrel. Loss-of-function alleles such as CYP2C19*2 and *3 encode genes with nucleotide changes that inactivate or reduce the enzyme’s activity and result in reduced metabolism of clopidogrel. Heterozygous carriers of a reduced function allele and a CYP2C19*1 allele are intermediate metabolizers (IM) while carriers of 2 reduced function alleles are poor metabolizers (PM) of clopidogrel. The CYP2C19*2 and CYP2C19*3 alleles account for over 85% of reduced function alleles in whites and over 99% in Asians. Other less frequent alleles associated with reduced metabolism include CYP2C19*4, *5, *6, *7, and *8. The CYP2C19*17 allele represents a gain-of-function allele and carriers of this allele (namely *1/*17 heterozygotes and *17/*17 homozygotes) have increased CYP2C19 activity and are termed ultra-rapid metabolizers (UM).

Pharmacodynamic studies have shown diminished anti-platelet response to clopidogrel for IMs and PMs compared to EMs. The relative difference in platelet inhibition (reduction in maximal platelet aggregation in response to clopidogrel) between genotype groups is typically greater than 30% (reviewed by [[29](#_ENREF_29)]). Recent publications have evaluated platelet response to higher than the standard 75mg/day clopidogrel dose in CYP2C19 intermediate and poor metabolizers and have shown improvement in some but not all patients [[30](#_ENREF_30), [31](#_ENREF_31)]. In patients with stable cardiovascular disease, tripling of the dose (225mg/day) resulted in equivalent platelet inhibition as observed in EMs on a standard dose; by contrast, doses as high as 300mg/day did not result in the same degree of platelet inhibition response in PMs [[30](#_ENREF_30)].

The association between CYP2C19 genotype and clopidogrel treatment outcome and adverse events has been supported by several cohort studies [[5](#_ENREF_5), [22-24](#_ENREF_22)], post-hoc clinical trial analyses [[16](#_ENREF_16), [32](#_ENREF_32)] as well as a number of meta-analyses [[25](#_ENREF_25), [27](#_ENREF_27), [28](#_ENREF_28)]. Other studies have provided a lack of support for the association. These include a placebo controlled trial that showed no significant reduction in efficacy of clopidogrel in *2 and *3 carriers compared to placebo. However, this study was based on patients with ACS or atrial fibrillation with relatively low rates of PCI with stenting (14.5%) compared with the majority of other published studies (>70%) [[33](#_ENREF_33)]. More recently, since the October 2010 PAG review of the clopidogrel-CYP2C19 PhAESIS report, a large meta-analysis examined the association of CYP2C19 *2 carrier status with risk of adverse cardiovascular outcome and concluded that carriers were not at clinically relevant increased risk [[34](#_ENREF_34)]. Closer examination of all published data has supported clear and significant evidence for a differential effect of genotype on risk of major adverse cardiovascular outcomes following PCI compared to other clopidogrel indications [[35](#_ENREF_35)] with the weight of published evidence demonstrating significant increased risk of MACE and stent thrombosis in carriers of loss-of-function alleles (IMs and PMs) compared to non-carriers. The key primary studies evaluating the association between CYP2C19 genotype and clopidogrel treatment outcome are summarized in **Table S2**.

Gain-of-function CYP2C19*17 allele carriers demonstrate a greater platelet response to standard doses of clopidogrel. These ultra-rapid metabolizers (UM) of clopidogrel have been shown to have an enhanced platelet response to clopidogrel [[36](#_ENREF_36)] and show a concomitant increased risk of major bleeding [[32](#_ENREF_32), [36](#_ENREF_36)] as well as increased drug efficacy [[11](#_ENREF_11), [33](#_ENREF_33)].

Finally, the clinical response to clopidogrel for individuals heterozygous for a loss-of-function allele (such as CYP2C19*2) and the CYP2C19*17 gain-of-function allele remains unclear. However, a 2010 study examined the *2 and *17 alleles with respect to ADP-induced platelet aggregation [[36](#_ENREF_36)]. A gene-dose effect was observed for both variants such that there was a gradual decrease of platelet aggregation in patients that were carriers of two *2 alleles > those with one *2 allele and lacking a *17 > carriers of both*17 and*2 > those lacking both*17 or *2 alleles > those with one *17 allele and lacking a *2 > and carriers of two *17 alleles (p<0.001).

**S1.4 Strength of evidence scoring of CYP2C19 variants**

More than 30 polymorphic variants or ‘star alleles’ of CYP2C19 have been described to date (www.cypalleles.ki.se/cyp2c19.htm; updated 3/7/11). Of the variants reviewed in this report, CYP2C19*1, CYP2C19*2, CYP2C19*3, CYP2C19*4, CYP2C19*5, CYP2C19*8 and CYP2C19*17 have clinical outcomes data available and are all assigned evidence code “1” (**Table S3**)**.** CYP2C19*6 and CYP2C19*7 are assigned evidence code “6_scd_” and “6_se_” respectively since the highest evidence available is PK/PD evidence for another drug along with molecular data that supports effect on enzyme function. CYP2C19*10 is assigned evidence code 8 and CYP2C19*9 and CYP2C19*12 are assigned evidence code “11” since the highest evidence they have is for molecular functional study with a probe drug and another drug respectively. Variants CYP2C19*SD and CYP2C19 G439X were evaluated based on their presence on the DMET-plus genechip and are assigned evidence code “13” since they lack clinical or molecular functional data, being identified through gene sequencing studies. CYP2C19*13-15 are assigned evidence code “14” as they do not appear to consistently alter CYP2C9 activity. **Table S3** provides a summary of the metabolic phenotypes, frequency and evidence scoring of the CYP2C19 variants.

**S1.5 Clopidogrel-CYP2C19 genotype-phenotype interpretation**

To classify the diploid individual to a predicted CYP2C19-drug metabolism phenotype, we used the convention from published studies [[16](#_ENREF_16), [17](#_ENREF_17)] and the predicted combined effect of the two inherited CYP2C19*alleles. Extensive metabolizers (EM) are defined as having two alleles conferring normal or near-normal activity; ultra-rapid metabolizers (UM) as those with two increased activity alleles or one normal and one increased activity allele; intermediate metabolizers (IM) as those with one normal and one reduced activity allele; and poor metabolizers (PM) are those with two reduced activity alleles. Currently the drug metabolizing phenotype for the presence of one increased activity and one reduced activity alleles (e.g. CYP2C19*2/*17) is unknown. This highlights the need for further clinical and population-based studies to further elucidate the phenotypic effect in these compound genetic variant carriers. The CYP2C19-Clopidogrel genotype-phenotype interpretation for all the expected genetic variant combinations (diplotypes) is provided in the extended Punnett square **Table S4.**

**S1.6 FDA and other clinical association guidelines**

In March 2010, the FDA issued a ‘Black Box Warning’ of diminished effectiveness in poor metabolizers in the revised Plavix drug label [[37](#_ENREF_37)]. This stated that (a) at recommended doses, PMs convert less Plavix to active metabolite and have diminished platelet inhibition; (b) PMs with ACS or undergoing PCI who are treated with Plavix at recommended doses are at risk of CVD death, heart attack and stroke; (c) CYP2C19 genotyping tests are available to identify PMs; and (d) alternative treatment strategy (dosing or medication) should be considered for PMs. The revised label does not include recommendations for IMs or UMs and does not recommend specific dosing strategies for PMs.

The Plavix label also warns that concomitant use of Plavix and strong or moderate CYP2C19 inhibitors should be avoided.

In July 2010, the American Heart Association (AHA) and the American College of Cardiology Foundation (ACCF) published a Clinical Alert in response to the FDA's black box warning on clopidogrel [[34](#_ENREF_34)]. They stated that: “the evidence base is insufficient to recommend routine genetic testing at the present time” and that "clinical judgment is required to assess clinical risk and variability in patients considered at increased risk. Genetic testing to determine if a patient is predisposed to poor clopidogrel metabolism ("poor metabolizers") may be considered before starting clopidogrel therapy in patients believed to be at moderate or high risk for poor outcomes. This might include, among others, patients undergoing elective high-risk PCI procedures (e.g., treatment of extensive and/or very complex disease)."

Therefore, at the present time there are no standardized clinical guidelines to identify and manage patients with an inadequate response to clopidogrel. optimal dose regimen for poor metabolizers have yet to be determined [[30](#_ENREF_30), [31](#_ENREF_31)]. Several alternatives have been suggested, such as higher loading or maintenance doses of clopidogrel in IMs [[30](#_ENREF_30)], dual therapy with aspirin or treatment with another antiplatelet medication [[34](#_ENREF_34)]. Further clinical studies are required to guide physicians on how to manage patients genetically predisposed to inadequate or potentially enhanced response to clopidogrel.

**S1.7 Gaps in clopidogrel PGx knowledge**

Overall there is a fairly large body of evidence supporting the association of many of the CYP2C19 loss-of-function variants with risk of adverse cardiovascular events specifically in patients with acute coronary syndromes undergoing percutaneous coronary interventions. However, further prospective studies are needed to elucidate the clinical utility, if any, of genotype-guided clopidogrel therapy for other indications of antiplatelet therapy. The results of the clopidogrel PhAESIS review has also highlighted 15 CYP2C19 gene variants (*9, *10, *11, *12, *13, *14, *15, *16, *18, *19, *22, *23, *24, *25 and *26) with insufficient evidence for an effect on clopidogrel response. In addition, within the group of variants with demonstrated effect on clopidogrel response there are genotype combinations (diplotypes) where clinical outcomes have not been fully elucidated. This includes diplotype carries of loss-of-function and gain-of-function (*17) variants and individuals classed as UMs, for which the effect of enhanced response to clopidogrel with respect to drug efficacy and risk of bleeding remains unclear.

**S2.0 PPIs-CYP2C19** (Reviewed by PAG in June 2011)

**S2.1 Description and mechanism of action of proton pump inhibitors**

Proton pump inhibitors (PPIs) bind to hydrogen-potassium adenosine triphosphatase (H^+^/K^+^-ATPase) pumps within the cytoplasm of the parietal cell of the stomach to inhibit secretion of gastric acid into the upper gastrointestinal tract [[38](#_ENREF_38), [39](#_ENREF_39)]. PPI antisecretory effects reduce meal-stimulated gastric output and secretion volume, and raise levels of serum gastrin involved in acid secretion signaling [[40](#_ENREF_40)] for treating acid-related disorders, including duodenal and gastric ulcers, gastroesophageal reflux disease (GERD or GORD), erosive esophagitis (EE), Barrett’s esophagus, pathological hypersecretory conditions (e.g. Zollinger-Ellison syndrome (ZES), multiple endocrine adenomas and systemic mastocytosis), non-steroidal antiinflamatory drug (NSAID)-associated gastric ulcer, and heartburn [[41](#_ENREF_41)]. Polytherapy with PPIs and antibiotics is used to eradicate *Helicobacter pylori* in patients with peptic ulcer disease or symptomatic non-ulcer disease; and PPI/NSAID therapy treats arthritis, ankylosing spondylitis, and reduces risk of NSAID-associated gastric ulcers.
Clinical efficacy depends on maintaining sufficient drug exposure to raise intragastric pH (>3 for duodenal ulcers, >4 for gastric ulcers and EE, >6 for bleeding gastric ulcers); and this drug response correlates best with plasma drug levels measured by the area under the drug concentration/time curve (AUC). The rate of PPI clearance by metabolic cytochrome P450 (CYP) enzymes or other mechanisms therefore affects drug efficacy [[38](#_ENREF_38), [39](#_ENREF_39)]. A number of studies have shown CYP2C19 genetics to influence the pharmacokinetics, pharmacodynamics, and clinical outcomes of PPIs.

**S2.2 Pharmacogenomic studies of PPIs**

Genetic variation in CYP2C19 influences PPI pharmacokinetics and efficacy [[38](#_ENREF_38), [39](#_ENREF_39)] to the extent that different PPIs are substrates for CYP2C19 (omeprazole > pantoprazole > lansoprazole > rabeprazole) [[42](#_ENREF_42)]. CYP2C19 metabolizer status shows significant variable in *in vivo* PPI pharmacokinetics for common CYP2C19 genotypes [[43-51](#_ENREF_43)], particularly in comparing poor metabolizers (PMs) with extensive metabolizers (EMs). In addition, *in vitro* studies showed significantly altered omeprazole activity for several uncommon alleles: CYP2C19*9, *10, *16, *18, *19, A161P, W212C, and D360N [[52](#_ENREF_52)]. Studies differ in assessing the effect of the gain-of-function CYP2C19*17 allele. Some studies suggest *17/*17 carriers are ultra-rapid metabolizers (UMs) [[43](#_ENREF_43), [53](#_ENREF_53)]; *1/*17 carriers are between EMs and UMs; and *2/*17 carriers are IMs [[54](#_ENREF_54)]. However, other studies show no significant pharmacokinetic or PPI response differences for *17 [[43](#_ENREF_43), [44](#_ENREF_44), [54](#_ENREF_54)].

CYP2C19 metabolizer status relates to variability in PPI response both in terms of pharmacodynamics (e.g. inhibition of intragastric pH, serum gastrin levels), and cure rates for GERD and *H. pylori*. CYP2C19 genetics are associated with PPI response in most studies (for pantoprazole, lansoprazole, omeprazole, rabeprazole, or mixed PPI treatment) [[48-51](#_ENREF_48), [54-60](#_ENREF_54)], but not all (for rabeprazole alone and pantoprazole/antibiotic co-therapy) [[45](#_ENREF_45), [46](#_ENREF_46), [61](#_ENREF_61), [62](#_ENREF_62)]. A summary of CYP2C19 pharmacogenetics on PPI response is detailed in the next section.

Other studies show controversial evidence for the combined effect of CYP2C19 with other gene variants on PPI response. CYP2C19*1/*2 carriers with the ABCB1 rs1045642 (3435C>T) TT genotype showed significantly higher GERD cure rates [[63](#_ENREF_63)]. However, this seems contrary to data from a study of renal transplant patients showing the 3435 C allele was associated with significantly increased lansoprazole AUC and Cmax in CYP2C19 EMs during tacrolimus co-treatment [[64](#_ENREF_64)]. In addition, no genetic effect was seen for PPI efficacy in *H. pylori* cure rates using pantoprazole-antibiotics polytherapy [[45](#_ENREF_45), [62](#_ENREF_62)]. IL1B rs16944 (-511T>C) and CYP2C19 variants were associated with *H. pylori* cure rates in some studies [[56](#_ENREF_56), [65](#_ENREF_65), [66](#_ENREF_66)], but not in others [[67](#_ENREF_67)].

**S2.3 CYP2C19 and PPI response**

CYP2C19 metabolizer status relates to PPI response, where carriers of low-activity CYP2C19 genotypes exhibit greater response; while carriers of high-activity CYP2C19 genotypes show the lowest treatment effects [[51](#_ENREF_51)] (**Table S5**). Based upon evidence from four studies of nineteen CYP2C19 *17/*17 carriers in an efficacy study of pantoprazole with antibiotics for *H. pylori* treatment (n=125) [[54](#_ENREF_54)], and in pharmacokinetic studies of omeprazole (n=17-97) [[43](#_ENREF_43), [44](#_ENREF_44), [47](#_ENREF_47)], the Royal Dutch Association for the Advancement of Pharmacy recommends PPI dose increases to compensate for enhanced metabolism in UMs: increase dose 50-100% for esomeprazole, 200% for lansoprazole, 100-200% for omeprazole, 400% for pantoprazole, and no recommendation for rabeprazole [[68](#_ENREF_68)]. However, the data seems equivocal since only two pharmacokinetic study showed significant pharmacokinetic differences for *17 carriers [[43](#_ENREF_43), [47](#_ENREF_47)]; while the other two studies show no significant difference in *H. pylori* eradication or omeprazole metabolism for *17 [[44](#_ENREF_44), [54](#_ENREF_54)]. Dose reductions are recommended in the drug label for certain subpopulations, such as delayed-release pantoprazole in pediatric CYP2C19 PMs and for immediate-release omeprazole in Asians (based on greater relative CYP2C19 PM frequency than other ethnic groups).

***Gastroesophageal Reflux Disease (GERD) and Erosive Esophagus (EE)*** CYP2C19 metabolizer status is significantly associated with cure rates. In a study in 65 subjects of unknown ethnicity GERD cure rates were significantly increased in proportion with lower plasma levels of lansoprazole and CYP2C19 metabolizer status: EMs (*1/*1: 45.8% cure rate), IMs (*1/*2, *1/*3: 67.9% cure rate), PMs (*2/*2, *2/*3, *3/*3: 84.6% cure rate) [[55](#_ENREF_55)]. Significantly improved cure rates increased in proportion with CYP2C19 metabolizer status in a study of 88 Japanese given 8 weeks’ lansoprazole treatment for GERD [[58](#_ENREF_58)], and in Iranians given 4 weeks’ omeprazole treatment for EE [[59](#_ENREF_59)]. However, in a study in 119 Japanese given 6-12 months’ omeprazole treatment for GERD, CYP2C19 metabolizer status did not significantly impact the cure rate [[61](#_ENREF_61)]. One pharmacogenetic study shows the utility of CYP2C19 genotype in the PPI test, which employs a high-dose regimen of PPIs as a diagnostic tool to identify GERD among patients presenting symptoms. Determination of CYP2C19 genotype significantly improved the sensitivity and accuracy of predicting GERD by the PPI test. Briefly, CYP2C19 metabolizer status was determined in 158 Chinese patients with GERD and erosive esophagus: 63 EMs (*1/*1), 75 IMs (*1/*2, *1/*3), 20 PMs (*2/*2, *2/*3, *3/*3). Defining therapeutic response as 50% reduction of symptoms, significant improvement of diagnostic specificity and accuracy was achieved for EMs and IMs in predicting rabeprazole and pantoprazole response [[60](#_ENREF_60)].

***Helicobacter pylori*** CYP2C19 metabolizer status was predictive of PPI treatment success in eradicating *H. plyori* in several studies, with lower metabolizer status although not all were statistically significant [[69](#_ENREF_69)]. For example, in a study of 164 patients given omeprazole or rabeprazole with antibiotics improved healing was seen in carriers with low CYP2C19 metabolizer status, although the difference was not significant: EM (73-81% cure rate), IM and PM (83-88% cure rate) (genotypes not given) [[70](#_ENREF_70)]; and in a study of 139 Polish Caucasians given pantoprazole with or without antibiotics, cure rates improved with reduced metabolizer status, although the difference was not significant: EM (*1/*1, *1/*17, *17/*17: 70.8% cure rate), IM (*1/*2, *2/*17: 83.9% cure rate), PM (*2/*2: 100% cure rate) [[45](#_ENREF_45)]. In a study of 183 subjects, cure rate was significantly improved for carriers with low CYP2C19 metabolizer status for rabeprazole with antibiotics (p=0.038): EM (62.5 cure rate), IM (87.1% cure rate) (genotypes not given); non-significant differences were seen for omeprazole with antibiotics: EM (76.2%), IM and PM (90% cure rate); and the cure rate for lansoprazole with antibiotics was significantly high regardless of CYP2C19 metabolizer status (89-90% for EM, IM, PM) [[71](#_ENREF_71)]. In a study of 249 subjects given omeprazole, lansoprazole, or rabeprazole with antibiotics, cure rates improved with lower CYP2C19 metabolizer status, although the difference was not significant: EM (69.1% cure rate), IM (74.4% cure rate), PM (83.7% cure rate); but treatment failure was significantly associated with CYP2C19 EM status (OR=3.00, p=0.03) [[56](#_ENREF_56)].

**S2.4 Strength of evidence scoring of CYP2C19 variants**

More than 30 polymorphic variants or ‘star alleles’ of CYP2C19 have been described to date by the CYP Allele Nomenclature Committee (www.cypalleles.ki.se/cyp2c19.htm, updated 3/7/11). Of the variants reviewed in this report, CYP2C19*1, *2, and *3 have significant clinical outcomes data and are assigned evidence code “1” (**Table S6**). CYP2C19*17 has only suggestive clinical outcome data but significant in vivo pharmacokinetic data, thus is assigned evidence code “2” CYP2C19*5, *6, *8, *9, and *10 have significant in vitro pharmacokinetic data, thus are assigned evidence code “3”. CYP2C19*4 has significant clinical outcomes data for another drug (i.e. clopidogrel) and is a null mutation type, thus is assigned evidence code “5n”. CYP2C19*7 has significant in vivo pharmacokinetic data for another drug and is a splice defect mutation type, thus is assigned evidence code “6se”. CYP2C19*12 has protein instability data in vitro, thus is assigned evidence code “11”. CYP2C19*16, *18, and *19 variants have significant in vitro pharmacokinetic data but are rare, thus are assigned evidence code “12”. CYP2C19*SD and G439X variants have no in vitro or in vivo data, thus are assigned evidence code “13”. CYP2C19*13, *14, *15, M74T, E122A, and F168L have normal or near normal activity in vitro, thus are assigned evidence code “14”. **Table S6** provides a summary predicted allelic phenotypes, allele frequency, and evidence score relating each CYP2C19 to observed or predicted PPI effects, including those with good evidence (score ≤7), and those that lack sufficient evidence (score ≥ 8).

**S2.5 PPI-CYP2C19 genotype-phenotype interpretation**

The predicted metabolic phenotype of CYP2C19 genotypes include extensive metabolizers (EMs) with two normal-activity alleles (*1/*1), ultra-rapid metabolizers (UMs) carrying two increased-activity alleles (*17/*17), intermediate metabolizers (IMs) with one normal and one reduced activity allele (e.g. *1/*2), and poor metabolizers (PMs) carry two reduced activity alleles (e.g. *2/*2). Heterozygous carriers of the enhanced-activity allele with a reduced-activity allele (e.g. *2/*17) are designated IMs [[54](#_ENREF_54)]. Currently data is lacking to predict the drug metabolizing phenotype for carriers of one normal-activity allele with an increased-activity allele (e.g. *1/*17), although it is estimated to be between UM and EM [[43](#_ENREF_43)]. Genotype-phenotype interpretation for all possible genotypes from variants included for CPMC analysis are summarized in the extended Punnett square **Table S7**.

**S2.6 FDA and Other Clinical Association Guidelines**

Dose reduction is recommended in the omeprazole drug label for Asians and in the pantoprazole drug label for pediatric patients who are CYP2C19 PMs. CYP2C19 IMs and PMs show improved PPI efficacy as measured by intragastric pH inhibition, duration of inhibition, and cure rates for GERD and *H. pylori*, while high-activity CYP2C19 genotypes show lower PPI efficacy. Irrespective of CYP2C19 genetics, PPIs are contraindicated for co-use with other CYP2C19-dependent drugs and drugs that depend on low gastric pH; and this effect is further mitigated in low metabolizers of CYP2C19.

**S2.7 Gaps in PPI PGx knowledge**

There is a paucity of data exploring the effects of *17, in particular the *17/*17 genotype, on PPI response. Available data assessing the effect of the gain-of-function CYP2C19*17 allele with PPIs shows differing results [[43](#_ENREF_43), [44](#_ENREF_44), [47](#_ENREF_47), [53](#_ENREF_53), [54](#_ENREF_54)]. Carriers of the *17/*17 genotype, which are expected to possess the highest metabolic activity of all CYP2C19 genotypes, show differences in PPI pharmacokinetics in some studies [[45](#_ENREF_45), [47](#_ENREF_47)], but not others [[44](#_ENREF_44)]. Furthermore, *17 homozygotes do not appear to show clinically relevant differences in treatment response [[54](#_ENREF_54)], however further studies are needed to clarify the clinical impact, if any, of this variant [[72](#_ENREF_72)]. In addition, given that many of the published studies have either not interrogated the variant that defines the *17 allele or have grouped *17 homozygotes and *1/*17 heterozygotes with *1/*1 as extensive metabolizers [[45](#_ENREF_45), [46](#_ENREF_46), [48-50](#_ENREF_48), [55](#_ENREF_55), [56](#_ENREF_56)], the effect of true *1 homozygosity on the PK and treatment response to PPIs needs further investigation. Finally, numerous CYP2C19 variants (with evidence code 8-13) have less than substantial evidence to evaluate their potential impact on PPIs.

**S3.0 Celecoxib-CYP2C9** (Reviewed by PAG in March 2012)

**S3.1 Description and mechanism of action of celecoxib**Celecoxib (Celebrex) is a non-steroidal anti-inflammatory drug (NSAID) with analgesic and antipyretic properties that selectively inhibits cyclooxygenase-2 (COX-2). Pharmacodynamic effects of celecoxib include reduction in joint tenderness, pain, and swelling, and water and/or sodium retention via inhibition of PGE2 synthesis. Celecoxib is used in the treatment of osteoarthritis, rheumatoid arthritis, acute pain, painful menstruation, and menstrual symptoms, and to reduce numbers of colon and rectum polyps in patients with familial adenomatous polyposis. However, following the withdrawal of COX-2 selective NSAID, rofecoxib (Vioxx) in 2004, concerns about adverse events and lack of comprehensive efficacy data a press release from the European Medicines Agency review of celecoxib for the use of familial adenomatous polyposis deemed the benefits of celecoxib (marketed as Onsenal) do not to outweigh life-threatening gastrointestinal bleeding and cardiovascular adverse events. A 20,000 high-risk patient study of the risk profile of celecoxib compared to non-selective NSAIDs is currently underway, with completion expected in 2014.

**S3.2 Pharmacogenomic studies of celecoxib**

Pharmacogenetics studies show functional variants of CYP2C9, which is the predominant inactivation pathway (65-90%) [[73](#_ENREF_73)], are associated with variation in celecoxib pharmacokinetics and effects. In a study of 17 Caucasians given a single oral dose of 200 mg celecoxib, the AUC was increased about 2.2-fold and the clearance was correspondingly lower in three CYP2C9*3 carriers (two *1/*3 and one *3/*3) compared to wild type. In this study two *1/*2 heterozygotes showed little difference in celecoxib AUC or clearance compared to twelve CYP2C9 EMs [[73](#_ENREF_73)]. In another study in 21 healthy Caucasians, the CYP2C9*3 allele was associated with decreased celecoxib clearance in additive manner, with three *3 homozygotes showing greatest reduction of activity, and four *1/*3 carriers showing intermediate reduction in metabolic activity. The *2 allele by contrast showed no significant effect on pharmacokinetics [[74](#_ENREF_74)]. In a study of 13 individuals of unknown ethnicity, given 7 days of 200 mg per day doses of celecoxib, three *3/*3 carriers showed significantly greater t1/2, AUC, Cmax, and significantly less clearance (7-fold less) than wild type; while three *1/*3 carriers and seven wild type subjects showed celecoxib pharmacokinetics similar to wild type [[75](#_ENREF_75)]. In a study of eleven pediatric patients with recurrent solid tumors or refractory acute lymphoblastic leukemia, single-dose and multi-dose pharmacokinetics were measured for 250 mg/m^2^ twice daily celecoxib [[76](#_ENREF_76)]. Data showed children exhibit similar extent and rate of absorption, but significantly greater clearance and faster elimination half-life than adults for a comparable dose by weight. This translates to approximately 50% lower celecoxib exposure in children than in adults for the same relative dose. Subsequent analysis in four of the children showed CYP2C9 influenced pharmacokinetics. One CYP2C9*3/*3 subject exhibited significantly higher celecoxib exposure and lower relative elimination than three other subjects (one *1/*2 and two *1/*1). The CYP2C9*3 homozygote showed a 10-fold increase in celecoxib AUC, one-tenth the clearance, and more than 7-fold increased half-life compared to other genotypes for a single dose. Steady-state AUC was also 10-fold greater as calculated from a 6-8-fold greater Cmax. In the 5 to 62.5 weeks treatment, no sign of acute cardiovascular or other toxicity was attributed to celecoxib. The authors designate IM status for a single CYP2C9*1/*2 heterozygote, although the pharmacokinetic parameters of this subject were similar to wild type, with the steady-state half-life, AUC, and clearance values within the range of that of wild type carriers. Thus, the CYP2C9*1/*2 genotype may better follow EM status. No heterozygote carriers of *3 were tested to assess the genetic model of the *3 variant.

**S3.3 CYP2C9 and celecoxib response**

Only two studies explore association between CYP2C9 variants and celecoxib effects; and of those only one gives data specific to celecoxib**.** The Adenoma Prevention with celecoxib trial evaluated the contribution of 357 CYP2C9*2 (*1/*2, *2/*2) and 201 CYP2C9*3 (*1/*3, *2/*3, *3/*3) genotype carrier groups to 1,102 non-carriers on celecoxib response and adverse events in preventing recurrence of colorectal adenomas. Adverse drug reactions were deemed too great for the safe use of celecoxib for colorectal adenomas; however data from trials reveal the contribution of CYP2C9 variants on celecoxib. CYP2C9*2 and *3 carriers showed no significant improvement in reducing risk of adenoma recurrence compared to wild type carriers with low doses (200 mg twice daily) or high doses (400 mg twice daily) of celecoxib; although the *3 carrier group showed slightly improved efficacy (RR=0.41) compared to non-carriers (RR=0.54-0.56) at the high dose. However, celecoxib response was preferentially greater at high dose among *3 carriers, with about 20% greater reduction in the risk of adenoma recurrence (RR=0.51, p=0.001). The cumulative incidence of cardiovascular and thrombotic events was increased in both the *2 (RR=2.75) and *3 (RR=2.69) carrier groups compared to wild type (RR=1.37) at high doses [[77](#_ENREF_77)]. Another study showed the effect of multiple NSAIDs, including 15 of 78 subjects taking celecoxib, in which fifteen CYP2C9*1/*2 and twelve CYP2C9*1/*3 carriers showed disproportionately greater odds of gastroduodenal bleeding from acute (<1 month) doses. The odds ratio was smaller for *2 than *3 heterozygotes (3.8 versus 12.9, respectively) [[78](#_ENREF_78)]. However, because the data were not stratified by drug, and the majority of subjects were taking another NSAIDs (e.g. diclofenac, ibuprofen, naproxen, piroxicam), it is not possible to ascribe this result to CYP2C9 on celecoxib alone. Clinical outcomes studies are summarized in **Table S8**.

**S3.4 Strength of evidence scoring of CYP2C9 variants**

More than 57 polymorphic variants or ‘star alleles’ of CYP2C9 have been described to date (www.cypalleles.ki.se/cyp2c9.htm, updated 12/20/2012). Of the nineteen CYP2C9 variants evaluated here, variants with known or predicted to affect celecoxib include: CYP2C9*2, *3, *5, *6, and *11. CYP2C9*2 and *3 are assigned Evidence code “1”. CYP2C9*5 and *11 are assigned Evidence code “4scd”. CYP2C9*6 is assigned Evidence code “5n”. Variants with insufficient evidence for influence on celecoxib effects include: CYP2C9*4, *7, *8, *9, *10, *12, *13, *15, *16, *25, Y358C, A441A, and G475G. CYP2C9*13 is assigned Evidence code “9”. CYP2C9*8 and *14 are assigned Evidence code “10”. CYP2C9*12, *15, *16 and *25 are assigned Evidence code “11”. CYP2C9*4 is assigned Evidence code “12”. CYP2C9 Y358C, A441A, and G475G are assigned Evidence code “13” and CYP2C9*7, *9, and *10 are assigned Evidence code “14”. Variant allele phenotypes, frequency, and evidence scoring are summarized in **Table S9**.

**S3.5 Celecoxib-CYP2C9 genotype-phenotype interpretation**

The pharmacokinetics of celecoxib is significantly altered by CYP2C9 variants, but there is only one study relating CYP2C9 variant effects on celecoxib efficacy and tolerance [[77](#_ENREF_77)]. In terms of clinical outcome, the *2 and *3 variants exhibit increased risk of celecoxib-induced adverse events at high doses (400 mg twice daily) with the greatest risk in *3/*3 homozygotes. Carriers of the *3 allele (*1/*3, *2/*3, *3/*3) also show greater celecoxib response (less recurrence of colorectal adenomas) at high doses, although this treatment is outweighed by the prohibitive risk of adverse events. Given the paucity of published data, both on less characterized variants with reduced-activity (*5, *6 and *11) and on the drug response and tolerance in heterozygotes (i.e. those assigned as IM in **Table S10**), all homozygotes or compound heterozygotes for a reduced activity variant (i.e. those assigned as PMs), are expected to be at increased risk of ADR when on high dose celecoxib. The therapeutic response at low dose for all metabolizer types and at high dose for IMs is currently unknown. Based on this combined information, the genotype-phenotype interpretation for all possible genotypes included for CPMC analysis are summarized in **Table S10**.

**S3.6 FDA and other clinical association guidelines**

The Federal Drug Administration issues a list of pharmacogenomic biomarkers to be used to identify drug responders versus non-responders avoid adverse drug events and provide dosing guidelines for affected populations. As of January 2012 the following label [[79](#_ENREF_79)] sections contain specific information regarding CYP2C9 on celecoxib (FDA Pharmacogenomic Biomarkers, www.fda.gov). Per the FDA: “CYP2C9 activity is reduced in individuals with genetic polymorphisms that lead to reduced enzyme activity, such as those homozygous for the CYP2C9*2 and CYP2C9*3 polymorphisms. Limited data from 4 published reports that included a total of 8 subjects with the homozygous CYP2C9*3/*3 genotype showed celecoxib systemic levels that were 3- to 7-fold higher in these subjects compared to subjects with CYP2C9*1/*1 or *I/*3 genotypes. The pharmacokinetics of celecoxib have not been evaluated in subjects with other CYP2C9 polymorphisms, such as *2, *5, *6, *9 and *11. It is estimated that the frequency of the homozygous *3/*3 genotype is 0.3% to 1.0% in various ethnic groups”.

The drug label cites CYP2C9*2 homozygotes as having reduced enzyme activity, but explains that pharmacokinetics of celecoxib have not been evaluated in subjects with *2 among other CYP2C9 variants, which agrees with a search of the literature to date. Per the drug label (Use in Specific Populations), “patients who are known or suspected to be poor CYP2C9 metabolizers based on genotype or previous history/experience with other CYP2C9 substrates (such as warfarin, phenytoin) should be administered celecoxib with caution”. Per the dosage, administration, and drug interactions section: consider a dose at 50% of the recommended starting dose in poor metabolizers (i.e. CYP2C9*3/*3). Consider using alternative management in juvenile rheumatoid arthritis patients who are poor metabolizers. A study of single dose and multiple dose pharmacokinetics for four pediatric patients (two CYP2C9*1/*1, one *1/*2, and one *3/*3 carrier) given 250 mg/ m^2^ celecoxib for antiangiogenic treatment of a solid tumor showed a relative 10-fold increase in celecoxib AUC (108,251 vs 11,798 mean microg/L-h), about one-tenth clearance (0.09 vs 0.6 mean L/h-kg), and more than 7-fold increased half-life (30 vs. 4 mean h) in the *3 homozygote compared to the other genotypes for a single dose. Steady-state AUC was 10-fold greater. None of the subjects treated between 5 and 62.5 weeks showed symptoms of acute cardiovascular or other toxicity in this short-term study. However, longer use, higher dosing, and/or comorbidities that exacerbate the metabolic profile of the CYP2C9 PM may lead to adverse reactions.

**S3.7 Gaps in celecoxib PGx knowledge**

Despite studies showing the impact of CYP2C9 variation on celecoxib pharmacokinetics, only one study evaluates CYP2C9 pharmacogenetics (*2 and *3) on celecoxib-specific response and tolerance. Several CYP2C9 variants with evidence of reduced function with other substrates (*4, *8, *12-*16, and *25) have not been studied for their impact on celecoxib; and other CYP2C9 variants found in mutation screens have no functional data at all (Y358C, A441A, and G475G).

**S4.0 Warfarin-CYP2C9/VKORC1/CYP4F2** (Reviewed by PAG in March 2010)

**S4.1 Description and mechanism of action of warfarin**

Warfarin, a synthetic derivative of the plant chemical coumarin, is a highly prescribed and effective oral anticoagulant used for the treatment and prevention of thrombotic events. Coumarins (warfarin, Coumadin, Jantoven and others) have a narrow therapeutic window and wide inter-individual variability making dosing problematic and requiring significant patient management to avoid serious adverse drug reactions (ADR). Dosing is determined empirically, often based on age and underlying conditions, with adjustments made until the target International Normalized Ratio (INR) therapeutic range of between 2 and 3 is achieved and maintained [[80-82](#_ENREF_80)]. Empiric starting doses range from 3-5mg/day, but stable doses once achieved can range from 1-20mg/day [[83](#_ENREF_83), [84](#_ENREF_84)]. Over-anticoagulation (an INR greater than 3) can result in dangerous bleeding episodes and under-anticoagulation (an INR < 2) is associated with an increased risk of thrombosis. Warfarin-associated ADRs, are one of the most common causes for emergency department visits in the US [[85](#_ENREF_85), [86](#_ENREF_86)] resulting in about 60,000-85,000 serious bleeding events and 17,000 strokes annually. Clinical factors, demographic variables and variation in genes have been shown to explain as much as 59% of variance in warfarin dose in Caucasians [[87](#_ENREF_87)].

Warfarin is a racemic mixture of the *R*- and *S*-stereoisomers with the *S*-enantiomer exhibiting 2 to 5 times more anticoagulant activity than the *R*-enantiomer in humans. The S isomer is metabolized primarily by the CYP450 enzyme CYP2C9. Vitamin K is essential for activation of clotting factors and warfarin is thought to interfere with clotting factor synthesis by reducing the regeneration of vitamin K from vitamin K epoxide in the vitamin K cycle. This is achieved through inhibition of the VKORC1 subunit of the enzyme complex, vitamin K epoxide reductase (VKOR). CYP4F2 is the primary human liver microsomal Vitamin K1 oxidase that removes vitamin K from the vitamin K cycling pathway [[88](#_ENREF_88)]. CYP4F2 may be an important counterpart to VKORC1 in limiting excessive accumulation of vitamin K.

**S4.2 Pharmacogenomic studies of warfarin**

Genetic polymorphisms in *VKORC1* and *CYP2C9*, genes controlling vitamin K1 (VK1) epoxide reduction and (*S*)-warfarin metabolism, respectively, are established major contributors to interindividual variability in warfarin dose. Several studies have also supported a lesser but significant role for CYP4F2, encoding the vitamin K oxidase, in modulating warfarin dose requirements. Evidence supporting a role for *VKORC1*, *CYP2C9* and *CYP4F2* is summarized below. Minor roles for genetic variations in GGCX (activator of vitamin K-dependent clotting factors), EPHX1 (a putative subunit of the VKOR complex), CALU (thought to inhibit transfer of VKH2 to GGCX) and APOE (role in transport of vitamin K to the liver) in warfarin dose prediction have also been suggested [[89-94](#_ENREF_89)]. However, these results have not been consistently replicated across studies, and for now the data are inconclusive [[87](#_ENREF_87), [89-91](#_ENREF_89), [95-97](#_ENREF_95)].

**S4.31 CYP2C9 and warfarin response**

CYP2C9 is a cytochrome P450 (CYP450) enzyme responsible for the metabolic clearance of up to 15%-20% of all drugs undergoing Phase I metabolism [[98](#_ENREF_98), [99](#_ENREF_99)]. The CYP2C9 gene, located on chromosome 10q24.1 is highly polymorphic (www.cypalleles.ki.se/cyp2c9.htm), and includes functional variants of major pharmacogenetic importance. CYP2C9*2 and CYP2C9*3, are the most common variants in Caucasians and the most extensively studied CYP2C9 alleles. Both have been shown to be significantly associated with over-anticoagulations (INR>3) in the first 2 weeks of therapy [[100](#_ENREF_100)], increased risk of bleeding in patients [[101](#_ENREF_101)] and collectively shown to explain as much as 12% of variation in warfarin dose requirements in Caucasians [[87](#_ENREF_87)]. Individuals with the *2 and *3 variants take a longer time to reach target INR on starting warfarin therapy and are therefore at increased risk of bleeding complications [[102](#_ENREF_102), [103](#_ENREF_103)]. The enzymatic activity of CYP2C9*2 and CYP2C*3 alleles are about 50% and 10% respectively of the wild type CYP2C9*1 enzyme [[73](#_ENREF_73), [104](#_ENREF_104)]. Both variants have significantly lower frequencies in African and Asian populations compared to Caucasian populations (**Table S11**) [[99](#_ENREF_99), [105](#_ENREF_105)]. Several other rare or population specific CYP2C9 variants have also been described. These include the reduced activity CYP2C9*5, *6, *8 and *11 alleles, found in populations of African descent, and CYP2C9*14 found in East Asians that are also associated with lower warfarin dose requirements in the respective populations [[96](#_ENREF_96), [106](#_ENREF_106), [107](#_ENREF_107)].

**S4.32 Strength of evidence scoring of CYP2C9 variants**

More than 57 polymorphic variants or ‘star alleles’ of CYP2C9 have been described to date (www.cypalleles.ki.se/cyp2c9.htm, updated 12/20/2012). Of the polymorphic variants or ‘star alleles’ of CYP2C9 that have been described, CYP2C9*1, CYP2C9*2, CYP2C9*3, CYP2C9*5, CYP2C9*6, CYP2C9*8, CYP2C9*11 and CYP2C9*14 have warfarin specific clinical outcomes data available and are all assigned Evidence code “1” (**Table S11).**  CYP2C9*13 is assigned Evidence code “9” since the highest evidence available is clinical evidence for other drug(s) without supporting evidence that the codon change, L90P, has broad effect on metabolism of all substrate drugs. CYP2C9*12, CYP2C9*15, CYP2C9*16 and CYP2C9*25 are assigned Evidence code “11” since the highest evidence is for molecular functional study with another drug. CYP2C9*4 is assigned evidence code “12scd” since it was detected in a single Japanese individual who required a low dose of phenytoin and the codon change is at a residue in the substrate recognition site of CYP2C9. Variants Y358C, A441A and G475G were evaluated based on their presence on the DMET-plus genechip and are assigned Evidence code “13” since they lack clinical or molecular functional data, being identified through gene sequencing studies. CYP2C9*7, CYP2C9*9 and CYP2C9*10 are assigned Evidence code “14” as they do not appear to alter CYP2C9 activity. Variant allele phenotypes, frequency, and evidence scoring are summarized in **Table S11**.

**S4.33 Warfarin-CYP2C9 genotype-phenotype interpretation**

Classification of the diploid individual to a predicted drug metabolism (dose requirement) phenotype is provided in the CYP2C9-warfarin Punnett square table (**Table S12)** based on expected CYP2C9 genotypes for variants with evidence code ≤7 (see **Table S11**) and published data. Individuals with 1 copy of the CYP2C9*2 allele (*1/*2) are considered slow metabolizers of S-warfarin; those homozygous for CYP2C9*2 (*2/*2) or who carry at least 1 copy of the CYP2C9*3 or CYP2C9*5 or CYP2C9*6 alleles are very slow metabolizers. There is insufficient published clinical data to know whether individuals carrying just one copy of CYP2C9*8, CYP2C9*11 or CYP2C9*14 (*1/*8, *1/*11, *1/*14) are ‘slow’ or ‘very slow’ metabolizers of S-warfarin, given the current data these variants are assumed to be slow metabolizers like CYP2C9*2. In general, warfarin dosing requirements decrease by CYP2C9 metabolism phenotype from EM>IM>PM, with IM and PM individuals at increased risk of bleeding [[101](#_ENREF_101)].

**S4.41 VKORC1 and warfarin response**

The vitamin K epoxide reductase complex subunit-1, encoded by the gene VKORC1 on chromosome 16p11.2, is the target through which warfarin exerts its therapeutic effect. In Caucasians there are two common VKORC1 haplotypes that explain 25% of variance in warfarin dose. The low-dose haplotype group (A) or VKORC1*2, defined by the presence of the promoter -1639G>A ‘A’ allele (and other tightly linked tag-SNPs including the 1173C>T; rs9934438), and the high-dose haplotype group (B), determined by the absence of the *2 allele (e.g. detection of the -1639G>A ‘G’ allele) [[108-111](#_ENREF_108)] (**Table S13**). Haplotype group A homozygotes (VKORC1-1639G>A, ‘AA’ genotype) are the most warfarin sensitive and trend towards a lower warfarin maintenance dose (2.7±0.2 mg per day); heterozygotes for Haplotype A and haplotype B (‘AG’ genotype) require intermediate warfarin doses (4.9±0.2mg per day) and homozygotes for haplotype B (‘GG’) require a higher relative warfarin dose (6.2±0.3 mg per day) [[108](#_ENREF_108)]. Haplotype group A VKORC1 polymorphisms lead to a more rapid achievement of a therapeutic INR, but also a shorter time to reach an INR over 4, which is associated with bleeding [[112](#_ENREF_112)]. Genetic variation in the VKORC1 gene appears to be the most important genetic factor determining variability in warfarin dose, and accounting for approximately three times the effect of variation in the CYP2C9 gene in Caucasians [[87](#_ENREF_87), [108](#_ENREF_108), [113](#_ENREF_113), [114](#_ENREF_114)]. Similar observations have been reported in Asian patients [[115](#_ENREF_115), [116](#_ENREF_116)]. In addition, VKORC1 polymorphisms are thought to explain at least in part why Asian Americans are generally more sensitive to warfarin (higher proportion of group A haplotypes) [[108](#_ENREF_108), [117](#_ENREF_117)], while African Americans are on average relatively resistant (higher proportion of group B haplotypes).

There are additional rare or population specific coding variants of VKORC1, associated with warfarin resistance (e.g. the recessive hereditary disorder, vitamin K-dependent clotting factors, combined deficiency 2 (VKCFD2)), where carriers either require higher doses or do not respond to warfarin [[118](#_ENREF_118), [119](#_ENREF_119)].

**S4.42 Strength of evidence scoring of VKORC1 variants**

VKORC1*2 is assigned evidence code “1” since there are clinical outcomes data supporting the association of this variant with reduced daily warfarin maintenance dose requirements (**Table S13**). VKORC1*3 and VKORC1*4 define subtypes of the high-dose haplotype group (B) and are assigned evidence code “14” based on the redundancy of information given the tight LD with absence of CYP2C9*2 variant. Alleles that are rare or lack sufficient evidence for an effect on drug response are assigned evidence code “11” (VKORC1 R98W) or evidence code “12” (VKORC1 L128R, R58G, V29L and V45A.Interestingly the wild type haplotype VKORC1*1 that corresponds to the reference sequence AY587020, is very rare in Caucasian and Asian populations (frequency <0.1%) but appears to be common in Africans (frequency>30%) [[108](#_ENREF_108), [110](#_ENREF_110)]. Given the haplotype structure of the gene, VKORC1*1 by definition also represents a subtype of the high-dose haplotype group (B). However given the lack of specific functional or clinical studies confirming the dosage requirement in carriers of this variant, it has been assigned an evidence code “13”. Variant allele phenotypes, frequency, and evidence scoring are summarized in **Table S13**.

**S4.43 Warfarin-VKORC1 genotype-phenotype interpretation**

A summary of the warfarin dose requirement by VKORC1 genotype, based on published data as described above is presented in **Table S14**.

**S4.51 CYP4F2 and warfarin response**

CYP4F2 is the primary human liver microsomal Vitamin K1 oxidase that removes vitamin K from the vitamin K cycling pathway [[88](#_ENREF_88)].The CYP4F2 gene is located within a cluster of cytochrome P450 genes on chromosome 19p13 (www.cypalleles.ki.se/cyp4f2.htm). A non-synonymous genetic variant of CYP4F2, V433M (rs2108622) that results in reduced enzyme activity [[88](#_ENREF_88), [120](#_ENREF_120)], has been shown to have a modest but significant impact on dose requirements for two of the most frequently prescribed coumarins, warfarin and acenocoumarol [[114](#_ENREF_114), [121-126](#_ENREF_121)]. Dosing algorithms that incorporate clinical and genetic factors such as genetic variants in VKORC1, CYP2C9 and CYP4F2, age, and weight explain up to 60% of interindividual variability in warfarin dose requirement, with CYP4F2 accounting for 1-7% of this variability [[121](#_ENREF_121), [123](#_ENREF_123), [124](#_ENREF_124)].

**S4.52 Strength of evidence scoring of CYP4F2 variants**

Two variant ‘star alleles’ for CYP4F2 (CYP4F2*2 and CYP4F2*3) have been documented to date (www.cypalleles.ki.se/cyp4f2.htm, updated 5/2008). Variant allele phenotypes, frequency, and evidence scoring are summarized in **Table S15**. CYP4F2*3 (rs2108622) that encodes the V433M codon change has been assigned an evidence code of 1 based on clinical outcomes data. CYP4F2*2 has an evidence code of 14 based on normal enzyme activity *in vitro*. At present, no other genetic variant in CYP4F2 has been associated with variability in warfarin dose requirements. Variants P13R, W12C, H343H, P55P, G93G, N112N, A116A, G185V and L278F were evaluated based on their presence on the DMET-plus genechip and are assigned Evidence code “13” since they lack clinical or molecular functional data, being identified through gene sequencing studies.

**S4.53 Warfarin-CYP4F2 genotype-phenotype interpretation**

Reduced CYP4F2 activity results in increased hepatic vitamin K levels and a requirement for a higher warfarin dose. Classification of the CYP4F2 rs2108622 genotypes to a predicted drug metabolism phenotype (**Table S16**) is guided by published data that suggests a linear additive effect of the T allele such that wild type CC individuals have a relative lower warfarin dose requirement, CT individuals have an intermediate and TT individuals require a higher warfarin dose [[123](#_ENREF_123)].

**S4.6 US Food and Drug Administration (FDA) and Other Clinical Association Guidelines**

In 2007, the FDA added pharmacogenomic information to the warfarin drug label [[127](#_ENREF_127)] and in 2010 this was updated with expected warfarin therapeutic dose range recommendations based on CYP2C9 and VKORC1 genotypes (see **Table S17**).

**S4.7 Gaps in warfarin PGx knowledge**

Despite the evidence for lower warfarin requirements in carriers of specific rare/non-Caucasian variants such as CYP2C9*8, *11 and *14 further clinical studies are needed to clarify the diplotype associated dose ranges. In addition, the functional consequence of other rare CYP2C9 variants (those with evidence code ≥8 in **Table S11**) on warfarin metabolism and therapeutic dose requirement needs to be elucidated. Further analysis is needed to clarify the clinical phenotype associated with the VKORC1*1 reference haplotype (assumed to be a high dose Haplotype B subtype). Although CYP4F2 is known to have a significant influence on warfarin dose requirement and is currently included in a number of dosing algorithms (based on clinical and genetic factors) [[95](#_ENREF_95)], there are no genotype only based therapeutic dosing guidelines that combine the effect of the three key genes associated with variable warfarin dose requirements. Inclusion of rare/non-Caucasian CYP2C9 variants and the CYP4F2 V433M variant in a genotype only based warfarin dosing guideline table would serve as a valuable update to the current FDA genotype based recommended daily therapeutic dosing ranges.

**S5.0 Codeine-CYP2D6** (Reviewed by PAG in October 2010)

**S5.1 Description and mechanism of action of codeine**

Codeine sulfate is an opioid analgesic indicated for the relief of mild to moderately severe pain where the use of an opioid analgesic is appropriate. Codeine may also be used to control a cough or diarrhea. The most frequently observed adverse reactions with codeine administration include drowsiness, lightheadedness, dizziness, sedation, shortness of breath, nausea, vomiting, sweating, constipation, and other potential adverse reactions.

Codeine elicits its analgesiceffect through its metabolite, the opiate morphine. Opiate receptors are coupled with G-protein receptors and function as both positive and negative regulators of synaptic transmission via G-proteins that activate effector proteins. Binding of the opiate stimulates the exchange of GTP for GDP on the G-protein complex. As the effector system is adenylate cyclase and cAMP located at the inner surface of the plasma membrane, opioids decrease intracellular cAMP by inhibiting adenylate cyclase. Subsequently, the release of nociceptive neurotransmitters such as substance P, GABA, dopamine, acetylcholine and noradrenaline is inhibited. Opioids also inhibit the release of vasopressin, somatostatin, insulin and glucagon. Codeine's analgesic activity is, most likely, due to its conversion to morphine. Opioids close N-type voltage-operated calcium channels (OP2-receptor agonist) and open calcium-dependent inwardly rectifying potassium channels (OP3 and OP1 receptor agonist). This results in hyperpolarization and reduced neuronal excitability.

About 70-80% of administered dose of codeine is metabolized by conjugation with glucuronic acid to codeine-6glucuronide (C6G) and via *O*-demethylation to morphine (about 5-10%) and *N*-demethylation to norcodeine (about 10%) respectively. UDP-glucuronosyltransferase (UGT) 2B7 and 2B4 are the major enzymes mediating glucurodination of codeine to C6G. Cytochrome P450 2D6 is the major enzyme responsible for conversion of codeine to morphine and P450 3A4 is the major enzyme mediating conversion of codeine to norcodeine. Morphine and norcodeine are further metabolized by conjugation with glucuronic acid. The glucuronide metabolites of morphine are morphine-3-glucuronide (M3G) and morphine-6-glucuronide (M6G). Morphine and M6G are known to have analgesic activity in humans. The principal pathways for metabolism of codeine occur in the liver, although some metabolism occurs in the intestine and brain [[128](#_ENREF_128)]. Between 0-15% of codeine is O-demethylated to morphine, the most active metabolite, which has 200 fold greater affinity for the mu opioid receptor compared to codeine. This metabolic reaction is performed by CYP2D6 [[129-131](#_ENREF_129)].

**S5.2 Pharmacogenomic studies of codeine**

CYP2D6 is a member of the cytochrome P450 gene family, a group of enzymes responsible for Phase I metabolism and elimination of endogenous substrates and a wide array of drugs such as antidepressants, neuroleptics, antiarrhytmics, analgesics, antiemetics and anticancer agents. CYP2D6 is primarily expressed in the liver and is responsible for the metabolism of 25% of all drugs on the market [[132](#_ENREF_132), [133](#_ENREF_133)]. The CYP2D6 gene is highly polymorphic with many important SNPs, haplotypes and copy number variants and is one of the most commonly studied genes in the codeine metabolism pathway. Polymorphisms that are ‘silent’ do not affect the enzyme activity and result in the expression of a protein with normal CYP2D6 activity. These functional alleles are the most common alleles and are associated with an extensive metabolizer (EM) phenotype. Poor Metabolizer (PM) alleles are non-functional or ‘Null’ alleles. These are less common and can be a whole gene deletion or nucleotide changes that result in no protein expression or inactivation of the enzyme’s activity. Another class of alleles, result from gene variants that reduce enzyme activity producing an intermediate metabolizer (IM) phenotype. An individual's highest functioning CYP2D6 allele predicts his/her phenotypic activity [[134](#_ENREF_134), [135](#_ENREF_135)]. Thus, individuals with two null alleles have impaired metabolism of CYP2D6 substrates and are classified as poor metabolizers; those with an active allele and a null allele or two active alleles have normal substrate metabolism and are classified as normal or extensive metabolizers; individuals with two low activity alleles or one low and one null allele are referred to as intermediate metabolizers since their CYP2D6 enzyme activity is between extensive and poor metabolizers. There are also a number of gene duplications and multiplications (up to 13 copies have been observed) which have been seen to occur with many different CYP2D6 haplotypes, including CYP2D6*1, CYP2D6*2, CYP2D6*4, CYP2D6*10, and CYP2D6*41 [[136](#_ENREF_136)]. Multiplications of the normal activity allele (e.g. CYP2D6*1 and CYP2D6*2) create an ultra-rapid metabolizing (UM) status, which are associated with the ultra-rapid metabolism of CYP2D6 substrates. Multiplication of null or low activity genes does not appear to alter the drug metabolizing phenotype.

There are alternative methods for classifying a metabolizer phenotype from CYP2D6 genotype. An example of a gene dose method is illustrated in Table S16, which assigns a functional allele count (activity score) to each CYP2D6 allele and provides an overall genotype score (gene dose level) which can then be translated to a CYP2D6 metabolic phenotype [[137](#_ENREF_137)]. This scoring method has shown to correlate well with CYP2D6 catalytic activity for codeine and other substrates [[137-140](#_ENREF_137)].

**S5.3 CYP2D6 and codeine response**

Since codeine is a pro-drug, and the morphine metabolite is considered to confer the majority of pain relief, it is expected that PM individuals would get little or no pain relief due to their inability to metabolize codeine into morphine. Mutliple pharmacokinetic (PK) studies have shown that PMs have a significantly lower metabolic capacity and higher dextromethorphan (substrate) mean metabolic ratio (MR) than EMs, and are unable to metabolize codeine into morphine [[139](#_ENREF_139), [141](#_ENREF_141), [142](#_ENREF_142)]. Measuring relief from pain is a very subjective measure, and pain tolerance may be a very personal factor, thus it is a difficult endpoint to measure accurately. The few studies that address differences in pain relief between PMs and non-PMs show inconsistent evidence. A randomized double-blind control trial with a 3-way cross-over design showed that PM individuals (n=9) given codeine are not as pain tolerant as EMs (n=9) [[143](#_ENREF_143)]. The EMs had a significantly longer mean time in pain tolerance than PMs. PMs did not show a significant difference in pain tolerance compared with the placebo arm. Also, PMs had only trace amounts of morphine and morphine metabolites (M3G and M6G) in their serum after codeine administration whereas EMs produced measurable amounts of all these metabolites. However, no significant differences were found in the PK parameters in codeine levels (Cmax, AUC, t1/2 or CI of codeine, not morphine) between these PMs and EMs. One study suggests that codeine itself has an analgesic effect, as some PM patients recovering from oral surgery reported pain relief at a higher codeine dose with no detectable morphine metabolites in their serum [[144](#_ENREF_144)]. Conversely, clinical studies of other opioid-related medications, which are also metabolized by the CYP2D6 enzyme, have shown significantly higher non-response rate for specific dosing of drug, need for rescue medication, and higher hospital admissions for pain management in PM patients compared to non-PMs [[145](#_ENREF_145), [146](#_ENREF_146)].

While there is not consistent evidence about whether PM patients get less pain relief from codeine, it is clear that PM individuals are unable to metabolize this codeine into morphine. Given that they may suffer the common side effects from a drug with little clinical efficacy, it would be prudent to choose alternative medications which do not require CYP2D6 metabolism for analgesia or possibly prescribe higher doses for persons with PM, and possibly an IM genotype.

Most of the serious adverse events with codeine use and CYP2D6 metabolism involve overdosing of this medicine by ultra-rapid metabolizers (UM). The duplication of *1 or *2 (active) alleles of CYP2D6 is predictive of extremely high hydroxylation capacity of the enzyme [[138](#_ENREF_138), [147-149](#_ENREF_147)] showed that CYP2D6 functional allele duplications are associated with PK differences and posited that UMs may experience differences in codeine metabolism with the potential for intoxication effects when compared to EMs. In a CYP2D6 substrate-tested group, carriers of 3 or more copies of functional CYP2D6 alleles had significantly lower MR than carriers of 2 functional alleles [[148](#_ENREF_148)]. The effects of codeine in UM may be particularly toxic in susceptible populations, such as renal failure patients, respiratory-compromised patients, and infants (exposed to codeine through breast milk) who are unable to withstand high doses of morphine production. The UM phenotype has been reported in several case reports of morphine intoxication and even death in susceptible individuals, such as infants whose UM mothers took codeine while breastfeeding [[150](#_ENREF_150), [151](#_ENREF_151)]. Other case studies reported adverse effects in UM adults, including a woman who suffered pain in the epigastrum [[152](#_ENREF_152)] and a man with bilateral pneumonia who suffered from respiratory depression after treatment with codeine and had high levels of morphine in his serum [[153](#_ENREF_153)].

Aside from case reports of morphine intoxication in UMs, one case-control study has been published examining the clinical effect of codeine given to women after childbirth who were breastfeeding their children [[150](#_ENREF_150)]. Out of 72 mother-child pairs, 17 infants experienced respiratory distress symptoms. Two of the 17 affected and 1 of the 55 unaffected mother-child pairs had duplication of an active CYP2D6 allele (*1 or *2). Given that most of the breastfed infants who experienced respiratory distress had mothers who were not classified as UMs, there are likely other factors involved in this ADR. These could include abnormalities in other genes critical for codeine metabolism, as well as varying maternal intake of codeine. A small study by Lotsch et al. highlights potential problems with UM phenotyping, as only 50% of individuals with codeine UM phenotypes (n=8) presented with CYP2D6 gene duplications in *1 or *2 alleles [[141](#_ENREF_141)]. This is some evidence that UM phenotypes are missed by genotyping for duplication of CYP2D6 functional alleles even if the causes behind this misclassification could be due to genotyping assays used, unknown increased activity CYP2D6 alleles or other genetic variants involved with this phenotype. Clinical outcomes studies are summarized in **Table S19**.

**S5.4 Strength of evidence scoring of CYP2D6 variants**

More than 105 polymorphic variants or ‘star alleles’ of CYP2D6 have been described to date (www.cypalleles.ki.se/cyp2d6.htm, updated 2/2013). Of the variants reviewed in this report, CYP2D6*1, *2, *3, *4, *5, *29 and *2×N have clinical outcomes data and are all assigned evidence code “1”. CYP2D6*1×N is supported by PK data and is assigned evidence code “2”. CYP2D6*4×N is assigned an evidence code of “4” based on in vitro molecular functional studies. CYP2D6*6, *7, *10, *14, *21, *41 and *10×N are assigned evidence code 5 based on clinical evidence for another drug along with molecular data that supports their effect on enzyme function. CYP2D6*9, *17, *36, *40, *42, *44, *56 and *4×N have PK/PD evidence for another drug as well as molecular data that supports their effect on enzyme function and are assigned evidence code “6”. Variants with insufficient evidence for influence on codeine response include: CYP2D6*18 (evidence code 10), *12 and *15 (evidence code 11), *8, *11, *19, *20, *38 (evidence code 12), *65 (evidence code 13) and *39 (evidence code 14). **Table S20** provides a summary of the functional activity, frequency and evidence scoring of CYP2D6 variants.

**S5.5 Codeine-CYP2D6 genotype-phenotype interpretation**

For CPMC purposes, the following general rule is applied for assigning phenotype to genotype for CYP2D6: First, CYP2D6 alleles are classified according to their predicted metabolic functional activity (normal, increased, and decreased or no (null allele) enzymatic function) according to evidence in the literature (**Table S20**). Second, diplotype-phenotype classifications are assigned as: extensive metabolizer (EM), defined as two alleles conferring normal or near-normal activity; intermediate metabolizers (IM) as those homozygous for a decreased activity allele or heterozygous for decreased activity allele and null allele; poor metabolizers (PM) are those with two null activity alleles; and ultra-rapid metabolizers (UM) as those with greater than two copies of a normal activity allele through gene duplications/multiplications of a normal activity allele. Multiplication of null and low activity genes does not appear to alter the drug metabolizing phenotype. In general, IM or PM classification is associated with significantly lower codeine metabolism (or none for PM) into morphine than EMs, which may also be associated with little pain relief (analgesic effect). Those with duplicate PM alleles (*4) or IM alleles (*10 or *41) have not shown much evidence of phenotype change in codeine metabolism, and are predicted to have the phenotype of the highest functioning allele in the genotype. The CYP2D6-codeine genotype-phenotype interpretation for all the expected genetic variant combinations (diplotypes) is provided in the extended Punnett square table (**Table S21**).

**S5.6 FDA and Other Clinical Association Guidelines**

As adapted from the codeine drug label: “Codeine is an opioid analgesic pro-drug, typically used for pain relief. It is metabolized by CYP2D6 into morphine, which is the active drug form. CYP2D6 PMs and UMs may experience different efficacy. Mothers who are UMs and breast-feeding should be particularly aware of potential danger to breastfed infants. UMs convert codeine into its active metabolite, morphine, more rapidly and completely than other people. This rapid conversion results in higher than expected serum morphine levels. Even at labeled dosage regimens, individuals who are ultra-rapid metabolizers may experience overdose symptoms such as extreme sleepiness, confusion, or shallow breathing; and potentially dangerously high serum morphine levels can be delivered to breastfed infants of UM mothers. Therefore, maternal use of codeine can potentially lead to serious adverse reactions, including death, in nursing infants.”

**S5.7 Gaps in codeine PGx knowledge**

There is a limited number and size of studies relating ADRs and CYP2D6 UM metabolizer status. Given the serious consequences of the intoxification effects especially in infants of breastfed mothers the recommendations in this report and by the FDA are based on a small number of case reports. Therefore, further studies are needed to elucidate the broader effect of UM metabolizer status and consequences of codeine use in both adult and children. There are a number of CYP2D6 alleles that lack sufficient functional and clinical response data (those with evidence scores ≥ 2 in Table S19) that need further study. In addition, the functional consequence of >2N copies of intermediate (IM) CYP2D6 alleles in individuals with gene multiplications needs to be determined. This should be evaluated for all intermediate alleles where gene copy number increases have been reported (e.g. *10×N and *41×N). Given the structural complexity of the CYP2D6 gene, accurate genotyping and interpretation of genetic results may be challenging for some diplotypes. For example, where a gene copy number increase is detected in heterozygotes for a normal activity allele and a reduced activity allele, the functional consequence will depend on which allele is multiplicated. Genotyping assays need to also accurately detect gene deletions given the relatively high frequency of the CYP2D6*5 allele. There is also a complex linkage disequilibrium (LD) pattern between specific markers in some populations. For example, the intermediate activity *10 allele, common in Asian populations is in high LD with the CYP2D7 exon 9 gene conversion *36 (also known as *10C) which is associated with low or null activity. Therefore both variants should be interrogated to more accurately assign metabolizer status as an IM (e.g. *10/*10 individual) vs. a possible PM (*10C/*10C).

**S6.0 Thiopurines-TPMT** (Reviewed by PAG in October 2010)

**S6.1 Description and mechanism of action of thiopurines**

The thiopurine drugs—azathioprine (AZA), 6-mercaptopurine (6-MP), and 6-thioguanine (6-TG)—are cytotoxic agents commonly used in the treatment of chronic inflammatory diseases, hematological malignancies, and to prevent tissue rejection following transplantation.

The thiopurines are inactive prodrugs that require intracellular activation, catalyzed by multiple enzymes, to exert cytotoxicity [[154](#_ENREF_154)]. The first step in AZA activation is driven by exposure to sulphydryl containing compounds in the plasma and tissues. This results in a primarily non-enzymatic conversion of AZA to 6-MP and an imidazole group [[155](#_ENREF_155)]. 6-MP is further metabolized and converted into thioguanosine monophosphate (TGMP). Subsequently, TGMP is further metabolized by a series of kinases and reductases into thioguanine nucleotide diphosphates (TGDP) and triphosphates (TGTP). The active metabolite TGTP can also induce apoptosis in activated T cells by inhibition of Rac1 [[156](#_ENREF_156)]. This mechanism may be particularly important in autoimmune and chronic inflammatory diseases that rely on pathogenic memory T-cells.

The activation pathway that leads to biotransformation of 6-MP and 6-TG to active metabolites is in competition with inactivation pathways catalyzed by xanthine oxidase (XDH) or the polymorphic thiopurine methyltransferase (TPMT). TIMP, an intermediate in the conversion of 6-MP to TGMP, can also act as a substrate for TPMT, leading to the production of S-methyl-thioinosine 5-monophosphate (6-Me-Thio-IMP) which is a strong inhibitor of purine de novo synthesis (PDNS) [[157](#_ENREF_157), [158](#_ENREF_158)]. Inhibition of PDNS is another way in which AZA and 6-MP exert a cytotoxic effect. Inhibition of PDNS is an established method for achieving immunosuppression and blocking proliferation of various types of lymphocyte cell lines [[159](#_ENREF_159)].

AZA and 6-MP, have a relatively narrow therapeutic index, are not effective in one-third of patients and up to one-fifth of patients discontinue thiopurine therapy due to adverse reactions. Inter-individual differences in both efficacy and toxicity are largely explained by differences in thiopurine metabolism (reviewed in [[155](#_ENREF_155), [160](#_ENREF_160)]). In general, TPMT deficiency is associated with early and more severe myelotoxicity; and high TPMT activity may be associated with poor treatment response [[161](#_ENREF_161), [162](#_ENREF_162)] or higher risk of relapse [[163](#_ENREF_163)], with higher doses required to achieve therapeutic effect [[164](#_ENREF_164)].

**S6.2 Pharmacogenomic studies of thiopurines**

The four key enzymes that have been investigated for their role in modulating the therapeutic and adverse effects of thiopurines include: Thiopurine methyl transferase (TPMT), xanthine oxidase (XDH), inosine triphosphate pyrophosphatase (ITPA), and glutathion-S-transferase (GST). There have only been a small number and size of studies conducted so far for XDH. Further research needs to be conducted to elucidate whether variation in XDH plays a role in modulating risk of thiopurine adverse reactions [[165](#_ENREF_165), [166](#_ENREF_166)]. There has also been an inconsistency in the association of IPTA with AZA related allergic reactions, which results in the need for additional larger well-designed studies to clarify this potential relationship. However, despite the literature suggesting consistency in AZA-induced myelosuppression, it seems variants in ITPA do not appear to play a significant role [[167-170](#_ENREF_167)]. There appears to be an important and clinically significant association of GST modulating thiopurines, but further studies are required to confirm these findings and to assess the utility of prior-to treatment genotyping of GST genes to prevent AZA-related ADRs [[171-175](#_ENREF_171)]. The most intensively studied genetic variants with regard to their clinical implications and/or molecular mechanisms related to thiopurine drug response are in TPMT (described in the sections below).

**S6.3 TPMT and thiopurine response**

Deficiency of TPMT enzyme activity was first described 30 years ago by Weinshilboum and Sladek [[176](#_ENREF_176)] who observed that 1 in 10 Caucasians showed intermediate and 1 in 300 showed deficient TPMT activity. It has been this association that propelled the adoption of TPMT testing into clinical practice to identify individuals deficient in TPMT and avoid the associated serious adverse drug reaction. As such, the drug labels for AZA, 6-MP and 6-TG include a warning about TPMT activity and risk of ADRs, with a recommendation that TPMT activity is tested either by direct enzyme activity or genotype assessment prior to thiopurine drug commencement and substantial dosage reductions be considered in patients that are deficient [[177-179](#_ENREF_177)]. Numerous small and large studies in various patient populations have demonstrated significant association between TPMT metabolic phenotype and/or genotype both with adverse reactions (such as myelosuppression) and clinical response to thiopurine therapy (some examples are described below and in **Table S22)**. Nevertheless, TPMT deficiency only accounts for about 25% of all cases of thiopurine-induced myelosuppression and TPMT testing cannot predict a number of other adverse reactions associated with thiopurine therapy including allergic reactions, hepatotoxicity, pancreatitis, nausea and vomiting [[180](#_ENREF_180)].

TPMT variants associated with thiopurine drug response include TPMT*2, *34A, *3B, and *3C. TPMT*3A, defined by the presence of two non-synonymous variants Ala154Thr and Tyr240Cys, is the most common variant allele associated with low TPMT activity in Caucasians (frequency approximately 5%) [[181-184](#_ENREF_181)]. TPMT*3B contains only the exon 7 SNP (Ala154Thr) and occurs rarely while TPMT*3C contains only the exon 10 SNP (Tyr240Cys), occurs rarely in Caucasian populations while representing the most common variant allele in East Asian (~2%) and African populations (~5%) [[181](#_ENREF_181), [185-187](#_ENREF_185)]. TPMT*2, results in an Ala80Pro amino acid substitution and is much less common than either TPMT*3A or TPMT*3C [[181](#_ENREF_181), [186](#_ENREF_186), [188](#_ENREF_188)]. The molecular mechanism for reduced activity of the TPMT*2 and TPMT*3A, *3B and *3C variants is due to enhanced degradation of TPMT allozymes encoded by these variants [[189-191](#_ENREF_189)]. There are other very rare alleles that have been observed in single individuals but not observed in population screens, which include TPMT*3D, *5, *7, *10-*15, *18-*20, *22, *23, and*25. Other rare, low activity alleles such as TPMT*4, *6, *16 and *21 have been identified at very low frequencies in Caucasian (*4, *16 and *21) and Asian (*6) populations [[192-195](#_ENREF_192)].

A number of meta-analyses of published studies have been carried out to further examine the relationship between TPMT polymorphisms and thiopurine toxicity [[180](#_ENREF_180), [196](#_ENREF_196)]. Dong and colleagues evaluated nine studies that assessed the relationship between TPMT variants and thiopurine-induced toxicity in a total of 1,309 IBD patients [[196](#_ENREF_196)]. The meta-analysis showed a 2.93-fold (95% CI: 1.68-5.09, P = 0.0001) increase in the incidence of TPMT gene mutations (includes all those homozygous or heterozygous for TPMT*3A and *3C variants) in IBD patients with thiopurine-induced overall ADRs (bone marrow toxicity (BMT), hepatotoxicity, pancreatitis, gastrointestinal disturbances and other adverse reactions leading to reduction of thiopurine dose or discontinuation of therapy); and 5.93-fold (95% CI: 2.96-11.88, P < 0.00001) for BMT, compared with controls. They also showed that there was no support for an association of TPMT variants with other specific thiopurine-induced ADRs such as hepatotoxicity (P=0.43) and pancreatitis (P=0.98). In another larger meta-analysis by Higgs et al., the authors aimed to quantify the increased risk of thiopurine-induced myelosuppression in patients with intermediate TPMT activity [[180](#_ENREF_180)]. They conducted a systematic review of published studies that explored the relationship between thiopurine induced hematological ADRs and either TPMT genetic variants (genotype) or TPMT enzyme activity (phenotype) in patients on thiopurine therapy [[180](#_ENREF_180)]. The summary odds ratio for leucopenia in patients with either intermediate TPMT activity or heterozygous for a TPMT variant allele that confers reduced activity was 4.19 (95% CI: 3.20-5.48; P<0.00001) compared to wild-type TPMT patients. Although a four-fold increased risk of leucopenia in patients with one TPMT reduced activity variant allele or intermediate activity was demonstrated, the authors caution interpretation of the magnitude of the odds ratio in part because of the presence of heterogeneity. In addition, given that 1) the increased risk was for mild as opposed to severe leucopenia, and 2) lymphopenia is a normal response to thiopurine-induced immunosuppression, and 3) individuals with intermediate TPMT activity have raised levels of active metabolite, it remains unclear as to whether modest lymphopenia reflects a clinically relevant adverse event or is an indication of effective thiopurine treatment [[180](#_ENREF_180)].

A number of studies have tried to evaluate the effectiveness of TPMT screening in guiding treatment dosing regimens [[161](#_ENREF_161), [162](#_ENREF_162), [197-201](#_ENREF_197)]. These studies have led to some recommendations for TPMT-guided thiopurine treatment. A general consensus opinion is that: (a) patients with normal or high TPMT activity should be administered a full dose of thiopurine drug at the outset [[197](#_ENREF_197)]; (b) patients with intermediate TPMT activity can be treated with fewer side effects by reducing the standard dose by 50-67% [[201-203](#_ENREF_201)]; (c) treatment is generally contraindicated for patients with deficient TPMT activity (homozygous for reduced activity TPMT variant alleles), although treatment with a reduced regime of less than 20% standard dose has been successful in some patients [[198](#_ENREF_198), [199](#_ENREF_199), [201](#_ENREF_201)]; (d) reduction of thiopurine dose to 25% of normal is recommended in cases where allopurinol is co-prescribed [[161](#_ENREF_161), [201](#_ENREF_201)]; and (e) regular monitoring of full blood count and liver function tests should still be carried out since the majority of adverse reactions to thiopurine drugs, including myelosuppression, are not explained by TPMT activity [[197](#_ENREF_197)].

**S6.4 Strength of evidence scoring of TPMT variants**

The human TPMT gene consists of 10 exons and spans 34kb of DNA on chromosome 6p22.3 [[183](#_ENREF_183)], and has a pseudogene located on chromosome 18 [[204](#_ENREF_204)]. Twenty-nine variant alleles have been identified [[205](#_ENREF_205), [206](#_ENREF_206)], the majority involving nonsynonymous SNPs and are associated with decreased activity in vitro [[182](#_ENREF_182), [207](#_ENREF_207), [208](#_ENREF_208)].

Of these variants TPMT*1, TPMT*2, TPMT*3A, TPMT*3B, and TPMT*3C have clinical outcomes data available and are all assigned evidence code “1” (**Table S23**)**.** TPMT*4 has been assigned an evidence code “2” since the variant lacks clinical outcomes data, but the literature provides pharmacokinetic evidence. TPMT variants with insufficient evidence include: TPMT*(GCC)5/7 with preliminary molecular functional data only assigned evidence code “11”; TPMT*3D, *5-*7, *10-*18, *20-*23 and *25,have been assigned evidence code “12” since they are rare variants each identified in a single individual with limited functional data on the effect of the variant; and TPMT*8, *9 and *24, as they do not appear to alter TPMT activity. **Table S23** provides a summary the metabolic phenotypes, frequency and evidence scoring of the TPMPT variants.

**S6.5 Thiopurine-TPMT genotype-phenotype interpretation**

Classification of the diploid individual to a predicted drug metabolizer status and associated drug response (ADR) phenotype is provided in the Punnett square table based on expected TPMT genotypes and published data (see **Table S24** below**)**.

**S6.6 FDA and Other Clinical Association Guidelines**

AZA carries a black box warning regarding the risk of neoplasia following chronic immunosuppression due to AZA use. Individuals using AZA should be advised of the mutagenic potential and of the risk for hematologic toxicities.

Adapted from the package labels of AZA [[177](#_ENREF_177)], 6-MP [[178](#_ENREF_178)], and 6-TG [[179](#_ENREF_179)], homozygous-deficient patients (two non-functional TPMT alleles) given usual doses of mercaptopurine can accumulate excessive cellular concentrations of active thioguanine nucleotides that predispose them to toxicity. Heterozygous patients with low or intermediate TPMT activity accumulate higher concentrations of active thioguanine nucleotides than people with normal TPMT activity and are more likely to experience toxicity. TMPT genotyping or phenotyping (red blood cell TPMT activity) can identify patients who are homozygous deficient or have low or intermediate TPMT activity. Caution must be used with metabolic phenotyping since some co-administered drugs can influence measurement of TPMT activity in the blood, and recent blood transfusions will misrepresent a patient’s actual TPMT activity. Dosage reduction is recommended in patients with reduced TPMT activity. Early drug discontinuation may be considered in patients with abnormal CBC results that do not respond to dose reduction. TPMT testing cannot substitute for complete blood count (CBC) monitoring in patients receiving AZA.

**S6.7 Gaps in thiopurine PGx knowledge**

While there is clinically relevant data for TPMT*1, *3A-*C further clinical evidence is needed to confirm the predicted effect of the rare *4 variant on thiopurine drug response. In addition, the functional consequence of other rare TPMT variants (those with evidence code ≥8 in **Table S23**) on thiopurine therapeutic response needs to be elucidated. Given the apparent high rate of rare possibly “private” mutations reported in the TPMT gene, the impact of TPMT variants on thiopurine ADRs may be underestimated by studies that only interrogate the established more common variants. The clinical implications of TPMT intermediate metabolizers may need further study. Although there is a significant increased risk of leucopenia in IM patients this appears to be primarily mild leucopenia and it remains unclear as to whether this is a clinically relevant adverse event or an indication of effective thiopurine treatment [[180](#_ENREF_180)].

**S7.0 Simvastatin-SLCO1B1** (Reviewed by PAG in March 2012)

**S7.1 Description and mechanism of action of simvastatin**

Statins act primarily at the site of cholesterol synthesis in the liver [[209](#_ENREF_209)] to inhibit hydroxymethylglutaryl (HMG) CoA reductase, which causes a reduction in hepatic cholesterol levels, increased hepatic uptake of cholesterol, and lower plasma levels of cholesterol and low-density lipids (www.DrugBank.ca). Statins are used to treat hypercholesterolemia and dyslipidemia for the prevention of heart disease, stroke, and myocardial infarction.

**S7.2 Pharmacogenomic studies of simvastatin**

Variation in pharmacokinetic genes influences simvastatin disposition, response, and side effects. Genetic associations have been found for CYP3A4, CYP3A5, CYP2D6, ABCB1, and SLCO1B1. The most substantial pharmacogenetic effects of SCLO1B1 are detailed in the next section, while evidence for the other genes is briefly described here.

***ABCB1*** Simvastatin is an ABCB1 substrate [[210](#_ENREF_210)] and also inhibits ABCB1 [[211](#_ENREF_211)]. Variants in and near ABCB1, including rs378924, rs1922242, 1236C>T (G412G), 2677G>T/A (A893S/T), and 3435C>T (I1143I), have been significantly associated with greater simvastatin levels, improved response, and fewer side effects. In a study of 28 Caucasians treated with simvastatin and atorvastatin, carriers with the variant 1235-2677-3435 TTT/TTT diplotype showed modest but significantly higher simvastatin AUC compared to carriers with the reference CGC/CGC diplotype [[212](#_ENREF_212)]. In a study of 116 Caucasians, carriers with variant 1236C>T and 2677G>T/A genotypes showed significantly improved lipid and cholesterol response; and significantly fewer myalgia cases were seen among carriers of 1236, 2677, and 3435 variants and the variant 1235-2677-3435 T-nonG-T haplotype [[213](#_ENREF_213)]. In a case-control study of 1,885 Caucasians given multiple statins including simvastatin, risk of myocardial infarction was significantly reduced in carriers of rs378924 and rs1922242 variants, while the variant 1235-2677-3435 TTT haplotype showed a non-significant trend for improved response [[214](#_ENREF_214)].

***CYP3A4 and CYP3A5*** Both simvastatin and its active metabolites, which account for the main effects of simvastatin, are substrates for CYP3A4 and CYP3A5 [[215](#_ENREF_215)]. Carriers of the poor metabolizer status CYP3A5*3/*3 genotype showed significantly improved response and greater risk of adverse drug reactions [[216-219](#_ENREF_216)]. Significantly improved response was also seen in carriers of CYP3A4*4 [[220](#_ENREF_220)] and *22 [[220](#_ENREF_220), [221](#_ENREF_221)] variants. Furthermore, this association was found after adjusting for the influence of CYP3A4*1B and CYP3A5*3/*3 [[221](#_ENREF_221)]. No association was found between the high-activity CYP3A4*1B variant and simvastatin efficacy [[213](#_ENREF_213)] or intolerance or discontinuation due to adverse events [[222](#_ENREF_222)].

***CYP2D6*** Although CYP2D6 is not known to metabolize simvastatin [[223](#_ENREF_223)], two studies showed simvastatin response was associated with CYP2D6 variants [[224](#_ENREF_224), [225](#_ENREF_225)], while other studies showed no support for the role of CYP2D6 genetics in simvastatin metabolism or response [[222](#_ENREF_222), [223](#_ENREF_223), [226](#_ENREF_226)].

**S7.3 SLCO1B1 and clinical outcomes of simvastatin**

Altered simvastatin efficacy and tolerability are associated with SLCO1B1 variation. SLCO1B1 mutations influence statin class drugs differently according to their unique physicochemical characteristics. The V174A (521T>C) variant, for example, leads to a greater increase in the systemic exposure of simvastatin than atorvastatin, pravastatin, or rosuvastatin [[214](#_ENREF_214), [227](#_ENREF_227)]. To account for these differences within the drug class, data for statins other than simvastatin in this report are regarded as ‘other drug’ in scoring the evidence. Two non-synonymous variants, SLCO1B1 V174A (521T>C) and N130D (388A>G) have been evaluated more extensively than other SLCO1B1 variants for association with simvastatin therapeutic outcome. The data show consistent significant evidence for association of V174A with increased risk of ADR (statin induced myopathy) but ambiguous data on simvastatin response (efficacy). The data for N130D are generally more ambiguous for prediction of both efficacy and tolerance [[214](#_ENREF_214), [217](#_ENREF_217), [228-232](#_ENREF_228)], with some evidence for increased efficacy in carriers of the variant allele. Given that V174A and N130D are harbored together in at least seven common haplotypes at up to 16% frequency for a given haplotype, to discern the effects of different SLCO1B1 variants as they naturally occur in discrete haplotypes, a summary of the phenotypic data for multi-SNP haplotypes and key variants is given in **Table S25**, and used to determine the evidence scores of SCLO1B1 alleles in **Table S26**.

There is a relationship between greater hepatic uptake, lower systemic exposure, and greater likelihood of drug response; while lower hepatic uptake and increased systemic levels lead to increased risk of statin-induced myopathy. With some exceptions, research shows V174A and V174A-harboring haplotypes are significantly associated with decreased statin uptake activity [[231-237](#_ENREF_231)], increased side effects[[222](#_ENREF_222), [228](#_ENREF_228), [229](#_ENREF_229), [238](#_ENREF_238)], and decreased efficacy [[217](#_ENREF_217), [229](#_ENREF_229), [239](#_ENREF_239), [240](#_ENREF_240)]. However, three other studies show no significant allelic association for V174A with statin efficacy [[216](#_ENREF_216), [217](#_ENREF_217)]. Furthermore, the *15 haplotype, which harbors N130D-V174A in cis, showed significantly decreased uptake activity for statins [[231-234](#_ENREF_231)], but opposite to the expected relationship, significantly decreased risk of simvastatin-induced myopathy (p=0.03), and a non-significant trend toward less risk of myopathy for the V174A-L643F haplotype (p=0.06) in a case-control study of 172 Europeans [[229](#_ENREF_229)].

Considering the V174A mutation only, the effect on simvastatin response (efficacy) has some conflicting evidence. In a study of 16,643 Europeans, LDL-cholesterol reduction was significantly associated with 1.28% less efficacy per copy of the V174A allele (p<0.0001) based upon joint genotype testing with the N130D variant [[229](#_ENREF_229)]. However, no significant association for simvastatin response was found in two other studies. One study of 291 Caucasians with prior myocardial infarction showed no association between the V174A variant and HDL-cholesterol reduction in patients taking 40 mg daily simvastatin [[216](#_ENREF_216)]; and V174A showed no significant allelic association with changes in triglycerides, HDL-cholesterol and LDL-cholesterol in 2,454 Caucasians given simvastatin.

Despite discordance in efficacy results for the V174A variant, the data is more consistent for risk of simvastatin-induced adverse drug reactions, sufficient for assigning evidence code “1” to the V174A allele. V174A showed significant allelic association with increased risk of myopathy with simvastatin in 20,837 combined patients in two studies from the United Kingdom [[228](#_ENREF_228), [229](#_ENREF_229)]. In 4,196 type II diabetics from Scotland, the V174A variant was associated with 2-fold increased risk of drug intolerance (p=0.043) [[228](#_ENREF_228)]; and in a case-control study of 175 Europeans from the United Kingdom, the risk of simvastatin-induced myopathy was estimated 4.5-fold greater in heterozygotes, and 16.9-fold greater in homozygote carriers (p=2E-9) in patients given 80 mg simvastatin [[229](#_ENREF_229)]. In a study of hypercholesterolemic subjects taking multiple statins, the V174A variant was significantly associated with drug intolerance (drug discontinuation, myalgia, and elevated creatine kinase) in 99 subjects with statin-induced adverse events, with the greatest risk for simvastatin [[222](#_ENREF_222)]. To minimize adverse drug reactions, the estimated maximum simvastatin dose is 80mg for 174V/V (521T/T) wild type homozygotes, 40mg for 174V/A (521T/C) heterozygotes, and 20mg for 174A/A (521C/C) variant homozygotes [[241](#_ENREF_241)].

The data for the N130D variant is mixed for both the risk of adverse reactions and efficacy of simvastatin. The N130D (388A>G) variant is significantly associated with lower simvastatin intolerance in 4,196 diabetics from Scotland (OR=0.71, p=0.026) [[228](#_ENREF_228)], but no significant difference simvastatin-induced myopathy in a case-control genome-wide association study of 175 European subjects [[229](#_ENREF_229)]. Simvastatin response was significantly improved for 16,643 Caucasians, in joint genotype testing of the N130D and V174A alleles, with 0.62% more LDL-cholesterol reduction per copy of N130D allele (p=0.0005) [[229](#_ENREF_229)]; but two other studies in 2,353 combined patients showed no significant effect of the N130D variant on simvastatin efficacy. In a study of 1,885 Caucasians, the adjusted odds ratio of statin-mediated efficacy for incidence of myocardial infarction was unchanged for 1,014 carriers of zero copies (OR=0.47, 0.36-0.63), compared to 720 carriers of one copy (OR=0.35, 0.25-0.49), and 151 carriers of two copies of the N130D variant (OR=0.43, 0.21-1.53) [[214](#_ENREF_214)]. In 468 subjects taking simvastatin, response was not significantly altered by the N130D variant [[217](#_ENREF_217)]. Similarly, carriers of the *1B allele, carrying only the N130D variant, showed discordant data for SLCO1B1 substrate activity. Pravastatin activity was increased in one study [[230](#_ENREF_230)], but three other studies showed no significant difference in activity for pravastatin [[231](#_ENREF_231)], rosuvastatin [[232](#_ENREF_232)], and a probe substrate [[230](#_ENREF_230)].

Several SLCO1B1 alleles have not been evaluated for clinical effects, but show reduced activity for pravastatin or rosuvastatin: *1C, *2, *3, *5, *6, *7, *12, *13, *16’, *17’’, *18’ and g.-11187G>A [[230-232](#_ENREF_230), [235](#_ENREF_235), [236](#_ENREF_236)]. The R580X variant shows decreased SLCO1B1 expression [[242](#_ENREF_242)]. The *9 allele shows reduced activity for rosuvastatin [[232](#_ENREF_232)] and a probe substrate [[230](#_ENREF_230)], but no effect on pravastatin efficacy in a study that had only one carrier for comparison [[234](#_ENREF_234)]. The L543W allele shows increased risk of adverse reactions to pravastatin [[243](#_ENREF_243)]. Other SLCO1B1 alleles show no change in statin activity: *1J, *4, *5B, *8, *10, *11, *14, *16’’, *18’’, *19, and *20 [[232](#_ENREF_232), [235](#_ENREF_235)]; and a few of the variants within these haplotypes showed no allelic association to clinical outcomes. For example, the P155 variant in SLCO1B1*4 and *14 showed no association with risk of simvastatin-induced myopathy [[229](#_ENREF_229)]; and in terms of statin response (lipid profile improvement), no association was found for L643F, G488A, P155T, or F73L [[217](#_ENREF_217)]. Finally, other SLCO1B1 alleles have yet to be functionally characterized: *16’’, *18+F199F, *21+L191L, H9-C, H12-C, H10-C, and H11-C [[219](#_ENREF_219), [244](#_ENREF_244)]. Clinical outcomes studies for simvastatin and SLCO1B1 are summarized in **Table S26**, including studies with other statins that inform the effects of particular haplotypes.

**S7.4 Strength of evidence scoring of SLCO1B1 variants**

More than 20 common coding single nucleotide polymorphisms are described for SLCO1B1 (according to dbSNP). Functional variants, including some with opposite phenotypes, are harbored in phase on the same chromosome in haplotypes. The star allele designation for SLCO1B1 haplotypes is not unified in the literature by different investigators, thus are designated in this report with an increasing number of prime marks per increasing number of variants that define the haplotype.

Unrelated to the haplotypic context in which it may be harbored, the V174A (521T>C) mutation shows consistent allelic association to increased risk of simvastatin-induced adverse reactions, thus is assigned Evidence code “1”. The SLCO1B1*3 and *6 alleles are predicted to show increased risk of adverse events based upon in vitro activity data and the location of these mutations in highly conserved sequence. The SLCO1B1*6 allele is assigned Evidence code “4scd”. The SLCO1B1*3 allele is assigned Evidence code “7scd”. Other SCLO1B1 alleles show less substantial evidence for a genetic phenotype related to simvastatin. The SLCO1B1*7 and D655G alleles are assigned Evidence code “8”. The SLCO1B1*16’, P336R, and -11187G>A alleles are assigned Evidence code “10”. The SLCO1B1*18’ variant is assigned Evidence code “11”. The SLCO1B1*1C, L193R, L543W alleles are assigned Evidence code “12”. The C485F, R580X, I211M, C613R, and L626X are assigned Evidence code “13”. The SLCO1B1*2, N130D, P155T (*4), D465G (*8), G488A (*9), L191L (*1J), E667G (*11), F199F, and L643F alleles are assigned Evidence code “14”. Variant phenotypes, frequency, and evidence scoring are summarized in **Table S27**.

**S7.5 Simvastatin-SLCO1B1 Genotype-Phenotype Interpretation**

The predicted phenotype of a particular diploid individual is based upon available data from published studies, based upon the combined effect of inherited SLCO1B1 alleles. Carriers of the V174A variant show increased risk of simvastatin-induced myopathy, while carriers of low-activity transporter alleles (SLCO1B1*3 and *6) are predicted to show increased risk of adverse events. The phenotypic outcome of combined alleles is estimated based upon known or predicted data. Genotype-phenotype interpretation for included SLCO1B1 genotypes are summarized in the extended Punnett square (**Table S28**).

**S7.6 FDA and Other Clinical Association Guidelines**

The Federal Drug Administration issues a list of pharmacogenomic biomarkers to be used to identify drug responders versus non-responders, avoid adverse drug events, and provide dosing guidelines for affected populations. The simvastatin drug label [[245](#_ENREF_245)], updated on 10/09/2007 does not include pharmacogenomics data for any gene related to drug dosing or risk of adverse drug reactions. However, the following drug label comments refer to risk of myopathy and dosing. In a clinical trial database in which 41,050 patients were treated with simvastatin with 24,747 (approximately 60%) treated for at least 4 years, the incidence of myopathy was approximately 0.02%, 0.08% and 0.53% at 20, 40 and 80 mg/day, respectively. In these trials, patients were carefully monitored and some interacting medicinal products were excluded. Due to the increased risk of myopathy, including rhabdomyolysis, associated with the 80 mg dose of simvastatin, patients unable to achieve their LDL-C goal utilizing the 40 mg dose of simvastatin should not be titrated to the 80 mg dose, but should be placed on alternative LDL-C-lowering treatment(s) that provides greater LDL-C lowering.

**S7.7 Gaps in simvastatin PGx knowledge**

Simultaneous genotyping of multiple variable sites is required to avoid potential gaps of information in interpreting simvastatin pharmacogenetic data. Simvastatin tolerance related to the N130D SNP depends upon the haplotypic context. The SLCO1B1 N130D mutation is harbored in haplotypes with both increased transporter activity (SLCO1B1*1B) and decreased transporter activity (e.g. SLCO1B1*15, *17, *18’). The N130D variant allele frequency is 0.44, while the SLCO1B1*1B allele frequency is only 0.08 in Caucasians. Thus, interrogation of SLCO1B1 variants commonly in phase with N130D, such as V174A, is needed to differentiate patients with increased risk of statin-induced myopathy from those with decreased risk of adverse reactions (N130D only carriers). Other recently identified SLCO1B1 haplotypes (e.g. SLCO1B1*10, *1C, H9-12-C) have yet to be functionally characterized or have not been clinically evaluated.

**REFERENCES**

1. Steinhubl SR, Moliterno DJ: **The role of the platelet in the pathogenesis of atherothrombosis.** *American journal of cardiovascular drugs : drugs, devices, and other interventions* 2005, **5:**399-408.

2. Brandt JT, Close SL, Iturria SJ, Payne CD, Farid NA, Ernest CS, 2nd, Lachno DR, Salazar D, Winters KJ: **Common polymorphisms of CYP2C19 and CYP2C9 affect the pharmacokinetic and pharmacodynamic response to clopidogrel but not prasugrel.** *Journal of thrombosis and haemostasis : JTH* 2007, **5:**2429-2436.

3. Kazui M, Nishiya Y, Ishizuka T, Hagihara K, Farid NA, Okazaki O, Ikeda T, Kurihara A: **Identification of the human cytochrome P450 enzymes involved in the two oxidative steps in the bioactivation of clopidogrel to its pharmacologically active metabolite.** *Drug metabolism and disposition: the biological fate of chemicals* 2010, **38:**92-99.

4. Taubert D, von Beckerath N, Grimberg G, Lazar A, Jung N, Goeser T, Kastrati A, Schomig A, Schomig E: **Impact of P-glycoprotein on clopidogrel absorption.** *Clinical pharmacology and therapeutics* 2006, **80:**486-501.

5. Simon T, Verstuyft C, Mary-Krause M, Quteineh L, Drouet E, Meneveau N, Steg PG, Ferrieres J, Danchin N, Becquemont L, et al: **Genetic determinants of response to clopidogrel and cardiovascular events.** *The New England journal of medicine* 2009, **360:**363-375.

6. Mega JL, Close SL, Wiviott SD, Shen L, Walker JR, Simon T, Antman EM, Braunwald E, Sabatine MS: **Genetic variants in ABCB1 and CYP2C19 and cardiovascular outcomes after treatment with clopidogrel and prasugrel in the TRITON-TIMI 38 trial: a pharmacogenetic analysis.** *Lancet* 2010, **376:**1312-1319.

7. Gladding P, Webster M, Zeng I, Farrell H, Stewart J, Ruygrok P, Ormiston J, El-Jack S, Armstrong G, Kay P, et al: **The pharmacogenetics and pharmacodynamics of clopidogrel response: an analysis from the PRINC (Plavix Response in Coronary Intervention) trial.** *JACC Cardiovascular interventions* 2008, **1:**620-627.

8. Shuldiner AR, O'Connell JR, Bliden KP, Gandhi A, Ryan K, Horenstein RB, Damcott CM, Pakyz R, Tantry US, Gibson Q, et al: **Association of cytochrome P450 2C19 genotype with the antiplatelet effect and clinical efficacy of clopidogrel therapy.** *JAMA : the journal of the American Medical Association* 2009, **302:**849-857.

9. Jeong YH, Kim IS, Park Y, Kang MK, Koh JS, Hwang SJ, Kwak CH, Hwang JY: **Carriage of cytochrome 2C19 polymorphism is associated with risk of high post-treatment platelet reactivity on high maintenance-dose clopidogrel of 150 mg/day: results of the ACCEL-DOUBLE (Accelerated Platelet Inhibition by a Double Dose of Clopidogrel According to Gene Polymorphism) study.** *JACC Cardiovascular interventions* 2010, **3:**731-741.

10. Spiewak M, Malek LA, Kostrzewa G, Kisiel B, Serafin A, Filipiak KJ, Ploski R, Opolski G: **Influence of C3435T multidrug resistance gene-1 (MDR-1) polymorphism on platelet reactivity and prognosis in patients with acute coronary syndromes.** *Kardiologia polska* 2009, **67:**827-834.

11. Tiroch KA, Sibbing D, Koch W, Roosen-Runge T, Mehilli J, Schomig A, Kastrati A: **Protective effect of the CYP2C19 *17 polymorphism with increased activation of clopidogrel on cardiovascular events.** *American heart journal* 2010, **160:**506-512.

12. Angiolillo DJ, Fernandez-Ortiz A, Bernardo E, Ramirez C, Cavallari U, Trabetti E, Sabate M, Jimenez-Quevedo P, Hernandez R, Moreno R, et al: **Lack of association between the P2Y12 receptor gene polymorphism and platelet response to clopidogrel in patients with coronary artery disease.** *Thrombosis research* 2005, **116:**491-497.

13. von Beckerath N, von Beckerath O, Koch W, Eichinger M, Schomig A, Kastrati A: **P2Y12 gene H2 haplotype is not associated with increased adenosine diphosphate-induced platelet aggregation after initiation of clopidogrel therapy with a high loading dose.** *Blood coagulation & fibrinolysis : an international journal in haemostasis and thrombosis* 2005, **16:**199-204.

14. Smith SM, Judge HM, Peters G, Armstrong M, Fontana P, Gaussem P, Daly ME, Storey RF: **Common sequence variations in the P2Y12 and CYP3A5 genes do not explain the variability in the inhibitory effects of clopidogrel therapy.** *Platelets* 2006, **17:**250-258.

15. Giusti B, Gori AM, Marcucci R, Saracini C, Sestini I, Paniccia R, Valente S, Antoniucci D, Abbate R, Gensini GF: **Cytochrome P450 2C19 loss-of-function polymorphism, but not CYP3A4 IVS10 + 12G/A and P2Y12 T744C polymorphisms, is associated with response variability to dual antiplatelet treatment in high-risk vascular patients.** *Pharmacogenetics and genomics* 2007, **17:**1057-1064.

16. Mega JL, Close SL, Wiviott SD, Shen L, Hockett RD, Brandt JT, Walker JR, Antman EM, Macias W, Braunwald E, Sabatine MS: **Cytochrome p-450 polymorphisms and response to clopidogrel.** *The New England journal of medicine* 2009, **360:**354-362.

17. Varenhorst C, James S, Erlinge D, Brandt JT, Braun OO, Man M, Siegbahn A, Walker J, Wallentin L, Winters KJ, Close SL: **Genetic variation of CYP2C19 affects both pharmacokinetic and pharmacodynamic responses to clopidogrel but not prasugrel in aspirin-treated patients with coronary artery disease.** *European heart journal* 2009, **30:**1744-1752.

18. Hulot JS, Bura A, Villard E, Azizi M, Remones V, Goyenvalle C, Aiach M, Lechat P, Gaussem P: **Cytochrome P450 2C19 loss-of-function polymorphism is a major determinant of clopidogrel responsiveness in healthy subjects.** *Blood* 2006, **108:**2244-2247.

19. Umemura K, Furuta T, Kondo K: **The common gene variants of CYP2C19 affect pharmacokinetics and pharmacodynamics in an active metabolite of clopidogrel in healthy subjects.** *Journal of thrombosis and haemostasis : JTH* 2008, **6:**1439-1441.

20. Frere C, Cuisset T, Morange PE, Quilici J, Camoin-Jau L, Saut N, Faille D, Lambert M, Juhan-Vague I, Bonnet JL, Alessi MC: **Effect of cytochrome p450 polymorphisms on platelet reactivity after treatment with clopidogrel in acute coronary syndrome.** *The American journal of cardiology* 2008, **101:**1088-1093.

21. Trenk D, Hochholzer W, Fromm MF, Chialda LE, Pahl A, Valina CM, Stratz C, Schmiebusch P, Bestehorn HP, Buttner HJ, Neumann FJ: **Cytochrome P450 2C19 681G>A polymorphism and high on-clopidogrel platelet reactivity associated with adverse 1-year clinical outcome of elective percutaneous coronary intervention with drug-eluting or bare-metal stents.** *Journal of the American College of Cardiology* 2008, **51:**1925-1934.

22. Sibbing D, Stegherr J, Latz W, Koch W, Mehilli J, Dorrler K, Morath T, Schomig A, Kastrati A, von Beckerath N: **Cytochrome P450 2C19 loss-of-function polymorphism and stent thrombosis following percutaneous coronary intervention.** *European heart journal* 2009, **30:**916-922.

23. Giusti B, Gori AM, Marcucci R, Saracini C, Sestini I, Paniccia R, Buonamici P, Antoniucci D, Abbate R, Gensini GF: **Relation of cytochrome P450 2C19 loss-of-function polymorphism to occurrence of drug-eluting coronary stent thrombosis.** *The American journal of cardiology* 2009, **103:**806-811.

24. Collet JP, Hulot JS, Pena A, Villard E, Esteve JB, Silvain J, Payot L, Brugier D, Cayla G, Beygui F, et al: **Cytochrome P450 2C19 polymorphism in young patients treated with clopidogrel after myocardial infarction: a cohort study.** *Lancet* 2009, **373:**309-317.

25. Mega JL, Simon T, Collet JP, Anderson JL, Antman EM, Bliden K, Cannon CP, Danchin N, Giusti B, Gurbel P, et al: **Reduced-function CYP2C19 genotype and risk of adverse clinical outcomes among patients treated with clopidogrel predominantly for PCI: a meta-analysis.** *JAMA : the journal of the American Medical Association* 2010, **304:**1821-1830.

26. Sofi F, Giusti B, Marcucci R, Gori AM, Abbate R, Gensini GF: **Cytochrome P450 2C19*2 polymorphism and cardiovascular recurrences in patients taking clopidogrel: a meta-analysis.** *The pharmacogenomics journal* 2011, **11:**199-206.

27. Hulot JS, Collet JP, Silvain J, Pena A, Bellemain-Appaix A, Barthelemy O, Cayla G, Beygui F, Montalescot G: **Cardiovascular risk in clopidogrel-treated patients according to cytochrome P450 2C19*2 loss-of-function allele or proton pump inhibitor coadministration: a systematic meta-analysis.** *Journal of the American College of Cardiology* 2010, **56:**134-143.

28. Sofi F, Marcucci R, Gori AM, Giusti B, Abbate R, Gensini GF: **Clopidogrel non-responsiveness and risk of cardiovascular morbidity. An updated meta-analysis.** *Thrombosis and haemostasis* 2010, **103:**841-848.

29. Ma TK, Lam YY, Tan VP, Kiernan TJ, Yan BP: **Impact of genetic and acquired alteration in cytochrome P450 system on pharmacologic and clinical response to clopidogrel.** *Pharmacology & therapeutics* 2010, **125:**249-259.

30. Mega JL, Hochholzer W, Frelinger AL, 3rd, Kluk MJ, Angiolillo DJ, Kereiakes DJ, Isserman S, Rogers WJ, Ruff CT, Contant C, et al: **Dosing clopidogrel based on CYP2C19 genotype and the effect on platelet reactivity in patients with stable cardiovascular disease.** *JAMA : the journal of the American Medical Association* 2011, **306:**2221-2228.

31. Cuisset T, Quilici J, Cohen W, Fourcade L, Saut N, Pankert M, Gaborit B, Carrieri P, Morange PE, Bonnet JL, Alessi MC: **Usefulness of high clopidogrel maintenance dose according to CYP2C19 genotypes in clopidogrel low responders undergoing coronary stenting for non ST elevation acute coronary syndrome.** *The American journal of cardiology* 2011, **108:**760-765.

32. Wallentin L, James S, Storey RF, Armstrong M, Barratt BJ, Horrow J, Husted S, Katus H, Steg PG, Shah SH, et al: **Effect of CYP2C19 and ABCB1 single nucleotide polymorphisms on outcomes of treatment with ticagrelor versus clopidogrel for acute coronary syndromes: a genetic substudy of the PLATO trial.** *Lancet* 2010, **376:**1320-1328.

33. Pare G, Mehta SR, Yusuf S, Anand SS, Connolly SJ, Hirsh J, Simonsen K, Bhatt DL, Fox KA, Eikelboom JW: **Effects of CYP2C19 genotype on outcomes of clopidogrel treatment.** *The New England journal of medicine* 2010, **363:**1704-1714.

34. Holmes DR, Jr., Dehmer GJ, Kaul S, Leifer D, O'Gara PT, Stein CM: **ACCF/AHA clopidogrel clinical alert: approaches to the FDA "boxed warning": a report of the American College of Cardiology Foundation Task Force on clinical expert consensus documents and the American Heart Association endorsed by the Society for Cardiovascular Angiography and Interventions and the Society of Thoracic Surgeons.** *Journal of the American College of Cardiology* 2010, **56:**321-341.

35. Johnson JA, Roden DM, Lesko LJ, Ashley E, Klein TE, Shuldiner AR: **Clopidogrel: a case for indication-specific pharmacogenetics.** *Clinical pharmacology and therapeutics* 2012, **91:**774-776.

36. Sibbing D, Gebhard D, Koch W, Braun S, Stegherr J, Morath T, Von Beckerath N, Mehilli J, Schomig A, Schuster T, Kastrati A: **Isolated and interactive impact of common CYP2C19 genetic variants on the antiplatelet effect of chronic clopidogrel therapy.** *Journal of thrombosis and haemostasis : JTH* 2010, **8:**1685-1693.

37. **Plavix Drug label December 2011** [[[http://www.accessdata.fda.gov/drugsatfda_docs/label/2011/020839s055lbl.pdf]](http://www.accessdata.fda.gov/drugsatfda_docs/label/2011/020839s055lbl.pdf%5d)]

38. Fock KM, Ang TL, Bee LC, Lee EJ: **Proton pump inhibitors: do differences in pharmacokinetics translate into differences in clinical outcomes?** *Clinical pharmacokinetics* 2008, **47:**1-6.

39. Shin JM, Sachs G: **Pharmacology of proton pump inhibitors.** *Current gastroenterology reports* 2008, **10:**528-534.

40. Bonapace ES, Fisher RS, Parkman HP: **Does fasting serum gastrin predict gastric acid suppression in patients on proton-pump inhibitors?** *Digestive diseases and sciences* 2000, **45:**34-39.

41. Der G: **An overview of proton pump inhibitors.** *Gastroenterology nursing : the official journal of the Society of Gastroenterology Nurses and Associates* 2003, **26:**182-190.

42. Ishizaki T, Horai Y: **Review article: cytochrome P450 and the metabolism of proton pump inhibitors--emphasis on rabeprazole.** *Alimentary pharmacology & therapeutics* 1999, **13 Suppl 3:**27-36.

43. Sim SC, Risinger C, Dahl ML, Aklillu E, Christensen M, Bertilsson L, Ingelman-Sundberg M: **A common novel CYP2C19 gene variant causes ultrarapid drug metabolism relevant for the drug response to proton pump inhibitors and antidepressants.** *Clinical pharmacology and therapeutics* 2006, **79:**103-113.

44. Rocha A, Coelho EB, Moussa SA, Lanchote VL: **Investigation of the in vivo activity of CYP3A in Brazilian volunteers: comparison of midazolam and omeprazole as drug markers.** *European journal of clinical pharmacology* 2008, **64:**901-906.

45. Gawronska-Szklarz B, Siuda A, Kurzawski M, Bielicki D, Marlicz W, Drozdzik M: **Effects of CYP2C19, MDR1, and interleukin 1-B gene variants on the eradication rate of Helicobacter pylori infection by triple therapy with pantoprazole, amoxicillin, and metronidazole.** *European journal of clinical pharmacology* 2010, **66:**681-687.

46. Hu YM, Mei Q, Xu XH, Hu XP, Hu NZ, Xu JM: **Pharmacodynamic and kinetic effect of rabeprazole on serum gastrin level in relation to CYP2C19 polymorphism in Chinese Hans.** *World journal of gastroenterology : WJG* 2006, **12:**4750-4753.

47. Baldwin RM, Ohlsson S, Pedersen RS, Mwinyi J, Ingelman-Sundberg M, Eliasson E, Bertilsson L: **Increased omeprazole metabolism in carriers of the CYP2C19*17 allele; a pharmacokinetic study in healthy volunteers.** *British journal of clinical pharmacology* 2008, **65:**767-774.

48. Furuta T, Ohashi K, Kosuge K, Zhao XJ, Takashima M, Kimura M, Nishimoto M, Hanai H, Kaneko E, Ishizaki T: **CYP2C19 genotype status and effect of omeprazole on intragastric pH in humans.** *Clinical pharmacology and therapeutics* 1999, **65:**552-561.

49. Horai Y, Kimura M, Furuie H, Matsuguma K, Irie S, Koga Y, Nagahama T, Murakami M, Matsui T, Yao T, et al: **Pharmacodynamic effects and kinetic disposition of rabeprazole in relation to CYP2C19 genotypes.** *Alimentary pharmacology & therapeutics* 2001, **15:**793-803.

50. Shirai N, Furuta T, Moriyama Y, Okochi H, Kobayashi K, Takashima M, Xiao F, Kosuge K, Nakagawa K, Hanai H, et al: **Effects of CYP2C19 genotypic differences in the metabolism of omeprazole and rabeprazole on intragastric pH.** *Alimentary pharmacology & therapeutics* 2001, **15:**1929-1937.

51. Hunfeld NG, Touw DJ, Mathot RA, Mulder PG, RH VANS, Kuipers EJ, Kooiman JC, Geus WP: **A comparison of the acid-inhibitory effects of esomeprazole and pantoprazole in relation to pharmacokinetics and CYP2C19 polymorphism.** *Alimentary pharmacology & therapeutics* 2010, **31:**150-159.

52. Wang H, An N, Wang H, Gao Y, Liu D, Bian T, Zhu J, Chen C: **Evaluation of the effects of 20 nonsynonymous single nucleotide polymorphisms of CYP2C19 on S-mephenytoin 4'-hydroxylation and omeprazole 5'-hydroxylation.** *Drug metabolism and disposition: the biological fate of chemicals* 2011, **39:**830-837.

53. Sibbing D, Koch W, Gebhard D, Schuster T, Braun S, Stegherr J, Morath T, Schomig A, von Beckerath N, Kastrati A: **Cytochrome 2C19*17 allelic variant, platelet aggregation, bleeding events, and stent thrombosis in clopidogrel-treated patients with coronary stent placement.** *Circulation* 2010, **121:**512-518.

54. Kurzawski M, Gawronska-Szklarz B, Wrzesniewska J, Siuda A, Starzynska T, Drozdzik M: **Effect of CYP2C19*17 gene variant on Helicobacter pylori eradication in peptic ulcer patients.** *European journal of clinical pharmacology* 2006, **62:**877-880.

55. Furuta T, Shirai N, Watanabe F, Honda S, Takeuchi K, Iida T, Sato Y, Kajimura M, Futami H, Takayanagi S, et al: **Effect of cytochrome P4502C19 genotypic differences on cure rates for gastroesophageal reflux disease by lansoprazole.** *Clinical pharmacology and therapeutics* 2002, **72:**453-460.

56. Take S, Mizuno M, Ishiki K, Nagahara Y, Yoshida T, Inaba T, Yamamoto K, Okada H, Yokota K, Oguma K, Shiratori Y: **Interleukin-1beta genetic polymorphism influences the effect of cytochrome P 2C19 genotype on the cure rate of 1-week triple therapy for Helicobacter pylori infection.** *The American journal of gastroenterology* 2003, **98:**2403-2408.

57. Sagar M, Tybring G, Dahl ML, Bertilsson L, Seensalu R: **Effects of omeprazole on intragastric pH and plasma gastrin are dependent on the CYP2C19 polymorphism.** *Gastroenterology* 2000, **119:**670-676.

58. Kawamura M, Ohara S, Koike T, Iijima K, Suzuki J, Kayaba S, Noguchi K, Hamada S, Noguchi M, Shimosegawa T, Study Group of G: **The effects of lansoprazole on erosive reflux oesophagitis are influenced by CYP2C19 polymorphism.** *Alimentary pharmacology & therapeutics* 2003, **17:**965-973.

59. Zendehdel N, Biramijamal F, Hossein-Nezhad A, Zendehdel N, Sarie H, Doughaiemoghaddam M, Pourshams A: **Role of cytochrome P450 2C19 genetic polymorphisms in the therapeutic efficacy of omeprazole in Iranian patients with erosive reflux esophagitis.** *Archives of Iranian medicine* 2010, **13:**406-412.

60. Tseng PH, Lee YC, Chiu HM, Wang HP, Lin JT, Wu MS: **A comparative study of proton-pump inhibitor tests for Chinese reflux patients in relation to the CYP2C19 genotypes.** *Journal of clinical gastroenterology* 2009, **43:**920-925.

61. Ohkusa T, Maekawa T, Arakawa T, Nakajima M, Fujimoto K, Hoshino E, Mitachi Y, Hamada S, Mine T, Kawahara Y, et al: **Effect of CYP2C19 polymorphism on the safety and efficacy of omeprazole in Japanese patients with recurrent reflux oesophagitis.** *Alimentary pharmacology & therapeutics* 2005, **21:**1331-1339.

62. Oh JH, Dong MS, Choi MG, Yoo HW, Lee SB, Park YI, Chung IS: **Effects of CYP2C19 and MDR1 genotype on the eradication rate of Helicobacter pylori infection by triple therapy with pantoprazole, amoxycillin and clarithromycin.** *Journal of gastroenterology and hepatology* 2009, **24:**294-298.

63. Gawronska-Szklarz B, Wrzesniewska J, Starzynska T, Pawlik A, Safranow K, Ferenc K, Drozdzik M: **Effect of CYP2C19 and MDR1 polymorphisms on cure rate in patients with acid-related disorders with Helicobacter pylori infection.** *European journal of clinical pharmacology* 2005, **61:**375-379.

64. Miura M, Satoh S, Tada H, Saito M, Kagaya H, Inoue K, Sagae Y, Kanno S, Ishikawa M, Habuchi T, Suzuki T: **Influence of ABCB1 C3435T polymorphism on the pharmacokinetics of lansoprazole and gastroesophageal symptoms in Japanese renal transplant recipients classified as CYP2C19 extensive metabolizers and treated with tacrolimus.** *International journal of clinical pharmacology and therapeutics* 2006, **44:**605-613.

65. Furuta T, Shirai N, Xiao F, El-Omar EM, Rabkin CS, Sugimura H, Ishizaki T, Ohashi K: **Polymorphism of interleukin-1beta affects the eradication rates of Helicobacter pylori by triple therapy.** *Clinical gastroenterology and hepatology : the official clinical practice journal of the American Gastroenterological Association* 2004, **2:**22-30.

66. Sugimoto M, Furuta T, Yamaoka Y: **Influence of inflammatory cytokine polymorphisms on eradication rates of Helicobacter pylori.** *Journal of gastroenterology and hepatology* 2009, **24:**1725-1732.

67. Zhang L, Mei Q, Li QS, Hu YM, Xu JM: **The effect of cytochrome P2C19 and interleukin-1 polymorphisms on H. pylori eradication rate of 1-week triple therapy with omeprazole or rabeprazole, amoxycillin and clarithromycin in Chinese people.** *Journal of clinical pharmacy and therapeutics* 2010, **35:**713-722.

68. Swen JJ, Nijenhuis M, de Boer A, Grandia L, Maitland-van der Zee AH, Mulder H, Rongen GA, van Schaik RH, Schalekamp T, Touw DJ, et al: **Pharmacogenetics: from bench to byte--an update of guidelines.** *Clinical pharmacology and therapeutics* 2011, **89:**662-673.

69. Chaudhry AS, Kochhar R, Kohli KK: **Genetic polymorphism of CYP2C19 & therapeutic response to proton pump inhibitors.** *The Indian journal of medical research* 2008, **127:**521-530.

70. Dojo M, Azuma T, Saito T, Ohtani M, Muramatsu A, Kuriyama M: **Effects of CYP2C19 gene polymorphism on cure rates for Helicobacter pylori infection by triple therapy with proton pump inhibitor (omeprazole or rabeprazole), amoxycillin and clarithromycin in Japan.** *Digestive and liver disease : official journal of the Italian Society of Gastroenterology and the Italian Association for the Study of the Liver* 2001, **33:**671-675.

71. Inaba T, Mizuno M, Kawai K, Yokota K, Oguma K, Miyoshi M, Take S, Okada H, Tsuji T: **Randomized open trial for comparison of proton pump inhibitors in triple therapy for Helicobacter pylori infection in relation to CYP2C19 genotype.** *Journal of gastroenterology and hepatology* 2002, **17:**748-753.

72. Mazer-Amirshahi M, van den Anker J: **Impact of the CYP2C19*17 polymorphism on the pharmacokinetics and pharmacodynamics of proton pump inhibitors.** *Journal of clinical pharmacology* 2013, **53:**359.

73. Tang C, Shou M, Rushmore TH, Mei Q, Sandhu P, Woolf EJ, Rose MJ, Gelmann A, Greenberg HE, De Lepeleire I, et al: **In-vitro metabolism of celecoxib, a cyclooxygenase-2 inhibitor, by allelic variant forms of human liver microsomal cytochrome P450 2C9: correlation with CYP2C9 genotype and in-vivo pharmacokinetics.** *Pharmacogenetics* 2001, **11:**223-235.

74. Kirchheiner J, Stormer E, Meisel C, Steinbach N, Roots I, Brockmoller J: **Influence of CYP2C9 genetic polymorphisms on pharmacokinetics of celecoxib and its metabolites.** *Pharmacogenetics* 2003, **13:**473-480.

75. Lundblad MS, Ohlsson S, Johansson P, Lafolie P, Eliasson E: **Accumulation of celecoxib with a 7-fold higher drug exposure in individuals homozygous for CYP2C9*3.** *Clinical pharmacology and therapeutics* 2006, **79:**287-288.

76. Stempak D, Bukaveckas BL, Linder M, Koren G, Baruchel S: **Cytochrome P450 2C9 genotype: impact on celecoxib safety and pharmacokinetics in a pediatric patient.** *Clinical pharmacology and therapeutics* 2005, **78:**309-310.

77. Chan AT, Zauber AG, Hsu M, Breazna A, Hunter DJ, Rosenstein RB, Eagle CJ, Hawk ET, Bertagnolli MM: **Cytochrome P450 2C9 variants influence response to celecoxib for prevention of colorectal adenoma.** *Gastroenterology* 2009, **136:**2127-2136 e2121.

78. Pilotto A, Seripa D, Franceschi M, Scarcelli C, Colaizzo D, Grandone E, Niro V, Andriulli A, Leandro G, Di Mario F, Dallapiccola B: **Genetic susceptibility to nonsteroidal anti-inflammatory drug-related gastroduodenal bleeding: role of cytochrome P450 2C9 polymorphisms.** *Gastroenterology* 2007, **133:**465-471.

79. **Celecoxib Drug Label February 2011** [[[http://www.accessdata.fda.gov/drugsatfda_docs/label/2011/020998s033,021156s003lbl.pdf]](http://www.accessdata.fda.gov/drugsatfda_docs/label/2011/020998s033,021156s003lbl.pdf%5d)]

80. Hirsh J, Dalen JE, Anderson DR, Poller L, Bussey H, Ansell J, Deykin D, Brandt JT: **Oral anticoagulants: mechanism of action, clinical effectiveness, and optimal therapeutic range.** *Chest* 1998, **114:**445S-469S.

81. Group TEAFTS: **Optimal oral anticoagulant therapy in patients with nonrheumatic atrial fibrillation and recent cerebral ischemia. .** *The New England journal of medicine* 1995, **333:**5-10.

82. Fuster V, Ryden LE, Asinger RW, Cannom DS, Crijns HJ, Frye RL, Halperin JL, Kay GN, Klein WW, Levy S, et al: **ACC/AHA/ESC guidelines for the management of patients with atrial fibrillation: executive summary. A Report of the American College of Cardiology/ American Heart Association Task Force on Practice Guidelines and the European Society of Cardiology Committee for Practice Guidelines and Policy Conferences (Committee to Develop Guidelines for the Management of Patients With Atrial Fibrillation): developed in Collaboration With the North American Society of Pacing and Electrophysiology.** *Journal of the American College of Cardiology* 2001, **38:**1231-1266.

83. Garcia D, Regan S, Crowther M, Hughes RA, Hylek EM: **Warfarin maintenance dosing patterns in clinical practice: implications for safer anticoagulation in the elderly population.** *Chest* 2005, **127:**2049-2056.

84. Johnson JA, Gong L, Whirl-Carrillo M, Gage BF, Scott SA, Stein CM, Anderson JL, Kimmel SE, Lee MT, Pirmohamed M, et al: **Clinical Pharmacogenetics Implementation Consortium Guidelines for CYP2C9 and VKORC1 genotypes and warfarin dosing.** *Clinical pharmacology and therapeutics* 2011, **90:**625-629.

85. McWilliam A LR, Nardinelli C. : **Health care savings from personalizing medicine using genetic testing: The care for warfarin.** In *Book Health care savings from personalizing medicine using genetic testing: The care for warfarin.* (Editor ed.^eds.), vol. Working Paper 06-23. . pp. 1-17. City; 2006:1-17.

86. Shehab N, Sperling LS, Kegler SR, Budnitz DS: **National estimates of emergency department visits for hemorrhage-related adverse events from clopidogrel plus aspirin and from warfarin.** *Archives of internal medicine* 2010, **170:**1926-1933.

87. Wadelius M, Chen LY, Lindh JD, Eriksson N, Ghori MJ, Bumpstead S, Holm L, McGinnis R, Rane A, Deloukas P: **The largest prospective warfarin-treated cohort supports genetic forecasting.** *Blood* 2009, **113:**784-792.

88. McDonald MG, Rieder MJ, Nakano M, Hsia CK, Rettie AE: **CYP4F2 is a vitamin K1 oxidase: An explanation for altered warfarin dose in carriers of the V433M variant.** *Molecular pharmacology* 2009, **75:**1337-1346.

89. Rieder MJ, Reiner AP, Rettie AE: **Gamma-glutamyl carboxylase (GGCX) tagSNPs have limited utility for predicting warfarin maintenance dose.** *Journal of thrombosis and haemostasis : JTH* 2007, **5:**2227-2234.

90. Wadelius M, Chen LY, Eriksson N, Bumpstead S, Ghori J, Wadelius C, Bentley D, McGinnis R, Deloukas P: **Association of warfarin dose with genes involved in its action and metabolism.** *Human genetics* 2007, **121:**23-34.

91. Pautas E, Moreau C, Gouin-Thibault I, Golmard JL, Mahe I, Legendre C, Taillandier-Heriche E, Durand-Gasselin B, Houllier AM, Verrier P, et al: **Genetic factors (VKORC1, CYP2C9, EPHX1, and CYP4F2) are predictor variables for warfarin response in very elderly, frail inpatients.** *Clinical pharmacology and therapeutics* 2010, **87:**57-64.

92. Kimmel SE, Christie J, Kealey C, Chen Z, Price M, Thorn CF, Brensinger CM, Newcomb CW, Whitehead AS: **Apolipoprotein E genotype and warfarin dosing among Caucasians and African Americans.** *The pharmacogenomics journal* 2008, **8:**53-60.

93. Sconce EA, Daly AK, Khan TI, Wynne HA, Kamali F: **APOE genotype makes a small contribution to warfarin dose requirements.** *Pharmacogenetics and genomics* 2006, **16:**609-611.

94. Vecsler M, Loebstein R, Almog S, Kurnik D, Goldman B, Halkin H, Gak E: **Combined genetic profiles of components and regulators of the vitamin K-dependent gamma-carboxylation system affect individual sensitivity to warfarin.** *Thrombosis and haemostasis* 2006, **95:**205-211.

95. Lubitz SA, Scott SA, Rothlauf EB, Agarwal A, Peter I, Doheny D, Van Der Zee S, Jaremko M, Yoo C, Desnick RJ, Halperin JL: **Comparative performance of gene-based warfarin dosing algorithms in a multiethnic population.** *Journal of thrombosis and haemostasis : JTH* 2010, **8:**1018-1026.

96. Cavallari LH, Langaee TY, Momary KM, Shapiro NL, Nutescu EA, Coty WA, Viana MA, Patel SR, Johnson JA: **Genetic and clinical predictors of warfarin dose requirements in African Americans.** *Clinical pharmacology and therapeutics* 2010, **87:**459-464.

97. Lal S, Sandanaraj E, Jada SR, Kong MC, Lee LH, Goh BC, Lee SC, Chowbay B: **Influence of APOE genotypes and VKORC1 haplotypes on warfarin dose requirements in Asian patients.** *British journal of clinical pharmacology* 2008, **65:**260-264.

98. Ali ZK, Kim RJ, Ysla FM: **CYP2C9 polymorphisms: considerations in NSAID therapy.** *Current opinion in drug discovery & development* 2009, **12:**108-114.

99. Lee CR, Goldstein JA, Pieper JA: **Cytochrome P450 2C9 polymorphisms: a comprehensive review of the in-vitro and human data.** *Pharmacogenetics* 2002, **12:**251-263.

100. Lindh JD, Lundgren S, Holm L, Alfredsson L, Rane A: **Several-fold increase in risk of overanticoagulation by CYP2C9 mutations.** *Clinical pharmacology and therapeutics* 2005, **78:**540-550.

101. Sanderson S, Emery J, Higgins J: **CYP2C9 gene variants, drug dose, and bleeding risk in warfarin-treated patients: a HuGEnet systematic review and meta-analysis.** *Genetics in medicine : official journal of the American College of Medical Genetics* 2005, **7:**97-104.

102. Aithal GP, Day CP, Kesteven PJ, Daly AK: **Association of polymorphisms in the cytochrome P450 CYP2C9 with warfarin dose requirement and risk of bleeding complications.** *Lancet* 1999, **353:**717-719.

103. Higashi MK, Veenstra DL, Kondo LM, Wittkowsky AK, Srinouanprachanh SL, Farin FM, Rettie AE: **Association between CYP2C9 genetic variants and anticoagulation-related outcomes during warfarin therapy.** *JAMA : the journal of the American Medical Association* 2002, **287:**1690-1698.

104. Kusama M, Maeda K, Chiba K, Aoyama A, Sugiyama Y: **Prediction of the effects of genetic polymorphism on the pharmacokinetics of CYP2C9 substrates from in vitro data.** *Pharmaceutical research* 2009, **26:**822-835.

105. Sistonen J, Fuselli S, Palo JU, Chauhan N, Padh H, Sajantila A: **Pharmacogenetic variation at CYP2C9, CYP2C19, and CYP2D6 at global and microgeographic scales.** *Pharmacogenetics and genomics* 2009, **19:**170-179.

106. Kim HS, Lee SS, Oh M, Jang YJ, Kim EY, Han IY, Cho KH, Shin JG: **Effect of CYP2C9 and VKORC1 genotypes on early-phase and steady-state warfarin dosing in Korean patients with mechanical heart valve replacement.** *Pharmacogenetics and genomics* 2009, **19:**103-112.

107. Zhao F, Loke C, Rankin SC, Guo JY, Lee HS, Wu TS, Tan T, Liu TC, Lu WL, Lim YT, et al: **Novel CYP2C9 genetic variants in Asian subjects and their influence on maintenance warfarin dose.** *Clinical pharmacology and therapeutics* 2004, **76:**210-219.

108. Rieder MJ, Reiner AP, Gage BF, Nickerson DA, Eby CS, McLeod HL, Blough DK, Thummel KE, Veenstra DL, Rettie AE: **Effect of VKORC1 haplotypes on transcriptional regulation and warfarin dose.** *The New England journal of medicine* 2005, **352:**2285-2293.

109. D'Andrea G, D'Ambrosio RL, Di Perna P, Chetta M, Santacroce R, Brancaccio V, Grandone E, Margaglione M: **A polymorphism in the VKORC1 gene is associated with an interindividual variability in the dose-anticoagulant effect of warfarin.** *Blood* 2005, **105:**645-649.

110. Geisen C, Watzka M, Sittinger K, Steffens M, Daugela L, Seifried E, Muller CR, Wienker TF, Oldenburg J: **VKORC1 haplotypes and their impact on the inter-individual and inter-ethnical variability of oral anticoagulation.** *Thrombosis and haemostasis* 2005, **94:**773-779.

111. Herman D, Peternel P, Stegnar M, Breskvar K, Dolzan V: **The influence of sequence variations in factor VII, gamma-glutamyl carboxylase and vitamin K epoxide reductase complex genes on warfarin dose requirement.** *Thrombosis and haemostasis* 2006, **95:**782-787.

112. Schwarz UI, Ritchie MD, Bradford Y, Li C, Dudek SM, Frye-Anderson A, Kim RB, Roden DM, Stein CM: **Genetic determinants of response to warfarin during initial anticoagulation.** *The New England journal of medicine* 2008, **358:**999-1008.

113. Wadelius M, Chen LY, Downes K, Ghori J, Hunt S, Eriksson N, Wallerman O, Melhus H, Wadelius C, Bentley D, Deloukas P: **Common VKORC1 and GGCX polymorphisms associated with warfarin dose.** *The pharmacogenomics journal* 2005, **5:**262-270.

114. Cooper GM, Johnson JA, Langaee TY, Feng H, Stanaway IB, Schwarz UI, Ritchie MD, Stein CM, Roden DM, Smith JD, et al: **A genome-wide scan for common genetic variants with a large influence on warfarin maintenance dose.** *Blood* 2008, **112:**1022-1027.

115. Veenstra DL, You JH, Rieder MJ, Farin FM, Wilkerson HW, Blough DK, Cheng G, Rettie AE: **Association of Vitamin K epoxide reductase complex 1 (VKORC1) variants with warfarin dose in a Hong Kong Chinese patient population.** *Pharmacogenetics and genomics* 2005, **15:**687-691.

116. Takahashi H, Wilkinson GR, Nutescu EA, Morita T, Ritchie MD, Scordo MG, Pengo V, Barban M, Padrini R, Ieiri I, et al: **Different contributions of polymorphisms in VKORC1 and CYP2C9 to intra- and inter-population differences in maintenance dose of warfarin in Japanese, Caucasians and African-Americans.** *Pharmacogenetics and genomics* 2006, **16:**101-110.

117. Limdi NA, Beasley TM, Crowley MR, Goldstein JA, Rieder MJ, Flockhart DA, Arnett DK, Acton RT, Liu N: **VKORC1 polymorphisms, haplotypes and haplotype groups on warfarin dose among African-Americans and European-Americans.** *Pharmacogenomics* 2008, **9:**1445-1458.

118. Scott SA, Edelmann L, Kornreich R, Desnick RJ: **Warfarin pharmacogenetics: CYP2C9 and VKORC1 genotypes predict different sensitivity and resistance frequencies in the Ashkenazi and Sephardi Jewish populations.** *American journal of human genetics* 2008, **82:**495-500.

119. Rost S, Fregin A, Ivaskevicius V, Conzelmann E, Hortnagel K, Pelz HJ, Lappegard K, Seifried E, Scharrer I, Tuddenham EG, et al: **Mutations in VKORC1 cause warfarin resistance and multiple coagulation factor deficiency type 2.** *Nature* 2004, **427:**537-541.

120. Stec DE, Roman RJ, Flasch A, Rieder MJ: **Functional polymorphism in human CYP4F2 decreases 20-HETE production.** *Physiological genomics* 2007, **30:**74-81.

121. Caldwell MD, Awad T, Johnson JA, Gage BF, Falkowski M, Gardina P, Hubbard J, Turpaz Y, Langaee TY, Eby C, et al: **CYP4F2 genetic variant alters required warfarin dose.** *Blood* 2008, **111:**4106-4112.

122. Takeuchi F, McGinnis R, Bourgeois S, Barnes C, Eriksson N, Soranzo N, Whittaker P, Ranganath V, Kumanduri V, McLaren W, et al: **A genome-wide association study confirms VKORC1, CYP2C9, and CYP4F2 as principal genetic determinants of warfarin dose.** *PLoS genetics* 2009, **5:**e1000433.

123. Borgiani P, Ciccacci C, Forte V, Sirianni E, Novelli L, Bramanti P, Novelli G: **CYP4F2 genetic variant (rs2108622) significantly contributes to warfarin dosing variability in the Italian population.** *Pharmacogenomics* 2009, **10:**261-266.

124. Perez-Andreu V, Roldan V, Anton AI, Garcia-Barbera N, Corral J, Vicente V, Gonzalez-Conejero R: **Pharmacogenetic relevance of CYP4F2 V433M polymorphism on acenocoumarol therapy.** *Blood* 2009, **113:**4977-4979.

125. Teichert M, Eijgelsheim M, Rivadeneira F, Uitterlinden AG, van Schaik RH, Hofman A, De Smet PA, van Gelder T, Visser LE, Stricker BH: **A genome-wide association study of acenocoumarol maintenance dosage.** *Human molecular genetics* 2009, **18:**3758-3768.

126. Liang R, Wang C, Zhao H, Huang J, Hu D, Sun Y: **Influence of CYP4F2 genotype on warfarin dose requirement-a systematic review and meta-analysis.** *Thrombosis research* 2012, **130:**38-44.

127. **Coumadin Drug Label October 2011** [[[http://www.accessdata.fda.gov/drugsatfda_docs/label/2011/009218s107lbl.pdf]](http://www.accessdata.fda.gov/drugsatfda_docs/label/2011/009218s107lbl.pdf%5d)]

128. Ohno S, Kawana K, Nakajin S: **Contribution of UDP-glucuronosyltransferase 1A1 and 1A8 to morphine-6-glucuronidation and its kinetic properties.** *Drug metabolism and disposition: the biological fate of chemicals* 2008, **36:**688-694.

129. Chen ZR, Somogyi AA, Reynolds G, Bochner F: **Disposition and metabolism of codeine after single and chronic doses in one poor and seven extensive metabolisers.** *British journal of clinical pharmacology* 1991, **31:**381-390.

130. Yue QY, Hasselstrom J, Svensson JO, Sawe J: **Pharmacokinetics of codeine and its metabolites in Caucasian healthy volunteers: comparisons between extensive and poor hydroxylators of debrisoquine.** *British journal of clinical pharmacology* 1991, **31:**635-642.

131. Madadi P, Koren G: **Pharmacogenetic insights into codeine analgesia: implications to pediatric codeine use.** *Pharmacogenomics* 2008, **9:**1267-1284.

132. Evans WE, Relling MV: **Pharmacogenomics: translating functional genomics into rational therapeutics.** *Science* 1999, **286:**487-491.

133. Eichelbaum M, Ingelman-Sundberg M, Evans WE: **Pharmacogenomics and individualized drug therapy.** *Annual review of medicine* 2006, **57:**119-137.

134. Gardiner SJ, Begg EJ: **Pharmacogenetics, drug-metabolizing enzymes, and clinical practice.** *Pharmacological reviews* 2006, **58:**521-590.

135. Zanger UM, Raimundo S, Eichelbaum M: **Cytochrome P450 2D6: overview and update on pharmacology, genetics, biochemistry.** *Naunyn-Schmiedeberg's archives of pharmacology* 2004, **369:**23-37.

136. Sistonen J, Sajantila A, Lao O, Corander J, Barbujani G, Fuselli S: **CYP2D6 worldwide genetic variation shows high frequency of altered activity variants and no continental structure.** *Pharmacogenetics and genomics* 2007, **17:**93-101.

137. Steimer W, Zopf K, von Amelunxen S, Pfeiffer H, Bachofer J, Popp J, Messner B, Kissling W, Leucht S: **Allele-specific change of concentration and functional gene dose for the prediction of steady-state serum concentrations of amitriptyline and nortriptyline in CYP2C19 and CYP2D6 extensive and intermediate metabolizers.** *Clinical chemistry* 2004, **50:**1623-1633.

138. Kirchheiner J, Schmidt H, Tzvetkov M, Keulen JT, Lotsch J, Roots I, Brockmoller J: **Pharmacokinetics of codeine and its metabolite morphine in ultra-rapid metabolizers due to CYP2D6 duplication.** *The pharmacogenomics journal* 2007, **7:**257-265.

139. Schenk PW, van Fessem MA, Verploegh-Van Rij S, Mathot RA, van Gelder T, Vulto AG, van Vliet M, Lindemans J, Bruijn JA, van Schaik RH: **Association of graded allele-specific changes in CYP2D6 function with imipramine dose requirement in a large group of depressed patients.** *Molecular psychiatry* 2008, **13:**597-605.

140. Gaedigk A, Simon SD, Pearce RE, Bradford LD, Kennedy MJ, Leeder JS: **The CYP2D6 activity score: translating genotype information into a qualitative measure of phenotype.** *Clinical pharmacology and therapeutics* 2008, **83:**234-242.

141. Lotsch J, Rohrbacher M, Schmidt H, Doehring A, Brockmoller J, Geisslinger G: **Can extremely low or high morphine formation from codeine be predicted prior to therapy initiation?** *Pain* 2009, **144:**119-124.

142. Sachse C, Brockmoller J, Bauer S, Roots I: **Cytochrome P450 2D6 variants in a Caucasian population: allele frequencies and phenotypic consequences.** *American journal of human genetics* 1997, **60:**284-295.

143. Eckhardt K, Li S, Ammon S, Schanzle G, Mikus G, Eichelbaum M: **Same incidence of adverse drug events after codeine administration irrespective of the genetically determined differences in morphine formation.** *Pain* 1998, **76:**27-33.

144. Quiding H, Lundqvist G, Boreus LO, Bondesson U, Ohrvik J: **Analgesic effect and plasma concentrations of codeine and morphine after two dose levels of codeine following oral surgery.** *European journal of clinical pharmacology* 1993, **44:**319-323.

145. Stamer UM, Musshoff F, Kobilay M, Madea B, Hoeft A, Stuber F: **Concentrations of tramadol and O-desmethyltramadol enantiomers in different CYP2D6 genotypes.** *Clinical pharmacology and therapeutics* 2007, **82:**41-47.

146. Shord SS, Cavallari LH, Gao W, Jeong HY, Deyo K, Patel SR, Camp JR, Labott SM, Molokie RE: **The pharmacokinetics of codeine and its metabolites in Blacks with sickle cell disease.** *European journal of clinical pharmacology* 2009, **65:**651-658.

147. Johansson I, Lundqvist E, Bertilsson L, Dahl ML, Sjoqvist F, Ingelman-Sundberg M: **Inherited amplification of an active gene in the cytochrome P450 CYP2D locus as a cause of ultrarapid metabolism of debrisoquine.** *Proceedings of the National Academy of Sciences of the United States of America* 1993, **90:**11825-11829.

148. Dahl ML, Johansson I, Bertilsson L, Ingelman-Sundberg M, Sjoqvist F: **Ultrarapid hydroxylation of debrisoquine in a Swedish population. Analysis of the molecular genetic basis.** *The Journal of pharmacology and experimental therapeutics* 1995, **274:**516-520.

149. Lovlie R, Daly AK, Molven A, Idle JR, Steen VM: **Ultrarapid metabolizers of debrisoquine: characterization and PCR-based detection of alleles with duplication of the CYP2D6 gene.** *FEBS letters* 1996, **392:**30-34.

150. Madadi P, Ross CJ, Hayden MR, Carleton BC, Gaedigk A, Leeder JS, Koren G: **Pharmacogenetics of neonatal opioid toxicity following maternal use of codeine during breastfeeding: a case-control study.** *Clinical pharmacology and therapeutics* 2009, **85:**31-35.

151. Koren G, Cairns J, Chitayat D, Gaedigk A, Leeder SJ: **Pharmacogenetics of morphine poisoning in a breastfed neonate of a codeine-prescribed mother.** *Lancet* 2006, **368:**704.

152. Dalen P, Frengell C, Dahl ML, Sjoqvist F: **Quick onset of severe abdominal pain after codeine in an ultrarapid metabolizer of debrisoquine.** *Therapeutic drug monitoring* 1997, **19:**543-544.

153. Gasche Y, Daali Y, Fathi M, Chiappe A, Cottini S, Dayer P, Desmeules J: **Codeine intoxication associated with ultrarapid CYP2D6 metabolism.** *The New England journal of medicine* 2004, **351:**2827-2831.

154. Zaza G, Cheok M, Krynetskaia N, Thorn C, Stocco G, Hebert JM, McLeod H, Weinshilboum RM, Relling MV, Evans WE, et al: **Thiopurine pathway.** *Pharmacogenetics and genomics* 2010, **20:**573-574.

155. Sahasranaman S, Howard D, Roy S: **Clinical pharmacology and pharmacogenetics of thiopurines.** *European journal of clinical pharmacology* 2008, **64:**753-767.

156. Tiede I, Fritz G, Strand S, Poppe D, Dvorsky R, Strand D, Lehr HA, Wirtz S, Becker C, Atreya R, et al: **CD28-dependent Rac1 activation is the molecular target of azathioprine in primary human CD4+ T lymphocytes.** *J Clin Invest* 2003, **111:**1133-1145.

157. Allan PW, Bennett LL, Jr.: **6-Methylthioguanylic acid, a metabolite of 6-thioguanine.** *Biochem Pharmacol* 1971, **20:**847-852.

158. Tay BS, Lilley RM, Murray AW, Atkinson MR: **Inhibition of phosphoribosyl pyrophosphate amidotransferase from Ehrlich ascites-tumour cells by thiopurine nucleotides.** *Biochem Pharmacol* 1969, **18:**936-938.

159. Elion GB: **The purine path to chemotherapy.** *Science* 1989, **244:**41-47.

160. Derijks LJ, Wong DR: **Pharmacogenetics of thiopurines in inflammatory bowel disease.** *Curr Pharm Des* 2010, **16:**145-154.

161. Ansari A, Arenas M, Greenfield SM, Morris D, Lindsay J, Gilshenan K, Smith M, Lewis C, Marinaki A, Duley J, Sanderson J: **Prospective evaluation of the pharmacogenetics of azathioprine in the treatment of inflammatory bowel disease.** *Alimentary pharmacology & therapeutics* 2008, **28:**973-983.

162. Ansari A, Hassan C, Duley J, Marinaki A, Shobowale-Bakre EM, Seed P, Meenan J, Yim A, Sanderson J: **Thiopurine methyltransferase activity and the use of azathioprine in inflammatory bowel disease.** *Alimentary pharmacology & therapeutics* 2002, **16:**1743-1750.

163. Campbell S, Kingstone K, Ghosh S: **Relevance of thiopurine methyltransferase activity in inflammatory bowel disease patients maintained on low-dose azathioprine.** *Alimentary pharmacology & therapeutics* 2002, **16:**389-398.

164. Dubinsky MC, Yang H, Hassard PV, Seidman EG, Kam LY, Abreu MT, Targan SR, Vasiliauskas EA: **6-MP metabolite profiles provide a biochemical explanation for 6-MP resistance in patients with inflammatory bowel disease.** *Gastroenterology* 2002, **122:**904-915.

165. Smith MA, Marinaki AM, Arenas M, Shobowale-Bakre M, Lewis CM, Ansari A, Duley J, Sanderson JD: **Novel pharmacogenetic markers for treatment outcome in azathioprine-treated inflammatory bowel disease.** *Alimentary pharmacology & therapeutics* 2009, **30:**375-384.

166. Hawwa AF, Millership JS, Collier PS, Vandenbroeck K, McCarthy A, Dempsey S, Cairns C, Collins J, Rodgers C, McElnay JC: **Pharmacogenomic studies of the anticancer and immunosuppressive thiopurines mercaptopurine and azathioprine.** *British journal of clinical pharmacology* 2008, **66:**517-528.

167. Allorge D, Hamdan R, Broly F, Libersa C, Colombel JF: **ITPA genotyping test does not improve detection of Crohn's disease patients at risk of azathioprine/6-mercaptopurine induced myelosuppression.** *Gut* 2005, **54:**565.

168. Gearry RB, Roberts RL, Barclay ML, Kennedy MA: **Lack of association between the ITPA 94C>A polymorphism and adverse effects from azathioprine.** *Pharmacogenetics* 2004, **14:**779-781.

169. Kurzawski M, Dziewanowski K, Lener A, Drozdzik M: **TPMT but not ITPA gene polymorphism influences the risk of azathioprine intolerance in renal transplant recipients.** *European journal of clinical pharmacology* 2009, **65:**533-540.

170. Marinaki AM, Ansari A, Duley JA, Arenas M, Sumi S, Lewis CM, Shobowale-Bakre el M, Escuredo E, Fairbanks LD, Sanderson JD: **Adverse drug reactions to azathioprine therapy are associated with polymorphism in the gene encoding inosine triphosphate pyrophosphatase (ITPase).** *Pharmacogenetics* 2004, **14:**181-187.

171. Schwab M, Klotz U: **Pharmacokinetic considerations in the treatment of inflammatory bowel disease.** *Clinical pharmacokinetics* 2001, **40:**723-751.

172. Eklund BI, Moberg M, Bergquist J, Mannervik B: **Divergent activities of human glutathione transferases in the bioactivation of azathioprine.** *Molecular pharmacology* 2006, **70:**747-754.

173. Seidegard J, Vorachek WR, Pero RW, Pearson WR: **Hereditary differences in the expression of the human glutathione transferase active on trans-stilbene oxide are due to a gene deletion.** *Proceedings of the National Academy of Sciences of the United States of America* 1988, **85:**7293-7297.

174. McLellan RA, Oscarson M, Alexandrie AK, Seidegard J, Evans DA, Rannug A, Ingelman-Sundberg M: **Characterization of a human glutathione S-transferase mu cluster containing a duplicated GSTM1 gene that causes ultrarapid enzyme activity.** *Molecular pharmacology* 1997, **52:**958-965.

175. Stocco G, Martelossi S, Barabino A, Decorti G, Bartoli F, Montico M, Gotti A, Ventura A: **Glutathione-S-transferase genotypes and the adverse effects of azathioprine in young patients with inflammatory bowel disease.** *Inflammatory bowel diseases* 2007, **13:**57-64.

176. Weinshilboum RM, Sladek SL: **Mercaptopurine pharmacogenetics: monogenic inheritance of erythrocyte thiopurine methyltransferase activity.** *American journal of human genetics* 1980, **32:**651-662.

177. **IMURAN drug label May 2011** [[[http://www.accessdata.fda.gov/drugsatfda_docs/label/2011/016324s034s035lbl.pdf]](http://www.accessdata.fda.gov/drugsatfda_docs/label/2011/016324s034s035lbl.pdf%5d)]

178. **PURINETHOL Drug Label May 2011** [[[http://www.accessdata.fda.gov/drugsatfda_docs/label/2011/009053s032lbl.pdf]](http://www.accessdata.fda.gov/drugsatfda_docs/label/2011/009053s032lbl.pdf%5d)]

179. **TABLOID Drug Label November 2004** [[[http://www.accessdata.fda.gov/drugsatfda_docs/label/2004/12429s022lbl.pdf]](http://www.accessdata.fda.gov/drugsatfda_docs/label/2004/12429s022lbl.pdf%5d)]

180. Higgs JE, Payne K, Roberts C, Newman WG: **Are patients with intermediate TPMT activity at increased risk of myelosuppression when taking thiopurine medications?** *Pharmacogenomics* 2010, **11:**177-188.

181. McLeod HL, Pritchard SC, Githang'a J, Indalo A, Ameyaw MM, Powrie RH, Booth L, Collie-Duguid ES: **Ethnic differences in thiopurine methyltransferase pharmacogenetics: evidence for allele specificity in Caucasian and Kenyan individuals.** *Pharmacogenetics* 1999, **9:**773-776.

182. Salavaggione OE, Wang L, Wiepert M, Yee VC, Weinshilboum RM: **Thiopurine S-methyltransferase pharmacogenetics: variant allele functional and comparative genomics.** *Pharmacogenetics and genomics* 2005, **15:**801-815.

183. Szumlanski C, Otterness D, Her C, Lee D, Brandriff B, Kelsell D, Spurr N, Lennard L, Wieben E, Weinshilboum R: **Thiopurine methyltransferase pharmacogenetics: human gene cloning and characterization of a common polymorphism.** *DNA Cell Biol* 1996, **15:**17-30.

184. Tai HL, Krynetski EY, Yates CR, Loennechen T, Fessing MY, Krynetskaia NF, Evans WE: **Thiopurine S-methyltransferase deficiency: two nucleotide transitions define the most prevalent mutant allele associated with loss of catalytic activity in Caucasians.** *American journal of human genetics* 1996, **58:**694-702.

185. Ameyaw MM, Collie-Duguid ES, Powrie RH, Ofori-Adjei D, McLeod HL: **Thiopurine methyltransferase alleles in British and Ghanaian populations.** *Human molecular genetics* 1999, **8:**367-370.

186. Collie-Duguid ES, Pritchard SC, Powrie RH, Sludden J, Collier DA, Li T, McLeod HL: **The frequency and distribution of thiopurine methyltransferase alleles in Caucasian and Asian populations.** *Pharmacogenetics* 1999, **9:**37-42.

187. Hon YY, Fessing MY, Pui CH, Relling MV, Krynetski EY, Evans WE: **Polymorphism of the thiopurine S-methyltransferase gene in African-Americans.** *Human molecular genetics* 1999, **8:**371-376.

188. Krynetski EY, Schuetz JD, Galpin AJ, Pui CH, Relling MV, Evans WE: **A single point mutation leading to loss of catalytic activity in human thiopurine S-methyltransferase.** *Proceedings of the National Academy of Sciences of the United States of America* 1995, **92:**949-953.

189. Tai HL, Krynetski EY, Schuetz EG, Yanishevski Y, Evans WE: **Enhanced proteolysis of thiopurine S-methyltransferase (TPMT) encoded by mutant alleles in humans (TPMT*3A, TPMT*2): mechanisms for the genetic polymorphism of TPMT activity.** *Proceedings of the National Academy of Sciences of the United States of America* 1997, **94:**6444-6449.

190. Wang L, Sullivan W, Toft D, Weinshilboum R: **Thiopurine S-methyltransferase pharmacogenetics: chaperone protein association and allozyme degradation.** *Pharmacogenetics* 2003, **13:**555-564.

191. Wang L, Nguyen TV, McLaughlin RW, Sikkink LA, Ramirez-Alvarado M, Weinshilboum RM: **Human thiopurine S-methyltransferase pharmacogenetics: variant allozyme misfolding and aggresome formation.** *Proceedings of the National Academy of Sciences of the United States of America* 2005, **102:**9394-9399.

192. Larovere LE, de Kremer RD, Lambooy LH, De Abreu RA: **Genetic polymorphism of thiopurine S-methyltransferase in Argentina.** *Ann Clin Biochem* 2003, **40:**388-393.

193. Lee SS, Kim WY, Jang YJ, Shin JG: **Duplex pyrosequencing of the TPMT*3C and TPMT*6 alleles in Korean and Vietnamese populations.** *Clinica chimica acta; international journal of clinical chemistry* 2008, **398:**82-85.

194. Schaeffeler E, Eichelbaum M, Reinisch W, Zanger UM, Schwab M: **Three novel thiopurine S-methyltransferase allelic variants (TPMT*20, *21, *22) - association with decreased enzyme function.** *Hum Mutat* 2006, **27:**976.

195. Schaeffeler E, Fischer C, Brockmeier D, Wernet D, Moerike K, Eichelbaum M, Zanger UM, Schwab M: **Comprehensive analysis of thiopurine S-methyltransferase phenotype-genotype correlation in a large population of German-Caucasians and identification of novel TPMT variants.** *Pharmacogenetics* 2004, **14:**407-417.

196. Dong XW, Zheng Q, Zhu MM, Tong JL, Ran ZH: **Thiopurine S-methyltransferase polymorphisms and thiopurine toxicity in treatment of inflammatory bowel disease.** *World journal of gastroenterology : WJG* 2010, **16:**3187-3195.

197. Anstey AV, Wakelin S, Reynolds NJ: **Guidelines for prescribing azathioprine in dermatology.** *Br J Dermatol* 2004, **151:**1123-1132.

198. Kaskas BA, Louis E, Hindorf U, Schaeffeler E, Deflandre J, Graepler F, Schmiegelow K, Gregor M, Zanger UM, Eichelbaum M, Schwab M: **Safe treatment of thiopurine S-methyltransferase deficient Crohn's disease patients with azathioprine.** *Gut* 2003, **52:**140-142.

199. Lennard L, Lilleyman JS, Van Loon J, Weinshilboum RM: **Genetic variation in response to 6-mercaptopurine for childhood acute lymphoblastic leukaemia.** *Lancet* 1990, **336:**225-229.

200. Oselin K, Anier K: **Inhibition of human thiopurine S-methyltransferase by various nonsteroidal anti-inflammatory drugs in vitro: a mechanism for possible drug interactions.** *Drug metabolism and disposition: the biological fate of chemicals* 2007, **35:**1452-1454.

201. Sanderson J, Ansari A, Marinaki T, Duley J: **Thiopurine methyltransferase: should it be measured before commencing thiopurine drug therapy?** *Ann Clin Biochem* 2004, **41:**294-302.

202. Lennard L, Lilleyman JS: **Individualizing therapy with 6-mercaptopurine and 6-thioguanine related to the thiopurine methyltransferase genetic polymorphism.** *Therapeutic drug monitoring* 1996, **18:**328-334.

203. Relling MV, Hancock ML, Rivera GK, Sandlund JT, Ribeiro RC, Krynetski EY, Pui CH, Evans WE: **Mercaptopurine therapy intolerance and heterozygosity at the thiopurine S-methyltransferase gene locus.** *Journal of the National Cancer Institute* 1999, **91:**2001-2008.

204. Lee D, Szumlanski C, Houtman J, Honchel R, Rojas K, Overhauser J, Wieben ED, Weinshilboum RM: **Thiopurine methyltransferase pharmacogenetics. Cloning of human liver cDNA and a processed pseudogene on human chromosome 18q21.1.** *Drug metabolism and disposition: the biological fate of chemicals* 1995, **23:**398-405.

205. Roberts RL, Gearry RB, Bland MV, Sies CW, George PM, Burt M, Marinaki AM, Arenas M, Barclay ML, Kennedy MA: **Trinucleotide repeat variants in the promoter of the thiopurine S-methyltransferase gene of patients exhibiting ultra-high enzyme activity.** *Pharmacogenetics and genomics* 2008, **18:**434-438.

206. Wang L, Pelleymounter L, Weinshilboum R, Johnson JA, Hebert JM, Altman RB, Klein TE: **Very important pharmacogene summary: thiopurine S-methyltransferase.** *Pharmacogenetics and genomics* 2010, **20:**401-405.

207. Garat A, Cauffiez C, Renault N, Lo-Guidice JM, Allorge D, Chevalier D, Houdret N, Chavatte P, Loriot MA, Gala JL, Broly F: **Characterisation of novel defective thiopurine S-methyltransferase allelic variants.** *Biochem Pharmacol* 2008, **76:**404-415.

208. Ujiie S, Sasaki T, Mizugaki M, Ishikawa M, Hiratsuka M: **Functional characterization of 23 allelic variants of thiopurine S-methyltransferase gene (TPMT*2 - *24).** *Pharmacogenetics and genomics* 2008, **18:**887-893.

209. Neuvonen PJ: **Drug interactions with HMG-CoA reductase inhibitors (statins): the importance of CYP enzymes, transporters and pharmacogenetics.** *Current opinion in investigational drugs* 2010, **11:**323-332.

210. Hochman JH, Pudvah N, Qiu J, Yamazaki M, Tang C, Lin JH, Prueksaritanont T: **Interactions of human P-glycoprotein with simvastatin, simvastatin acid, and atorvastatin.** *Pharmaceutical research* 2004, **21:**1686-1691.

211. Sakaeda T, Fujino H, Komoto C, Kakumoto M, Jin JS, Iwaki K, Nishiguchi K, Nakamura T, Okamura N, Okumura K: **Effects of acid and lactone forms of eight HMG-CoA reductase inhibitors on CYP-mediated metabolism and MDR1-mediated transport.** *Pharmaceutical research* 2006, **23:**506-512.

212. Keskitalo JE, Kurkinen KJ, Neuvoneni PJ, Niemi M: **ABCB1 haplotypes differentially affect the pharmacokinetics of the acid and lactone forms of simvastatin and atorvastatin.** *Clinical pharmacology and therapeutics* 2008, **84:**457-461.

213. Fiegenbaum M, da Silveira FR, Van der Sand CR, Van der Sand LC, Ferreira ME, Pires RC, Hutz MH: **The role of common variants of ABCB1, CYP3A4, and CYP3A5 genes in lipid-lowering efficacy and safety of simvastatin treatment.** *Clinical pharmacology and therapeutics* 2005, **78:**551-558.

214. Peters BJ, Rodin AS, Klungel OH, van Duijn CM, Stricker BH, van't Slot R, de Boer A, Maitland-van der Zee AH: **Pharmacogenetic interactions between ABCB1 and SLCO1B1 tagging SNPs and the effectiveness of statins in the prevention of myocardial infarction.** *Pharmacogenomics* 2010, **11:**1065-1076.

215. Fujino H, Saito T, Tsunenari Y, Kojima J, Sakaeda T: **Metabolic properties of the acid and lactone forms of HMG-CoA reductase inhibitors.** *Xenobiotica; the fate of foreign compounds in biological systems* 2004, **34:**961-971.

216. Bailey KM, Romaine SP, Jackson BM, Farrin AJ, Efthymiou M, Barth JH, Copeland J, McCormack T, Whitehead A, Flather MD, et al: **Hepatic metabolism and transporter gene variants enhance response to rosuvastatin in patients with acute myocardial infarction: the GEOSTAT-1 Study.** *Circulation Cardiovascular genetics* 2010, **3:**276-285.

217. Thompson JF, Man M, Johnson KJ, Wood LS, Lira ME, Lloyd DB, Banerjee P, Milos PM, Myrand SP, Paulauskis J, et al: **An association study of 43 SNPs in 16 candidate genes with atorvastatin response.** *The pharmacogenomics journal* 2005, **5:**352-358.

218. Wilke RA, Moore JH, Burmester JK: **Relative impact of CYP3A genotype and concomitant medication on the severity of atorvastatin-induced muscle damage.** *Pharmacogenetics and genomics* 2005, **15:**415-421.

219. Kivisto KT, Niemi M: **Influence of drug transporter polymorphisms on pravastatin pharmacokinetics in humans.** *Pharmaceutical research* 2007, **24:**239-247.

220. Wang D, Guo Y, Wrighton SA, Cooke GE, Sadee W: **Intronic polymorphism in CYP3A4 affects hepatic expression and response to statin drugs.** *The pharmacogenomics journal* 2011, **11:**274-286.

221. Elens L, Becker ML, Haufroid V, Hofman A, Visser LE, Uitterlinden AG, Stricker B, van Schaik RH: **Novel CYP3A4 intron 6 single nucleotide polymorphism is associated with simvastatin-mediated cholesterol reduction in the Rotterdam Study.** *Pharmacogenetics and genomics* 2011, **21:**861-866.

222. Voora D, Shah SH, Spasojevic I, Ali S, Reed CR, Salisbury BA, Ginsburg GS: **The SLCO1B1*5 genetic variant is associated with statin-induced side effects.** *Journal of the American College of Cardiology* 2009, **54:**1609-1616.

223. Prueksaritanont T, Ma B, Yu N: **The human hepatic metabolism of simvastatin hydroxy acid is mediated primarily by CYP3A, and not CYP2D6.** *British journal of clinical pharmacology* 2003, **56:**120-124.

224. Mulder AB, van Lijf HJ, Bon MA, van den Bergh FA, Touw DJ, Neef C, Vermes I: **Association of polymorphism in the cytochrome CYP2D6 and the efficacy and tolerability of simvastatin.** *Clinical pharmacology and therapeutics* 2001, **70:**546-551.

225. Nordin C, Dahl ML, Eriksson M, Sjoberg S: **Is the cholesterol-lowering effect of simvastatin influenced by CYP2D6 polymorphism?** *Lancet* 1997, **350:**29-30.

226. Geisel J, Kivisto KT, Griese EU, Eichelbaum M: **The efficacy of simvastatin is not influenced by CYP2D6 polymorphism.** *Clinical pharmacology and therapeutics* 2002, **72:**595-596.

227. Seifertova D, Basny Z, Havluj J: **[Use of electroshock for vital indications].** *Ceskoslovenska psychiatrie* 1990, **86:**321-323.

228. Donnelly LA, Doney AS, Tavendale R, Lang CC, Pearson ER, Colhoun HM, McCarthy MI, Hattersley AT, Morris AD, Palmer CN: **Common nonsynonymous substitutions in SLCO1B1 predispose to statin intolerance in routinely treated individuals with type 2 diabetes: a go-DARTS study.** *Clinical pharmacology and therapeutics* 2011, **89:**210-216.

229. Group SC, Link E, Parish S, Armitage J, Bowman L, Heath S, Matsuda F, Gut I, Lathrop M, Collins R: **SLCO1B1 variants and statin-induced myopathy--a genomewide study.** *The New England journal of medicine* 2008, **359:**789-799.

230. Tirona RG, Leake BF, Merino G, Kim RB: **Polymorphisms in OATP-C: identification of multiple allelic variants associated with altered transport activity among European- and African-Americans.** *The Journal of biological chemistry* 2001, **276:**35669-35675.

231. Nishizato Y, Ieiri I, Suzuki H, Kimura M, Kawabata K, Hirota T, Takane H, Irie S, Kusuhara H, Urasaki Y, et al: **Polymorphisms of OATP-C (SLC21A6) and OAT3 (SLC22A8) genes: consequences for pravastatin pharmacokinetics.** *Clinical pharmacology and therapeutics* 2003, **73:**554-565.

232. Ho RH, Tirona RG, Leake BF, Glaeser H, Lee W, Lemke CJ, Wang Y, Kim RB: **Drug and bile acid transporters in rosuvastatin hepatic uptake: function, expression, and pharmacogenetics.** *Gastroenterology* 2006, **130:**1793-1806.

233. Deng JW, Song IS, Shin HJ, Yeo CW, Cho DY, Shon JH, Shin JG: **The effect of SLCO1B1*15 on the disposition of pravastatin and pitavastatin is substrate dependent: the contribution of transporting activity changes by SLCO1B1*15.** *Pharmacogenetics and genomics* 2008, **18:**424-433.

234. Ho RH, Choi L, Lee W, Mayo G, Schwarz UI, Tirona RG, Bailey DG, Michael Stein C, Kim RB: **Effect of drug transporter genotypes on pravastatin disposition in European- and African-American participants.** *Pharmacogenetics and genomics* 2007, **17:**647-656.

235. Niemi M, Schaeffeler E, Lang T, Fromm MF, Neuvonen M, Kyrklund C, Backman JT, Kerb R, Schwab M, Neuvonen PJ, et al: **High plasma pravastatin concentrations are associated with single nucleotide polymorphisms and haplotypes of organic anion transporting polypeptide-C (OATP-C, SLCO1B1).** *Pharmacogenetics* 2004, **14:**429-440.

236. Mwinyi J, Johne A, Bauer S, Roots I, Gerloff T: **Evidence for inverse effects of OATP-C (SLC21A6) 5 and 1b haplotypes on pravastatin kinetics.** *Clinical pharmacology and therapeutics* 2004, **75:**415-421.

237. Pasanen MK, Neuvonen M, Neuvonen PJ, Niemi M: **SLCO1B1 polymorphism markedly affects the pharmacokinetics of simvastatin acid.** *Pharmacogenetics and genomics* 2006, **16:**873-879.

238. Kameyama Y, Yamashita K, Kobayashi K, Hosokawa M, Chiba K: **Functional characterization of SLCO1B1 (OATP-C) variants, SLCO1B1*5, SLCO1B1*15 and SLCO1B1*15+C1007G, by using transient expression systems of HeLa and HEK293 cells.** *Pharmacogenetics and genomics* 2005, **15:**513-522.

239. Niemi M, Neuvonen PJ, Hofmann U, Backman JT, Schwab M, Lutjohann D, von Bergmann K, Eichelbaum M, Kivisto KT: **Acute effects of pravastatin on cholesterol synthesis are associated with SLCO1B1 (encoding OATP1B1) haplotype *17.** *Pharmacogenetics and genomics* 2005, **15:**303-309.

240. Tachibana-Iimori R, Tabara Y, Kusuhara H, Kohara K, Kawamoto R, Nakura J, Tokunaga K, Kondo I, Sugiyama Y, Miki T: **Effect of genetic polymorphism of OATP-C (SLCO1B1) on lipid-lowering response to HMG-CoA reductase inhibitors.** *Drug metabolism and pharmacokinetics* 2004, **19:**375-380.

241. Niemi M: **Transporter pharmacogenetics and statin toxicity.** *Clinical pharmacology and therapeutics* 2010, **87:**130-133.

242. Weaver YM, Hagenbuch B: **Several conserved positively charged amino acids in OATP1B1 are involved in binding or translocation of different substrates.** *The Journal of membrane biology* 2010, **236:**279-290.

243. Morimoto K, Oishi T, Ueda S, Ueda M, Hosokawa M, Chiba K: **A novel variant allele of OATP-C (SLCO1B1) found in a Japanese patient with pravastatin-induced myopathy.** *Drug metabolism and pharmacokinetics* 2004, **19:**453-455.

244. Pasanen MK, Neuvonen PJ, Niemi M: **Global analysis of genetic variation in SLCO1B1.** *Pharmacogenomics* 2008, **9:**19-33.

245. **Simvastatin Drug Label October 2007** [[[http://www.accessdata.fda.gov/drugsatfda_docs/label/2007/021961lbl.pdf]](http://www.accessdata.fda.gov/drugsatfda_docs/label/2007/021961lbl.pdf%5d)]
